# Prevalence of stunting and its determinants among children under five in 35 Sub-Saharan African countries (2011–2024): Insights from recent demographic health survey data using a generalized linear mixed-effects model with robust poisson regression

**DOI:** 10.1371/journal.pone.0344358

**Published:** 2026-03-13

**Authors:** Abdulkerim Hassen Moloro, Kebede Gemeda Sabo, Kusse Urmale Mare, Beriso Furo Wengoro, Eshetu Elfios Endrias, Roda Mehadi Ibrahim, Teshager Dubie, Oumer Abdulkadir Ebrahim, Begetayinoral Kussia Lahole

**Affiliations:** 1 Department of Nursing, College of Medicine and Health Sciences, Samara University, Samara, Ethiopia; 2 Department of Biomedical Sciences, College of Medicine and Health Sciences, Samara University, Samara, Ethiopia; 3 Department of Nursing, School of Nursing, College of Medicine and Health Sciences, Wolaita Sodo University, Wolaita Sodo, Ethiopia; 4 Department of midwifery, College of Medicine and Health Sciences, Jigjiga University, Jigjiga, Ethiopia; 5 Department of Veterinary Medicine, College of Veterinary Medicine and Animal Sciences, Samara University, Samara, Ethiopia; 6 Department of Public Health, College of Medicine and Health Sciences, Samara University, Semera, Ethiopia; 7 Department of Midwifery, College of Medicine and Health Sciences, Arba Minch University, Arba Minch, Ethiopia; Innovative Aid, CANADA

## Abstract

**Background:**

Despite efforts by initiatives like the World Bank’s ‘All Hands-on Deck’ and UNICEF’s programs to address stunting through multisectoral approaches, the burden of stunting remains alarmingly high in sub-Saharan Africa. This study utilized recent large-scale survey data from 35 SSA countries (2011–2024) to estimate the pooled prevalence of stunting and its determinants among children under 5 years of age. Key variables such as antenatal care visits, postnatal care, and maternal nutritional indicators, which previous studies did not account for, are incorporated into the analysis.

**Methods:**

A secondary analysis was conducted using recent demographic and health survey data (2011–2024) from 35 sub-Saharan African (SSA) countries. A total weighted sample of 191,953 children under 5 years of age was included in the analysis. Descriptive and inferential analyses were performed using STATA 17. Forest plots were utilized to illustrate both pooled and country-specific stunting rates. Determinants of stunting were identified through a multilevel mixed-effects Poisson regression model with robust variance. The adjusted prevalence ratios and their 95% confidence intervals were used to assess the strength and statistical significance of associations.

**Result:**

The pooled prevalence of stunting among children under 5 years of age in 35 sub-Saharan African countries was 29.89% (95% CI: 26.63, 33.14%), with the lowest level in Gabon (13.91%) and the highest in Burundi (55.80%). Being male children (aPR = 1.24, 95% CI: 1.21–1.26), being aged 12 months or older (aPR: ≥ 1.81, p < 0.01), insufficient antenatal care (ANC) visits (aPR: ≥ 1.17, p < 0.01), lack of postnatal visits(aPR = 1.03, 95% CI: 1.07, 1.05), children perceived as small or average at birth (aPR: ≥ 1.16, p < 0.01), mother without a higher education (aPR: ≥ 1.94, p < 0.01), living in a poor or average wealth household (aPR: ≥ 1.23, p < 0.01) were significant predictors of stunting. Conversely, maternal overweight (aPR = 0.81, 95% CI: 0.77–0.84) and obese mothers (aPR = 0.88, 95% CI: 0.85–0.90) were associated lower prevalence of stunting.

**Conclusion:**

Study revealed significant country-level variations and rates exceeding 30% in 15 countries, signaling a major public health concern. The key individual, household and contextual factors associated with stunting in this study suggest the need for immediate actions expanding antenatal and postnatal care, promoting facility-based deliveries, enhancing maternal education, and media outreach. Long term strategies must tackle poverty, food systems, and equitable nutrition access, supported by governance and stability. A multisectoral approach integrating health, education, agriculture, WASH, and social protection ensures substantiable child growth, complemented by longitudinal research for policy coherence.

## Introduction

Stunting defined as a length/height-for-age measurement below −2 Standard Deviations from the median [1], remains a critical and persistent public health crisis in sub-Saharan Africa (SSA). It is the result of chronic malnutrition, a condition that compromises physical function and the maintenance of key bodily processes [2], and it leads to significant impairments in physical, psychological, and cognitive growth [3].

Sub-Saharan Africa carries a disproportionately heavy burden of childhood stunting, with pooled data from 35 countries indicating an pooled prevalence of 35% [4]. National disparities are remarkable, ranging from as low as 17% in Gabon to as high as 59% in Burundi [5], while Nigeria reports a prevalence of 36.5% among children under five [6]. This situation persists despite a global decline in stunting rates, as the absolute number of affected children in Africa has risen [7], underscoring a regional emergency within the worldwide context of 161 million stunted children [8].

The consequences in SSA are severe and lifelong, including increased child morbidity and mortality, impaired development, and higher disease risk [9,10]. Research has identified that stunting arises from a wide range of interrelated causes, which can be categorized into individual-level, household, and contextual factors. At the individual level, determinants include the child’s sex and age, maternal age and educational attainment, and birth interval [11,12]. Additional contributors such as birth order [13], antenatal care utilization [14], and duration of exclusive breastfeeding [13,15–19] have also been identified. Furthermore, studies report that inadequate food intake, low perceived birth weight, maternal body mass index (BMI), and poor sanitation significantly increase the risk of childhood stunting [20–22].

**Fig 1:**
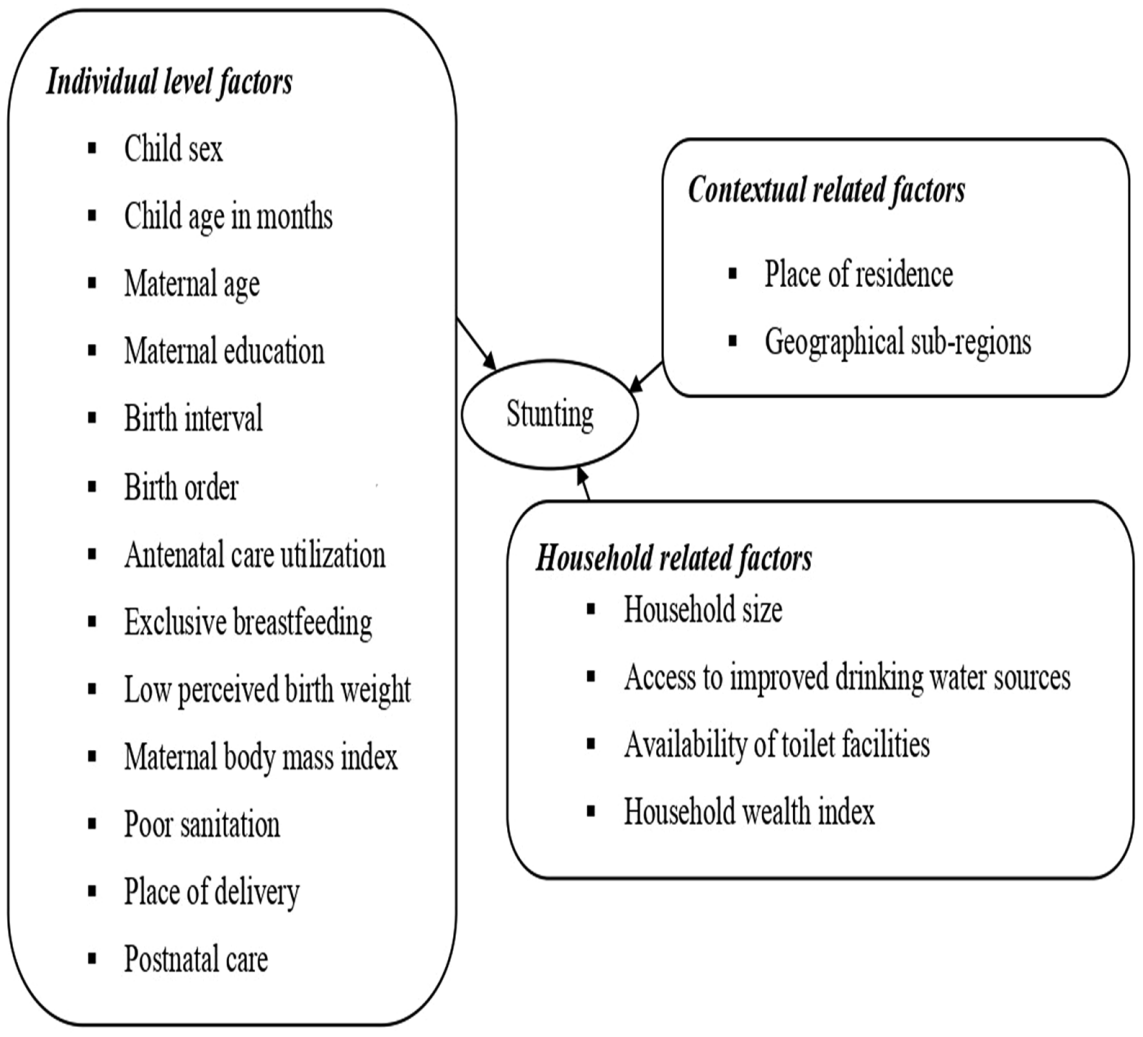
A conceptual framework showing link between stunting and its determinants among children under 5 years of age, 2011-2024.

Household-level determinants encompass household size, particularly households with multiple under-five children [14,16,23], access to improved drinking water sources [15], and availability of toilet facilities [24] and household wealth index [23,25]. Contextual factors including place of residence [14] and geographical sub-region [11,12] further affects child nutrition outcomes, underscoring the multifaceted nature of stunting and its dependence on both biological and socio-environmental conditions(Fig 1).

Stunting remains a major child health challenge in sub-Saharan Africa (SSA), despite decades of global and regional initiatives aimed at reducing malnutrition. Programs such as the World Bank’s “All Hands-on Deck” initiative, which promotes multisectoral strategies across agriculture, education, social protection, and WASH sectors, and UNICEF’s maternal and child nutrition programs have sought to accelerate progress [26,27]. Nevertheless, the burden of the stunting in SSA continues to be alarming high, and many countries are unlikely to achieve the Sustainable Developmental Goal (SSD) 2.2 target of reducing stunting by 40% by 2025 and eliminating all forms of malnutrition by 2030 [28,29].

While single country and regional studies have provided valuable insights, their scope is often limited, making it difficult to capture the broader patterns and disparities across the content. This study addresses that gap pooling nationally representative data from 35 SSA countries collected between 2011 and 2024. By combining data at this scale, the analysis enhances statistical power using Poisson regression with robust error variance [30,31] to reduce potential overestimation of odds ratios for common binary outcomes, improves generalizability, and allows for the identifications of common risk factors and cross-country variations that may not be visible in isolated studies.

Additionally, this analysis incorporated key variables such as antenatal care (ANC) visits during pregnancy, postnatal care visits, and maternal nutritional status indicators including BMI and height factors that were not considered in previous studies [4]. By integrating these variables, the pooled evidence is uniquely positioned to inform regional policy, guide resource allocation, and support collaborative interventions across countries. This study therefore provides a more comprehensive and representative picture of stunting in sub-Saharan Africa (SSA), underscoring the urgent need for coordinated, evidence-based strategies to address this persistent public health challenge. Accordingly, the study aimed to determine the pooled prevalence and key determinants of stunting among children under 5 years of age in 35 SSA countries, utilizing generalized linear mixed-effects modeling with robust Poisson regression analysis based on the most recent 2011–2024 Demographic and Health Survey (DHS) data

## Conceptual frame work

## Methods

### Study area, design, and period

This study conducted secondary analysis using data from the Demographic and Health Surveys (DHS) of 35 countries in Sub-Saharan Africa (SSA). The countries involved were Angola, Burkina Faso, Benin, Burundi, DR Congo, Republic of the Congo, Côte d’Ivoire, Cameroon, Ethiopia, Gabon, Ghana, Gambia, Guinea, Kenya, Comoros, Liberia, Lesotho, Madagascar, Mali, Mauritania, Malawi, Mozambique, Nigeria, Niger, Namibia, Rwanda, Sierra Leone, Senegal, Chad, Togo, Tanzania, Uganda, South Africa, Zambia, and Zimbabwe. The selection of country was based on the recent survey year, availability of a standardized and unrestricted dataset, and presence of observations for the outcome variable in the datasets.

The DHS surveys across all countries employed a cross-sectional study design to collect data on basic sociodemographic characteristics and various health indicators, including nutrition, maternal, and child health. For the current analysis, we included the countries that have their recent DHS conducted between 2011 and 2024. Since its inception, DHS has been conducted in over 85 low- and middle-income countries (LMICs) worldwide.

### Population, sampling technique and weight

The source population for this study comprised all children under five years old in 35 SSA countries. The study population included all children under 5 years of age in the survey. Across all countries, the surveys used a multistage stratified cluster sampling technique to select the study participants. In the first stage, each country was divided into clusters, and clusters were randomly selected based on the probability proportional to their contribution to overall country’s population. In the second stage, using the housing census as a sampling frame, a representative number of households was selected from each cluster.

To account for the complex survey design, non-response, and to ensure comparability across countries while preventing any single country or survey year from disproportionally influencing the pooled estimates, DHS sampling weights were applied as recommended. The weight variable (v005) was rescaled by dividing by 1,000,000 and incorporated into all analyses using the *svyset* command in Stata, accounting for primary sampling units (v021) and strata (v022). This produced a weighted analytic sample of **191, 953** children aged 0–59 months with complete data on the variables of interest (Table 1). Additionally, sensitivity analyses were conducted to assess the influence of individual countries by systematically excluding one country at a time and recalculating the pooled prevalence. This approach adjusts for unequal probabilities of selection and non-response within each survey, ensuring that each country’s contribution to the pooled prevalence is proportional to its population size.

**Table 1:** Survey years and sample sizes of children under 5 years of age from 35 Sub-Saharan African (SSA) countries included in the study, from 2011 to 2024.

Country	Survey year	Unweighted sample size	Weighted sample size
Angola	2023-2024	5,039	4,800
Burkina Faso	2021	5,731	5,696
Benin	2017-2018	11,670	11,717
Burundi	2016-2017	6,048	6,222
DR Congo	2023-2024	10,745	9,734
Congo	2011-2012	4,475	3,859
Cote d’Ivoire	2021	4,779	4,300
Cameroon	2018-2019	4,497	4,707
Ethiopia	2019	5,076	4,972
Gabon	2019-2021	5,343	5,009
Ghana	2022-2023	4,405	4,051
Gambia	2019-2020	3,811	3,511
Guinea	2018	3,466	3,374
Kenya	2022	17,327	15,336
Comoros	2012	2,387	2,468
Liberia	2019-2020	2,445	2,166
Lesotho	2023-2024	1,093	983
Madagascar	2021	5,778	5,719
Mali	2023-2024	13,936	14,163
Mauritania	2019-2021	9,830	9,955
Malawi	2015-2016	5,149	5,160
Mozambique	2022-2023	3,733	3,858
Nigeria	2023-2024	9,410	9,341
Niger	2012	5,576	5,112
Namibia	2013	1,558	1,438
Rwanda	2019-2020	3,809	3,918
Sierra Leone	2019	4,136	4,044
Senegal	2023	4,476	4,258
Chad	2014-2015	9,826	9,805
Togo	2013-2014	3,185	3,058
Tanzania	2022	4,807	4,866
Uganda	2016	4,423	4,350
South Africa	2016	1,113	1,080
Zambia	2024	3,810	3,710
Zimbabwe	2015	4,957	5,212
Total		**197,044**	**191,953**

### Data source

The data for this study were obtained from the DHS women’s questionnaire, focusing on children under five years of age (under 60 months), and from the Kids Record dataset (KR file) across 35 countries. All datasets were sourced directly from the official Demographic and Health Surveys (DHS) program website (https://dhsprogram.com/).

### Data extraction and management of missing observations

Prior to data extraction, we identified countries with DHS datasets from surveys conducted between 2011 and 2024, as our aim was to include only those with data collected during this timeframe. A standardized data collection tool and face-to-face interview were used to collect the survey’s data. To construct a pooled dataset, we first extracted variables relevant to the study from the data of the children under 5 years of age (under 60 months) from the Kids Record dataset (KR file) across 35 countries. The merged datasets from 35 sub-Saharan African countries were then appended by survey year, creating a pooled datasets for regional analysis of child stunting. Country identifiers and survey weights were retained to account for sampling design and cross-country differences. Variables are then recoded and categorized consistently across all 35 DHS surveys using the Guide to DHS statistics to ensure compatibility.

The handling of anthropometric data in this analysis adhered to the standardized protocols outlined in the Guide to DHS Statistics [32]. Children were excluded or dropped from the analyses of all anthropometric indices if they met any of the following criteria: (i) they were not weighed or measured during the survey, (ii) their recorded weight or height measurements were missing, (iii) their month or year of birth was unknown or missing (rendering accurate age calculation impossible for indices requiring it), or (iv) their derived height-for-age z-scores (HAZ) were flagged as biologically implausible (e.g., outside the range of −6 to +6 SD). These exclusions applied uniformly to both the denominator and numerator in prevalence analysis, constituting a complete-case analysis.

For the independent variables, complete case analysis was employed, retaining only observations with non-missing values for all covariates included in the final model. These steps enhance the transparency and validity of our findings in light of potential cross-country variations in data completeness. A detailed explanation of the DHS methodology, guidelines, and procedures for handling missing data is available on the DHS website [33].

### Variables and measurements

#### Dependent Variable.

**Stunting (height-for-age z-scores):** height for age of children dichotomized as normal (not stunted) if height for age ≥ −2 SD and stunted if height of age < −2 SD form WHO child growth reference ((height/age standard deviation (new who)). Weight measurements were obtained using light weight SECA mother-infant scales with a digital screen designed and manufactured under the guidance of UNICEF. Height measurements were obtained using the Shorr measuring board. Children younger than 24-months were measured for their height while lying down, and children older than 24 months were measured while standing.

**Independent Variables:** A total of 23 variables were incorporated into this study, selected through a systematic process that considered both their availability in the Demographic and Health Survey (DHS) and their documented associations with childhood stunting in prior research [34–36]. Each variable was chosen for its relevance to the study objectives and its role as a known or hypothesized determinants of stunting reflecting influences at individual, household, and contextual level. This study also included variables often overlooked in earlier work, such as antenatal care (ANC) visits during pregnancy postnatal care utilizations, and body mass index.

These variables were categorized into individual-level factors, household factors, and contextual factors. The individual-level factors included the sex of the child (male and female), age of the child in month, birth order (1, 2–4, and 5+), breastfeeding (still breastfeeding, ever breastfed and never breastfed) and perceived size at birth (large, average, and small). Other individual-level factors were maternal educational level (no formal education, primary, secondary, and higher), current maternal working status (yes and no), number of antenatal care visits during pregnancy (0, 1–3, and 4 or more) and postnatal checks within 2 months (yes and no). Variables such as maternal age (15 –24 and 25–49), age at first birth, antenatal visits during pregnancy (0, 1–3, and 4 or more), place of delivery (home, health facility, other), marital status (single and married) and maternal nutritional status were also considered.

Household factors included household size (small, medium, and large), exposure to media (yes and no), and wealth index (poor, middle, rich). Contextual factors encompassed the place of residence (urban and rural) and geographical sub-regions (West, East, Central, and Southern).

Maternal nutritional status, described using Body Mass Index (BMI) according to the WHO adult BMI classification [37]. BMI was calculated by dividing each woman’s weight in kilograms by the square of her height in meters (kg/m²). The survey utilized an electronic SECA 874 flat scale for weight measurements, specially designed for mobile use, and a shore measuring board for height. For analysis, women were categorized into four nutritional status groups based on their BMI: underweight (BMI < 18.5 kg/m²), normal weight (BMI 18.5–24.9 kg/m²), overweight (BMI 25.0–29.9 kg/m²), and obesity (BMI ≥ 30.0 kg/m²) [38].

### Data management and statistical analysis

Stata version 17 was utilized for data cleaning and analysis. Prior to the analysis, the presence of the outcome variable in the DHS dataset for each country was confirmed. All the variables considered in the study were reviewed for missing values. Subsequently, the datasets from 35 SSA countries were appended and weighted to maintain sample representativeness and obtain reliable estimates and standard errors. The pooled prevalence of child stunting was calculated using weighted data on the number of affected children with outcome variable and the total number of study participants in each country included in the analysis. The Stata command for meta-analysis “metan” was executed to present the country-specific and pooled estimates with 95% CI in a forest plot. To explore potential sources of heterogeneity, meta-regressions were conducted [39]. Additionally, sensitivity analyses were performed to evaluate the influence of individual country on the overall pooled estimates [39].

A multilevel mixed-effects Poisson regression model with robust error variance was fitted to identify determinants of stunting among children under five in SSA. This model was selected for two primary reasons. First, it directly estimates prevalence ratios, which are more interpretable and avoid the overestimation of association strength common with odds ratios from logistic regression when applied to common binary outcomes in cross-sectional data [30,31]. Second, its multilevel framework accounts for the hierarchical structure of the DHS data, where children are nested within households, and households within clusters, by including random intercepts at the household and cluster levels. Bivariable multilevel robust Poisson regression analysis was done and all variables with a p-value of less than 0.25 in this analysis were considered for multivariable multilevel robust Poisson regression model [30,31].

Prior to model fitting, we assessed the key assumption of the Poisson regression model. A key assumption of this model is that observations are independent and that the mean and variance of the dependent variable are equal. Model adequacy was then confirmed through goodness-of-fit tests: the deviance statistic (151,692.69) and Pearson statistic (83,880.11) both yielded p-values of 1.000, indicating an excellent fit. The data had a mean of 0.306 and a variance of 0.294, producing a mean-to-variance ratio of approximately 1.04. This close alignment supports the equidispersion assumption and validates the use of the Poisson model. Based on these findings, we applied Poisson regression to examine the effects of predictors on stunting, while carefully monitoring for influential observations and residual patterns [40]. Model fit was further assessed using Akaike (AIC) and Bayesian (BIC) Information Criteria, with lower values indicating better fit and parsimony.

In our analysis, five hierarchal models were fitted to select the model that best fits the data: null-model (a model with only outcome variable to assess the random variability in the intercept), model-I (a model with only individual-level explanatory variables), model-II (a model with only household-level explanatory variables), and model-III (a model with contextual-level factors) and model-IV (a model with only potential candidate variables from individual, household, and contextual-level factors). The “ meglm” command in Stata was used to fit these models.

Random variability in stunting across clusters was assessed by intra-class correlation coefficient (ICC), explained variance or proportion change in variance (PCV), and median odds ratio (MOR). Akaike’s information criteria (AIC), Bayesian information criteria (BIC), Log-likelihood (LL), and deviance (i.e.,-2*LL) values were used for model comparison. The presence of multicollinearity between explanatory variables was checked using variance inflation factor values and the values for the included variables ranged from **1.01 to 1.95,** suggesting that there was no multi-collinearity. Finally, in the multivariable analysis, a p-value less than 0.05 and an adjusted prevalence ratio with the corresponding 95% confidence interval was used to identify the factors associated with stunting among children under 5 years of age in 35 SSA countries.

### Ethical considerations

For this study, we utilized Demographic and Health Survey (DHS) data from 35 sub-Saharan African countries. The DHS survey procedures were approved by the ICF Institutional Review Board (IRB) and the respective host country IRB; therefore, no additional ethical approval was required for this secondary analysis. The dataset accessed contain no identifiable participant information, ensuring confidentiality and privacy. Access to the data was formally authorized by the DHS program, which serve as the institutional custodian of the datasets, through an online request submitted at http://www.dhsprogram.com and supported by authorization letter (AuthLetter_215093) on dated 01/19/2025.

## Results

The study included and analyzed a weighted sample of 191,953 mothers with children aged 0–59 months. Of these children, 50.49% (96,907) were male, and 20.01% (42,185) were under 12 months old. More than half of the children, 55.34% (96,976), had been breastfed at some point but were no longer breastfeeding. Stunting was highly prevalent, affecting 21.98% (42,185) of children under 12 months, compared to older age groups. Among the included children, 65.63% (125,969) lived in rural areas, and 53.29% (25,499) were from households in the poor wealth index category. Additionally, 42.23% (81,068) of the children were from West African countries, and 31.51% (56,237) were delivered at home (Table 2).

**Table 2:** Sociodemographic characteristic of the children under 5 years of age by stunting and their overall background status, in 35 SSA countries, 2011–2024 (n = 191,953).

Characteristics	Weighted Frequency (%)	Stunting
**Sex of the child**		
Male	96,907(50.49)	31,604(32.61)
Female	95,045(49.51)	26,642(28.03)
**Age of the child (months)**		
Less than 12 months	42,185(21.98)	7,156(16.96)
12–23 months	39,907(20.79)	13,492(33.81)
24–35 months	36,961(19.26)	14,225(38.49)
36–47 months	37,708(19.64)	13,108(34.76)
48–59 months	35,190(18.33)	10,264(29.17)
**Birth order**		
First	41,755(21.75)	11,640(27.88)
2–4	94,840(49.41)	27,615(29.12)
5 or higher	55,358(28.84)	18,991(34.31)
**Breastfeeding**		
Ever breastfeed	96,976(55.34)	34,331(35.40)
Never breastfeed	6,870(3.92)	2,149(31.28)
Still breastfeeding	71,397(40.74)	18,261(25.58)
**Perceived size at birth**		
Large	53,478(32.87)	15,044(28.13)
Average	81,999(50.40)	25,391(30.97)
Small	27,219(16.73)	10,537(38.71)
**Mothers’ educational status**		
No education	71,108(37.04)	25,867(36.38)
Primary	61,015(31.79)	20,175(33.07)
Secondary	51,005(26.57)	11,385(22.32)
Higher	8,824(4.60)	818(9.28)
**Mother currently working**		
Yes	109,618(77,227)	33,980(31.00)
No	77,227(41.33)	22,379(28.98)
**Mother’s age (years)**		
15-24	52,209(27.20)	16,514(31.63)
25-49	139,744(72.80)	41,732(29.86)
**Number of ANC visits**		
No visit	12,189(6.35)	4,919(40.36)
1 to 3 visits	39,646(20.65)	12,762(32.19)
4 and above visit	140,117(73.00)	40,565(28.95)
**Place of delivery**		
Home	56,237(31.51)	16,649(38.46)
Health facility	122,213(69.64)	38,187(61.54)
Postnatal care visits		
Yes	44,774(34.71)	11,787(26.33)
No	84,227(65.29)	25,394(30.15)
**Marital status**		
Single	42,811(22.30)	12,499(29.20)
Married	149,142(77.70)	45,747(30.67)
**Mothers age at 1st birth**		
Less than 20	110,643(57.64)	36,334(32.84)
20 and above	81,309(42.36)	21,912(26.95)
**Maternal nutritional status**		
Underweight	12,591(6.56)	5,131(40.76)
Normal weight	91,612(47.73)	31,342(34.21)
Overweight	26,149(13.62)	6,036(23.08)
Obese	61,600(32.09)	15,736(25.55)
**Household size**		
Small	16,665(8.68)	4,882(29.30)
Medium	12,723(6.63)	3,719(29.23)
Large	162,563(84.69)	49,644(30.54)
**Mass media exposure**		
No	65,415(34.08)	25,077(38.34)
Yes	126,538(65.92)	33,169(26.21)
**Wealth index**		
Poor	84,032(43.78)	25,499(53.29)
Middle	38,451(20.03)	11,668(20.53)
Rich	69,471(36.19)	21,080(28.18)
**Place of residence**		
Urban	65,983(34.37)	14,229(21.57)
Rural	125,969(65.63)	44,017(34.94)
**Subregion**		
West African Countries	81,068(42.23)	22,671(27.97)
East African Countries	47,706(24.85)	14,833(31.09)
Central African Countries	37,024(19.29)	12,289(33.19)
Southern African Countries	26,154(13.63)	8,452(26,154)

Of the 191,953 mothers included in the study, 139,744 (72.80%) were between 25 and 49 years old, and 149,142(77.70%) were currently married. Over one-third of the mothers, 71,108(37.04%), had no formal education, while only 8,824(4.60%) had completed higher education. The majority, 110,643(57.64%), had their first child before the age of twenty, and 126,538 (65.92%) had access to mass media. Regarding nutritional status, 12,591(6.56%) of the mothers were underweight, while 91,612(47.73%) had a body mass index (BMI) within the normal range. Additionally, 26,149 (13.62%) were overweight, and 61,600(32.09%) were classified as obese

### Pooled prevalence of stunting among children under 5 years of age in sub–Saharan African

**Fig 2:**
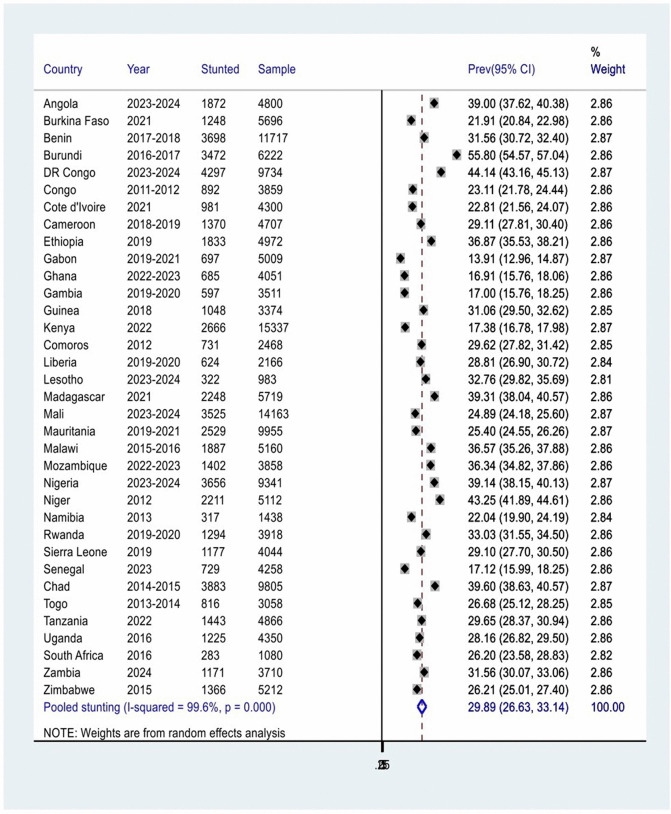
Forest plot of the country-level and pooled prevalence of stunting among children under 5 years of age across 35 SSA countries, 2011–2024.

The pooled prevalence of stunting among children under 5 years of age in 35 sub-Saharan African (SSA) countries was 29.89% (95% CI: 26.63, 33.14%), with significant variation observed across countries (I^2^ = 99.6, P-value = 0.000) (Fig 2). Gabon had the lowest stunting prevalence at 13.91%, while Burundi reported the highest at 55.80%. Among the 35 countries analyzed, 15 had a stunting prevalence of 30% or higher among children under 5 years of age.

### Handling heterogeneity

The random-effects model revealed considerable heterogeneity. To address this, sensitivity analysis, subgroup analysis, and meta-regression were conducted.

### Sensitivity analysis

**Fig 3:**
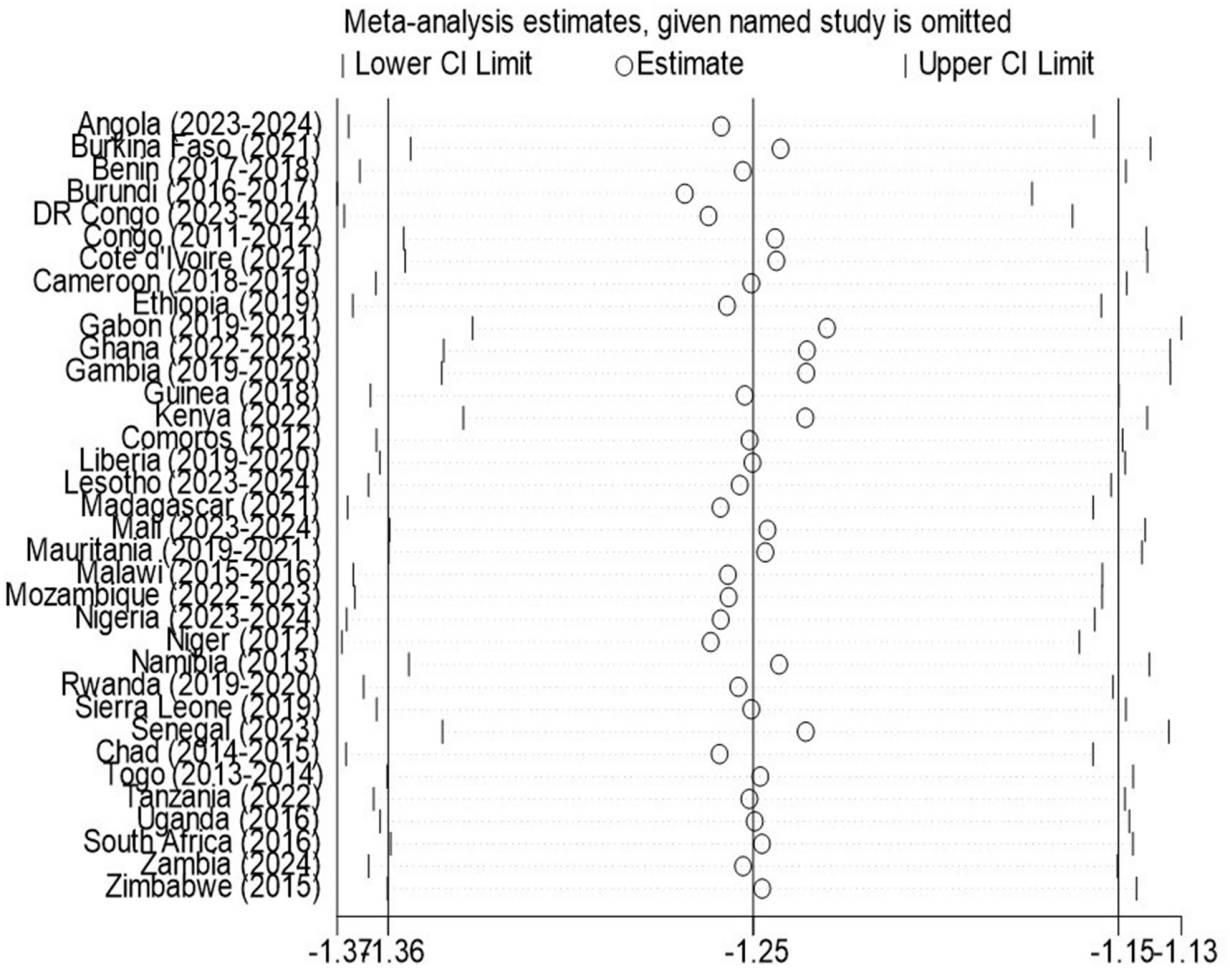
Sensitivity analysis plot forest plot of the country-level and pooled prevalence of stunting among children under 5 years of age across 35 SSA countries, 2011–2024.

Sensitivity analysis was performed to evaluate the eﬀect of individual country on the pooled estimated. When individual country was omitted, the pooled prevalence obtained was within the 95% CI of the overall pooled prevalence. This confirms the absence of single study impact on the overall pooled eﬀect size. Therefore, from the random effects model, there were no country that excessively influence the overall pooled estimate of stunting (Fig 3).

### Sub-group analysis

Subgroup analyses were performed by sub-region, and year of publication.

### Stunting by Sub-Region

**Fig 4:**
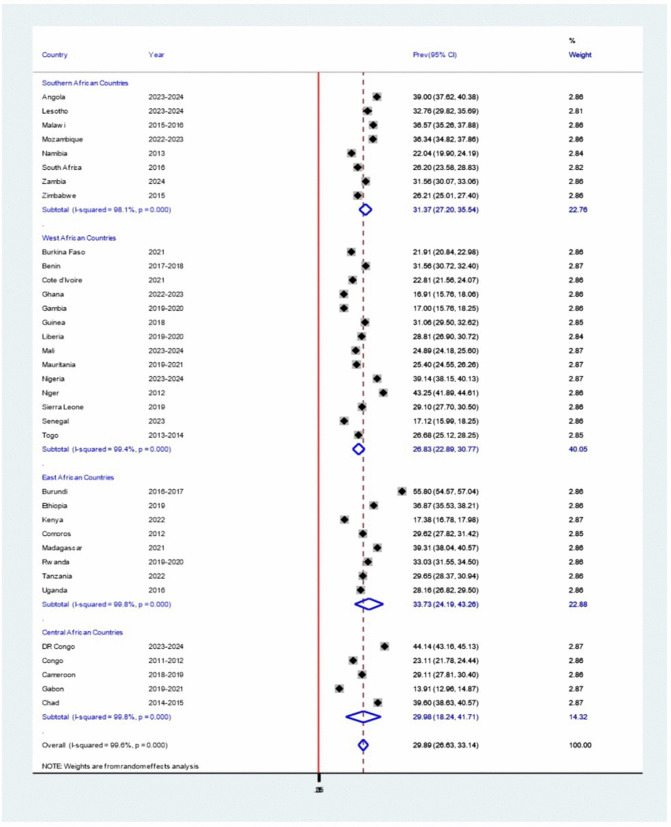
Forest plot showing the pooled prevalence of stunting among children under 5 years of age in sub-Saharan Africa, by subregion, based on a random-effects model analysis, 2011-2024.

Subgroup analysis by sub-region indicated that Est African countries had the highest pooled prevalence of stunting among children under 5 years of age (33.73%, 95% CI: 24.19–43.26), followed by Southern African countries (31.37%, 95% CI: 27.20–35.54), Central African countries (29.98%, 95% CI: 18.24–41.71) and West African countries (26.83%, 95% CI: 22.89–30.77). Substantial heterogeneity was observed in East and Central Africa (I² = 99.8%, P = 0.00), West Africa (I² = 99.4%, P = 0.00), and Southern Africa (I² = 98.1%, P = 0.00) (Fig 4).

### Stunting by Year of Survey

**Fig 5:**
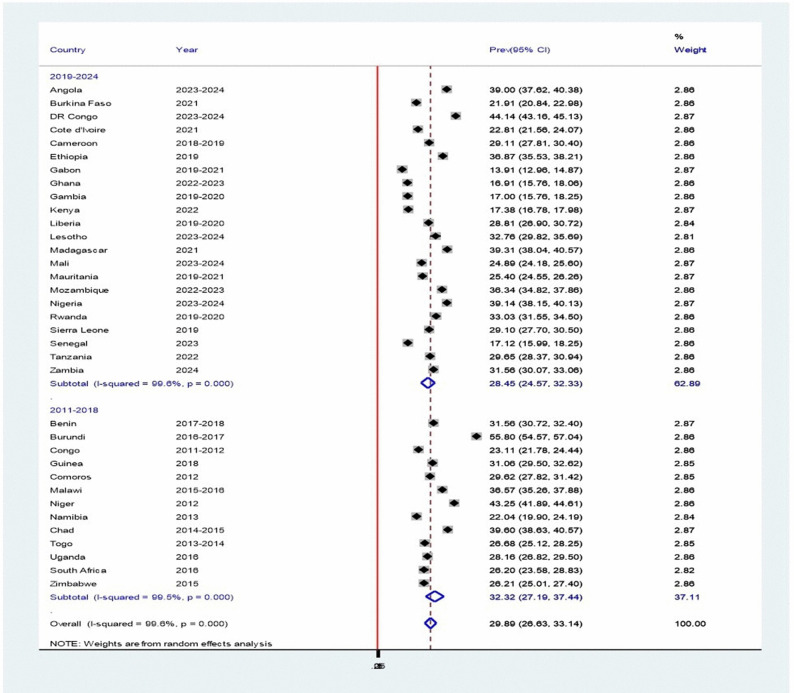
Forest plot showing the pooled prevalence of stunting among children under 5 years of age in sub-Saharan Africa, by survey year category, based on a random-effects model analysis, 2011-2024.

Subgroup analysis by year of survey showed that the pooled prevalence of stunting was highest in 2011–2018 (32.32%, 95% CI: 27.19–37.44) and lowest in 2019–2024 (28.45%, 95% CI: 24.57–32.33). Both periods demonstrated significant heterogeneity (2011–2018: I² = 99.5%, p < 0.00; 2020–2024: I² = 99.6%, p < 0.00) (Fig 5).

### Meta-regression

A meta-regression was performed to assess whether the year of survey, sample size, country, or sub-region could explain the heterogeneity in stunting prevalence (Table 3). None of these variables were statistically significant predictors of heterogeneity (all p-values > 0.05). The high residual heterogeneity in the pooled prevalence of stunting suggests that other unmeasured factors are responsible for the variability across studies

**Table 3:** Meta-regression for the studies of pooled stunting among children under 5 years of age in 35 sub-Sharan Africa, 2011-2024.

Outcome categories	Variables	Coefficients	P-value	[95% conf. interval]
*Pooled stunting among under five children*	Year of Survey	0.99	0.68	0.96, 1.02
	Sample size	1.00	0.65	0.99, 1.00
	Countries	0.99	0.22	0.98, 1.00
	Sub-region	1.06	0.19	0.96, 1.17

### Results of random-effects analysis (measures of variation)

The ICC (Intraclass Correlation Coefficient) value from the null model revealed that only about 26.8% of the total variance in stunting among under five children is attributable to differences between clusters. The remaining 73.2% of the variance reflects variation occurring within clusters, which encompasses individual, household, and broader contextual factors. This indicates that while cluster-level differences play a substantial role, the majority of the variability in stunting arises from determinants operating at multiple levels within clusters. In the final model (Model IV), the explained variance values demonstrated that around 47.2% of the total variation in the pooled prevalence of stunting among children under 5 years of age was influenced by the combined effects of individual level factors, household level factors and contextual factors-level factors.

Additionally, the presence of heterogeneity in stunting across clusters was supported by the MOR (Median Odds Ratio) values of 1.96 and 1.46 in the null and full models, respectively. This suggests that children under 5 years of age in clusters with higher pooled prevalence of stunting had approximately 1.96 times greater odds of stunting compared to those in clusters with lower levels of pooled prevalence of stunting. Model 4 was identified as the best-fitted model, as it exhibited the lowest AIC (Akaike Information Criterion), BIC (Bayesian Information Criterion), and deviance values (Table 4).

**Table 4:** Selection of most parsimonious model (measures of variation) at cluster or community level for stunting among children under 5 years of age in 35 SSA countries, 2011–2024.

Measure of variation	Models				
	Null-Model	Model I	Model II	Model III	Model IV
Cluster-level variance (SE)	0.5(0.05)	0.15(0.001)	0.2(0.03)	0.3(0.04)	0.16(0.025)
Intra-class correlation (%) or VPC	26.8%	13.8%	12.7%	11.4%	10.2%
Explained variance (%) or PCV	***Reference***	70%	60%	40%	47.2%
Median odds ratio	1.96	1.44	1.5	1.68	1.46
Model summary					
Akaike’s information criteria	245122.6	138191.7	169678.7	241377.5	**98254.67**
Bayesian information criteria	245153.1	138453.6	169807	241448.8	**98639.09**
Log-likelihood	−122558.28	−69068.872	−84826.365	−120681.75	**−49086.333**
Deviance	245116.55	138137.74	169652.73	241363.5	**98172.666**

***VPC*** *= Variance Partitioning Coefficient,* ***PCV*** *= Proportion Change in Variance*

### Determinants of stunting among children under 5 years of age in 35 sub–Saharan African Countries

Our analysis showed that the prevalence of stunting was 1.24 times higher among male children compared to female children (aPR = 1.24, 95% CI: 1.21–1.26). Using children under 12 months of age as the reference category, the prevalence of stunting was 2.07 times higher among those aged 12–23 months (aPR = 2.07, 95% CI: 2.00–2.14). It was 2.41 times higher among children aged 24–35 months (aPR = 2.41, 95% CI: 2.32–2.50), 2.32 times higher among those aged 36–47 months (aPR = 2.32, 95% CI: 2.23–2.42), and 1.81 times higher among children aged 48–59 months (aPR = 1.81, 95% CI: 1.71–1.90) (Table 5).

**Table 5:** Bivariable and multivariable multilevel mixed-effects robust Poisson regression analysis of determinants of stunting among children under 5 years of age in 35 SSA countries, 2011–2024.

Covariates	Stunting		cPR with 95%	aPR with 95%
	Yes	No		
**Sex of the child**				
Male	31,604(32.61)	65,303(67.39)	1.16(1.14, 1.18) *	1.24(1.21, 1.26) **
Female	26,642(28.03)	68,402(71.97)	1	1
**Age of the child (months)**				
Less than 12 months	7,156(16.96)	35,029(83.04)	1	1
12–23 months	13,492(33.81)	26,415(66.19)	1.99(1.93, 2.06) *	2.07(2.00, 2.14) **
24–35 months	14,225(38.49)	22,736(61.51)	2.26(2.19, 2.34) *	2.41(2.32, 2.50) **
36–47 months	13,108(34.76)	24,599(65.24)	2.05(1.98, 2.12) *	2.32(2.23, 2.42) **
48–59 months	10,264(29.17)	24,926(70.83)	1.72(1.66, 1.78) *	1.81(1.71, 1.90) **
**Birth order**				
First	11,640(27.88)	30,115(72.12)	1	1
2–4	27,615(29.12)	67,224(70.88)	1.04(1.01, 1.06) *	1.01(0.98, 1.05)
5 or higher	18,991(34.31)	36,367(65.69)	1.21(1.18, 1.24) *	1.08(1.03, 1.12) **
**Perceived size at birth**				
Large	15,044(28.13)	38,434(71.87)	1	1
Average	25,391(30.97)	56,608(69.03)	1.10(1.07, 1.12) *	1.16(1.13, 1.19) **
Small	10,537(38.71)	16,682(61.29)	1.37(1.34, 1.41) *	1.48(1.43, 1.52) **
**Mothers’ educational status**				
No education	25,867(36.38)	45,241(63.62)	3.84(3.49, 4.22) *	1.94(1.74, 2.18) **
Primary	20,175(33.07)	40,839(66.93)	3.49(3.17, 3.84) *	1.95(1.74, 2.19) **
Secondary	11,385(22.32)	39,619(77.68)	2.37(2.15, 2.61) *	1.68(1.51, 1.88) **
Higher	818(9.28)	8,006(90.72)	1	1
**Mother’s age (years)**				
15-24	16,514(31.63)	35,694(68.37)	1.05(1.03, 1.07) *	1.09(1.06, 1.13) **
25-49	41,732(29.86)	98,012(70.14)	1	1
**Number of ANC visits**				
No visit	4,919(40.36)	7,269(59.64)	1.36(1.32, 1.41) *	1.17(1.13, 1.21) **
1 to 3 visits	12,762(32.19)	26,884(67.81)	1.10(1.08, 1.12) *	1.11(1.09, 1.14) **
4 and above visit	40,565(28.95)	99,552(71.05)	1	1
**Place of delivery**				
Home	16,649(38.46)	32,199(26.68)	1.44(1.41, 1.47) *	1.11(1.08, 1.14) **
Health facility	38,186(61.54)	88,466(73.32)	1	1
**Postnatal care visits**				
Yes	11,787(26.33)	32,986(73.67)	1	1
No	25,394(30.15)	58,832(69.85)	1.14(1.11, 1.17) *	1.03(1.07, 1.05) **
**Marital status**				
Single	12,499(29.20)	30,311(70.80)	0.94(0.92, 0.96) *	1.04(1.02, 1.07) **
Married	45,747(30.67)	103,39(69.33)	1	1
**Mothers age at 1st birth**				
Less than 20	36,334(32.84)	74,309(67.16)	1.20(1.18, 1.22) *	1(0.97, 1.02)
20 and above	21,912(26.95)	59,397(73.05)	1	1
**Maternal nutritional status**				
Underweight	5,131(40.76)	7,459(59.24)	1.19(1.15, 1.22) *	1.12(1.08, 1.16) **
Normal weight	31,342(34.21)	60,270(65.79)	1	1
Overweight	6,036(23.08)	20,113(76.92)	0.68(0.66 0.70) *	0.81(0.77, 0.84) **
Obese	15,736(25.55)	45,863(74.45)	0.75(0.73, 0.77) *	0.88(0.85, 0.90) **
**Household size**				
Small	4,882(29.30)	11,782(70.70)	1	1
Medium	3,719(29.23)	9,004(70.77)	1(0.95, 1.04)	0.99(0.94, 1.05)
Large	49,644(30.54)	112,919(69.46)	1.04(1.01, 1.07) *	1.04(1.04, 1.08) **
**Mass media exposure**				
No	25,077(38.34)	40,337(61.66)	1.44(1.41, 1.47) *	1.11(1.08, 1.13) **
Yes	33,169(26.21)	93,368(73.79)	1	1
**Wealth index**				
Poor	16,637(38.34)	52,993(39.63)	2.26(2.17, 2.36) *	1.31(1.24, 1.38) **
Middle	11,958(31.10)	26,492(19.81)	1.83(1.75, 1.91) *	1.23(1.17, 1.29) **
Richer	9,793(26.42)	54,221(40.55)	***1***	***1***
**Place of residence**				
Urban	14,229(21.57)	51,754(78.43)	1	1
Rural	44,017(34.94)	81,952(65.06)	1.61(1.56, 1.66) *	1.07(1.04, 1.11) **

***cPR****: Crude Prevalence Ratio,* ***aPR***: Adjusted Prevalence Ratio, *: Significant in crude prevalence ratio, **: Significant in adjusted prevalence ratio

Children born in the fifth or higher birth order had an 8% higher prevalence of stunting compared to those born as first-order children (aPR = 1.08, 95% CI: 1.03–1.12). Using children perceived as large at birth as the reference category, the prevalence of stunting was 16% higher among those perceived as average size at birth (aPR = 1.16, 95% CI: 1.13–1.19) and 48% higher among those perceived as small size at birth (aPR = 1.48, 95% CI: 1.43–1.52). Compared to children whose mothers had a higher level of formal education, the prevalence of stunting was 1.94 times higher among children whose mothers had no formal education (aPR = 1.94, 95% CI: 1.74–2.18), 1.95 times higher among those whose mothers had only primary education (aPR = 1.95, 95% CI: 1.74–2.19), and 1.68 times higher among those whose mothers had secondary education (aPR = 1.68, 95% CI: 1.51–1.88).

Using mothers aged 25 years and above as the reference category, the prevalence of stunting was 9% higher among children of mothers aged 15–24 years (aPR = 1.09, 95% CI: 1.06–1.13). Compared to children whose mothers had four or more antenatal care (ANC) visits, the prevalence of stunting was 17% higher among those whose mothers had no ANC visits (aPR = 1.17, 95% CI: 1.13–1.21) and 11% higher among those whose mothers had only one to three visits (aPR = 1.11, 95% CI: 1.09–1.14). In addition, children delivered at home had an 11% higher prevalence of stunting than those delivered in health facilities (aPR = 1.11, 95% CI: 1.08–1.14). Children whose mothers did not attend postnatal visits had a 3% higher prevalence of stunting compared with those whose mothers attended such visits (aPR = 1.03, 95% CI: 1.01–1.05). Moreover, the prevalence of stunting was 1.04 times higher among children of single mothers than among those of married mothers (aPR = 1.04, 95% CI: 1.02–1.07).

This study revealed that maternal BMI was significantly associated with childhood stunting. Compared to children of mothers with a normal BMI, the prevalence of stunting was 12% higher among children of underweight mothers (aPR = 1.12, 95% CI: 1.08–1.16). In contrast, the prevalence of stunting was 19% lower among children of overweight mothers compared to those of mothers with a normal BMI (aPR = 0.81, 95% CI: 0.77–0.84). Similarly, the prevalence of stunting was 12% lower among children of obese mothers compared to those of mothers with a normal BMI (aPR = 0.88, 95% CI: 0.85–0.90).

This study revealed that household size was significantly associated with childhood stunting. Compared to children from smaller households, the prevalence of stunting was 4% higher among children from large households (aPR = 1.04, 95% CI: 1.04–1.08). In addition, children whose mothers reported no exposure to mass media such as television, radio, or newspapers had an 11% higher prevalence of stunting compared to children whose mothers had some media exposure (aPR = 1.11, 95% CI: 1.08–1.13).

Moreover, compared to children from rich households, the prevalence of stunting was 31% higher among children from poor households (aPR = 1.31, 95% CI: 1.24–1.38). Similarly, the prevalence of stunting was 23% higher among children from middle wealth index households compared to those from rich households (aPR = 1.23, 95% CI: 1.17–1.29). Furthermore, children living in rural areas had a 7% higher prevalence of stunting compared to their urban counterparts (aPR = 1.07, 95% CI: 1.04–1.11).

## Discussion

Reducing stunting in sub-Saharan Africa (SSA) continues to pose a significant challenge, yet various programmatic initiatives are being introduced to drive progress. The World Bank’s “All Hands-on Deck” initiative advocates for a multisectoral strategy, involving sectors such as agriculture, education, social protection, and water, sanitation, and hygiene (WASH) [41]. Similarly, UNICEF’s programs prioritize maternal, infant, and young child nutrition, micronutrient supplementation, the management of severe acute malnutrition, nutrition in emergencies, and enhancing water and sanitation at the community level [42]. Despite these efforts, the burden of stunting remains high in sub-Saharan African countries. These integrated approaches are specifically designed to accelerate stunting reduction in SSA, with a particular emphasis on East Africa, where the challenge persists [4].

This study analyzed pooled data from nationally representative surveys conducted across 35 sub-Saharan African (SSA) countries between 2011 and 2024. The findings revealed that the overall pooled prevalence of stunting among children under 5 years of age in SSA was 29.89% (95% CI: 26.63, 33.14%). The lowest stunting prevalence rate was recorded in Gabon at 13.74%, while the highest prevalence of stunting was observed in Burundi at 55.74%. Among the 35 countries analyzed, 14 had a stunting prevalence of 30% or higher among children under 5 years of age. This finding is consistent with World Health Organization prevalence threshold or cut-off values for public health significance in which prevalence of stunting 20 to <30% is high and greater than or equal to 30% is very high [43]. Additionally, the pooled prevalence of stunting aligns with findings from studies carried out in 65 Low- and Middle-Income Countries(29.0%) [44], 62 LMICs worldwide(29.1%) [45], Tajikistan (27%) [46], and Tanzania (28%) [47].

However, the observed prevalence is lower than that reported in previous studies conducted in Ethiopia (33.58%) [48], Low- and Middle-Income Countries(38.8%) [49], East Africa(33.3%, 39%) [50,51], Sub-Saharan Africa(33.2%) [51], Pakistan(44%) [52], Ethiopia(37%) [53], Kenya (39%) [54], Sub-Saharan Africa(35%) [4], Tanzania(35%) [55], Vietnam (44%) [56], Chitwan (37.3%) [57], and Belahara VDC (37%) [58],. Additionally, this finding was higher than the prevalence of stunting reported in studies conducted in China (4.4%) [59], Bangladesh (25%) [60], Bhutan(21%) [61], and UNICEF-WHO-WB Joint Child Malnutrition Estimates(22.3%) [62].

A potential explanation for this finding could be the differences in nutritional practices, sociocultural and environmental factors across the countries. Variations in the scope of the studies, the year of research, population characteristics, and methodologies employed might have contributed to the observed differences. For instance, earlier studies were limited in scope, focusing on individual countries and utilizing diverse analytical approaches [4,24,63–66] and some studies also included a restricted number of countries and population characteristics such as ages [4]. Moreover, another main reason could be attributed to the prolonged malnutrition among children under 5 years of age in Sub-Saharan Africa (SSA), stemming from persistent poverty in the region [67]. Furthermore, the limited economic resources of many African nations can hinder access to sufficient and nutritious food, contributing to the prevalence of stunting [68].

Although the pooled prevalence of stunting among children under 5 years of age in 35 SSA countries was estimated at 29.89% (95% CI: 26.63, 33.14%), the exceptional high heterogeneity (I² = 99.6%, p < 0.000) underscores substantial contextual variation across countries. This variation is not statistical only but also reflects meaningful differences in underlying determinants, health system capacity, and sociopolitical environments. Subgroup analysis revealed that East African countries had highest stunting burden (33.73%), while West African showed comparatively lower prevalence (26.83%), suggesting region drivers such as climate vulnerability, food system resilience, or maternal education levels.

However, meta-regression indicated that broad variables like survey year, sample size, country, and sub-region did not significantly explain this heterogeneity (all p-values > 0.05), pointing to the influence of more unmeasured factors such as governance quality, conflict exposure, or local nutrition policies. These findings highlight the limitations of the relying solely on pooled estimate for policy guidance and emphasizes the need for country level diagnostics to identify context specific risk factors. Future research should explore these modifiers systematically to inform tailored interventions rather than one size fits all strategies.

Our findings revealed that stunting among children under 5 years of age in sub-Saharan African countries is shaped by a mix of individual, household-level, and contextual factors. One significant predictor was the sex of the child, with male children more likely to experience stunting than females. This finding aligns with a study conducted in Sub-Saharan African (SSA) countries, as well as studies conducted in Zambia and Ethiopia [4,65,69,70]. The consistency of this association across diverse settings suggests a robust pattern that may reflect biological vulnerability, differential care practices, or gendered exposure to environmental risks. Evidence from longitudinal studies in sub-Saharan Africa indicates that male children are more prone to growth faltering during the first year of life [71]. However, findings from certain interventions program such as nutritional supplementation and maternal education initiatives suggest that these sex-based disparities in stunting may diminish over time [72].

This indicates that while male children may be biologically predisposed to stunting, contextual factors such as household factors, caregiving practices, and health system responsiveness can modify these risks. What remain uncertain is whether the sex differential is primarily driven by biological susceptibility or by modifiable social determinants. Future research, particularly longitudinal interventions studies, is needed to clarify and separate the overlapping biological and socio-behavioral pathways, in order to determine whether targeted strategies for boys could achieve greater reductions in stunting prevalence.

The prevalence of stunting was significantly higher among older children in the age groups of 12–23 months, 24–35 months, 36–47 months, and 48–59 months compared to children under 12 months of age. This finding indicates that the risk of stunting rises as a child grows older. Similar age-related patterns have been reported in studies conducted in Ethiopia [48,53,65,73], Somalia [74], Rwanda [75], and Zimbabwe [76], suggesting that growth faltering often becomes more pronounced after infancy.

A prospective birth cohort studies have demonstrated that growth deficit often accelerates between 6 and 24 months, reflecting both biological vulnerability and the transition to inadequate diets and increased exposure to infections [77,78]. Interventions trial have further shown that timely interventions during this critical window period can reduce stunting prevalence [79]. The novel contribution of this study is the documentation of this established association within our specific, previously less investigated population, thereby strengthening the geographical evidence base. What remain uncertain, however is, the extent to which age-related differences are modifiable through targeted interventions and whether the persistency of stunting in to later childhood reflects irreversible early childhood damage or ongoing exposure to adverse conditions.

Children born in the fifth or higher birth order had an 8% higher prevalence of stunting compared to those born as first-order children. This finding aligns with earlier studies that have examined child nutritional status, considering birth order as a significant confounding factor [80–83]. Cohort studies have shown that later born children often experience reduced access to household resources, shorter breastfeeding duration, and greater exposure to infections, which collectively contribute to growth faltering [84,85]. Interventions study also demonstrated that empowering mothers to space births and manage household resources can mitigate the nutritional advantages faced by higher order children [86,87]. However, the extent to which birth order effects are biologically driven versus socially mediated, and whether targeted interventions such as birth spacing programs or nutrition support for larger family can fully offset these risk remains uncertain.

The perceived size of a baby at birth showed a significant association with stunting status, with children born small and average size more likely to be stunted compared to those born large. This finding is consistent with studies conducted in Africa and other developing countries, such as Indonesia and Ethiopia [88–90] as well as Nepal [91], Bangladesh [92] and Ethiopia [93]. Cohort studies conducted in Ethiopia and Malawi have shown that children born small remain at elevated risk of stunting well into the first two years of life, even after accounting for postnatal feeding practices [94]. Another interventional study on maternal nutrition supplementation programs in Ghana and Tanzania, further confirmed that improving maternal diet and antenatal care can reduce the incidence of the low birth weight and subsequently lower stunting prevalence [95,96]. However, uncertainties remain regarding the extent to which postnatal interventions (e.g., complementary feeding programs) can fully offset the disadvantage of being born small, suggesting that prevention strategies must prioritize maternal health and antenatal care.

The study revealed that compared to children whose mothers had a higher level of formal education, the prevalence of stunting was 1.94 times higher among children whose mothers had no formal education, 1.95 times higher among those whose mothers had only primary education, and 1.68 times higher among those whose mothers had secondary education. This finding is supported by a systematic review of studies published worldwide between 2004 and 2014, as well as by research conducted in Ethiopia, Zambia and Nepal [4,97–100]. Previous intervention studies have established that maternal education is a protective factor. For example, community based nutritional programs in Uganda and Tanzania show that incorporating maternal literacy and health educations components resulted in significantly lower stunting prevalence in intervention group versus control group [101,102]. However, whether maternal nutritional counselling can only compensate for the disadvantage of low maternal education in resource-constrained settings remains uncertain. Future research should explore whether combining educational empowerment with direct nutrition interventions yields synergistic effects in reducing stunting.

Children born to mothers aged 15–24 were more likely to experience higher levels of stunting compared to those born to mothers aged 25–49. This finding is consistent with the study conducted in Ethiopia [103]. This may be due to the fact that babies born to younger mothers are more prone to preterm birth and low birth weight, which can increase the risk of neonatal infections and malnutrition, including stunting [104]. Another study also showed that younger maternal age is often associated with limited caregiving experience, reduced autonomy in household decision-making, and lower access to health and nutrition services contributing higher stunting prevalence [105]. However, further research needs maternal age itself to act as an independent risk factor versus being mediated by socioeconomic status, maternal education or health service utilization.

Mothers’ antenatal care (ANC) visits were strongly associated with child stunting. Children whose mothers did not attend any antenatal follow-up and 1–3 ANC visits more likely to be stunted compared to those whose mothers had four or more ANC visits. This finding is supported by a study conducted in Ethiopia [106–108], Zambia [109], Latin America [110,111], and Bangladesh [112]. This may be attributed to the fact that antenatal care (ANC) programs are structured to identify high-risk mothers and deliver nutritional and educational support, such as advice on food hygiene, diet, and lifestyle. These initiatives target key factors that play a significant role in enhancing child nutrition. ANC programs are advocated as an effective approach to lowering the prevalence of low birth weight, and evidence highlighting their effectiveness in reducing adverse pregnancy outcomes in developing countries is steadily increasing [113,114]. However, barriers such as inequitable health system coverage, geographical isolation and weak governance in service delivery restrict women access to timely and quality antenatal care services [115,116].

In this study, the place of delivery showed a significant association with child stunting. Specifically, children born at home were more likely to be stunted compared to those born at health facilities. This finding aligns with a study conducted in Ethiopia [4,117]. A possible explanation for this association is that mothers who deliver at home are less likely to receive proper postnatal care, including vaccinations, which are critical for the healthy growth and development of children. Vaccinations help prevent several vaccine-preventable diseases and ensure children receive essential nutrients, contributing to better overall health outcomes [118].

The study revealed that postnatal care visits were associated with childhood stunting. Mothers who did not attend postnatal care services were 1.03 times more likely to have a stunted child compared to those who did. This finding is consistent with a study conducted in Ethiopia [119]. Study confirmed that strengthening maternal contact with health systems during the postnatal period contributes to improved child growth outcomes [120]. However, limited attendance at postnatal care is not solely a matter of maternal choice but reflects broader structural determinants. Barriers such as poverty, inequitable health system coverage, weak governance in service delivery and in some context conflict or geographical isolation restrict women access to timely and quality postnatal services [115,116].

Addressing stunting therefore requires structural interventions such as equitable health infrastructure, improving governance and accountability in maternal and child health programs, and strengthening social protection alongside efforts to promote household-level health-seeking behaviors. Additionally, the extent to which postnatal care independently reduces stunting versus acting synergistically with other maternal and child health interventions such as antenatal care, breastfeeding promotion, and community nutrition programs remains uncertain.

Another factor associated with stunting was marital status. In the study, the prevalence of child stunting was higher among children of single mothers than among children with married mothers. This finding consistent with previous study [121]. The possible explanation may be attributed to socioeconomic challenges, such as limited financial resources, reduced access to healthcare, and lower social support. Single mothers often face greater difficulties in providing adequate nutrition and care for their children due to these constraints. Additionally, the stress and time demands associated with single parenthood may further hinder their ability to ensure optimal child-rearing conditions, contributing to poorer growth outcomes. These factors collectively increase the risk of stunting in children of single mothers [122].

This study found that maternal body mass index was associated with childhood stunting. Compared to children whose mothers had a normal body mass index (BMI), the prevalence of stunting was higher among children of underweight mothers and lower among those of overweight mothers, and obese mothers. These findings were consistent with previous study conducted in low-income and middle-income countries [123], Ethiopia [124,125], Bangladesh [126] and Nigeria [6]. While poor maternal nutrition likely contributes to inadequate nutrient transfer during pregnancy and lactation, resulting in impaired child growth, the relationship between maternal BMI and stunting extends beyond individual level behaviors. Lower stunting rates among children of overweight or obese mothers may reflect not only higher caloric intake but also broader socioeconomic advantages, such as improved household food security, access to diverse diets, and greater resilience to shocks [127].

These maternal outcomes are shaped by structural determinants, including poverty, food system functioning, governance and conflict that influence both maternal nutrition and child health. For instance, household insecure regions or conflict affected setting may face systematic barriers to adequate nutrition regardless of maternal practices [127,128]. Thus, while balanced maternal nutrition remains critical, these findings underscore the needs to situate maternal and household factors within wider structural contexts. Addressing stunting requires not only promoting individual behavior change but also tackling systematic inequities through poverty reduction, strengthening food systems, improving governance and ensuring stability.

This study revealed that child stunting was high among households with large family sizes. This finding is consistent with a previous study conducted in sub-Saharan Africa [129], and Ethiopia [130,131]. This may be due to the fact that higher prevalence of child stunting in households with large family sizes may be due to resource dilution, where limited financial and food resources are spread thinly among many family members, reducing the quantity and quality of nutrition available for each child. Larger families may also face challenges in providing adequate healthcare and sanitation, further exacerbating the risk of stunting. Additionally, caregivers in large families may have less time and capacity to ensure proper feeding practices and childcare, contributing to poorer growth outcomes [132].

The results of this study also revealed that mothers’ exposure to mass media was significantly associated with high prevalence of child stunting. Children whose mothers had not media exposure were more likely to be stunted compared to those whose mothers had exposure to media. This finding was consistent with studies conducted in Pakistan, Bangladesh and Ethiopia [4,133,134]. Media plays a vital role in promoting sociocultural and economic development, which can contribute to improved nutritional outcomes [135]. However, access to mass media is shaped by systemic issues such as poverty, inequitable information and communication infrastructures, governance of public health messaging, and in some contexts, conflict that limits dissemination of reliable information [136]. These structural barriers constrain households’ ability to access knowledge about nutrition, sanitation, and child health, thereby reinforcing cycles of stunting [137]. Therefore, addressing these challenges require system level interventions.

Our study revealed a significant positive association between the household wealth index and child stunting. Compared to children from the richest household wealth index, child from the poor and middle households’ wealth index had higher odds of being stunted. While these findings align with a systematic review and meta-analysis conducted in Africa between 2000 and 2013 and study conducted in Sub-Saharan Africa [4,138], as well as studies from low-income countries such as Nepal, Zambia, and Nigeria [99,100,139], the implications extend beyond household-level economic status [140]. Household wealth reflects broader structural determinants including entrenched poverty, inequitable food systems, weak governance, and in some settings, the destabilizing effects of conflict that constrain access to adequate nutrition, health services, and safe environments for children [141,142]. Thus, stunting should be understood not only as a consequence of limited household resources but also as a manifestation of systemic inequities.

Furthermore, contextual-level variables specifically, place of residence were significantly associated with child stunting. Children from rural areas had higher prevalence of being stunted compared to their urban counterparts. A finding supported by studies conducted in Pakistan and Ethiopia [143,144]. This may be attributed to the better nutritional status of urban children, which stems from improved maternal prenatal and postnatal care, higher-quality complementary feeding practices, and better immunization coverage. Additionally, urban children benefit from advantages such as family employment opportunities, stronger social and family networks, and greater access to healthcare and social services, all of which contribute to their improved nutritional status compared to rural children [145].

### Strength and limitation of the study

This study utilized a recent, nationally representative dataset encompassing 191,953 children under five from 35 sub-Saharan African countries surveyed between 2011 and 2024. The large sample size substantially enhanced the statistical power to detect true effect sizes and improved the generatability of the findings across diverse settings. Advanced statistical methods, including multilevel mixed-effects Poisson regression with robust variance, were employed to strengthen the analysis and account for the hierarchical nature of the DHS data. By pooling data across countries, the study estimated the prevalence of stunting and its determinants, revealing significant and concerning heterogeneity between countries that persisted even after subgroup and sensitivity analyses. Importantly, the inclusion of key maternal and child health variables such as antenatal care (ANC) and postnatal care (PNC) visits, and maternal body mass index (BMI) addressed gaps overlooked in previous studies [4], thereby providing a more comprehensive understanding of stunting determinants.

Nonetheless, several limitations should be acknowledged. Health system variables, including health insurance coverage, the availability and type of health facilities, and broader country-level contextual factors such as political stability, were not assessed. Furthermore, the cross-sectional design of the DHS surveys precludes establishing causal relationships between independent and dependent variables. While significant associations were identified, determinants such as maternal nutritional status and household media exposure are likely influenced by broader socioeconomic conditions that also directly affect child stunting. Maternal BMI, for example, may shape child nutrition but is itself conditioned by household food insecurity and poverty, while media exposure may influence health behaviors yet remains dependent on socioeconomic access. Without longitudinal data, the temporal of these relationships remains uncertain. Therefore, the findings should be interpreted as highlighting important populations-level correlates rather than confirming causal pathways. Future prospective cohort studies are warranted to disentangle these relationships and clarify the causal mechanisms underlying child stunting in SSA.

## Conclusion

In conclusion, this study analyzed pooled data from 35 sub-Saharan African (SSA) countries and found an overall stunting prevalence of 29.72% among children under 5 years of age, with significant variations across countries, ranging from 13.74% in Gabon to 55.74% in Burundi. Fourteen countries had stunting rates exceeding 30%, indicating a very high public health concern as per WHO thresholds.

Our study identified individual, household, and contextual factors significantly linked to stunting in children under 5 years of age. At the individual level, key predictors included being male, increased age of child, higher birth order (fifth or more), small or medium birth size, insufficient antenatal care (ANC) visits (none or 1–3), lack of postnatal visits, lower maternal education (no formal, primary, or secondary education), home delivery, single, unmarried status, and maternal underweight, all of which were associated with increased prevalence of stunting. In contrast, maternal overweight or obesity were linked to reduced prevalence of stunting. At the household level, factors such as lack of maternal media exposure (television, radio, or newspapers), large family size, and lower household wealth (poor, middle, rich) were associated with higher prevalence of stunting. Additionally, at the contextual level, being rural residence was a significant predictor of stunting.

To address persistently high prevalence of stunting in sub-Saharan Africa, interventions should be clearly prioritized by feasibility and timeline but framed within both household level and structural determinants. Immediate actions remain critical such as improving maternal and child healthcare access through increased antenatal and postnatal care visit, promoting facility-based deliveries, and enhancing maternal education to strengthen feeding practices and nutrition awareness. Expanding media outreach can also provide rapid gains in health and nutrition knowledge.

However, this effort must be complemented by long-term structural and systems level investments that tackle the root cause of the stunting. Poverty alleviation, resilient food systems, and equitable access to nutritious diets are central to reducing vulnerability. Governance and political stability play a decisive role in ensuring effective implementation of nutrition policies while conflict and displacement exacerbate food insecurity and health service disruptions. Addressing gender inequality in child nutrition, improved access to clean water and sanitation, expanding clean cooking fuels, and bridging between urban-rural disparities through strengthened healthcare infrastructure and social protection programs are equally vital.

A multisectoral approach like integrating health, education, agriculture, food systems and WASH sectors is essential to ensure both short term impact and sustainable improvements in child growth and developments. Future research should incorporate health systems variables like facility availability and quality alongside country level factors like governance, policy coherence and conflict dynamic. Longitudinal design is needed to establish casual pathways and guide more effective policy strategies that balance individual behavior change with structural reforms.

## Abbreviations

AIC: Akaike’s Information Criteria
APR: Adjusted Prevalence Ratio
BIC: Bayesian Information Criteria
CI: Confidence Interval
CPR: Crude Prevalence Ratio
DHS: Demographic and Health Survey
ICC: Intra Class Correlation Coefficient
IRB: Institutional Review Board
LL: Log-Likelihood
MOR: Median Odds Ratio
PCV: Proportional Change in Variance
SSA: Sub-Saharan Africa

## References

[1] de Onis M, Branca F. Childhood stunting: a global perspective. Matern Child Nutr. 2016;12 Suppl 1(Suppl 1):12–26. doi: 10.1111/mcn.12231 27187907 PMC5084763

[2] Callanan A. Food and nutrition handbook. 2015.

[3] Bharali N, Singh KhN, Mondal N. Composite Index of Anthropometric Failure (CIAF) among Sonowal Kachari tribal preschool children of flood effected region of Assam, India. AR. 2019;82(2):163–76. doi: 10.2478/anre-2019-0012

[4] Takele BA, Gezie LD, Alamneh TS. Pooled prevalence of stunting and associated factors among children aged 6-59 months in Sub-Saharan Africa countries: A Bayesian multilevel approach. PLoS One. 2022;17(10):e0275889. doi: 10.1371/journal.pone.0275889 36228030 PMC9560624

[5] Food R, Affordable HDM. Food security and nutrition in the World. 2022.

[6] Lawal SA, Okunlola DA, Adegboye OA, Adedeji IA. Mother’s education and nutritional status as correlates of child stunting, wasting, underweight, and overweight in Nigeria: Evidence from 2018 Demographic and Health Survey. Nutr Health. 2024;30(4):821–30. doi: 10.1177/02601060221146320 36591921

[7] UNICEF. WHO/World Bank Joint Child Malnutrition Estimates, 2021 Edition. 2022. https://data.unicef.org/topic/nutrition/malnutrition

[8] Mbogori T, Kimmel K, Zhang M, Kandiah J, Wang Y. Nutrition transition and double burden of malnutrition in Africa: A case study of four selected countries with different social economic development. AIMS Public Health. 2020;7(3):425–39. doi: 10.3934/publichealth.2020035 32968668 PMC7505783

[9] Deshpande A, Ramachandran R. Early childhood stunting and later life outcomes: A longitudinal analysis. Econ Hum Biol. 2022;44:101099. doi: 10.1016/j.ehb.2021.101099 34933274

[10] De Sanctis V, Soliman A, Alaaraj N, Ahmed S, Alyafei F, Hamed N. Early and Long-term Consequences of Nutritional Stunting: From Childhood to Adulthood. Acta Biomed. 2021;92(1):e2021168. doi: 10.23750/abm.v92i1.11346 33682846 PMC7975963

[11] Ahmed KY, Agho KE, Page A, Arora A, Ogbo FA, On Behalf Of The Global Maternal And Child Health Research Collaboration GloMACH. Mapping Geographical Differences and Examining the Determinants of Childhood Stunting in Ethiopia: A Bayesian Geostatistical Analysis. Nutrients. 2021;13(6):2104. doi: 10.3390/nu13062104 34205375 PMC8234472

[12] Quamme SH, Iversen PO. Prevalence of child stunting in Sub-Saharan Africa and its risk factors. Clinical Nutrition Open Science. 2022;42:49–61. doi: 10.1016/j.nutos.2022.01.009

[13] Dapi Nzefa L, Monebenimp F, Äng C. Undernutrition among children under five in the Bandja village of Cameroon, Africa. South African Journal of Clinical Nutrition. 2018;32(2):46–50. doi: 10.1080/16070658.2018.1448503

[14] Amare M, Benson T, Fadare O, Oyeyemi M. Study of the determinants of chronic malnutrition in northern nigeria: quantitative evidence from the nigeria demographic and health surveys. Food Nutr Bull. 2018;39(2):296–314. doi: 10.1177/0379572118392002 29642714

[15] Adeba E. Prevalence of Chronic Malnutrition (Stunting) and Determinant Factors among Children Aged 0-23 Months in Western Ethiopia: A Cross-Sectional Study. J Nutr Disorders Ther. 2014;04(02). doi: 10.4172/2161-0509.1000148

[16] Awoke A, Ayana M, Gualu T. Determinants of severe acute malnutrition among under five children in rural Enebsie Sarmidr District, East Gojjam Zone, North West Ethiopia, 2016. BMC Nutrition. 2018;4(1):4. 32153868 10.1186/s40795-018-0211-5 PMC7050716

[17] Ahmed MM, Hokororo A, Kidenya BR, Kabyemera R, Kamugisha E. Prevalence of undernutrition and risk factors of severe undernutrition among children admitted to Bugando Medical Centre in Mwanza, Tanzania. BMC Nutr. 2016;2(1). doi: 10.1186/s40795-016-0090-6

[18] Rose ES, Blevins M, González-Calvo L, Ndatimana E, Green AF, Lopez M, et al. Determinants of undernutrition among children aged 6 to 59 months in rural Zambézia Province, Mozambique: Results of two population-based serial cross-sectional surveys. BMC Nutr. 2015;1:41. doi: 10.1186/s40795-015-0039-1 27182448 PMC4864006

[19] Mukabutera A, Thomson DR, Hedt-Gauthier BL, Basinga P, Nyirazinyoye L, Murray M. Risk factors associated with underweight status in children under five: an analysis of the 2010 Rwanda Demographic Health Survey (RDHS). BMC Nutr. 2016;2(1). doi: 10.1186/s40795-016-0078-2

[20] Tibilla M. The nutritional impact of the world food programme supported supplementary feeding programme on children less than five years in rural Tamale, Ghana. 2007.

[21] M yalew B. Prevalence and Factors Associated with Stunting, Underweight and Wasting: A Community Based Cross Sectional Study among Children Age 6-59 Months at Lalibela Town, Northern Ethiopia. J Nutr Disorders Ther. 2014;04(02). doi: 10.4172/2161-0509.1000147

[22] Amare ZY, Ahmed ME, Mehari AB. Determinants of nutritional status among children under age 5 in Ethiopia: further analysis of the 2016 Ethiopia demographic and health survey. Global Health. 2019;15(1):62. doi: 10.1186/s12992-019-0505-7 31694661 PMC6836473

[23] Poda GG, Hsu C-Y, Chao JC-J. Factors associated with malnutrition among children <5 years old in Burkina Faso: evidence from the Demographic and Health Surveys IV 2010. Int J Qual Health Care. 2017;29(7):901–8. doi: 10.1093/intqhc/mzx129 29045661

[24] Haile D, Azage M, Mola T, Rainey R. Exploring spatial variations and factors associated with childhood stunting in Ethiopia: spatial and multilevel analysis. BMC Pediatr. 2016;16:49. doi: 10.1186/s12887-016-0587-9 27084512 PMC4833938

[25] Talukder A. Factors Associated with Malnutrition among Under-Five Children: Illustration using Bangladesh Demographic and Health Survey, 2014 Data. Children (Basel). 2017;4(10):88. doi: 10.3390/children4100088 29048355 PMC5664018

[26] Skoufias E, Vinha K, Sato R. Reducing Stunting through Multisectoral Efforts in Sub-Saharan Africa. Journal of African Economies. 2021;30(4):324–48. doi: 10.1093/jae/ejaa016

[27] UNICEF. Nutrition Strategy 2020-2030: Accelerating Stunting Reduction in Sub Saharan Africa. New York: UNICEF. 2019.

[28] Desa U. Transforming our world: the 2030 agenda for sustainable development. 2016.

[29] Stevens GA, Finucane MM, Paciorek CJ, Flaxman SR, White RA, Donner AJ, et al. Trends in mild, moderate, and severe stunting and underweight, and progress towards MDG 1 in 141 developing countries: a systematic analysis of population representative data. Lancet. 2012;380(9844):824–34. doi: 10.1016/S0140-6736(12)60647-3 22770478 PMC3443900

[30] Bursac Z, Gauss CH, Williams DK, Hosmer DW. Purposeful selection of variables in logistic regression. Source Code Biol Med. 2008;3:17. doi: 10.1186/1751-0473-3-17 19087314 PMC2633005

[31] Hosmer Jr DW, Lemeshow S, Sturdivant RX. Applied logistic regression. John Wiley & Sons. 2013.

[32] Croft TN, Marshall AM, Allen CK, Arnold F, Assaf S, Balian S. Guide to DHS statistics. Rockville: ICF. 2018.

[33] USAID. Demographic and health survey (DHS) methodology. https://dhsprogram.com/methodology/Survey-Types/DHS-Methodology.cfm. 2023.

[34] Rahman MS, Howlader T, Masud MS, Rahman ML. Association of Low-Birth Weight with Malnutrition in Children under Five Years in Bangladesh: Do Mother’s Education, Socio-Economic Status, and Birth Interval Matter?. PLoS One. 2016;11(6):e0157814. doi: 10.1371/journal.pone.0157814 27355682 PMC4927179

[35] Ntenda PAM. Association of low birth weight with undernutrition in preschool-aged children in Malawi. Nutr J. 2019;18(1):51. doi: 10.1186/s12937-019-0477-8 31477113 PMC6719380

[36] Aboagye RG, Seidu A-A, Ahinkorah BO, Arthur-Holmes F, Cadri A, Dadzie LK, et al. Dietary Diversity and Undernutrition in Children Aged 6-23 Months in Sub-Saharan Africa. Nutrients. 2021;13(10):3431. doi: 10.3390/nu13103431 34684435 PMC8537414

[37] WHO. Obesity and overweight. https://www.who.int/news-room/fact-sheets/detail/obesity-and-overweight. 2021.

[38] Weir CB, Jan A. BMI classification percentile and cut off points. 2019.

[39] Cooper H, Hedges LV, Valentine JC. The handbook of research synthesis and meta-analysis. Russell Sage Foundation. 2019.

[40] Greene W. Functional forms for the negative binomial model for count data. Economics Letters. 2008;99(3):585–90. doi: 10.1016/j.econlet.2007.10.015

[41] Skoufias E, Vinha K, Sato R. All hands on deck: reducing stunting through multisectoral efforts in Sub-Saharan Africa. World Bank Publications. 2019.

[42] UNICEF U. Nutrition Strategy 2020-2030: Accelerating Stunting Reduction in Sub-Saharan Africa. New York: UNICEF. 2019.

[43] de Onis M, Arimond M, Webb P, Croft T, Saha K, de Onis M, et al. Prevalence thresholds for wasting, overweight and stunting in children under 5 years. Public Health Nutrition. 2018;:1–5. doi: 10.1017/S1368980018002434 30296964 PMC6390397

[44] Akombi BJ, Chitekwe S, Sahle BW, Renzaho AMN. Estimating the Double Burden of Malnutrition among 595,975 Children in 65 Low- and Middle-Income Countries: A Meta-Analysis of Demographic and Health Surveys. Int J Environ Res Public Health. 2019;16(16):2886. doi: 10.3390/ijerph16162886 31412530 PMC6720202

[45] Ssentongo P, Ssentongo AE, Ba DM, Ericson JE, Na M, Gao X. Global, regional and national epidemiology and prevalence of child stunting, wasting and underweight in low-and middle-income countries, 2006–2018. Scientific Reports. 2021;11(1):5204. 33664313 10.1038/s41598-021-84302-w PMC7933191

[46] The state of the world’s children 2016 report. New York: UNICEF. 2016.

[47] Kejo D, Mosha TCE, Petrucka P, Martin H, Kimanya ME. Prevalence and predictors of undernutrition among underfive children in Arusha District, Tanzania. Food Sci Nutr. 2018;6(8):2264–72. doi: 10.1002/fsn3.798 30510726 PMC6261180

[48] Seifu BL, Tesema GA, Fentie BM, Yehuala TZ, Moloro AH, Mare KU. Geographical variation in hotspots of stunting among under-five children in Ethiopia: A geographically weighted regression and multilevel robust Poisson regression analysis. PLoS One. 2024;19(5):e0303071. doi: 10.1371/journal.pone.0303071 38743707 PMC11093352

[49] Li Z, Kim R, Vollmer S, Subramanian SV. Factors Associated With Child Stunting, Wasting, and Underweight in 35 Low- and Middle-Income Countries. JAMA Netw Open. 2020;3(4):e203386. doi: 10.1001/jamanetworkopen.2020.3386 32320037 PMC7177203

[50] Tesema GA, Yeshaw Y, Worku MG, Tessema ZT, Teshale AB. Pooled prevalence and associated factors of chronic undernutrition among under-five children in East Africa: A multilevel analysis. PLoS One. 2021;16(3):e0248637. doi: 10.1371/journal.pone.0248637 33765094 PMC7993805

[51] Akombi BJ, Agho KE, Merom D, Renzaho AM, Hall JJ. Child malnutrition in sub-Saharan Africa: A meta-analysis of demographic and health surveys (2006-2016). PLoS One. 2017;12(5):e0177338. doi: 10.1371/journal.pone.0177338 28494007 PMC5426674

[52] Khan S, Zaheer S, Safdar NF. Determinants of stunting, underweight and wasting among children < 5 years of age: evidence from 2012-2013 Pakistan demographic and health survey. BMC Public Health. 2019;19(1):358. doi: 10.1186/s12889-019-6688-2 30935382 PMC6444880

[53] Raru TB, Ayana GM, Merga BT, Negash B, Deressa A, Birhanu A, et al. Magnitude of under-nutrition among under five children in Ethiopia based on 2019 Mini-Ethiopia Demographic and Health Survey: Generalized Linear Mixed Model (GLMM). BMC Nutr. 2022;8(1):113. doi: 10.1186/s40795-022-00598-5 36253811 PMC9575191

[54] Badake Q, Maina I, Mboganie M, Muchemi G, Kihoro E, Chelimo E. Nutritional status of children under five years and associated factors in Mbeere South District, Kenya. African Crop Science Journal. 2014;22:799–806.

[55] Mamiro PS, Kolsteren P, Roberfroid D, Tatala S, Opsomer AS, Van Camp JH. Feeding practices and factors contributing to wasting, stunting, and iron-deficiency anaemia among 3-23-month old children in Kilosa district, rural Tanzania. J Health Popul Nutr. 2005;23(3):222–30. 16262018

[56] Hien NN, Kam S. Nutritional status and the characteristics related to malnutrition in children under five years of age in Nghean, Vietnam. J Prev Med Public Health. 2008;41(4):232–40. doi: 10.3961/jpmph.2008.41.4.232 18664729

[57] Ruwali D. Nutritional Status of Children Under Five Years of Age and Factors Associated in Padampur VDC, Chitwan. Health Prospect. 2018;10:14–8. doi: 10.3126/hprospect.v10i0.5639

[58] Sapkota V, Gurung C. Community medicine and family health department, Maharajgunj campus, Institute of Medicine, Tribhuwan University. Journal of Nepal Health Research Council. 2009;7(2):120–6.

[59] Li H, Yuan S, Fang H, Huang G, Huang Q, Wang H, et al. Prevalence and associated factors for stunting, underweight and wasting among children under 6 years of age in rural Hunan Province, China: a community-based cross-sectional study. BMC Public Health. 2022;22(1):483. doi: 10.1186/s12889-022-12875-w 35277139 PMC8917668

[60] Sultana P, Rahman MM, Akter J. Correlates of stunting among under-five children in Bangladesh: a multilevel approach. BMC Nutr. 2019;5:41. doi: 10.1186/s40795-019-0304-9 32153954 PMC7050784

[61] Kang Y, Aguayo VM, Campbell RK, Dzed L, Joshi V, Waid JL, et al. Nutritional status and risk factors for stunting in preschool children in Bhutan. Matern Child Nutr. 2018;14 Suppl 4(Suppl 4):e12653. doi: 10.1111/mcn.12653 30412341 PMC6587444

[62] UNICEF-WHO-WB Joint Child Malnutrition Estimates inter-agency group. Child malnutrition estimates for the indicators stunting, wasting, overweight and underweight describe the magnitude and patterns of under- and overnutrition. 2022.

[63] Aboagye RG, Ahinkorah BO, Seidu A-A, Frimpong JB, Archer AG, Adu C, et al. Birth weight and nutritional status of children under five in sub-Saharan Africa. PLoS One. 2022;17(6):e0269279. doi: 10.1371/journal.pone.0269279 35679306 PMC9182265

[64] Hailu BA, Bogale GG, Beyene J. Spatial heterogeneity and factors influencing stunting and severe stunting among under-5 children in Ethiopia: spatial and multilevel analysis. Sci Rep. 2020;10(1):16427. doi: 10.1038/s41598-020-73572-5 33009463 PMC7532151

[65] Muche A, Gezie LD, Baraki AG-E, Amsalu ET. Predictors of stunting among children age 6–59 months in Ethiopia using Bayesian multi-level analysis. Scientific Reports. 2021;11(1):3759. 33580097 10.1038/s41598-021-82755-7 PMC7881183

[66] Tamir TT, Techane MA, Dessie MT, Atalell KA. Applied nutritional investigation spatial variation and determinants of stunting among children aged less than 5 y in Ethiopia: A spatial and multilevel analysis of Ethiopian Demographic and Health Survey 2019. Nutrition. 2022;103–104:111786. doi: 10.1016/j.nut.2022.111786 35970098

[67] Harttgen K, Klasen S, Vollmer S. Economic Growth and Child Undernutrition in sub‐Saharan Africa. Population & Development Rev. 2013;39(3):397–412. doi: 10.1111/j.1728-4457.2013.00609.x

[68] Ahmad D, Afzal M, Imtiaz A. Effect of socioeconomic factors on malnutrition among children in Pakistan. Futur Bus J. 2020;6(1). doi: 10.1186/s43093-020-00032-x

[69] Fantay Gebru K, Mekonnen Haileselassie W, Haftom Temesgen A, Oumer Seid A, Afework Mulugeta B. Determinants of stunting among under-five children in Ethiopia: a multilevel mixed-effects analysis of 2016 Ethiopian demographic and health survey data. BMC Pediatr. 2019;19(1):176. doi: 10.1186/s12887-019-1545-0 31153381 PMC6544992

[70] Wamani H, Astrøm AN, Peterson S, Tumwine JK, Tylleskär T. Boys are more stunted than girls in sub-Saharan Africa: a meta-analysis of 16 demographic and health surveys. BMC Pediatr. 2007;7:17. doi: 10.1186/1471-2431-7-17 17425787 PMC1865375

[71] Mertens A, Benjamin-Chung J, Colford JM Jr, Coyle J, van der Laan MJ, Hubbard AE, et al. Causes and consequences of child growth faltering in low-resource settings. Nature. 2023;621(7979):568–76. doi: 10.1038/s41586-023-06501-x 37704722 PMC10511328

[72] Nyamasege CK, Kimani-Murage EW, Wanjohi M, Kaindi D, Wagatsuma Y. Effect of maternal nutritional education and counselling on children’s stunting prevalence in urban informal settlements in Nairobi, Kenya. Public Health Nutr. 2021;24(12):3740–52. doi: 10.1017/S1368980020001962 32693855 PMC7611536

[73] Teshome B, Kogi-Makau W, Getahun Z, Taye G. Magnitude and determinants of stunting in children underfive years of age in food surplus region of Ethiopia: The case of West Gojam Zone. Ethiopian Journal of Health Development. 2010;23(2). doi: 10.4314/ejhd.v23i2.53223

[74] Nor SM, Mwajuma J, Mohamed MAA. Determinants of malnutrition among children under five years in SOS Hospital, Mogadishu. 2016.

[75] Niyigena DI, Semayira CA, Mutabazi M, Ntirushwamaboko N, Habimana J de D, Iyakaremye D, et al. Feeding Practices and Nutritional Status among Children Aged from Six to 23 Months in Western Province, Rwanda: A cross-sectional study. Rwanda J Med Health Sci. 2023;6(2):228–38. doi: 10.4314/rjmhs.v6i2.14 40568404 PMC12110511

[76] Marume A, Archary M, Mahomed S. Predictors of stunting among children aged 6-59 months, Zimbabwe. Public Health Nutr. 2023;26(4):820–33. doi: 10.1017/S1368980023000046 36621006 PMC10131138

[77] MAL-ED Network Investigators. Relationship between growth and illness, enteropathogens and dietary intakes in the first 2 years of life: findings from the MAL-ED birth cohort study. BMJ Glob Health. 2017;2(4):e000370. doi: 10.1136/bmjgh-2017-000370 29333282 PMC5759708

[78] González-Fernández D, Cousens S, Rizvi A, Chauhadry I, Soofi SB, Bhutta ZA. Infections and nutrient deficiencies during infancy predict impaired growth at 5 years: Findings from the MAL-ED study in Pakistan. Front Nutr. 2023;10:1104654. doi: 10.3389/fnut.2023.1104654 36875830 PMC9982131

[79] Jardí C, Casanova BD, Arija V. Nutrition Education Programs Aimed at African Mothers of Infant Children: A Systematic Review. Int J Environ Res Public Health. 2021;18(14):7709. doi: 10.3390/ijerph18147709 34300158 PMC8305319

[80] Rahman M. Association between order of birth and chronic malnutrition of children: a study of nationally representative Bangladeshi sample. Cad Saude Publica. 2016;32(2):e00011215. doi: 10.1590/0102-311X00011215 26958818

[81] Khan REA, Raza MA. Nutritional status of children in Bangladesh: measuring composite index of anthropometric failure (CIAF) and its determinants. 2014.

[82] Shapiro-Mendoza C, Selwyn BJ, Smith DP, Sanderson M. Parental pregnancy intention and early childhood stunting: findings from Bolivia. Int J Epidemiol. 2005;34(2):387–96. doi: 10.1093/ije/dyh354 15561748

[83] Ukwuani FA, Suchindran CM. Implications of women’s work for child nutritional status in sub-Saharan Africa: a case study of Nigeria. Soc Sci Med. 2003;56(10):2109–21. doi: 10.1016/s0277-9536(02)00205-8 12697201

[84] Saaka M, Aggrey B. Effect of Birth Interval on Foetal and Postnatal Child Growth. Scientifica (Cairo). 2021;2021:6624184. doi: 10.1155/2021/6624184 34471555 PMC8405331

[85] Zhao M, Wu H, Liang Y, Liu F, Bovet P, Xi B. Breastfeeding and Mortality Under 2 Years of Age in Sub-Saharan Africa. Pediatrics. 2020;145(5):e20192209. doi: 10.1542/peds.2019-2209 32321779

[86] Dasgupta DP, Sujon MSH, Afroz M, Mazumder S, Suman SM, Khan MTF. Impact of Birth Spacing and Maternal-Child Risk Factors on Childhood Stunting, Wasting, Underweight, and their Coexistence in South Asia. Wasting, Underweight, and their Coexistence in South Asia.

[87] Damtie Y, Kefale B, Yalew M, Arefaynie M, Adane B. Short birth spacing and its association with maternal educational status, contraceptive use, and duration of breastfeeding in Ethiopia. A systematic review and meta-analysis. PLoS One. 2021;16(2):e0246348. doi: 10.1371/journal.pone.0246348 33534830 PMC7857626

[88] Gebru KF, Temesgen AH, Seid AO, Mulugeta BA. Determinants of stunting among under-five children in Ethiopia: a multilevel mixed-effects analysis of 2016 Ethiopian demographic and health survey data. BMC Pediatrics. 2019;19(1):1–13. 31153381 10.1186/s12887-019-1545-0 PMC6544992

[89] Amsalu Feleke BM. Magnitude of Stunting and Associated Factors Among 6-59 Months Old Children in Hossana Town, Southern Ethiopia. J Clinic Res Bioeth. 2015;6(1):1.

[90] Ramli, Agho KE, Inder KJ, Bowe SJ, Jacobs J, Dibley MJ. Prevalence and risk factors for stunting and severe stunting among under-fives in North Maluku province of Indonesia. BMC Pediatr. 2009;9:64. doi: 10.1186/1471-2431-9-64 19818167 PMC2765943

[91] Tiwari R, Ausman LM, Agho KE. Determinants of stunting and severe stunting among under-fives: evidence from the 2011 Nepal Demographic and Health Survey. BMC Pediatr. 2014;14:239. doi: 10.1186/1471-2431-14-239 25262003 PMC4263111

[92] Rahman A, Chowdhury S. Determinants of chronic malnutrition among preschool children in Bangladesh. J Biosoc Sci. 2007;39(2):161–73. doi: 10.1017/S0021932006001295 16566847

[93] Yimer G. Malnutrition among children in Southern Ethiopia: Levels and risk factors. 2000.

[94] Goddard FG, Hunegnaw BM, Luu J, Haneuse S, Zeleke M, Mohammed Y. Child stunting from birth to age two years: The Birhan Cohort in Ethiopia. medRxiv. 2023. doi: 2023.05.20.23290246

[95] Habtu M, Agena AG, Umugwaneza M, Mochama M, Munyanshongore C. Effectiveness of Integrated Maternal Nutrition Intervention Package on Birth Weight in Rwanda. Front Nutr. 2022;9:874714. doi: 10.3389/fnut.2022.874714 35938121 PMC9353189

[96] Bigool M. Effects of free maternal healthcare on stunting in children under five years of age: Evidence from Ghana. Development Policy Review. 2024;42(4). doi: 10.1111/dpr.12771

[97] P T. Review of maternal effects on early childhood stunting. 2014.

[98] Budhathoki SS, Bhandari A, Gurung R, Gurung A, Kc A. Stunting Among Under 5-Year-Olds in Nepal: Trends and Risk Factors. Matern Child Health J. 2020;24(Suppl 1):39–47. doi: 10.1007/s10995-019-02817-1 31776750 PMC7048700

[99] Mzumara B, Bwembya P, Halwiindi H, Mugode R, Banda J. Factors associated with stunting among children below five years of age in Zambia: evidence from the 2014 Zambia demographic and health survey. BMC Nutr. 2018;4:51. doi: 10.1186/s40795-018-0260-9 32153912 PMC7050779

[100] Akombi BJ, Agho KE, Hall JJ, Wali N, Renzaho AMN, Merom D. Stunting, Wasting and Underweight in Sub-Saharan Africa: A Systematic Review. Int J Environ Res Public Health. 2017;14(8):863. doi: 10.3390/ijerph14080863 28788108 PMC5580567

[101] Makoka D, Masibo PK. Is there a threshold level of maternal education sufficient to reduce child undernutrition? Evidence from Malawi, Tanzania and Zimbabwe. BMC Pediatr. 2015;15:96. doi: 10.1186/s12887-015-0406-8 26297004 PMC4546212

[102] Ickes S, Baguma C, Brahe CA, Myhre JA, Bentley ME, Adair LS, et al. Maternal participation in a nutrition education program in Uganda is associated with improved infant and young child feeding practices and feeding knowledge: a post-program comparison study. BMC Nutr. 2017;3:32. doi: 10.1186/s40795-017-0140-8 31354959 PMC6660008

[103] Asgedom YS, Mare KU, Asmare ZA, Asebe HA, Kase BF, et al. Levels of stunting associated factors among under-five children in Ethiopia: A multi-level ordinal logistic regression analysis. 2024. 10.1371/journal.pone.0296451 PMC10760711 38165921

[104] Finlay JE, Özaltin E, Canning D. The association of maternal age with infant mortality, child anthropometric failure, diarrhoea and anaemia for first births: evidence from 55 low- and middle-income countries. BMJ Open. 2011;1(2):e000226. doi: 10.1136/bmjopen-2011-000226 22021886 PMC3191600

[105] Chilinda ZB, Wahlqvist ML, Lee M-S, Huang Y-C. Higher maternal autonomy is associated with reduced child stunting in Malawi. Sci Rep. 2021;11(1):3882. doi: 10.1038/s41598-021-83346-2 33594098 PMC7886910

[106] Tariku B, Mulugeta A, Tsadik M, Azene G. Prevalence and Risk Factors of Child Malnutrition in Community Based Nutrition Program Implementing and Nonimplementing Districts from South East Amhara, Ethiopia. OALib. 2014;01(03):1–17. doi: 10.4236/oalib.1100425

[107] Shiferaw ABG, Rajalakshmi M, Masresha A, Yohannes MA. Stunting and its determinants among children aged 6–59 months in northern Ethiopia: A cross-sectional study. 2018. 10.1155/2018/1078480 PMC6036796 30046469

[108] Abeway S, Gebremichael B, Murugan R, Assefa M, Adinew YM. Stunting and Its Determinants among Children Aged 6-59 Months in Northern Ethiopia: A Cross-Sectional Study. J Nutr Metab. 2018;2018:1078480. doi: 10.1155/2018/1078480 30046469 PMC6036796

[109] Bwalya BB, Lemba M, Mapoma CC, Mutombo N. Factors Associated with Stunting among Children Aged 6-23 Months in Zambian: Evidence from the 2007 Zambia Demographic and Health Survey. IJANHS. 2015;3(1):116–31. doi: 10.23953/cloud.ijanhs.146

[110] Forero-Ramirez N, Gamboa LF, Bedi A, Sparrow R. Child malnutrition and prenatal care: evidence from three Latin American countries. Rev Panam Salud Publica. 2014;35(3):163–71. 24793862

[111] Ramirez NGL, Bedi AS, Sparrow R. Child malnutrition and antenatal care: evidence from three Latin American countries. 2012. 24793862

[112] Svefors P, Sysoev O, Ekstrom E-C, Persson LA, Arifeen SE, Naved RT, et al. Relative importance of prenatal and postnatal determinants of stunting: data mining approaches to the MINIMat cohort, Bangladesh. BMJ Open. 2019;9(8):e025154. doi: 10.1136/bmjopen-2018-025154 31383692 PMC6687011

[113] Habibov NNFL. Does prenatal healthcare improve child birthweight outcomes in Azerbaijan? Results of the national Demographic and health survey. Econ Hum Biol. 2011;9(1):56–65. doi: 10.1016/j.ehb.2010.08.003 20851064

[114] G-G G. The impact of adequate prenatal care on urban birth outcomes: an analysis in a developing country context. Econ Dev Cult Change. 2013;62(1):95–130. doi: 10.1086/671716

[115] Langlois ÉV, Miszkurka M, Zunzunegui MV, Ghaffar A, Ziegler D, Karp I. Inequities in postnatal care in low- and middle-income countries: a systematic review and meta-analysis. Bull World Health Organ. 2015;93(4):259–270G. doi: 10.2471/BLT.14.140996 26229190 PMC4431556

[116] Abdulraheem A, Ononokpono D, Raimi M. Breaking barriers to safe motherhood: how social, cultural, and geographic inequalities shape skilled birth attendance in Nigeria. Sociol Int J. 2025;9(5):188–200.

[117] Zeleke BM, Rahman A. Prevalence and Determinants of Chronic Malnutrition Among Under-5 Children in Ethiopia. Int J Child Health Nutr. 2013;2(3):230–6. doi: 10.6000/1929-4247.2013.02.03.5

[118] U C, Cww C. Stunting: and what it is and what it means. https://www.concernusa.org/story/what-is-stunting/. 2019. Accessed 2021.

[119] Tadesse SEMT. Prevalence and associated factors of stunting among children aged 6–59 months in Delanta district; North East Ethiopia. 2019.

[120] Elisaria E, Mrema J, Bogale T, Segafredo G, Festo C. Effectiveness of integrated nutrition interventions on childhood stunting: a quasi-experimental evaluation design. BMC Nutr. 2021;7(1):17. doi: 10.1186/s40795-021-00421-7 33980311 PMC8117266

[121] Mwamba MS, Gerrior S, Taylor R. Relationship Between Single Motherhood Status and Stunting Among Children Under 5 in Kgatleng, Botswana. JSBHS. 2022;16(1). doi: 10.5590/jsbhs.2022.16.1.03

[122] Gibbs CM, Wendt A, Peters S, Hogue CJ. The impact of early age at first childbirth on maternal and infant health. Paediatr Perinat Epidemiol. 2012;26 Suppl 1(0 1):259–84. doi: 10.1111/j.1365-3016.2012.01290.x 22742615 PMC4562289

[123] Black RE, Walker SP, Bhutta ZA, Christian P, de Onis M. Maternal and child undernutrition and overweight in low-income and middle-income countries. 2013. 10.1016/S0140-6736(13)60937-X 23746772

[124] Amaha ND, Woldeamanuel BT. Maternal factors associated with moderate and severe stunting in Ethiopian children: analysis of some environmental factors based on 2016 demographic health survey. Nutr J. 2021;20(1):18. doi: 10.1186/s12937-021-00677-6 33639943 PMC7916293

[125] Berhe K, Seid O, Gebremariam Y, Berhe A, Etsay N. Risk factors of stunting (chronic undernutrition) of children aged 6 to 24 months in Mekelle City, Tigray Region, North Ethiopia: An unmatched case-control study. PLoS One. 2019;14(6):e0217736. doi: 10.1371/journal.pone.0217736 31181094 PMC6557492

[126] Mostafa KSM. Socio-economic determinants of severe and moderate stunting among under-five children of rural Bangladesh. Malays J Nutr. 2011;17(1):105–18. 22135870

[127] Kota K, Pongou R, Chomienne M-H. Impact of household food insecurity on the use of maternal health services in the Savanes region, Togo: a qualitative study. BMC Public Health. 2025;25(1):2040. doi: 10.1186/s12889-025-23220-2 40457279 PMC12128485

[128] Okyere J, Budu E, Aboagye RG, Seidu A-A, Ahinkorah BO, Yaya S. Socioeconomic determinants of the double burden of malnutrition among women of reproductive age in sub-Saharan Africa: A cross-sectional study. Health Sci Rep. 2024;7(5):e2071. doi: 10.1002/hsr2.2071 38742095 PMC11089015

[129] Yaya S, Oladimeji O, Odusina EK, Bishwajit G. Household structure, maternal characteristics and children’s stunting in sub-Saharan Africa: evidence from 35 countries. Int Health. 2022;14(4):381–9. doi: 10.1093/inthealth/ihz105 31927593 PMC9248065

[130] Bogale TY, Bala ET, Tadesse M, Asamoah BO. Prevalence and associated factors for stunting among 6–12 years old school age children from rural community of Humbo district, Southern Ethiopia. BMC public health. 2018;18:1–8. 10.1186/s12889-018-5561-z PMC5968559 29793479

[131] Asefa A, Girma D, Kaso AW, Ferede A, Agero G, Beyen TK. Prevalence of stunting and associated factors among under-five children in Robe Woreda, Arsi zone, Ethiopia. International Journal of Africa Nursing Sciences. 2024;21:100782. doi: 10.1016/j.ijans.2024.100782

[132] Desai S, Alva S. Maternal education and child health: is there a strong causal relationship? Demography. 1998;35(1):71–81. doi: 10.2307/3004028 9512911

[133] Sarma HKJ, Asaduzzaman M, Uddin F, Tarannum S, Hasan MM, et al. Factors influencing the prevalence of stunting among children aged below five years in Bangladesh. Food and nutrition bulletin. 2017;38(3):291–301. 28758423 10.1177/0379572117710103

[134] Ali SAA. Factors associated with stunting among children under five years of age in Pakistan: Evidence from PDHS 2012–13. J Community Public Health Nurs. 2018;4:1–4.

[135] Mbuya NVCR, Morimoto T, Thitsy S. Media and messages for nutrition and health: assessing media appropriateness for nutrition and health-related social and behavior change communication in four high stunting-burden provinces of Lao PDR. World Bank. 2020.

[136] Woldesenbet B, Tolcha A, Tsegaye B. Water, hygiene and sanitation practices are associated with stunting among children of age 24-59 months in Lemo district, South Ethiopia, in 2021: community based cross sectional study. BMC Nutr. 2023;9(1):17. doi: 10.1186/s40795-023-00677-1 36691099 PMC9869534

[137] Seiler J, Libby TE, Jackson E, Lingappa JR, Evans WD. Social Media-Based Interventions for Health Behavior Change in Low- and Middle-Income Countries: Systematic Review. J Med Internet Res. 2022;24(4):e31889. doi: 10.2196/31889 35436220 PMC9052020

[138] W ZT. Magnitude and determinants of stunting among children in Africa: a systematic review. Current Research in Nutrition and Food Science Journal. 2014;2(2):88–93.

[139] Akombi BJ, Agho KE, Hall JJ, Wali N, Renzaho AMN, Merom D. Stunting, Wasting and Underweight in Sub-Saharan Africa: A Systematic Review. Int J Environ Res Public Health. 2017;14(8):863. doi: 10.3390/ijerph14080863 28788108 PMC5580567

[140] Tsega Y, Endawkie A, Kebede SD, Abeje ET, Enyew EB, Daba C, et al. Trends of wealth-related inequality in stunting and its contributing factors among under-five children in Ethiopia: Decomposing the concentration index using Ethiopian Demographic Health Surveys 2011-2019. PLoS One. 2025;20(2):e0314646. doi: 10.1371/journal.pone.0314646 39937754 PMC11819561

[141] Kountchou A, Sonne S, Gadom G. The local impact of armed conflict on children’s nutrition and health outcomes: evidence from Chad. In: University of Oxford, 2019.

[142] Baye K, Laillou A, Chitweke S. Socio-Economic Inequalities in Child Stunting Reduction in Sub-Saharan Africa. Nutrients. 2020;12(1):253. doi: 10.3390/nu12010253 31963768 PMC7019538

[143] Tariq J, Sajjad A, Zakar R, Zakar MZ, Fischer F. Factors Associated with Undernutrition in Children under the Age of Two Years: Secondary Data Analysis Based on the Pakistan Demographic and Health Survey 2012⁻2013. Nutrients. 2018;10(6):676. doi: 10.3390/nu10060676 29861467 PMC6024574

[144] Woldeamanuel BT, Tesfaye TT. Risk Factors Associated with Under-Five Stunting, Wasting, and Underweight Based on Ethiopian Demographic Health Survey Datasets in Tigray Region, Ethiopia. J Nutr Metab. 2019;2019:6967170. doi: 10.1155/2019/6967170 31929903 PMC6939414

[145] Smith LC, Ruel MT, Ndiaye A. Why Is Child Malnutrition Lower in Urban Than in Rural Areas? Evidence from 36 Developing Countries. World Development. 2005;33(8):1285–305. doi: 10.1016/j.worlddev.2005.03.002

