# Supplementary material for: Prevalence of stunting and its determinants among children under five in 35 Sub-Saharan African countries (2011–2024): Insights from recent demographic health survey data using a generalized linear mixed-effects model with robust poisson regression
Source: PLoS One. 2026 Mar 13;21(3):e0344358. doi: 10.1371/journal.pone.0344358 (PMC12987452; doi:10.1371/journal.pone.0344358)
Supplement: Attachment — Submitted filename: PONE-D-25-15249_R1_Editor.pdf [file pone.0344358.s003.pdf]

PLOS One

Prevalence of Stunting and its Determinants Among Children Under Five in 35 Sub-Saharan African Countries (2011–2024): Insights from recent Demographic Health Survey Data Using a Generalized Linear Mixed-Effects Model with Robust Poisson Regression

--Manuscript Draft--

**Manuscript Number:**	PONE-D-25-15249R1
**Article Type:**	Research Article
**Full Title:**	Prevalence of Stunting and its Determinants Among Children Under Five in 35 Sub-Saharan African Countries (2011–2024): Insights from recent Demographic Health Survey Data Using a Generalized Linear Mixed-Effects Model with Robust Poisson Regression
**Short Title:**	Pooled Prevalence of Stunting and its Determinants Among Children Under Five in 35 Sub-Saharan African
**Corresponding Author:**	Abdulkerim Hassen Moloro<br>Samara University<br>ETHIOPIA
**Keywords:**	Stunting, Under-five, Demographic Health Survey, Sub-Saharan Africa
**Abstract:**	Abstract<br>Background: Despite efforts by initiatives like the World Bank's 'All Hands-on Deck' and UNICEF's programs to address stunting through multisectoral approaches, the burden of stunting remains alarmingly high in sub-Saharan Africa. This study utilized recent large-scale survey data from 35 SSA countries (2011–2024) to estimate the pooled prevalence of stunting and its determinants among children under 5 years of age. Key variables such as antenatal care visits, postnatal care, and maternal nutritional indicators, which previous studies did not account for, are incorporated into the analysis.<br>Methods: A secondary analysis was conducted using recent demographic and health survey data (2011–2024) from 35 sub-Saharan African (SSA) countries. A total weighted sample of 191,953 children under 5 years of age was included in the analysis. Descriptive and inferential analyses were performed using STATA 17. Forest plots were utilized to illustrate both pooled and country-specific stunting rates. Determinants of stunting were identified through a multilevel mixed-effects Poisson regression model with robust variance. The adjusted prevalence ratios and their 95% confidence intervals were used to assess the strength and statistical significance of associations.<br>Model comparison was evaluated by model statistical summary such as Akaike's Information Criterion (AIC), Bayesian Information Criterion (BIC), log-likelihood (LL), and deviance (-2*LL).<br>Result: The pooled prevalence of stunting among children under 5 years of age in 35 sub-Saharan African countries was 29.89% (95% CI: 26.63, 33.14%), with the lowest level in Gabon (13.91%) and the highest in Burundi (55.80%). Being male children (aPR =1.24, 95% CI: 1.21–1.26), being aged 12 months or older (aPR: ≥1.81, p<0.01), insufficient antenatal care (ANC) visits (aPR: ≥1.17, p<0.01), lack of postnatal visits(aPR = 1.03, 95% CI: 1.07, 1.05), children perceived as small or average at birth (aPR: ≥1.16, p<0.01), mother without a higher education (aPR: ≥1.94, p<0.01), living in a poor or average wealth household (aPR: ≥1.23, p<0.01) were significant predictors of stunting. Conversely, maternal overweight (aPR = 0.81, 95% CI: 0.77–0.84) and obese mothers (aPR = 0.88, 95% CI: 0.85–0.90) were associated lower prevalence of stunting.<br>Conclusion: Study revealed a stunting prevalence of 29.89% among children under 5 years of age across 35 sub-Saharan African countries, with significant country-level variations and rates exceeding 30% in 15 countries, signaling a major public health concern. Key individual, household, and contextual factors associated with stunting were identified, emphasizing the need for immediate actions expanding antenatal and postnatal care, promoting facility-based deliveries, enhancing maternal education, and media outreach. Long term strategies must tackle poverty, food systems, and equitable

Powered by Editorial Manager® and ProduXion Manager® from Aries Systems Corporation

	nutrition access, supported by governance and stability. A multisectoral approach integrating health, education, agriculture, WASH, and social protection ensures substantiable child growth, complemented by longitudinal research for policy coherence.<br>Keywords: Stunting, Under-five, Demographic Health Survey, Sub-Saharan Africa
**Order of Authors:**	Abdulkerim Hassen Moloro
	Kebede Gemeda Sabo
	Kusse Urmale Mare
	Beriso Furo Wengoro
	Eshetu Elfios Endrias
	Roda Mehadi Ibrahim
	Teshager Dubie
	Oumer Abdulkadir Ebrahim
	Begetayinoral Kussia Lahole
**Opposed Reviewers:**	
**Response to Reviewers:**	Author response to reviewer<br>Manuscript ID: PONE-D-25-15249<br>Manuscript Title: Prevalence of Stunting and its Determinants Among Children Under Five in 35 Sub-Saharan African Countries (2011–2024): Insights from recent Demographic Health Survey Data Using a Generalized Linear Mixed-Effects Model with Robust Poisson Regression<br>1.Response to Reviewer 1<br><br>Comments<br>Author Response Page number in the manuscript<br>1.I want to thank the editor and the authors for the chance to review this manuscript. The subject of childhood stunting in sub-Saharan Africa is deeply important, and I appreciate the careful work that has gone into this study. It was a privilege to read and reflect on the findings, and I hope my comments will be useful as you move the paper forward.<br>2.General feedback: This paper is very well put together and speaks to a pressing public health concern. The authors draw on DHS data from 35 countries and use appropriate statistical approaches, including robust Poisson regression and mixed-effects modeling, which help account for the survey design and avoid common issues with odds ratios. The sample size is impressive (over 190,000 children), which gives the study strong statistical power and makes the findings more widely applicable. The results are presented clearly, the methods are solid, and the conclusions are well supported by the data. Thank you very much for your encouragement and constructive comments.<br><br>Introduction<br>1.The introduction does a good job of highlighting the global and regional burden of stunting, but it feels crowded with statistics. The numbers are useful, yet they sometimes overwhelm the main message, which should be that stunting remains a serious child health issue in sub-Saharan Africa despite global progress.Revised as commented. 3<br>2.The authors note that their study fills a gap by using pooled data from 35 countries, but this point could be made more directly. At present, it is not fully clear how this analysis differs from single-country or regional studies and what unique value it adds. Readers should come away with a stronger sense of why this pooled approach is important. Revised as commented 3<br>3.The introduction leans heavily on prevalence estimates and less on the “why.” It would be stronger if it included more about the pathways linking maternal, household, and structural factors to stunting. This would help readers understand the reasoning behind the choice of variables and provide a clearer foundation for the study. A conceptual framework showing and visualizing pathways would be complementary. Revised as commented 5

Powered by Editorial Manager® and ProduXion Manager® from Aries Systems Corporation

	4.The section moves quickly between global, regional, and national levels. While this shows the breadth of the issue, the flow could be improved by focusing earlier and more directly on sub-Saharan Africa, since that is the real focus of the paper.Revised as commented 3<br>5.The writing is generally clear but could benefit from light editing to improve flow and fix awkward wording. For example, phrases like "which previous study not accounted incorporated in to analysis" should be revised for clarity.Revised as commented All<br>Methods<br>1.The paper mentions that 23 variables were included, but it is not entirely clear how these were chosen. While prior studies are referenced, the rationale for why these specific variables were prioritized over others could be explained better. Without that clarity, it is hard to know whether the model might be at risk of being overloaded.Revised as commented 8-9<br>2.The manuscript notes that children with missing data were excluded, but it is not clear how the missingness was assessed by the authors or whether its impact on representativeness was considered. Because this is a secondary dataset covering 35 countries, it is possible that patterns in missing data during collection shaped which cases were ultimately included. Adding a short note about this would help bring more clarity and transparency.Revised as commented 8-9<br>3.The study draws on surveys from 35 countries collected over more than a decade. Since survey years and tools may vary a bit, it would be helpful to explain how this variation was handled. For example, what steps were taken to make sure that one country or survey year did not carry too much weight in the pooled results?Revised as commented 6-7<br>4.The study draws on surveys from 35 countries collected over more than a decade. Since survey years and tools may vary a bit, it would be helpful to explain how this variation was handled. For example, what steps were taken to make sure that one country or survey year did not carry too much weight in the pooled results?Revised as commented 6<br>5.The authors mention applying weights, but it is not clear whether these were adjusted for pooling across countries. Since sample sizes vary widely, larger countries may dominate the results if weights are not harmonized. DHS weights make each survey nationally representative, but when pooled they do not automatically align, so rescaling or normalizing is often needed to ensure balanced contributions.Revised as commented 6-7<br>6.The authors also report AIC and BIC, but there is no discussion of whether Poisson assumptions were checked. Because stunting data are often over dispersed, ignoring this can bias standard errors and inferences. Robust Poisson regression helps, but a short note on whether overdispersion was considered would add confidence in the analysis.Revised as commented 11<br>7.Maternal BMI could affect child nutrition, but it could also be shaped by the same household food insecurity or poverty that drives stunting. Similarly, media exposure might influence health behaviors, but access to media is itself determined by socioeconomic status. Without temporal ordering, the relationships in the model can run both ways, which means the results should be interpreted as associations rather than causal effects. Authors should acknowledge the limits of causal interpretation given the cross-sectional design.Revised as commented 33<br>Result<br>1.There are places where the confidence intervals seem disordered (for example, lower bounds listed higher than upper bounds). These appear to be typosRevised as commented 21-22<br>2.In the country level variation, it is not clear whether differences across countries were statistically tested or whether these patterns are being highlighted simply for descriptive interest. Greater clarity here would helpRevised as commented. We added sensitivity analysis, sub-group and meta-regression for heterogeneity 15-19<br>3.Some of the associations are discussed in a way that suggests causal direction (for example, maternal BMI "reduces risk"), but the results come from a cross-sectional dataset. The narrative would be strengthened by consistently framing these as associations rather than causal effects. For instance, maternal BMI is described as being "associated with stunting," but the explanation reads as though underweight mothers cause poor nutrient transfer and overweight or obese mothers prevent stunting by having better food access. Media exposure is also presented as an association, yet the discussion suggests media itself changes behaviors and outcomes. In the same way, antenatal and postnatal care are framed as if they directly

reduce stunting, with wording that children of mothers who missed visits were “more likely” to be stunted because they missed out on nutrition education.Revised as commented 21-24

Discussion

1.Although the authors report associations in the results, parts of the discussion read as if the study demonstrates cause and effect. For example, maternal BMI, media exposure, and health service use are described in ways that imply they directly reduce or increase stunting. Because this is a cross-sectional study, the findings should be framed consistently as associations rather than causal effects. Revised as commented 29-30

2.The discussion does not fully explore the variation across countries. While the pooled analysis is informative, it can mask important differences between contexts. Reflecting on why some countries deviate from the overall pattern, and what that means for interventions, would add more depth.Revised as commented 25-26

3.Policy implications are mentioned but not clearly prioritized. For instance, maternal education, ANC/PNC attendance, and socioeconomic status are all important, but it would help to distinguish which determinants are more feasible targets for immediate policy action and which require longer-term structural investments.Revised as commented 33-34

4.The study is cited alongside some prior work, but the discussion could more strongly situate the findings within the broader literature on child stunting in sub-Saharan Africa. How do these results compare with evidence from longitudinal studies or interventions? This would give readers a clearer sense of what is confirmed, what is new, and what remains uncertain.Revised as commented 24-32

5.The limitations section is rather brief. Issues such as missing data, the cross-sectional design of DHS surveys, and the possibility of residual confounding deserve more explicit acknowledgment. Without this, readers may overestimate the strength of the conclusions.Revised as commented 32-33

6.Much of the interpretation centers on maternal and household factors. Bringing in structural determinants such as poverty, food systems, governance, or conflict would provide a fuller picture. Without this perspective, the discussion risks placing too much emphasis on individual behavior change as the solution. Think structural and systems-level changes. Revised as commented 24-32

2.Response to Reviewer 2

Comments
Author Response Page number in the manuscript

1.Thank you for submitting your manuscript to PLOS ONE. After careful consideration, we feel that it has merit but does not fully meet PLOS ONE’s publication criteria as it currently stands. Therefore, we invite you to submit a revised version of the manuscript that addresses the points raised during the review process.

2.This is a well-conceived and timely study that addresses a critical public health issue—stunting among under-five children in Sub-Saharan Africa (SSA). The manuscript demonstrates a solid grasp of epidemiological analysis and effectively utilizes secondary data from the Demographic and Health Surveys (DHS). The topic aligns with global priorities such as the Sustainable Development Goals (SDG 2.2), and the methodological rigor is commendable. Thank you very much for your encouragement and constructive comments.

General comments

1.You did not line-number your manuscript to help reviewers pinpoint the exact areas requiring revision in the manuscript. Revised as commented All

2.There is inconsistent use of terms for your outcome variable: "stunting among U-5 children", "stunting", "childhood stunting", "stunting among under five children", Revised as commented All

Abstract

1.Your abstract exceeds 300 words, so you need to be parsimonious in the reporting of your findings. You can report the most salient findings: the child's sex, age & birth size; the mother's education; and household wealth, i.e, those with aPR ≥±20%Revised as commented 2-3

2.Please revise this statement "Key variables such as antenatal care visits, postnatal care, and maternal nutritional indicators which previous study not accounted incorporated in to analysis". Suggestion: Key variables such as antenatal care visits, postnatal care, and maternal nutritional indicators, which previous studies did not

account for, are incorporated into the analysis.Revised as commented 2
3.Please revise this statement: "The adjusted prevalence ratio and 95% confidence intervals were used to indicate statistical significance and the strength of associations". Suggestion: "The adjusted prevalence ratios and their 95% confidence intervals were used to assess the strength and statistical significance of associations". Revised as commented 2
4.The abstract results are overloaded with too much information. Kindly reduce the results to the salient ones as suggested above. In fact, the entire multivariate analysis results in the result section need to be rewritten. Again, the term "children older age" is ambiguous, and the aPR value of 2.32, which you stated, is misleading. It is better to say: "being aged 12 months or older" (aPR: ≥1.81, p<0.01). Also, "mother without a higher education (aPR: ≥1.68, p<0.01), and living in a poor or average household (aPR: ≥1.23, p<0.01). Revised as per the comments. We tried as much as possible. 2
Methods
1.I suggest you merge the poorest and the poorer categories into one category, "poor", and merge the richer and the richest into one category as "rich", thus you will have three categories for the wealth index. This will provide a much better explanation of your findings. Revised as commented 10
2.The 2nd paragraph on Page 8: I see that you included "source of drinking water", "type of toilet facility", and "type of cooking" in your independent variable list. I suggest you exclude these variables because they have already been used to estimate the household wealth index (composite index). Using them again with the household wealth index in the model may not be statistically appropriate. Revised as commented 10
3.The first paragraph on Page 9: You mentioned that you used a multilevel mixed-effects Poisson regression. Later on in the paragraph, you mentioned that you applied a mixed-effects logistic regression model. Kindly review and revise the entire paragraph. Revised as commented 9-10
Result
1.I notice a haphazard presentation of your findings. Your presentation needs to be systematic and must align with the order of arrangement of the factors in the Tables. Revised as commented 21-24
2.Page 14: In the first paragraph, you might need to review this statement: "The ICC (Intraclass Correlation Coefficient) value from the null model revealed that only about 26.8% of the total variance in stunting among under five children is attributable to differences between clusters, while the remaining 73.2% was attributed to individual-level factors". How was the remaining attributed to individual-level factors alone, and what about the household and contextual factors?Revised as commented 20-21
3.Page 15, 1st paragraph: This statement totally negates what is in Table 4: "The prevalence of stunting was 16% higher (aPR = 1.16, 95% CI: 1.13, 1.19) among children perceived by their mothers as having a small size at birth and 48% higher (aPR = 1.48, 95% CI: 1.43, 1.52) among those perceived as having an average size at birth." Revised as commented All pages
4.Page 15, 3rd paragraph: The entire paragraph needs to be rewritten because it is ambiguous, and the findings are lumped together. Kindly use a sentence to present each finding, stating the reference category for each finding.   Revised as commented 21-24
5.Table 4 has no notation for the asterisk ** (page 18) Revised as commented 24
Discussion section
1.Page 21, Paragraph 2: Please revise the grammatical structure of this statement: "Study revealed that, postnatal visit associated with child stunting. Mothers who did not attend postnatal care services were 1.03 more likely to have a stunted child compared to those who did attend postnatal care. This finding consistent with study conducted in Ethiopia (91)." Revised as commented 30
2.The discussion lacks coherence. For instance, begin with ANC, then address the Place of delivery, followed by PNC (pages 21-22). Revised as commented 21-24
3.Page 22, paragraph 4: "This study found that, maternal body mass index associated with stunting". "was" is missing. Revised as commented

3.Response to Journal Requirements:

Comments
Author Response Page number in the manuscript

Powered by Editorial Manager® and ProduXion Manager® from Aries Systems Corporation

	1.Please ensure that your manuscript meets PLOS ONE's style requirements, including those for file naming. The PLOS ONE style templates can be found at: https://journals.plos.org/plosone/s/file?id=wjVg/PLOSOne_formatting_sample_main_body.pdf and https://journals.plos.org/plosone/s/file?id=ba62/PLOSOne_formatting_sample_title_authors_affiliations.pdf<br>Revised as commented All<br>2.We noticed you have some minor occurrence of overlapping text with the following previous publication(s), which needs to be addressed:<br>https://journals.plos.org/plosone/article?id=10.1371/journal.pone.0248637<br>https://bmcpregnancychildbirth.biomedcentral.com/articles/10.1186/s12884-024-07105-7 . In your revision ensure you cite all your sources (including your own works), and quote or rephrase any duplicated text outside the methods section. Further consideration is dependent on these concerns being addressed. Revised as commented All<br>3.Please note that PLOS ONE has specific guidelines on code sharing for submissions in which author-generated code underpins the findings in the manuscript. In these cases, we expect all author-generated code to be made available without restrictions upon publication of the work. Please review our guidelines at https://journals.plos.org/plosone/s/materials-and-software-sharing#loc-sharing-code and ensure that your code is shared in a way that follows best practice and facilitates reproducibility and reuse. Our manuscript does not strictly require code sharing because no novel code underpins in our findings All<br>4.In the online submission form you indicate that your data is not available for proprietary reasons and have provided a contact point for accessing this data. Please note that your current contact point is a co-author on this manuscript. According to our Data Policy, the contact point must not be an author on the manuscript and must be an institutional contact, ideally not an individual. Please revise your data statement to a non-author institutional point of contact, such as a data access or ethics committee, and send this to us via return email. Please also include contact information for the third-party organization, and please include the full citation of where the data can be found. Revised as commented 36<br>5.Please amend either the abstract on the online submission form (via Edit Submission) or the abstract in the manuscript so that they are identical. Revised as commented 2-3<br>6.Your ethics statement should only appear in the Methods section of your manuscript. If your ethics statement is written in any section besides the Methods, please delete it from any other section. Revised as commented 12
**Additional Information:**	
**Question**	**Response**
**Financial Disclosure**<br><br>Enter a financial disclosure statement that describes the sources of funding for the work included in this submission. Review the submission guidelines for detailed requirements. View published research articles from *PLOS ONE* for specific examples.<br><br>This statement is required for submission and **will appear in the published article** if the submission is accepted. Please make sure it is accurate.	The author(s) received no specific funding for this work.

**Funded studies**<br>Enter a statement with the following details:<br>• Initials of the authors who received each award<br>• Grant numbers awarded to each author<br>• The full name of each funder<br>• URL of each funder website<br>• Did the sponsors or funders play any role in the study design, data collection and analysis, decision to publish, or preparation of the manuscript?<br><br>Did you receive funding for this work?	
**Competing Interests**<br><br>Use the instructions below to enter a competing interest statement for this submission. On behalf of all authors, disclose any competing interests that could be perceived to bias this work—acknowledging all financial support and any other relevant financial or non-financial competing interests.<br><br>This statement is required for submission and **will appear in the published article** if the submission is accepted. Please make sure it is accurate and that any funding sources listed in your Funding Information later in the submission form are also declared in your Financial Disclosure statement.<br><br>View published research articles from *PLOS ONE* for specific examples.	The authors have declared that no competing interests exist.

**NO authors have competing interests**<br><br>Enter: *The authors have declared that no competing interests exist.*<br><br>**Authors with competing interests**<br><br>Enter competing interest details beginning with this statement:<br><br>*I have read the journal's policy and the authors of this manuscript have the following competing interests: [insert competing interests here]*<br><br>* typeset	
**Ethics Statement**<br><br>Enter an ethics statement for this submission. This statement is required if the study involved:<br><br>• Human participants<br>• Human specimens or tissue<br>• Vertebrate animals or cephalopods<br>• Vertebrate embryos or tissues<br>• Field research<br><br>Write "N/A" if the submission does not require an ethics statement.<br><br>General guidance is provided below. Consult the submission guidelines for detailed instructions. **Make sure that all information entered here is included in the Methods section of the manuscript.**	For this study, we utilized Demographic and Health Survey (DHS) data from 35 sub-Saharan African countries. The DHS survey procedures were approved by the ICF Institutional Review Board (IRB) and the respective host country IRB; therefore, no additional ethical approval was required for this secondary analysis. The dataset accessed contain no identifiable participant information, ensuring confidentiality and privacy. Access to the data was formally authorized by the DHS program, which serve as the institutional custodian of the datasets, through an online request submitted at http://www.dhsprogram.com and supported by authorization letter AuthLetter_215093 on dated 01/19/2025.

Powered by Editorial Manager® and ProduXion Manager® from Aries Systems Corporation

**Format for specific study types**<br><br>**Human Subject Research (involving human participants and/or tissue)**<br>• Give the name of the institutional review board or ethics committee that approved the study<br>• Include the approval number and/or a statement indicating approval of this research<br>• Indicate the form of consent obtained (written/oral) or the reason that consent was not obtained (e.g. the data were analyzed anonymously)<br><br>**Animal Research (involving vertebrate animals, embryos or tissues)**<br>• Provide the name of the Institutional Animal Care and Use Committee (IACUC) or other relevant ethics board that reviewed the study protocol, and indicate whether they approved this research or granted a formal waiver of ethical approval<br>• Include an approval number if one was obtained<br>• If the study involved *non-human primates*, add *additional details* about animal welfare and steps taken to ameliorate suffering<br>• If anesthesia, euthanasia, or any kind of animal sacrifice is part of the study, include briefly which substances and/or methods were applied<br><br>**Field Research**<br><br>Include the following details if this study involves the collection of plant, animal, or other materials from a natural setting:<br>• Field permit number<br>• Name of the institution or relevant body that granted permission	
**Data Availability**<br><br>Authors are required to make all data underlying the findings described fully available, without restriction, and from the time of publication. PLOS allows rare exceptions to address legal and ethical concerns. See the PLOS Data Policy and FAQ for detailed information.	Yes - all data are fully available without restriction

A Data Availability Statement describing where the data can be found is required at submission. Your answers to this question constitute the Data Availability Statement and **will be published in the article**, if accepted.<br><br>**Important:** Stating 'data available on request from the author' is not sufficient. If your data are only available upon request, select 'No' for the first question and explain your exceptional situation in the text box.<br><br>Do the authors confirm that all data underlying the findings described in their manuscript are fully available without restriction?	
**Describe where the data may be found in full sentences. If you are copying our sample text, replace any instances of XXX with the appropriate details.**<br><br>• If the data are **held or will be held in a public repository**, include URLs, accession numbers or DOIs. If this information will only be available after acceptance, indicate this by ticking the box below. For example: *All XXX files are available from the XXX database (accession number(s) XXX, XXX.*).<br>• If the data are all contained **within the manuscript and/or Supporting Information files**, enter the following: *All relevant data are within the manuscript and its Supporting Information files.*<br>• If neither of these applies but you are able to provide **details of access elsewhere**, with or without limitations, please do so. For example:<br><br>*Data cannot be shared publicly because of [XXX]. Data are available from the XXX Institutional Data Access / Ethics Committee (contact via XXX) for researchers who meet the criteria for access to confidential data.*<br><br>*The data underlying the results presented in the study are available from (include the name of the third party*	The datasets analyzed during current study are publicly available from the Demographic and Health Survey (DHS) program, managed by ICF international. The data can be accessed upon reasonable request through the DHS program website at http://www.dhsprogram.com. Researcher must register and submit a data access request through the site. For inquires regarding data access, please contact the DHS Data Archivist at archive@dhsprogram.com. The data used in this study are cited as: ICF. Demographic and Health Surveys (various years). Funded by USAID. Rockville, Maryland. Available at: http://dhsprogram.com.

Powered by Editorial Manager® and ProduXion Manager® from Aries Systems Corporation

and contact information or URL).<br>• This text is appropriate if the data are owned by a third party and authors do not have permission to share the data.<br><br>* typeset	
Additional data availability information:	

Powered by Editorial Manager® and ProduXion Manager® from Aries Systems Corporation

COVER LETTER

For Plos One

Mr. Abdulkerim Hassen Moloro
Samara University
Semera, Ethiopia

March 21, 2025

Dear Editors,

We wish to submit an original research (multilevel analysis of recent DHS in 35 sub-Saharan Africa) article entitled "**Pooled Prevalence of Stunting and its Determinants Among Children Under Five in 35 Sub-Saharan African Countries (2011–2024): Insights from recent Demographic Health Survey Data Using a Generalized Linear Mixed-Effects Model with Robust Poisson Regression**" for consideration by **PLOS ONE.**

We confirmed that this work is original and has not been published elsewhere, nor is it currently under consideration for publication elsewhere. This paper reports on the pooled prevalence of stunting and its determinants among under five children in 35 sub-Saharan Africa using recent DHS data and advanced statistical model. We believe that this multilevel analysis is appropriate for publication in **PLOS ONE** because it fulfils journal aims and scope.

Despite efforts by initiatives like the World Bank's 'All Hands-on Deck' and UNICEF's programs to address stunting through multisectoral approaches, the burden of stunting remains alarmingly high in sub-Saharan Africa. This study utilized recent large-scale survey data from 35 SSA countries (2011–2024) to estimate the pooled prevalence of stunting and its determinants among children under five. Key variables such as antenatal care visits, postnatal care, and maternal nutritional indicators which previous study not accounted incorporated in to analysis.

Therefore, this study aimed to determine the pooled prevalence of stunting and its determinants among under five children in 35 SSA countries based on the recent(2011-2024) DHS data using advanced statistical model and the findings may assist the Sustainable Development Goal (SDG3) in identifying gaps and deciding how to meet the 2030 target of reducing child malnutrition by 2.2 target as well as be used by health planners, policymakers, and the community to include the improvement of child stunting in SSA countries.

Authors declared no conflicts of interest.

Email: habdulkerim4@gmail.com .

Thank you for your consideration of this study.

Sincerely, Abdulkerim Hassen Moloro

Manuscript
Click here to access/download;Manuscript;Manuscript Revised.docx

Prevalence of Stunting and its Determinants Among Children Under Five in 35 Sub-Saharan African Countries (2011–2024): Insights from recent Demographic Health Survey Data Using a Generalized Linear Mixed-Effects Model with Robust Poisson Regression

Author's Name and Email

[1]Abdulkerim Hassen Moloro*, Email: habdulkerim4@gmail.com

[1]Kebede Gemeda Sabo, Email: kebedegemeda2008@gmail.com

[1]Kusse Urmale Mare, Email: kussesinbo@gmail.com

[2]Beriso Furo Wengoro, Email: berisafuro@gmail.com

[3]Eshetu Elfios Endrias, Email: eshetuelf21@gmail.com

[4]Roda Mehadi Ibrahim, Email: rozimahdi04@gmail.om

[5]Teshager Dubie, Email: teshagerdubie19@gmail.com

[6]Oumer Abdulkadir Ebrahim, Email: oumerbadana@gmail.com

[7]Begetayinoral Kussia Lahole, Email: begetakussia92@gmail.com

Authors Affiliation

[1]Department of Nursing, College of Medicine and Health Sciences, Samara University, Samara, Ethiopia

[2]Department of Biomedical Sciences, College of Medicine and Health Sciences, Samara University, Samara, Ethiopia

[3]Department of Nursing, School of Nursing, College of Medicine and Health Sciences, Wolaita Sodo University, Wolaita Sodo, Ethiopia

[4]Department of midwifery, College of Medicine and Health Sciences, Jigjiga University, Jigjiga, Ethiopia

[5]Department of Veterinary Medicine, College of Veterinary Medicine and Animal Sciences, Samara University, Semera, Ethiopia

[6]Department of Veterinary Medicine, College of Veterinary Medicine and Anima Science, Samara University, Samara, Ethiopia

[7]Department of Midwifery, College of Medicine and Health Sciences, Arba Minch University, Arba Minch, Ethiopia

Corresponding Authors

Abdulkerim Hassen Moloro*,
Department of Nursing
College of Medicine and Health Science
Samara University
Samara, Ethiopia
Email: habdulkerim4@gmail.com, ORCID: https://orcid.org/0000-0002-6081-4108

Abstract

Background: Despite efforts by initiatives like the World Bank's 'All Hands-on Deck' and UNICEF's programs to address stunting through multisectoral approaches, the burden of stunting remains alarmingly high in sub-Saharan Africa. This study utilized recent large-scale survey data from 35 SSA countries (2011–2024) to estimate the pooled prevalence of stunting and its determinants among children under 5 years of age. Key variables such as antenatal care visits, postnatal care, and maternal nutritional indicators, which previous studies did not account for, are incorporated into the analysis.

Methods: A secondary analysis was conducted using recent demographic and health survey data (2011–2024) from 35 sub-Saharan African (SSA) countries. A total weighted sample of 191,953 children under 5 years of age was included in the analysis. Descriptive and inferential analyses were performed using STATA 17. Forest plots were utilized to illustrate both pooled and country-specific stunting rates. Determinants of stunting were identified through a multilevel mixed-effects Poisson regression model with robust variance. The adjusted prevalence ratios and their 95% confidence intervals were used to assess the strength and statistical significance of associations. Model comparison was evaluated by model statistical summary such as Akaike's Information Criterion (AIC), Bayesian Information Criterion (BIC), log-likelihood (LL), and deviance (-2*LL).

Result: The pooled prevalence of stunting among children under 5 years of age in 35 sub-Saharan African countries was 29.89% (95% CI: 26.63, 33.14%), with the lowest level in Gabon (13.91%) and the highest in Burundi (55.80%). Being male children (aPR =1.24, 95% CI: 1.21–1.26), being aged 12 months or older (aPR: ≥1.81, p<0.01), insufficient antenatal care (ANC) visits (aPR: ≥1.17, p<0.01), lack of postnatal visits(aPR = 1.03, 95% CI: 1.07, 1.05), children perceived as small or average at birth (aPR: ≥1.16, p<0.01), mother without a higher education (aPR: ≥1.94, p<0.01), living in a poor or average wealth household (aPR: ≥1.23, p<0.01) were significant predictors of stunting. Conversely, maternal overweight (aPR = 0.81, 95% CI: 0.77–0.84) and obese mothers (aPR = 0.88, 95% CI: 0.85–0.90) were associated lower prevalence of stunting.

Conclusion: Study revealed a stunting prevalence of 29.89% among children under 5 years of age across 35 sub-Saharan African countries, with significant country-level variations and rates exceeding 30% in 15 countries, signaling a major public health concern. Key individual, household, and contextual factors associated with stunting were identified, emphasizing the need

for immediate actions expanding antenatal and postnatal care, promoting facility-based deliveries, enhancing maternal education, and media outreach. Long term strategies must tackle poverty, food systems, and equitable nutrition access, supported by governance and stability. A multisectoral approach integrating health, education, agriculture, WASH, and social protection ensures substantiable child growth, complemented by longitudinal research for policy coherence.

Keywords: Stunting, Under-five, Demographic Health Survey, Sub-Saharan Africa

Introduction

Stunting defined as a length/height-for-age measurement below -2 Standard Deviations from the median(1), remains a critical and persistent public health crisis in sub-Saharan Africa (SSA). It is the result of chronic malnutrition, a condition that compromises physical function and the maintenance of key bodily processes(2), and it leads to significant impairments in physical, psychological, and cognitive growth(3).

Sub-Saharan Africa carries a disproportionately heavy burden of childhood stunting, with pooled data from 35 countries indicating an pooled prevalence of 35%(4). National disparities are remarkable, ranging from as low as 17% in Gabon to as high as 59% in Burundi(5), while Nigeria reports a prevalence of 36.5% among children under five(6). This situation persists despite a global decline in stunting rates, as the absolute number of affected children in Africa has risen(7), underscoring a regional emergency within the worldwide context of 161 million stunted children(8).

The consequences in SSA are severe and lifelong, including increased child morbidity and mortality, impaired development, and higher disease risk(9, 10). Research has identified that stunting arises from a wide range of interrelated causes, which can be categorized into individual-level, household, and contextual factors. At the individual level, determinants include the child's sex and age, maternal age and educational attainment, and birth interval(11, 12). Additional contributors such as birth order (13), antenatal care utilization (14), and duration of exclusive breastfeeding(13, 15-19) have also been identified. Furthermore, studies report that inadequate food intake, low perceived birth weight, maternal body mass index (BMI), and poor sanitation significantly increase the risk of childhood stunting(20-22).

Household-level determinants encompass household size, particularly households with multiple under-five children (14, 16, 23), access to improved drinking water sources(15), and availability of toilet facilities(24) and household wealth index(23, 25). Contextual factors including place of residence(14) and geographical sub-region(11, 12) further affects child nutrition outcomes, underscoring the multifaceted nature of stunting and its dependence on both biological and socio-environmental conditions(Fig 1).

Stunting remains a major child health challenge in sub-Saharan Africa (SSA), despite decades of global and regional initiatives aimed at reducing malnutrition. Programs such as the World Bank's "All Hands-on Deck" initiative, which promotes multisectoral strategies across agriculture, education, social protection, and WASH sectors, and UNICEF's maternal and child nutrition programs have sought to accelerate progress(26, 27). Nevertheless, the burden of the stunting in SSA continues to be alarming high, and many countries are unlikely to achieve the Sustainable Developmental Goal (SSD) 2.2 target of reducing stunting by 40% by 2025 and eliminating all forms of malnutrition by 2030(28, 29).

While single country and regional studies have provided valuable insights, their scope is often limited, making it difficult to capture the broader patterns and disparities across the content. This study addresses that gap pooling nationally representative data from 35 SSA countries collected between 2011 and 2024. By combining data at this scale, the analysis enhances statistical power using Poisson regression with robust error variance(30, 31) to reduce potential overestimation of odds ratios for common binary outcomes, improves generalizability, and allows for the identifications of common risk factors and cross-country variations that may not be visible in isolated studies.

Additionally, this analysis incorporated key variables such as antenatal care (ANC) visits during pregnancy, postnatal care visits, and maternal nutritional status indicators including BMI and height factors that were not considered in previous studies(4). By integrating these variables, the pooled evidence is uniquely positioned to inform regional policy, guide resource allocation, and support collaborative interventions across countries. This study therefore provides a more comprehensive and representative picture of stunting in sub-Saharan Africa (SSA), underscoring the urgent need for coordinated, evidence-based strategies to address this persistent public health challenge. Accordingly, the study aimed to determine the pooled prevalence and key determinants

of stunting among children under 5 years of age in 35 SSA countries, utilizing generalized linear mixed-effects modeling with robust Poisson regression analysis based on the most recent 2011–2024 Demographic and Health Survey (DHS) data

Conceptual frame work

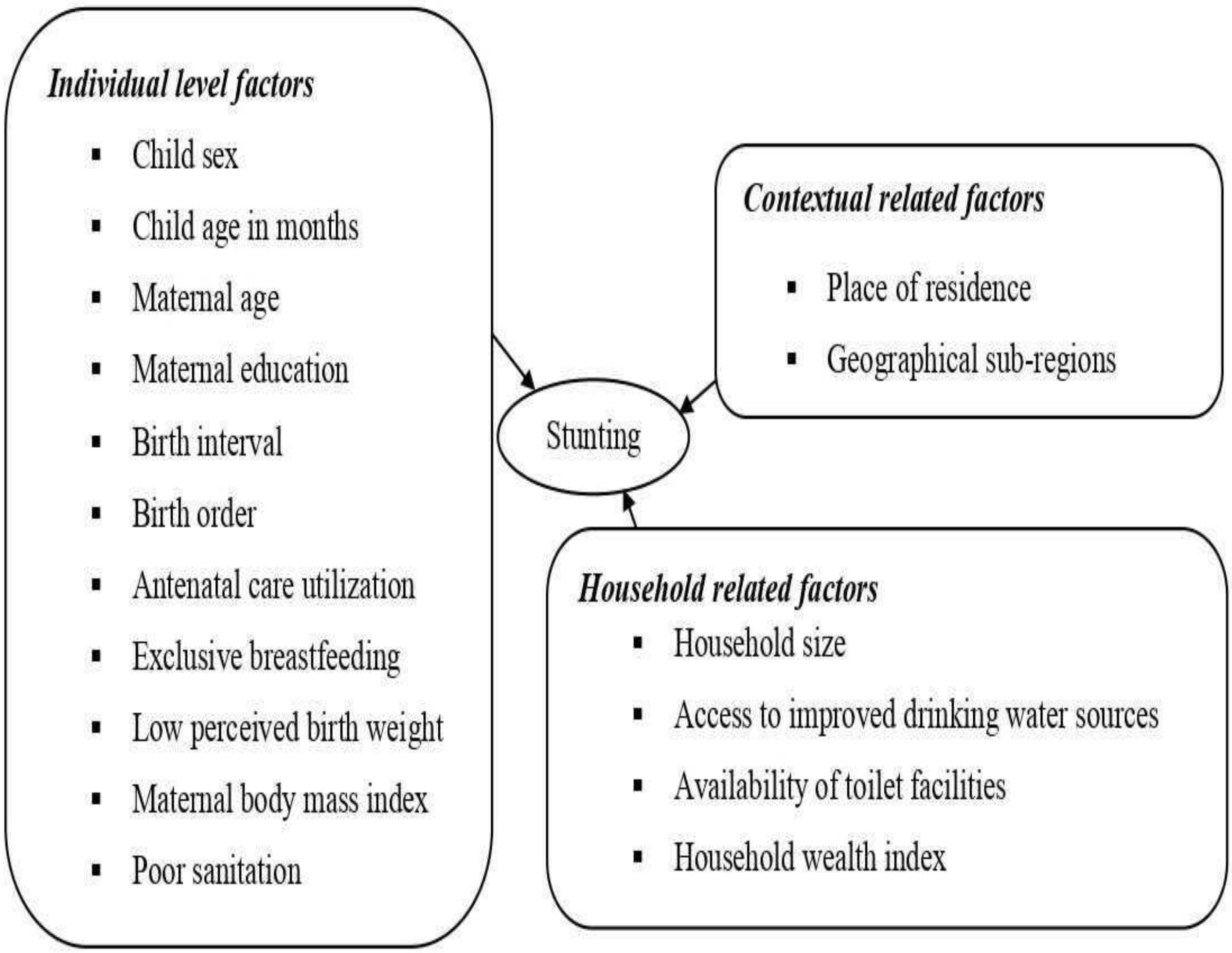

Figure 1: A conceptual framework showing link between stunting and its determinants among children under 5 years of age, 2011-2024.

Methods

Study Area, Design, and Period

This study conducted secondary analysis using data from the Demographic and Health Surveys (DHS) of 35 countries in Sub-Saharan Africa (SSA). The countries involved were Angola, Burkina

Faso, Benin, Burundi, DR Congo, Republic of the Congo, Côte d'Ivoire, Cameroon, Ethiopia, Gabon, Ghana, Gambia, Guinea, Kenya, Comoros, Liberia, Lesotho, Madagascar, Mali, Mauritania, Malawi, Mozambique, Nigeria, Niger, Namibia, Rwanda, Sierra Leone, Senegal, Chad, Togo, Tanzania, Uganda, South Africa, Zambia, and Zimbabwe. The selection of country was based on the recent survey year, availability of a standardized and unrestricted dataset, and presence of observations for the outcome variable in the datasets.

The DHS surveys across all countries employed a cross-sectional study design to collect data on basic sociodemographic characteristics and various health indicators, including nutrition, maternal, and child health. For the current analysis, we included the countries that have their recent DHS conducted between 2011 and 2024. Since its inception, DHS has been conducted in over 85 low- and middle-income countries (LMICs) worldwide.

Population, sampling technique and weight

The source population for this study comprised all children under five years old in 35 SSA countries. The study population included all children under 5 years of age in the survey. Across all countries, the surveys used a multistage stratified cluster sampling technique to select the study participants. In the first stage, each country was divided into clusters, and clusters were randomly selected based on the probability proportional to their contribution to overall country’s population. In the second stage, using the housing census as a sampling frame, a representative number of households was selected from each cluster.

To account for the complex survey design, non-response, and to ensure comparability across countries while preventing any single country or survey year from disproportionally influencing the pooled estimates, DHS sampling weights were applied as recommended. The weight variable (v005) was rescaled by dividing by 1,000,000 and incorporated into all analyses using the *svyset* command in Stata, accounting for primary sampling units (v021) and strata (v022). This produced a weighted analytic sample of **191, 953** children aged 0 to 59 months with complete data on the variables of interest (Table 1). Additionally, sensitivity analyses were conducted to assess the influence of individual countries by systematically excluding one country at a time and recalculating the pooled prevalence. This approach adjusts for unequal probabilities of selection

and non-response within each survey, ensuring that each country's contribution to the pooled prevalence is proportional to its population size.

Table-1: Survey years and sample sizes of children under 5 years of age from 35 Sub-Saharan African (SSA) countries included in the study, from 2011 to 2024

Country	Survey year	Unweighted sample size	Weighted sample size
**Angola**	2023-2024	5,039	4,800
**Burkina Faso**	2021	5,731	5,696
**Benin**	2017-2018	11,670	11,717
**Burundi**	2016-2017	6,048	6,222
**DR Congo**	2023-2024	10,745	9,734
**Congo**	2011-2012	4,475	3,859
**Cote d'Ivoire**	2021	4,779	4,300
**Cameroon**	2018-2019	4,497	4,707
**Ethiopia**	2019	5,076	4,972
**Gabon**	2019-2021	5,343	5,009
**Ghana**	2022-2023	4,405	4,051
**Gambia**	2019-2020	3,811	3,511
**Guinea**	2018	3,466	3,374
**Kenya**	2022	17,327	15,336
**Comoros**	2012	2,387	2,468
**Liberia**	2019-2020	2,445	2,166
**Lesotho**	2023-2024	1,093	983
**Madagascar**	2021	5,778	5,719
**Mali**	2023-2024	13,936	14,163
**Mauritania**	2019-2021	9,830	9,955
**Malawi**	2015-2016	5,149	5,160
**Mozambique**	2022-2023	3,733	3,858
**Nigeria**	2023-2024	9,410	9,341
**Niger**	2012	5,576	5,112
**Namibia**	2013	1,558	1,438
**Rwanda**	2019-2020	3,809	3,918
**Sierra Leone**	2019	4,136	4,044

**Senegal**	2023	4,476	4,258
**Chad**	2014-2015	9,826	9,805
**Togo**	2013-2014	3,185	3,058
**Tanzania**	2022	4,807	4,866
**Uganda**	2016	4,423	4,350
**South Africa**	2016	1,113	1,080
**Zambia**	2024	3,810	3,710
**Zimbabwe**	2015	4,957	5,212
**Total**		**197,044**	**191,953**

Data source

The data for this study were obtained from the DHS women's questionnaire, focusing on children under five years of age (under 60 months), and from the Kids Record dataset (KR file) across 35 countries. All datasets were sourced directly from the official Demographic and Health Surveys (DHS) program website (http://dhsprogram.com/).

Data extraction and management of missing observations

Prior to data extraction, we identified countries with DHS datasets from surveys conducted between 2011 and 2024, as our aim was to include only those with data collected during this timeframe. A standardized data collection tool and face-to-face interview were used to collect the survey's data. To construct a pooled dataset, we first extracted variables relevant to the study from the data of the children under 5 years of age (under 60 months) from the Kids Record dataset (KR file) across 35 countries. The merged datasets from 35 sub-Saharan African countries were then appended by survey year, creating a pooled datasets for regional analysis of child stunting. Country identifiers and survey weights were retained to account for sampling design and cross-country differences. Variables are then recoded and categorized consistently across all 35 DHS surveys using the Guide to DHS statistics to ensure compatibility.

The handling of anthropometric data in this analysis adhered to the standardized protocols outlined in the Guide to DHS Statistics(32). Children were excluded or dropped from the analyses of all anthropometric indices if they met any of the following criteria: (i) they were not weighed or measured during the survey, (ii) their recorded weight or height measurements were missing, (iii)

their month or year of birth was unknown or missing (rendering accurate age calculation impossible for indices requiring it), or (iv) their derived height-for-age z-scores (HAZ) were flagged as biologically implausible (e.g., outside the range of -6 to +6 SD). These exclusions applied uniformly to both the denominator and numerator in prevalence analysis, constituting a complete-case analysis.

For the independent variables, complete case analysis was employed, retaining only observations with non-missing values for all covariates included in the final model. These steps enhance the transparency and validity of our findings in light of potential cross-country variations in data completeness. A detailed explanation of the DHS methodology, guidelines, and procedures for handling missing data is available on the DHS website(33).

Variables and measurements

Dependent Variable:

Stunting (height-for-age z-scores): height for age of children dichotomized as normal (not stunted) if height for age ≥-2 SD and stunted if height of age <-2 SD form WHO child growth reference ((height/age standard deviation (new who)). Weight measurements were obtained using light weight SECA mother-infant scales with a digital screen designed and manufactured under the guidance of UNICEF. Height measurements were obtained using the Shorr measuring board. Children younger than 24-months were measured for their height while lying down, and children older than 24 months were measured while standing.

Independent Variables: A total of 23 variables were incorporated into this study, selected through a systematic process that considered both their availability in the Demographic and Health Survey (DHS) and their documented associations with childhood stunting in prior research(34-36). Each variable was chosen for its relevance to the study objectives and its role as a known or hypothesized determinants of stunting reflecting influences at individual, household, and contextual level. This study also included variables often overlooked in earlier work, such as antenatal care (ANC) visits during pregnancy postnatal care utilizations, and body mass index.

These variables were categorized into individual-level factors, household factors, and contextual factors. The individual-level factors included the sex of the child (male and female), age of the child in month, birth order (1, 2–4, and 5+), breastfeeding (still breastfeeding, ever breastfed and

never breastfed) and perceived size at birth (large, average, and small). Other individual-level factors were maternal educational level (no formal education, primary, secondary, and higher), current maternal working status (yes and no), number of antenatal care visits during pregnancy (0, 1–3, and 4 or more) and postnatal checks within 2 months (yes and no). Variables such as maternal age (15–24 and 25–49), age at first birth, antenatal visits during pregnancy (0, 1–3, and 4 or more), place of delivery (home, health facility, other), marital status (single and married) and maternal nutritional status were also considered.

Household factors included household size (small, medium, and large), exposure to media (yes and no), and wealth index (poor, middle, rich). Contextual factors encompassed the place of residence (urban and rural) and geographical sub-regions (West, East, Central, and Southern).

Maternal nutritional status, described using Body Mass Index (BMI) according to the WHO adult BMI classification(37). BMI was calculated by dividing each woman's weight in kilograms by the square of her height in meters (kg/m^2). The survey utilized an electronic SECA 874 flat scale for weight measurements, specially designed for mobile use, and a shore measuring board for height. For analysis, women were categorized into four nutritional status groups based on their BMI: underweight (BMI < 18.5 kg/m^2), normal weight (BMI 18.5–24.9 kg/m^2), overweight (BMI 25.0–29.9 kg/m^2), and obesity (BMI ≥ 30.0 kg/m^2)(38).

Data management and statistical analysis

Stata version 17 was utilized for data cleaning and analysis. Prior to the analysis, the presence of the outcome variable in the DHS dataset for each country was confirmed. All the variables considered in the study were reviewed for missing values. Subsequently, the datasets from 35 SSA countries were appended and weighted to maintain sample representativeness and obtain reliable estimates and standard errors. The pooled prevalence of child stunting was calculated using weighted data on the number of affected children with outcome variable and the total number of study participants in each country included in the analysis. The Stata command for meta-analysis "metan" was executed to present the country-specific and pooled estimates with 95% CI in a forest plot. To explore potential sources of heterogeneity, meta-regressions were conducted(39). Additionally, sensitivity analyses were performed to evaluate the influence of individual country on the overall pooled estimates (39).

A multilevel mixed-effects Poisson regression model with robust error variance was fitted to identify determinants of stunting among children under five in SSA. This model was selected for two primary reasons. First, it directly estimates prevalence ratios, which are more interpretable and avoid the overestimation of association strength common with odds ratios from logistic regression when applied to common binary outcomes in cross-sectional data(31, 40). Second, its multilevel framework accounts for the hierarchical structure of the DHS data, where children are nested within households, and households within clusters, by including random intercepts at the household and cluster levels. Bivariable multilevel robust Poisson regression analysis was done and all variables with a p-value of less than 0.25 in this analysis were considered for multivariable multilevel robust Poisson regression model (31, 40).

Prior to model fitting, we assessed the key assumption of the Poisson regression model. A key assumption of this model is that observations are independent and that the mean and variance of the dependent variable are equal. Model adequacy was then confirmed through goodness-of-fit tests: the deviance statistic (151,692.69) and Pearson statistic (83,880.11) both yielded p-values of 1.000, indicating an excellent fit. The data had a mean of 0.306 and a variance of 0.294, producing a mean-to-variance ratio of approximately 1.04. This close alignment supports the equidispersion assumption and validates the use of the Poisson model. Based on these findings, we applied Poisson regression to examine the effects of predictors on stunting, while carefully monitoring for influential observations and residual patterns(41). Model fit was further assessed using Akaike (AIC) and Bayesian (BIC) Information Criteria, with lower values indicating better fit and parsimony.

In our analysis, five hierarchal models were fitted to select the model that best fits the data: null-model (a model with only outcome variable to assess the random variability in the intercept), model-I (a model with only individual-level explanatory variables), model-II (a model with only household-level explanatory variables), and model-III (a model with contextual-level factors) and model-IV (a model with only potential candidate variables from individual, household, and contextual-level factors). The " meglm" command in Stata was used to fit these models.

Random variability in stunting across clusters was assessed by intra-class correlation coefficient (ICC), explained variance or proportion change in variance (PCV), and median odds ratio (MOR). Akaike's information criteria (AIC), Bayesian information criteria (BIC), Log-likelihood (LL),

and deviance (i.e.-2*LL) values were used for model comparison. The presence of multicollinearity between explanatory variables was checked using variance inflation factor values and the values for the included variables ranged from **1.01 to 1.95,** suggesting that there was no multi-collinearity. Finally, in the multivariable analysis, a p-value less than 0.05 and an adjusted prevalence ratio with the corresponding 95% confidence interval was used to identify the factors associated with stunting among children under 5 years of age in 35 SSA countries.

Ethical considerations

For this study, we utilized Demographic and Health Survey (DHS) data from 35 sub-Saharan African countries. The DHS survey procedures were approved by the ICF Institutional Review Board (IRB) and the respective host country IRB; therefore, no additional ethical approval was required for this secondary analysis. The dataset accessed contain no identifiable participant information, ensuring confidentiality and privacy. Access to the data was formally authorized by the DHS program, which serve as the institutional custodian of the datasets, through an online request submitted at http://www.dhsprogram.com and supported by authorization letter AuthLetter_215093 on dated 01/19/2025.

Results

The study included and analyzed a weighted sample of 191,953 mothers with children aged 0 to 59 months. Of these children, 50.49% (96,907) were male, and 20.01% (42,185) were under 12 months old. More than half of the children, 55.34% (96,976), had been breastfed at some point but were no longer breastfeeding. Stunting was highly prevalent, affecting 21.98% (42,185) of children under 12 months, compared to older age groups. Among the included children, 65.63% (125,969) lived in rural areas, and 53.29% (25,499) were from households in the poor wealth index category. Additionally, 42.23% (81,068) of the children were from West African countries, and 31.51% (56,237) were delivered at home (Table 2).

Of the 191,953 mothers included in the study, 139,744 (72.80%) were between 25 and 49 years old, and 149,142(77.70%) were currently married. Over one-third of the mothers, 71,108(37.04%), had no formal education, while only 8,824(4.60%) had completed higher education. The majority, 110,643(57.64%), had their first child before the age of twenty, and 126,538 (65.92%) had access to mass media. Regarding nutritional status, 12,591(6.56%) of the mothers were underweight,

while 91,612(47.73%) had a body mass index (BMI) within the normal range. Additionally, 26,149 (13.62%) were overweight, and 61,600(32.09%) were classified as obese

Table 2: Sociodemographic characteristic of the children under 5 years of age by stunting and their overall background status, in 35 SSA countries, 2011–2024 (n = **191,953**)

Characteristics	Weighted Frequency (%)	Stunting
**Sex of the child**		
Male	96,907(50.49)	31,604(32.61)
Female	95,045(49.51)	26,642(28.03)
**Age of the child (months)**		
Less than 12 months	42,185(21.98)	7,156(16.96)
12–23 months	39,907(20.79)	13,492(33.81)
24–35 months	36,961(19.26)	14,225(38.49)
36–47 months	37,708(19.64)	13,108(34.76)
48–59 months	35,190(18.33)	10,264(29.17)
**Birth order**		
First	41,755(21.75)	11,640(27.88)
2 to 4	94,840(49.41)	27,615(29.12)
5 or higher	55,358(28.84)	18,991(34.31)
**Breastfeeding**		
Ever breastfeed	96,976(55.34)	34,331(35.40)
Never breastfeed	6,870(3.92)	2,149(31.28)
Still breastfeeding	71,397(40.74)	18,261(25.58)
**Perceived size at birth**		
Large	53,478(32.87)	15,044(28.13)
Average	81,999(50.40)	25,391(30.97)
Small	27,219(16.73)	10,537(38.71)
**Mothers' educational status**		
No education	71,108(37.04)	25,867(36.38)
Primary	61,015(31.79)	20,175(33.07)
Secondary	51,005(26.57)	11,385(22.32)
Higher	8,824(4.60)	818(9.28)
**Mother currently working**		

Yes	109,618(77,227)	33,980(31.00)
No	77,227(41.33)	22,379(28.98)
**Postnatal care visits**		
Yes	44,774(34.71)	11,787(26.33)
No	84,227(65.29)	25,394(30.15)
**Mother's age (years)**		
15-24	52,209(27.20)	16,514(31.63)
25-49	139,744(72.80)	41,732(29.86)
**Number of ANC visits**		
No visit	12,189(6.35)	4,919(40.36)
1 to 3 visits	39,646(20.65)	12,762(32.19)
4 and above visit	140,117(73.00)	40,565(28.95)
**Place of delivery**		
Home	56,237(31.51)	16,649(38.46)
Health facility	122,213(69.64)	38,187(61.54)
**Marital status**		
Single	42,811(22.30)	12,499(29.20)
Married	149,142(77.70)	45,747(30.67)
**Mothers age at 1st birth**		
Less than 20	110,643(57.64)	36,334(32.84)
20 and above	81,309(42.36)	21,912(26.95)
**Maternal nutritional status**		
Underweight	12,591(6.56)	5,131(40.76)
Normal weight	91,612(47.73)	31,342(34.21)
Overweight	26,149(13.62)	6,036(23.08)
Obese	61,600(32.09)	15,736(25.55)
**Household size**		
Small	16,665(8.68)	4,882(29.30)
Medium	12,723(6.63)	3,719(29.23)
Large	162,563(84.69)	49,644(30.54)
**Mass media exposure**		
No	65,415(34.08)	25,077(38.34)
Yes	126,538(65.92)	33,169(26.21)

**Wealth index**		
Poor	84,032(43.78)	25,499(53.29)
Middle	38,451(20.03)	11,668(20.53)
Rich	69,471(36.19)	21,080(28.18)
**Place of residence**		
Urban	65,983(34.37)	14,229(21.57)
Rural	125,969(65.63)	44,017(34.94)
**Subregion**		
West African Countries	81,068(42.23)	22,671(27.97)
East African Countries	47,706(24.85)	14,833(31.09)
Central African Countries	37,024(19.29)	12,289(33.19)
Southern African Countries	26,154(13.63)	8,452(26,154)

Pooled prevalence of stunting among children under 5 years of age in sub–Saharan African

The pooled prevalence of stunting among children under 5 years of age in 35 sub-Saharan African (SSA) countries was 29.89% (95% CI: 26.63, 33.14%), with significant variation observed across countries ($I^2 = 99.6$, P-value = 0.000) (Fig 2). Gabon had the lowest stunting prevalence at 13.91%, while Burundi reported the highest at 55.80%. Among the 35 countries analyzed, 15 had a stunting prevalence of 30% or higher among children under 5 years of age.

Handling heterogeneity

The random-effects model revealed considerable heterogeneity. To address this, sensitivity analysis, subgroup analysis, and meta-regression were conducted.

Sensitivity analysis

Sensitivity analysis was performed to evaluate the effect of individual country on the pooled estimated. When individual country was omitted, the pooled prevalence obtained was within the 95% CI of the overall pooled prevalence. This confirms the absence of single study impact on the overall pooled effect size. Therefore, from the random effects model, there were no country that excessively influence the overall pooled estimate of stunting (Fig 3).

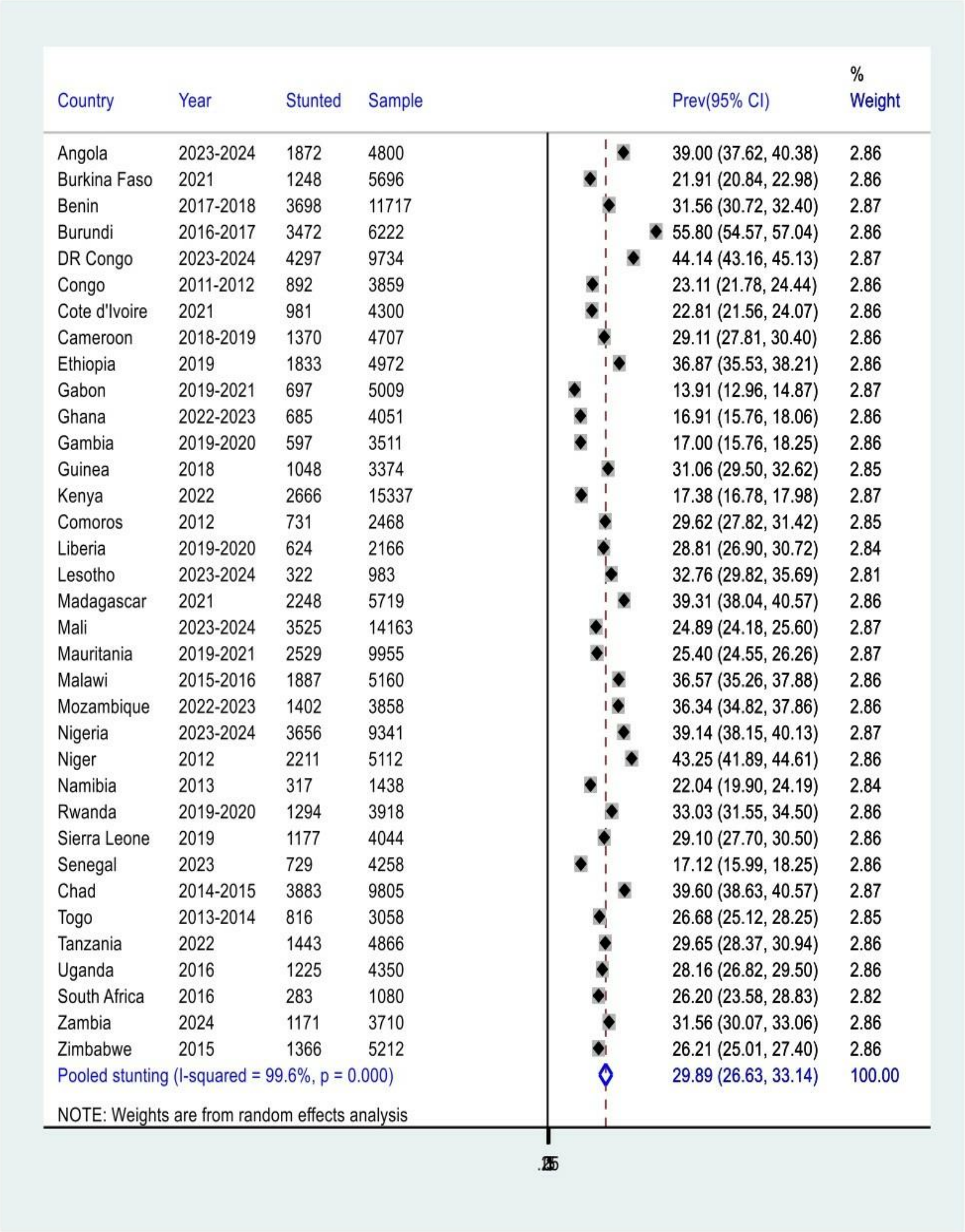

Country	Year	Stunted	Sample	Prev(95% CI)	% Weight
Angola	2023-2024	1872	4800	39.00 (37.62, 40.38)	2.86
Burkina Faso	2021	1248	5696	21.91 (20.84, 22.98)	2.86
Benin	2017-2018	3698	11717	31.56 (30.72, 32.40)	2.87
Burundi	2016-2017	3472	6222	55.80 (54.57, 57.04)	2.86
DR Congo	2023-2024	4297	9734	44.14 (43.16, 45.13)	2.87
Congo	2011-2012	892	3859	23.11 (21.78, 24.44)	2.86
Cote d'Ivoire	2021	981	4300	22.81 (21.56, 24.07)	2.86
Cameroon	2018-2019	1370	4707	29.11 (27.81, 30.40)	2.86
Ethiopia	2019	1833	4972	36.87 (35.53, 38.21)	2.86
Gabon	2019-2021	697	5009	13.91 (12.96, 14.87)	2.87
Ghana	2022-2023	685	4051	16.91 (15.76, 18.06)	2.86
Gambia	2019-2020	597	3511	17.00 (15.76, 18.25)	2.86
Guinea	2018	1048	3374	31.06 (29.50, 32.62)	2.85
Kenya	2022	2666	15337	17.38 (16.78, 17.98)	2.87
Comoros	2012	731	2468	29.62 (27.82, 31.42)	2.85
Liberia	2019-2020	624	2166	28.81 (26.90, 30.72)	2.84
Lesotho	2023-2024	322	983	32.76 (29.82, 35.69)	2.81
Madagascar	2021	2248	5719	39.31 (38.04, 40.57)	2.86
Mali	2023-2024	3525	14163	24.89 (24.18, 25.60)	2.87
Mauritania	2019-2021	2529	9955	25.40 (24.55, 26.26)	2.87
Malawi	2015-2016	1887	5160	36.57 (35.26, 37.88)	2.86
Mozambique	2022-2023	1402	3858	36.34 (34.82, 37.86)	2.86
Nigeria	2023-2024	3656	9341	39.14 (38.15, 40.13)	2.87
Niger	2012	2211	5112	43.25 (41.89, 44.61)	2.86
Namibia	2013	317	1438	22.04 (19.90, 24.19)	2.84
Rwanda	2019-2020	1294	3918	33.03 (31.55, 34.50)	2.86
Sierra Leone	2019	1177	4044	29.10 (27.70, 30.50)	2.86
Senegal	2023	729	4258	17.12 (15.99, 18.25)	2.86
Chad	2014-2015	3883	9805	39.60 (38.63, 40.57)	2.87
Togo	2013-2014	816	3058	26.68 (25.12, 28.25)	2.85
Tanzania	2022	1443	4866	29.65 (28.37, 30.94)	2.86
Uganda	2016	1225	4350	28.16 (26.82, 29.50)	2.86
South Africa	2016	283	1080	26.20 (23.58, 28.83)	2.82
Zambia	2024	1171	3710	31.56 (30.07, 33.06)	2.86
Zimbabwe	2015	1366	5212	26.21 (25.01, 27.40)	2.86
Pooled stunting (I-squared = 99.6%, p = 0.000)				29.89 (26.63, 33.14)	100.00

NOTE: Weights are from random effects analysis

Figure 2: Forest plot of the country-level and pooled prevalence of stunting among children under 5 years of age across 35 SSA countries, 2011–2024

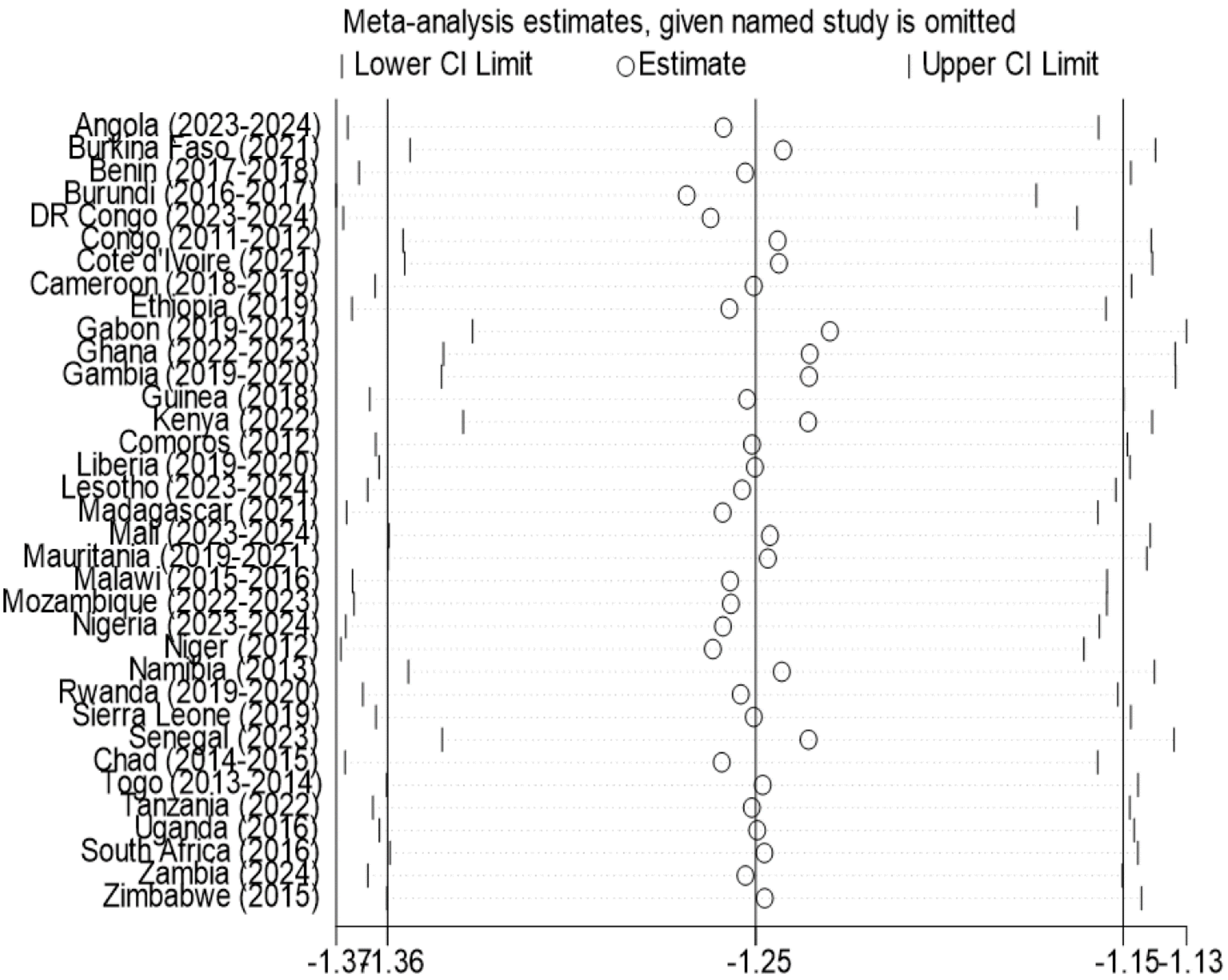

Figure 3: Sensitivity analysis plot forest plot of the country-level and pooled prevalence of stunting among children under 5 years of age across 35 SSA countries, 2011–2024

Sub-group analysis

Subgroup analyses were performed carried out by sub-region, and year of publication.

Stunting by Sub-Region

Subgroup analysis by sub-region indicated that Est African countries had the highest pooled prevalence of stunting among children under 5 years of age (33.73%, 95% CI: 24.19–43.26), followed by Southern African countries (31.37%, 95% CI: 27.20–35.54), Central African countries (29.98%, 95% CI: 18.24–41.71) and West African countries (26.83%, 95% CI: 22.89–30.77). Substantial heterogeneity was observed in East and Central Africa (I^2 = 99.8%, P = 0.00), West Africa (I^2 = 99.4%, P = 0.00), and Southern Africa (I^2 = 98.1%, P = 0.00) (Fig 4).

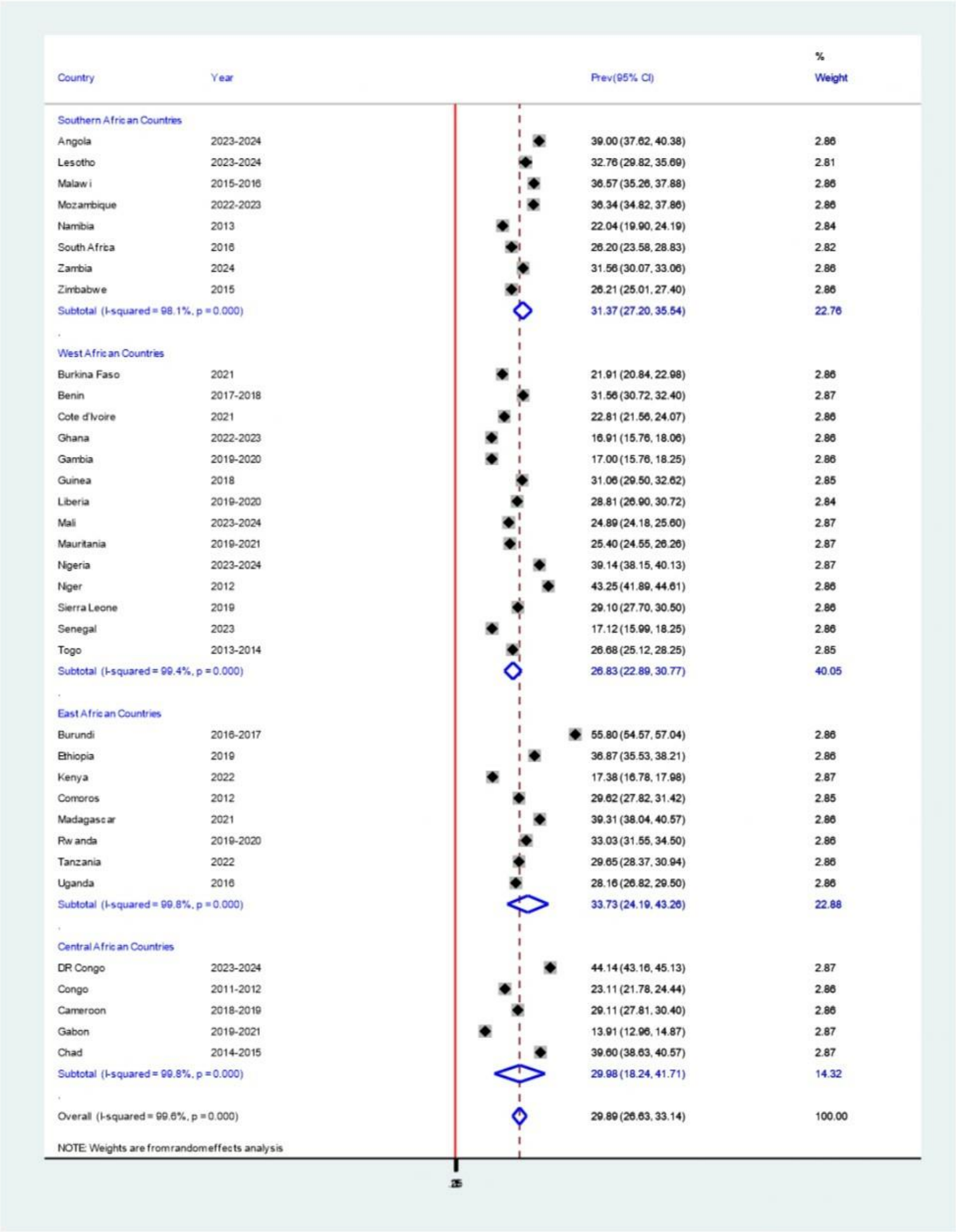

Figure 4: Forest plot showing the pooled prevalence of stunting among children under 5 years of age in sub-Saharan Africa, by subregion, based on a random-effects model analysis, 2011-2024.

Stunting by Year of Survey

Subgroup analysis by year of survey showed that the pooled prevalence of stunting was highest in 2011–2018 (32.32%, 95% CI: 27.19–37.44) and lowest in 2019–2024 (28.45%, 95% CI: 24.57–32.33). Both periods demonstrated significant heterogeneity (2011–2018: $I^2 = 99.5\%$, $p < 0.00$; 2020–2024: $I^2 = 99.6\%$, $p < 0.00$) (Fig 5).

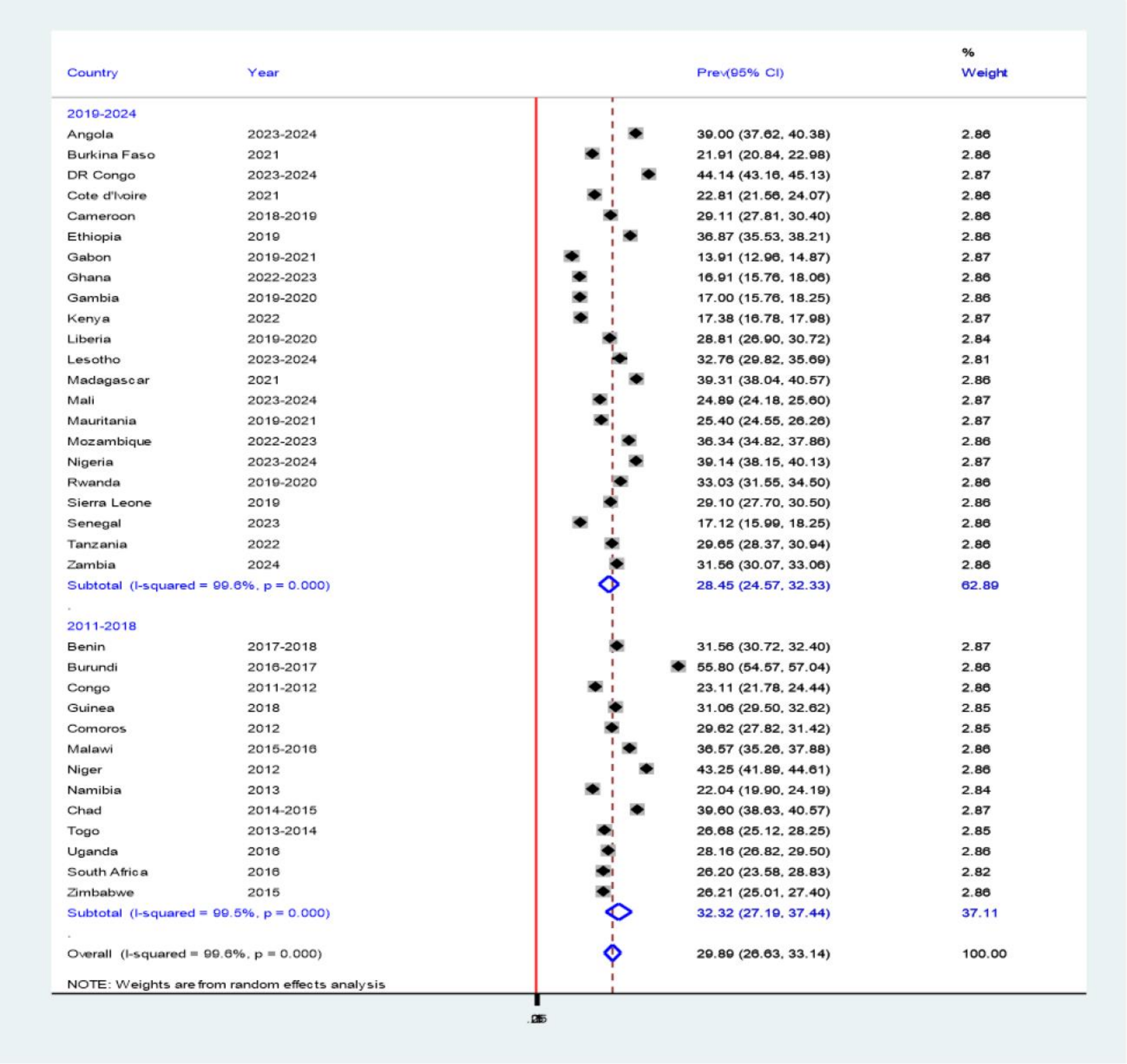

Figure 5: Forest plot showing the pooled prevalence of stunting among children under 5 years of age in sub-Saharan Africa, by survey year category, based on a random-effects model analysis, 2011-2024.

Meta-regression

A meta-regression was performed to assess whether the year of survey, sample size, country, or sub-region could explain the heterogeneity in stunting prevalence (Table 3). None of these variables were statistically significant predictors of heterogeneity (all p-values > 0.05). The high residual heterogeneity in the pooled prevalence of stunting suggests that other unmeasured factors are responsible for the variability across studies

Table 3: Meta-regression for the studies of pooled stunting among children under 5 years of age in 35 sub-Sharan Africa, 2011-2024.

Outcome categories	Variables	Coefficients	P-value	[95% conf. interval]
Pooled stunting among under five children	Year of Survey	0.99	0.68	0.96, 1.02
	Sample size	1.00	0.65	0.99, 1.00
	Countries	0.99	0.22	0.98, 1.00
	Sub-region	1.06	0.19	0.96, 1.17

Results of Random-Effects Analysis (Measures of Variation)

The ICC (Intraclass Correlation Coefficient) value from the null model revealed that only about 26.8% of the total variance in stunting among under five children is attributable to differences between clusters. The remaining 73.2% of the variance reflects variation occurring within clusters, which encompasses individual, household, and broader contextual factors. This indicates that while cluster-level differences play a substantial role, the majority of the variability in stunting arises from determinants operating at multiple levels within clusters. In the final model (Model IV), the explained variance values demonstrated that around 47.2% of the total variation in the pooled prevalence of stunting among children under 5 years of age was influenced by the combined effects of individual level factors, household level factors and contextual factors-level factors.

Additionally, the presence of heterogeneity in stunting across clusters was supported by the MOR (Median Odds Ratio) values of 1.96 and 1.46 in the null and full models, respectively. This suggests that children under 5 years of age in clusters with higher pooled prevalence of stunting had approximately 1.96 times greater odds of stunting compared to those in clusters with lower levels of pooled prevalence of stunting. Model 4 was identified as the best-fitted model, as it exhibited the lowest AIC (Akaike Information Criterion), BIC (Bayesian Information Criterion), and deviance values (Table 4).

Table 4: Selection of most parsimonious model (measures of variation) at cluster or community level for stunting among children under 5 years of age in 35 SSA countries, 2011–2024

**Measure of variation**	**Models**				
	**Null-Model**	**Model I**	**Model II**	**Model III**	**Model IV**
Cluster-level variance (SE)	0.5(0.05)	0.15(0.001)	0.2(0.03)	0.3(0.04)	0.16(0.025)
Intra-class correlation (%) or VPC	26.8%	13.8%	12.7%	11.4%	10.2%
Explained variance (%) or PCV	***Reference***	70%	60%	40%	47.2%
Median odds ratio	1.96	1.44	1.5	1.68	1.46
Model summary					
Akaike's information criteria	245122.6	138191.7	169678.7	241377.5	**98254.67**
Bayesian information criteria	245153.1	138453.6	169807	241448.8	**98639.09**
Log-likelihood	-122558.28	-69068.872	-84826.365	-120681.75	**-49086.333**
Deviance	245116.55	138137.74	169652.73	241363.5	**98172.666**

VPC *= Variance Partitioning Coefficient,* ***PCV*** *= Proportion Change in Variance*

Determinants of stunting among children under 5 years of age in 35 sub–Saharan African Countries

Our analysis showed that the prevalence of stunting was 1.24 times higher among male children compared to female children (aPR = 1.24, 95% CI: 1.21–1.26). Using children under 12 months of age as the reference category, the prevalence of stunting was 2.07 times higher among those aged 12–23 months (aPR = 2.07, 95% CI: 2.00–2.14). It was 2.41 times higher among children aged 24–35 months (aPR = 2.41, 95% CI: 2.32–2.50), 2.32 times higher among those aged 36–47 months (aPR = 2.32, 95% CI: 2.23–2.42), and 1.81 times higher among children aged 48–59 months (aPR = 1.81, 95% CI: 1.71–1.90) (Table 4).

Children born in the fifth or higher birth order had an 8% higher prevalence of stunting compared to those born as first-order children (aPR = 1.08, 95% CI: 1.03–1.12). Using children perceived as large at birth as the reference category, the prevalence of stunting was 16% higher among those perceived as average size at birth (aPR = 1.16, 95% CI: 1.13–1.19) and 48% higher among those perceived as small size at birth (aPR = 1.48, 95% CI: 1.43–1.52). Compared to children whose mothers had a higher level of formal education, the prevalence of stunting was 1.94 times higher among children whose mothers had no formal education (aPR = 1.94, 95% CI: 1.74–2.18), 1.95 times higher among those whose mothers had only primary education (aPR = 1.95, 95% CI: 1.74–2.19), and 1.68 times higher among those whose mothers had secondary education (aPR = 1.68, 95% CI: 1.51–1.88).

This study revealed that children whose mothers did not attend postnatal visits had a 3% higher prevalence of stunting compared to those whose mothers attended such visits (aPR = 1.03, 95% CI: 1.01–1.05). Using mothers aged 25 years and above as the reference category, the prevalence of stunting was 9% higher among children of mothers aged 15–24 years (aPR = 1.09, 95% CI: 1.06–1.13). Compared to children whose mothers had four or more antenatal care (ANC) visits, the prevalence of stunting was 17% higher among those whose mothers had no ANC visits (aPR = 1.17, 95% CI: 1.13–1.21) and 11% higher among those whose mothers had only one to three visits (aPR = 1.11, 95% CI: 1.09–1.14). In addition, children delivered at home had an 11% higher prevalence of stunting compared to those delivered in health facilities (aPR = 1.11, 95% CI: 1.08–1.14). Furthermore, compared to children of married mothers, the prevalence of stunting was 1.04 times higher among children of single mothers (aPR = 1.04, 95% CI: 1.02–1.07).

This study revealed that maternal BMI was significantly associated with childhood stunting. Compared to children of mothers with a normal BMI, the prevalence of stunting was 12% higher among children of underweight mothers (aPR = 1.12, 95% CI: 1.08–1.16). In contrast, the prevalence of stunting was 19% lower among children of overweight mothers compared to those of mothers with a normal BMI (aPR = 0.81, 95% CI: 0.77–0.84). Similarly, the prevalence of stunting was 12% lower among children of obese mothers compared to those of mothers with a normal BMI (aPR = 0.88, 95% CI: 0.85–0.90).

This study revealed that household size was significantly associated with childhood stunting. Compared to children from smaller households, the prevalence of stunting was 4% higher among children from large households (aPR = 1.04, 95% CI: 1.04–1.08). In addition, children whose mothers reported no exposure to mass media such as television, radio, or newspapers had an 11% higher prevalence of stunting compared to children whose mothers had some media exposure (aPR = 1.11, 95% CI: 1.08–1.13).

Moreover, compared to children from rich households, the prevalence of stunting was 31% higher among children from poor households (aPR = 1.31, 95% CI: 1.24–1.38). Similarly, the prevalence of stunting was 23% higher among children from middle wealth index households compared to those from rich households (aPR = 1.23, 95% CI: 1.17–1.29). Furthermore, children living in rural areas had a 7% higher prevalence of stunting compared to their urban counterparts (aPR = 1.07, 95% CI: 1.04–1.11).

Table 4: Bivariable and multivariable multilevel mixed-effects robust Poisson regression analysis of determinants of stunting among children under 5 years of age in 35 SSA countries, 2011–2024

Covariates	Stunting		cPR with 95%	aPR with 95%
	Yes	No		
**Sex of the child**				
Male	31,604(32.61)	65,303(67.39)	1.16(1.14, 1.18) *	***1.24(1.21, 1.26) *****
Female	26,642(28.03)	68,402(71.97)	1	1
**Age of the child (months)**				
Less than 12 months	7,156(16.96)	35,029(83.04)	1	1
12–23 months	13,492(33.81)	26,415(66.19)	1.99(1.93, 2.06) *	***2.07(2.00, 2.14) *****
24–35 months	14,225(38.49)	22,736(61.51)	2.26(2.19, 2.34) *	***2.41(2.32, 2.50) *****
36–47 months	13,108(34.76)	24,599(65.24)	2.05(1.98, 2.12) *	***2.32(2.23, 2.42) *****
48–59 months	10,264(29.17)	24,926(70.83)	1.72(1.66, 1.78) *	***1.81(1.71, 1.90) *****
**Birth order**				
First	11,640(27.88)	30,115(72.12)	1	1
2 to 4	27,615(29.12)	67,224(70.88)	1.04(1.01, 1.06) *	1.01(0.98, 1.05)
5 or higher	18,991(34.31)	36,367(65.69)	1.21(1.18, 1.24) *	***1.08(1.03, 1.12) *****
**Perceived size at birth**				
Large	15,044(28.13)	38,434(71.87)	1	1
Average	25,391(30.97)	56,608(69.03)	1.10(1.07, 1.12) *	***1.16(1.13, 1.19) *****
Small	10,537(38.71)	16,682(61.29)	1.37(1.34, 1.41) *	***1.48(1.43, 1.52) *****
**Mothers' educational status**				
No education	25,867(36.38)	45,241(63.62)	3.84(3.49, 4.22) *	***1.94(1.74, 2.18) *****
Primary	20,175(33.07)	40,839(66.93)	3.49(3.17, 3.84) *	***1.95(1.74, 2.19) *****
Secondary	11,385(22.32)	39,619(77.68)	2.37(2.15, 2.61) *	***1.68(1.51, 1.88) *****
Higher	818(9.28)	8,006(90.72)	1	1
**Postnatal care visits**				
Yes	11,787(26.33)	32,986(73.67)	1	1
No	25,394(30.15)	58,832(69.85)	1.14(1.11, 1.17) *	***1.03(1.07, 1.05) *****
**Mother's age (years)**				
15-24	16,514(31.63)	35,694(68.37)	1.05(1.03, 1.07) *	***1.09(1.06, 1.13) *****
25-49	41,732(29.86)	98,012(70.14)	1	1
**Number of ANC visits**				
No visit	4,919(40.36)	7,269(59.64)	1.36(1.32, 1.41) *	***1.17(1.13, 1.21) *****
1 to 3 visits	12,762(32.19)	26,884(67.81)	1.10(1.08, 1.12) *	***1.11(1.09, 1.14) *****
4 and above visit	40,565(28.95)	99,552(71.05)	1	1

**Place of delivery**				
Home	16,649(38.46)	32,199(26.68)	1.44(1.41, 1.47) *	***1.11(1.08, 1.14) *****
Health facility	38,186(61.54)	88,466(73.32)	1	1
**Marital status**				
Single	12,499(29.20)	30,311(70.80)	0.94(0.92, 0.96) *	***1.04(1.02, 1.07) *****
Married	45,747(30.67)	103,39(69.33)	1	1
**Mothers age at 1st birth**				
Less than 20	36,334(32.84)	74,309(67.16)	1.20(1.18, 1.22) *	1(0.97, 1.02)
20 and above	21,912(26.95)	59,397(73.05)	1	1
**Maternal nutritional status**				
Underweight	5,131(40.76)	7,459(59.24)	1.19(1.15, 1.22) *	***1.12(1.08, 1.16) *****
Normal weight	31,342(34.21)	60,270(65.79)	1	1
Overweight	6,036(23.08)	20,113(76.92)	0.68(0.66 0.70) *	***0.81(0.77, 0.84) *****
Obese	15,736(25.55)	45,863(74.45)	0.75(0.73, 0.77) *	***0.88(0.85, 0.90) *****
**Household size**				
Small	4,882(29.30)	11,782(70.70)	1	1
Medium	3,719(29.23)	9,004(70.77)	1(0.95, 1.04)	0.99(0.94, 1.05)
Large	49,644(30.54)	112,919(69.46)	1.04(1.01, 1.07) *	***1.04(1.04, 1.08) *****
**Mass media exposure**				
No	25,077(38.34)	40,337(61.66)	1.44(1.41, 1.47) *	***1.11(1.08, 1.13) *****
Yes	33,169(26.21)	93,368(73.79)	1	1
**Wealth index**				
Poor	16,637(38.34)	52,993(39.63)	2.26(2.17, 2.36) *	***1.31(1.24, 1.38) *****
Middle	11,958(31.10)	26,492(19.81)	1.83(1.75, 1.91) *	***1.23(1.17, 1.29) *****
Richer	9,793(26.42)	54,221(40.55)	***1***	***1***
**Place of residence**				
Urban	14,229(21.57)	51,754(78.43)	1	1
Rural	44,017(34.94)	81,952(65.06)	1.61(1.56, 1.66) *	***1.07(1.04, 1.11) *****

cPR*: Crude Prevalence Ratio,* ***aPR****: Adjusted Prevalence Ratio, *: Significant in crude prevalence ratio, **: Significant in adjusted prevalence ratio*

Discussion

Reducing stunting in sub-Saharan Africa (SSA) continues to pose a significant challenge, yet various programmatic initiatives are being introduced to drive progress. The World Bank's "All Hands-on Deck" initiative advocates for a multisectoral strategy, involving sectors such as agriculture, education, social protection, and water, sanitation, and hygiene (WASH)(42).

Similarly, UNICEF's programs prioritize maternal, infant, and young child nutrition, micronutrient supplementation, the management of severe acute malnutrition, nutrition in emergencies, and enhancing water and sanitation at the community level(43). Despite these efforts, the burden of stunting remains high in sub-Saharan African countries. These integrated approaches are specifically designed to accelerate stunting reduction in SSA, with a particular emphasis on East Africa, where the challenge persists(4).

This study analyzed pooled data from nationally representative surveys conducted across 35 sub-Saharan African (SSA) countries between 2011 and 2024. The findings revealed that the overall pooled prevalence of stunting among children under 5 years of age in SSA was 29.89% (95% CI: 26.63, 33.14%). The lowest stunting prevalence rate was recorded in Gabon at 13.74%, while the highest prevalence of stunting was observed in Burundi at 55.74%. Among the 35 countries analyzed, 14 had a stunting prevalence of 30% or higher among children under 5 years of age. This finding is consistent with World Health Organization prevalence threshold or cut-off values for public health significance in which prevalence of stunting 20 to <30% is high and greater than or equal to 30% is very high(44). Additionally, the pooled prevalence of stunting aligns with findings from studies carried out in 65 Low- and Middle-Income Countries(29.0%)(45), 62 LMICs worldwide(29.1%)(46), Tajikistan (27%)(47), and Tanzania (28%)(48).

However, the observed prevalence is lower than that reported in previous studies conducted in Ethiopia (33.58%)(49), Low- and Middle-Income Countries(38.8%)(50), East Africa(33.3%, 39%)(51, 52), Sub-Saharan Africa(33.2%)(52), Pakistan(44%)(53), Ethiopia(37%)(54), Kenya (39%)(55), Sub-Saharan Africa(35%)(4), Tanzania(35%)(56), Vietnam (44%)(57), Chitwan (37.3%)(58), and Belahara VDC (37%)(59),. Additionally, this finding was higher than the prevalence of stunting reported in studies conducted in China (4.4%)(60), Bangladesh (25%)(61), Bhutan(21%)(62), and UNICEF-WHO-WB Joint Child Malnutrition Estimates(22.3%)(63).

A potential explanation for this finding could be the differences in nutritional practices, sociocultural and environmental factors across the countries. Variations in the scope of the studies, the year of research, population characteristics, and methodologies employed might have contributed to the observed differences. For instance, earlier studies were limited in scope, focusing on individual countries and utilizing diverse analytical approaches(4, 64-68) and some

studies also included a restricted number of countries and population characteristics by ages (4). Moreover, another main reason could be attributed to the prolonged malnutrition among children under 5 years of age in Sub-Saharan Africa (SSA), stemming from persistent poverty in the region(69). Furthermore, the limited economic resources of many African nations can hinder access to sufficient and nutritious food, contributing to the prevalence of stunting(70).

Although the pooled prevalence of stunting among children under 5 years of age in 35 SSA countries was estimated at 29.89% (95% CI: 26.63, 33.14%), the exceptional high heterogeneity ($I^2 = 99.6\%$, $p < 0.000$) underscores substantial contextual variation across countries. This variation is not statistical only but also reflects meaningful differences in underlying determinants, health system capacity, and sociopolitical environments. Subgroup analysis revealed that East African countries had highest stunting burden (33.73%), while West African showed comparatively lower prevalence (26.83%), suggesting region drivers such as climate vulnerability, food system resilience, or maternal education levels.

However, meta-regression indicated that broad variables like survey year, sample size, country, and sub-region did not significantly explain this heterogeneity (all p-values > 0.05), pointing to the influence of more unmeasured factors such as governance quality, conflict exposure, or local nutrition policies. These findings highlight the limitations of the relying solely on pooled estimate for policy guidance and emphasizes the need for country level diagnostics to identify context specific risk factors. Future research should explore these modifiers systematically to inform tailored interventions rather than one size fits all strategies.

Our findings revealed that stunting among children under 5 years of age in sub-Saharan African countries is shaped by a mix of individual, household-level, and contextual factors. One significant predictor was the sex of the child, with male children more likely to experience stunting than females. This finding aligns with a study conducted in Sub-Saharan African (SSA) countries, as well as studies conducted in Zambia and Ethiopia(4, 67, 71, 72). The consistency of this association across diverse setting suggests a robust pattern that may reflect biological vulnerability, differential care practices, or gendered exposure to environmental risks. Evidence from longitudinal studies in sub-Saharan Africa indicates that male children are more prone to growth faltering during the first year of life(73). However, findings from certain interventions program such as nutritional

supplementation and maternal education initiatives suggest that these sex-based disparities in stunting may diminish over time(74).

This indicates that while male children may be biologically predisposed to stunting, contextual factors such as household factors, caregiving practices, and health system responsiveness can modify these risks. What remain uncertain is whether the sex differential is primarily driven by biological susceptibility or by modifiable social determinants. Future research, particularly longitudinal interventions studies, is needed to clarify and separate the overlapping biological and socio-behavioral pathways, in order to determine whether targeted strategies for boys could achieve greater reductions in stunting prevalence.

The prevalence of stunting was significantly higher among older children in the age groups of 12–23 months, 24–35 months, 36–47 months, and 48–59 months compared to children under 12 months of age. This finding indicates that the risk of stunting rises as a child grows older. Similar age-related patterns have been reported in studies conducted in Ethiopia(49, 54, 67, 75), Somalia(76), Rwanda(77), and Zimbabwe(78), suggesting that growth faltering often becomes more pronounced after infancy.

A prospective birth cohort studies have demonstrated that growth deficit often accelerates between 6 and 24 months, reflecting both biological vulnerability and the transition to inadequate diets and increased exposure to infections(79, 80). Interventions trial have further shown that timely interventions during this critical window period can reduce stunting prevalence(81). The novel contribution of this study is the documentation of this established association within our specific, previously less investigated population, thereby strengthening the geographical evidence base. What remain uncertain, however is, the extent to which age-related differences are modifiable through targeted interventions and whether the persistency of stunting in to later childhood reflects irreversible early childhood damage or ongoing exposure to adverse conditions.

Children born in the fifth or higher birth order had an 8% higher prevalence of stunting compared to those born as first-order children. This finding aligns with earlier studies that have examined child nutritional status, considering birth order as a significant confounding factor(82-85). Cohort studies have shown that later born children often experience reduced access to household resources, shorter breastfeeding duration, and greater exposure to infections, which collectively

contribute to growth faltering(86, 87). Interventions study also demonstrated that empowering mothers to space births and manage household resources can mitigate the nutritional advantages faced by higher order children(88, 89). However, the extent to which birth order effects are biologically driven versus socially mediated, and whether targeted interventions such as birth spacing programs or nutrition support for larger family can fully offset these risks were remains uncertain.

The perceived size of a baby at birth showed a significant association with stunting status, with children born small and average size more likely to be stunted compared to those born large. This finding is consistent with studies conducted in Africa and other developing countries, such as Indonesia and Ethiopia(90-92) as well as Nepal(93), Bangladesh(94) and Ethiopia(95). Cohort studies conducted in Ethiopia and Malawi have shown that children born small remain at elevated risk of stunting well into the first two years of life, even after accounting for postnatal feeding practices(96). Another interventional study on maternal nutrition supplementation programs in Ghana and Tanzania, further confirmed that improving maternal diet and antenatal care can reduce the incidence of the low birth weight and subsequently lower stunting prevalence(97, 98). However, uncertainties remain regarding the extent to which postnatal interventions (e.g., complementary feeding programs) can fully offset the disadvantage of being born small, suggesting that prevention strategies must prioritize maternal health and antenatal care.

The study revealed that compared to children whose mothers had a higher level of formal education, the prevalence of stunting was 1.94 times higher among children whose mothers had no formal education, 1.95 times higher among those whose mothers had only primary education, and 1.68 times higher among those whose mothers had secondary education. This finding is supported by a systematic review of studies published worldwide between 2004 and 2014, as well as by research conducted in Ethiopia, Zambia and Nepal(4, 99-102). Previous intervention studies have established that maternal education as protective factors. For example, community based nutritional programs in Uganda and Tanzania show that incorporating maternal literacy and health educations components resulted in significantly lower stunting prevalence versus control group(103, 104). However, maternal nutritional counselling can only compensate for the disadvantage of low maternal education in resource constrained setting remain uncertain. Future

research should explore whether combining educational empowerment with direct nutrition interventions yields synergistic effects in reducing stunting.

Children born to mothers aged 15–24 were more likely to experience higher levels of stunting compared to those born to mothers aged 25-49. This finding consistent with study conducted in Ethiopia(105). This may be due to the fact that babies born to younger mothers are more prone to preterm birth and low birth weight, which can increase the risk of neonatal infections and malnutrition, including stunting(106). Another study also showed that younger maternal age is often associated with limited caregiving experience, reduced autonomy in household decision-making, and lower access to health and nutrition services contributing higher stunting prevalence(107). However further research needs maternal age itself act as independent risk factors versus being mediated by socioeconomic status, maternal education or health service utilization.

Mothers' antenatal care (ANC) visits were strongly associated with child stunting. Children whose mothers did not attend any antenatal follow-up and 1 to 3 ANC visits more likely to be stunted compared to those whose mothers had four or more ANC visits. This finding is supported by a study conducted in Ethiopia(108-110), Zambia(111), Latin America(112, 113), and Bangladesh(114). This may be attributed to the fact that antenatal care (ANC) programs are structured to identify high-risk mothers and deliver nutritional and educational support, such as advice on food hygiene, diet, and lifestyle. These initiatives target key factors that play a significant role in enhancing child nutrition. ANC programs are advocated as an effective approach to lowering the prevalence of low birth weight, and evidence highlighting their effectiveness in reducing adverse pregnancy outcomes in developing countries is steadily increasing(115, 116). However, barriers such as inequitable health system coverage, geographical isolation and weak governance in service delivery restrict women access to timely and quality antenatal care services(117, 118).

In this study, the place of delivery showed a significant association with child stunting. Specifically, children born at home were more likely to be stunted compared to those born at health facilities. This finding aligns with a study conducted in Ethiopia(4, 119). A possible explanation for this association is that mothers who deliver at home are less likely to receive proper postnatal care, including vaccinations, which are critical for the healthy growth and development of children.

Vaccinations help prevent several vaccine-preventable diseases and ensure children receive essential nutrients, contributing to better overall health outcomes(120).

The study revealed that postnatal care visits were associated with childhood stunting. Mothers who did not attend postnatal care services were 1.03 times more likely to have a stunted child compared to those who did. This finding is consistent with a study conducted in Ethiopia(121). Study confirmed that strengthening maternal contact with health systems during the postnatal period contributes to improved child growth outcomes(122). However, limited attendance at postnatal care is not solely a matter of maternal choice but reflects broader structural determinants. Barriers such as poverty, inequitable health system coverage, weak governance in service delivery and in some context conflict or geographical isolation restrict women access to timely and quality postnatal services(117, 118).

Addressing stunting therefore requires structural interventions such as equitable health infrastructure, improving governance and accountability in maternal and child health programs, and strengthening social protection alongside efforts to promote household-level health-seeking behaviors. Additionally, the extent to which postnatal care independently reduces stunting versus acting synergistically with other maternal and child health interventions such as antenatal care, breastfeeding promotion, and community nutrition programs remains uncertain.

Another factor associated with stunting was marital status. In the study, the prevalence of child stunting was higher among children of single mothers than among children with married mothers. This finding consistent with previous study(123). The possible explanation may be attributed to socioeconomic challenges, such as limited financial resources, reduced access to healthcare, and lower social support. Single mothers often face greater difficulties in providing adequate nutrition and care for their children due to these constraints. Additionally, the stress and time demands associated with single parenthood may further hinder their ability to ensure optimal child-rearing conditions, contributing to poorer growth outcomes. These factors collectively increase the risk of stunting in children of single mothers(124).

This study found that maternal body mass index was associated with childhood stunting. Compared to children whose mothers had a normal body mass index (BMI), the prevalence of stunting was higher among children of underweight mothers and lower among those of overweight

mothers, and obese mothers. These findings were consistent with previous study conducted in low-income and middle-income countries(125), Ethiopia(126, 127), Bangladesh(128) and Nigeria(6). While poor maternal nutrition likely contributes to inadequate nutrient transfer during pregnancy and lactation, resulting in impaired child growth, the relationship between maternal BMI and stunting extends beyond individual level behaviors. Lower stunting rates among children of overweight or obese mothers may reflect not only higher caloric intake but also broader socioeconomic advantages, such as improved household food security, access to diverse diets, and greater resilience to shocks(129).

These maternal outcomes are shaped by structural determinants, including poverty, food system functioning, governance and conflict that influence both maternal nutrition and child health. For instance, household insecure regions or conflict affected setting may face systematic barriers to adequate nutrition regardless of maternal practices(129, 130). Thus, while balanced maternal nutrition remains critical, these findings underscore the needs to situate maternal and household factors within wider structural contexts. Addressing stunting requires not only promoting individual behavior change but also tackling systematic inequities through poverty reduction, strengthening food systems, improving governance and ensuring stability.

This study revealed that child stunting high among household with large family size. This finding similar with previous study conducted in sub-Saharan Africa(131), and Ethiopia(132, 133). This may be due to the fact that higher prevalence of child stunting in households with large family sizes may be due to resource dilution, where limited financial and food resources are spread thinly among many family members, reducing the quantity and quality of nutrition available for each child. Larger families may also face challenges in providing adequate healthcare and sanitation, further exacerbating the risk of stunting. Additionally, caregivers in large families may have less time and capacity to ensure proper feeding practices and childcare, contributing to poorer growth outcomes(134).

The results of this study also revealed that mothers' exposure to mass media was significantly associated with high prevalence of child stunting. Children whose mothers had not media exposure were more likely to be stunted compared to those whose mothers had exposure to media. This finding consistent with studies conducted in Pakistan, Bangladesh and Ethiopia(4, 135, 136). Media plays a vital role in promoting sociocultural and economic development, which can contribute to

improved nutritional outcomes(137). However, access to mass media is shaped by systemic issues such as poverty, inequitable information and communication infrastructures, governance of public health messaging, and in some contexts, conflict that limits dissemination of reliable information(138). These structural barriers constrain households' ability to access knowledge about nutrition, sanitation, and child health, thereby reinforcing cycles of stunting(139). Therefore, addressing these challenges require system level interventions.

Our study revealed a significant positive association between the household wealth index and child stunting. Compared to children from the richest household wealth index, child from the poor and middle households' wealth index had higher odds of being stunted. While these findings align with a systematic review and meta-analysis conducted in Africa between 2000 and 2013 and study conducted in Sub-Saharan Africa(4, 140), as well as studies from low-income countries such as Nepal, Zambia, and Nigeria(101, 141, 142), the implications extend beyond household-level economic status(143). Household wealth reflects broader structural determinants including entrenched poverty, inequitable food systems, weak governance, and in some settings, the destabilizing effects of conflict that constrain access to adequate nutrition, health services, and safe environments for children(144, 145). Thus, stunting should be understood not only as a consequence of limited household resources but also as a manifestation of systemic inequities.

Furthermore, contextual-level variables specifically, place of residence were significantly associated with child stunting. Children from rural areas had higher prevalence of being stunted compared to their urban counterparts. A finding supported by studies conducted in Pakistan and Ethiopia(146, 147). This may be attributed to the better nutritional status of urban children, which stems from improved maternal prenatal and postnatal care, higher-quality complementary feeding practices, and better immunization coverage. Additionally, urban children benefit from advantages such as family employment opportunities, stronger social and family networks, and greater access to healthcare and social services, all of which contribute to their improved nutritional status compared to rural children(148).

Strength and limitation of the study

This study utilized a recent, nationally representative dataset encompassing 191,953 children under five from 35 sub-Saharan African countries surveyed between 2011 and 2024. The large sample

size substantially enhanced the statistical power to detect true effect sizes and improved the generatability of the findings across diverse settings. Advanced statistical methods, including multilevel mixed-effects Poisson regression with robust variance, were employed to strengthen the analysis and account for the hierarchical nature of the DHS data. By pooling data across countries, the study estimated the prevalence of stunting and its determinants, revealing significant and concerning heterogeneity between countries that persisted even after subgroup and sensitivity analyses. Importantly, the inclusion of key maternal and child health variables such as antenatal care (ANC) and postnatal care (PNC) visits, and maternal body mass index (BMI) addressed gaps overlooked in previous studies(4), thereby providing a more comprehensive understanding of stunting determinants.

Nonetheless, several limitations should be acknowledged. Health system variables, including health insurance coverage, the availability and type of health facilities, and broader country-level contextual factors such as political stability, were not assessed. Furthermore, the cross-sectional design of the DHS surveys precludes establishing causal relationships between independent and dependent variables. While significant associations were identified, determinants such as maternal nutritional status and household media exposure are likely influenced by broader socioeconomic conditions that also directly affect child stunting. Maternal BMI, for example, may shape child nutrition but is itself conditioned by household food insecurity and poverty, while media exposure may influence health behaviors yet remains dependent on socioeconomic access. Without longitudinal data, the temporal of these relationships remains uncertain. Therefore, the findings should be interpreted as highlighting important populations-level correlates rather than confirming causal pathways. Future prospective cohort studies are warranted to disentangle these relationships and clarify the causal mechanisms underlying child stunting in SSA.

Conclusion

In conclusion, this study analyzed pooled data from 35 sub-Saharan African (SSA) countries and found an overall stunting prevalence of 29.72% among children under 5 years of age, with significant variations across countries, ranging from 13.74% in Gabon to 55.74% in Burundi. Fourteen countries had stunting rates exceeding 30%, indicating a very high public health concern as per WHO thresholds.

Our study identified individual, household, and contextual factors significantly linked to stunting in children under 5 years of age. At the individual level, key predictors included being male, increased age of child, higher birth order (fifth or more), small or medium birth size, insufficient antenatal care (ANC) visits (none or 1–3), lack of postnatal visits, lower maternal education (no formal, primary, or secondary education), home delivery, single marital status, and maternal underweight, all of which were associated with increased odds of stunting. In contrast, maternal overweight or obesity were linked to reduced odds of stunting. At the household level, factors such as lack of maternal media exposure (television, radio, or newspapers), large family size, and lower household wealth (poor, middle, rich) were associated with higher odds of stunting. Additionally, at the contextual level, being rural residence was a significant predictor of stunting.

To address persistently high prevalence of stunting in sub-Saharan Africa, interventions should be clearly prioritized by feasibility and timeline but framed within both household level and structural determinants. Immediate actions remain critical such as improving maternal and child healthcare access through increased antenatal and postnatal care visit, promoting facility-based deliveries, and enhancing maternal education to strengthen feeding practices and nutrition awareness. Expanding media outreach can also provide rapid gains in health and nutrition knowledge.

However, this effort must be complemented by long term structural and systems level investments that tackle the root cause of the stunting. Poverty alleviation, resilient food systems, and equitable access to nutritious diets are central to reducing vulnerability. Governance and political stability play a decisive role in ensuring effective implementation of nutrition policies while conflict and displacement exacerbate food insecurity and health service disruptions. Addressing gender inequality in child nutrition, improved access to clean water and sanitation, expanding clean cooking fuels, and bridging between urban-rural disparities through strengthened healthcare infrastructure and social protection programs are equality vital.

A multisectoral approach like integrating health, education, agriculture, food systems and WASH sectors is essential to ensure both short term impact and sustainable improvements in child growth and developments. Future research should incorporate health systems variable like facility availability and quality alongside country level factors like governance, policy coherence and conflict dynamic. Longitudinal design is needed to establish casual pathways and guide more effective policy strategies that balance individual behavior change with structural reforms.

Abbreviations and Acronyms

AIC: Akaike’s Information Criteria

APR: Adjusted Prevalence Ratio

BIC: Bayesian Information Criteria

CI: Confidence Interval

CPR: Crude Prevalence Ratio

DHS: Demographic and Health Survey

ICC: Intra Class Correlation Coefficient

IRB: Institutional Review Board

LL: Log-Likelihood

MOR: Median Odds Ratio

PCV: Proportional Change in Variance

SSA: Sub-Saharan Africa

Acknowledgements

The authors thank ICF International for granting access to the SSA DHS data set used in this study.

Authors' Contributions

A.H. conceptualization, formal analysis, investigation, methodology, writing original draft, review and editing. K.G. methodology, review and editing. K.U. methodology, software, review and editing. B.F. review and editing. E.E. methodology, software, review and editing. R.M. methodology, review and editing. T.D. methodology, software, review and editing. B.K. methodology, review and editing.

Funding

No specific fund was received for this work.

Data availability

The datasets analyzed during current study are publicly available from the Demographic and Health Survey (DHS) program, managed by ICF international. The data can be accessed upon reasonable request through the DHS program website at http://www.dhsprogram.com. Researcher must register and submit a data access request through the site. For inquires regarding data access, please contact the DHS Data Archivist at archive@dhsprogram.com. The data used in this study are cited as: ICF. Demographic and Health Surveys (various years). Funded by USAID. Rockville, Maryland. Available at: http://dhsprogram.com.

Declarations

Consent for publication

Not Applicable

Competing interests

The authors declare no competing interests.

References

1. De Onis M, Branca F. Childhood stunting: a global perspective. Maternal & child nutrition. 2016;12:12-26.
2. Callanan A. Food and Nutrition Handbook2015.
3. Bharali N, Singh KN, Mondal N. Composite Index of Anthropometric Failure (CIAF) among Sonowal Kachari tribal preschool children of flood effected region of Assam, India. Anthropological Review. 2019;82(2):163-76.
4. Takele BA, Gezie LD, Alamneh TS. Pooled prevalence of stunting and associated factors among children aged 6–59 months in Sub-Saharan Africa countries: A Bayesian multilevel approach. Plos one. 2022;17(10):e0275889.
5. FOOD R, AFFORDABLE HDM. Food security and nutrition in the World. 2022.
6. Lawal SA, Okunlola DA, Adegboye OA, Adedeji IA. Mother's education and nutritional status as correlates of child stunting, wasting, underweight, and overweight in Nigeria: Evidence from 2018 Demographic and Health Survey. Nutrition and Health. 2024;30(4):821-30.
7. UNICEF, UNICEF. WHO/World Bank Joint Child Malnutrition Estimates, 2021 Edition. Disponibil la: https://data unicef org/topic/nutrition/malnutrition. 2022.
8. Mbogori T, Kimmel K, Zhang M, Kandiah J, Wang Y. Nutrition transition and double burden of malnutrition in Africa: a case study of four selected countries with different social economic development. AIMS Public Health. 2020;7(3):425.
9. Deshpande A, Ramachandran R. Early childhood stunting and later life outcomes: A longitudinal analysis. Economics & Human Biology. 2022;44:101099.

10. Soliman A, De Sanctis V, Alaaraj N, Ahmed S, Alyafei F, Hamed N, et al. Early and long-term consequences of nutritional stunting: From childhood to adulthood. Acta Bio Medica: Atenei Parmensis. 2021;92(1):e2021168.
11. Ahmed KY, Agho KE, Page A, Arora A, Ogbo FA, Maternal G, et al. Mapping geographical differences and examining the determinants of childhood stunting in Ethiopia: a Bayesian geostatistical analysis. Nutrients. 2021;13(6):2104.
12. Quamme SH, Iversen PO. Prevalence of child stunting in Sub-Saharan Africa and its risk factors. Clinical Nutrition Open Science. 2022;42:49-61.
13. Dapi Nzefa L, Monebenimp F, Äng C. Undernutrition among children under five in the Bandja village of Cameroon, Africa. South African Journal of Clinical Nutrition. 2019;32(2):46-50.
14. Amare M, Benson T, Fadare O, Oyeyemi M. Study of the determinants of chronic malnutrition in Northern Nigeria: quantitative evidence from the Nigeria demographic and health surveys international food policy research institute (IFPRI) working paper 45 (September 2017). Food and nutrition bulletin. 2018;39(2):296-314.
15. Wolde T, Adeba E, Sufa A. Prevalence of chronic malnutrition (stunting) and determinant factors among children aged 0–23 months in Western Ethiopia: a cross-sectional study. Journal of Nutritional Disorders & Therapy. 2014;4(4):148-54.
16. Awoke A, Ayana M, Gualu T. Determinants of severe acute malnutrition among under five children in rural Enebsie Sarmidr District, East Gojjam Zone, North West Ethiopia, 2016. BMC nutrition. 2018;4(1):4.
17. Ahmed MM, Hokororo A, Kidenya BR, Kabyemera R, Kamugisha E. Prevalence of undernutrition and risk factors of severe undernutrition among children admitted to Bugando Medical Centre in Mwanza, Tanzania. BMC Nutrition. 2016;2(1):49.
18. Rose ES, Blevins M, González-Calvo L, Ndatimana E, Green AF, Lopez M, et al. Determinants of undernutrition among children aged 6 to 59 months in rural Zambézia Province, Mozambique: results of two population-based serial cross-sectional surveys. BMC nutrition. 2015;1(1):41.
19. Mukabutera A, Thomson DR, Hedt-Gauthier BL, Basinga P, Nyirazinyoye L, Murray M. Risk factors associated with underweight status in children under five: an analysis of the 2010 Rwanda Demographic Health Survey (RDHS). BMC Nutrition. 2016;2(1):40.
20. Tibilla M. The nutritional impact of the world food programmesupported supplementary feeding programme on children less than five years in rural tamale. Ghana. 2007.
21. Yalew B, Amsalu F, Bikes D. Prevalence and factors associated with stunting, underweight and wasting: a community based cross sectional study among children age 6-59 months at Lalibela Town, Northern Ethiopia. J Nutr Disorders Ther. 2014;4(147):2161-0509.1000147.
22. Amare ZY, Ahmed ME, Mehari AB. Determinants of nutritional status among children under age 5 in Ethiopia: further analysis of the 2016 Ethiopia demographic and health survey. Globalization and health. 2019;15:1-11.
23. Poda GG, Hsu C-Y, Chao JC-J. Factors associated with malnutrition among children< 5 years old in Burkina Faso: evidence from the Demographic and Health Surveys IV 2010. International Journal for Quality in Health Care. 2017;29(7):901-8.
24. Haile D, Azage M, Mola T, Rainey R. Exploring spatial variations and factors associated with childhood stunting in Ethiopia: spatial and multilevel analysis. BMC pediatrics. 2016;16(1):49.
25. Talukder A. Factors associated with malnutrition among under-five children: illustration using Bangladesh demographic and health survey, 2014 data. Children. 2017;4(10):88.
26. Skoufias E, Vinha K, Sato R. Reducing stunting through multisectoral efforts in sub-Saharan Africa. Journal of African Economies. 2021;30(4):324-48.
27. UNICEF. Nutrition Strategy 2020-2030: Accelerating Stunting Reduction in Sub Saharan Africa. New York: UNICEF. 2019. . 2019.
28. Desa U. Transforming our world: The 2030 agenda for sustainable development. 2016.

29. Stevens GA, Finucane MM, Paciorek CJ, Flaxman SR, White RA, Donner AJ, et al. Trends in mild, moderate, and severe stunting and underweight, and progress towards MDG 1 in 141 developing countries: a systematic analysis of population representative data. The lancet. 2012;380(9844):824-34.
30. Bursac Z, Gauss CH, Williams DK, Hosmer DW. Purposeful selection of variables in logistic regression. Source code for biology and medicine. 2008;3(1):17.
31. Hosmer Jr DW, Lemeshow S, Sturdivant RX. Applied logistic regression: John Wiley & Sons; 2013.
32. Croft TN, Marshall AM, Allen CK, Arnold F, Assaf S, Balian S. Guide to DHS statistics. Rockville: ICF. 2018;645:292-303.
33. USAID. Demographic and health survey(DHS) methodology. https://dhsprogram.com/methodology/Survey-Types/DHS-Methodology.cfm n.d. Available.
34. Rahman MS, Howlader T, Masud MS, Rahman ML. Association of low-birth weight with malnutrition in children under five years in Bangladesh: do mother's education, socio-economic status, and birth interval matter? PloS one. 2016;11(6):e0157814.
35. Ntenda PAM. Association of low birth weight with undernutrition in preschool-aged children in Malawi. Nutrition journal. 2019;18:1-15.
36. Aboagye RG, Seidu A-A, Ahinkorah BO, Arthur-Holmes F, Cadri A, Dadzie LK, et al. Dietary diversity and undernutrition in children aged 6–23 months in Sub-Saharan Africa. Nutrients. 2021;13(10):3431.
37. WHO. WHO . Obesity and overweight. 2021. Available: https://www.who.int/news-room/fact-sheets/detail/obesity-and-overweight [Ref list]. 2021.
38. Weir CB, Jan A. BMI classification percentile and cut off points. 2019.
39. Cooper H, Hedges LV, Valentine JC. The handbook of research synthesis and meta-analysis: Russell Sage Foundation; 2019.
40. Bursac Z, Gauss CH, Williams DK, Hosmer DW. Purposeful selection of variables in logistic regression. Source code for biology and medicine. 2008;3:1-8.
41. Greene W. Functional forms for the negative binomial model for count data. Economics Letters. 2008;99(3):585-90.
42. Skoufias E, Vinha K, Sato R. All hands on deck: reducing stunting through multisectoral efforts in Sub-Saharan Africa: World Bank Publications; 2019.
43. (2019) U. UNICEF. (2019). Nutrition Strategy 2020-2030: Accelerating Stunting Reduction in Sub-Saharan Africa. New York: UNICEF. 2019.
44. de Onis M BE, Arimond M, Webb P, Croft T, Saha K et al. de Onis M, Borghi E, Arimond M, Webb P, Croft T, Saha K et al. Prevalence thresholds for wasting, overweight and stunting in children under 5 years. Public Health Nutrition. 2018;1-5. doi:10.1017/S1368980018002434. 2018.
45. Akombi BJ, Chitekwe S, Sahle BW, Renzaho AM. Estimating the double burden of malnutrition among 595,975 children in 65 low-and middle-income countries: a meta-analysis of demographic and health surveys. International journal of environmental research and public health. 2019;16(16):2886.
46. Ssentongo P, Ssentongo AE, Ba DM, Ericson JE, Na M, Gao X, et al. Global, regional and national epidemiology and prevalence of child stunting, wasting and underweight in low-and middle-income countries, 2006–2018. Scientific reports. 2021;11(1):5204.
47. (2016). U. The state of the world's children 2016 report. New York: UNICEF. 2016.
48. Kejo D, Mosha TC, Petrucka P, Martin H, Kimanya ME. Prevalence and predictors of undernutrition among underfive children in Arusha District, Tanzania. Food science & nutrition. 2018;6(8):2264-72.
49. Seifu BL, Tesema GA, Fentie BM, Yehuala TZ, Moloro AH, Mare KU. Geographical variation in hotspots of stunting among under-five children in Ethiopia: A geographically weighted regression and multilevel robust Poisson regression analysis. Plos one. 2024;19(5):e0303071.
50. Li Z, Kim R, Vollmer S, Subramanian S. Factors associated with child stunting, wasting, and underweight in 35 low-and middle-income countries. JAMA network open. 2020;3(4):e203386-e.

51. Tesema GA, Yeshaw Y, Worku MG, Tessema ZT, Teshale AB. Pooled prevalence and associated factors of chronic undernutrition among under-five children in East Africa: a multilevel analysis. PLoS One. 2021;16(3):e0248637.
52. Akombi BJ, Agho KE, Merom D, Renzaho AM, Hall JJ. Child malnutrition in sub-Saharan Africa: A meta-analysis of demographic and health surveys (2006-2016). PloS one. 2017;12(5):e0177338.
53. Khan S, Zaheer S, Safdar NF. Determinants of stunting, underweight and wasting among children< 5 years of age: evidence from 2012-2013 Pakistan demographic and health survey. BMC public health. 2019;19:1-15.
54. Raru TB, Ayana GM, Merga BT, Negash B, Deressa A, Birhanu A, et al. Magnitude of under-nutrition among under five children in Ethiopia based on 2019 Mini-Ethiopia Demographic and Health Survey: Generalized Linear Mixed Model (GLMM). BMC nutrition. 2022;8(1):113.
55. Badake Q, Maina I, Mboganie M, Muchemi G, Kihoro E, Chelimo E, et al. Nutritional status of children under five years and associated factors in Mbeere South District, Kenya. African Crop Science Journal. 2014;22:799-806.
56. Mamiro PS, Kolsteren P, Roberfroid D, Tatala S, Opsomer AS, Van Camp JH. Feeding practices and factors contributing to wasting, stunting, and iron-deficiency anaemia among 3-23-month old children in Kilosa district, rural Tanzania. Journal of Health, Population and Nutrition. 2005:222-30.
57. Hien NN, Kam S. Nutritional status and the characteristics related to malnutrition in children under five years of age in Nghean, Vietnam. Journal of Preventive Medicine and Public Health. 2008;41(4):232-40.
58. Ruwali D. Nutritional status of children under five years of age and factors associated in Padampur VDC, Chitwan. Health prospect. 2011;10:14-8.
59. Sapkota V, Gurung C. Community Medicine and Family Health Department, Maharajgunj Campus, Institute of Medicine, Tribhuwan University. Journal of Nepal Health Research Council. 2009;7(2):120-6.
60. Li H, Yuan S, Fang H, Huang G, Huang Q, Wang H, et al. Prevalence and associated factors for stunting, underweight and wasting among children under 6 years of age in rural Hunan Province, China: a community-based cross-sectional study. BMC Public Health. 2022;22(1):483.
61. Sultana P, Rahman MM, Akter J. Correlates of stunting among under-five children in Bangladesh: a multilevel approach. BMC nutrition. 2019;5:1-12.
62. Kang Y, Aguayo VM, Campbell RK, Dzed L, Joshi V, Waid JL, et al. Nutritional status and risk factors for stunting in preschool children in Bhutan. Maternal & child nutrition. 2018;14:e12653.
63. Estimates U-W-WJCM. Child malnutrition estimates for the indicators stunting, wasting, overweight and underweight describe the magnitude and patterns of under- and overnutrition. UNICEF-WHO-WB Joint Child Malnutrition Estimates inter-agency group updates regularly the global and regional estimates in prevalence and numbers for each indicator. 2022.
64. Aboagye RG, Ahinkorah BO, Seidu A-A, Frimpong JB, Archer AG, Adu C, et al. Birth weight and nutritional status of children under five in sub-Saharan Africa. Plos one. 2022;17(6):e0269279.
65. Haile D, Azage M, Mola T, Rainey R. Exploring spatial variations and factors associated with childhood stunting in Ethiopia: spatial and multilevel analysis. BMC pediatrics. 2016;16:1-14.
66. Hailu BA, Bogale GG, Beyene J. Spatial heterogeneity and factors influencing stunting and severe stunting among under-5 children in Ethiopia: spatial and multilevel analysis. Scientific reports. 2020;10(1):16427.
67. Muche A, Gezie LD, Baraki AG-e, Amsalu ET. Predictors of stunting among children age 6–59 months in Ethiopia using Bayesian multi-level analysis. Scientific Reports. 2021;11(1):3759.
68. Tamir TT, Techane MA, Dessie MT, Atalell KA. Applied nutritional investigation spatial variation and determinants of stunting among children aged less than 5 y in Ethiopia: A spatial and multilevel analysis of Ethiopian Demographic and Health Survey 2019. Nutrition. 2022;103:111786.

69. Harttgen K, Klasen S, Vollmer S. Economic growth and child undernutrition in sub-Saharan Africa. Population and development review. 2013;39(3):397-412.
70. Ahmad D, Afzal M, Imtiaz A. Effect of socioeconomic factors on malnutrition among children in Pakistan. Future Business Journal. 2020;6:1-11.
71. Fantay Gebru K, Mekonnen Haileselassie W, Haftom Temesgen A, Oumer Seid A, Afework Mulugeta B. Determinants of stunting among under-five children in Ethiopia: a multilevel mixed-effects analysis of 2016 Ethiopian demographic and health survey data. BMC pediatrics. 2019;19:1-13.
72. Wamani H, Åstrøm AN, Peterson S, Tumwine JK, Tylleskär T. Boys are more stunted than girls in sub-Saharan Africa: a meta-analysis of 16 demographic and health surveys. BMC pediatrics. 2007;7:1-10.
73. Mertens A, Benjamin-Chung J, Colford Jr JM, Coyle J, Van der Laan MJ, Hubbard AE, et al. Causes and consequences of child growth faltering in low-resource settings. Nature. 2023;621(7979):568-76.
74. Nyamasege C, Kimani-Murage E, Wanjohi M, Kaindi D, Wagatsuma Y. Effect of maternal nutritional education and counselling on children's stunting prevalence in urban informal settlements in Nairobi, Kenya. Public health nutrition. 2021;24(12):3740-52.
75. Teshome B, Kogi-Makau W, Getahun Z, Taye G. Magnitude and determinants of stunting in children underfive years of age in food surplus region of Ethiopia: the case of west gojam zone. Ethiopian Journal of Health Development. 2009;23(2).
76. Nor SM, Mwajuma J, Mohamed MAA. Determinants of Malnutrition among Children under five years in SOS Hospital, Mogadishu. 2016.
77. Niyigena DI, Semayira CA, Mutabazi M, Ntirushwamaboko N, de Dieu Habimana J, Iyakaremye D, et al. Feeding practices and nutritional status among children aged from six to 23 months in Western Province, Rwanda: a cross-sectional study. Rwanda Journal of Medicine and Health Sciences. 2023;6(2):228-38.
78. Marume A, Archary M, Mahomed S. Predictors of stunting among children aged 6–59 months, Zimbabwe. Public Health Nutrition. 2023;26(4):820-33.
79. Investigators M-EN. Relationship between growth and illness, enteropathogens and dietary intakes in the first 2 years of life: findings from the MAL-ED birth cohort study. BMJ global health. 2017;2(4):e000370.
80. González-Fernández D, Cousens S, Rizvi A, Chauhadry I, Soofi SB, Bhutta ZA. Infections and nutrient deficiencies during infancy predict impaired growth at 5 years: Findings from the MAL-ED study in Pakistan. Frontiers in Nutrition. 2023;10:1104654.
81. Jardí C, Casanova BD, Arija V. Nutrition education programs aimed at african mothers of infant children: a systematic review. International Journal of Environmental Research and Public Health. 2021;18(14):7709.
82. Rahman M. Association between order of birth and chronic malnutrition of children: a study of nationally representative Bangladeshi sample. Cadernos de saude publica. 2016;32:e00011215.
83. Khan REA, Raza MA. Nutritional status of children in Bangladesh: measuring composite index of anthropometric failure (CIAF) and its determinants. 2014.
84. Shapiro-Mendoza C, Selwyn BJ, Smith DP, Sanderson M. Parental pregnancy intention and early childhood stunting: findings from Bolivia. International journal of epidemiology. 2005;34(2):387-96.
85. Ukwuani FA, Suchindran CM. Implications of women's work for child nutritional status in sub-Saharan Africa: a case study of Nigeria. Social science & medicine. 2003;56(10):2109-21.
86. Saaka M, Aggrey B. Effect of birth interval on foetal and postnatal child growth. Scientifica. 2021;2021(1):6624184.
87. Zhao M, Wu H, Liang Y, Liu F, Bovet P, Xi B. Breastfeeding and mortality under 2 years of age in sub-Saharan Africa. Pediatrics. 2020;145(5):e20192209.

88. Dasgupta DP, Sujon MSH, Afroz M, Mazumder S, Suman SM, Khan MTF. Impact of Birth Spacing and Maternal-Child Risk Factors on Childhood Stunting, Wasting, Underweight, and their Coexistence in South Asia. Wasting, Underweight, and their Coexistence in South Asia.
89. Damtie Y, Kefale B, Yalew M, Arefaynie M, Adane B. Short birth spacing and its association with maternal educational status, contraceptive use, and duration of breastfeeding in Ethiopia. A systematic review and meta-analysis. PLoS One. 2021;16(2):e0246348.
90. Gebru KF HW, Temesgen AH, Seid AO, Mulugeta BA. Determinants of stunting among under-five children in Ethiopia: a multilevel mixed-effects analysis of 2016 Ethiopian demographic and health survey data. BMC pediatrics. 2019; 19(1):1–13. 2019.
91. Moges B FA, Meseret S, Doyore F. Magnitude of stunting and associated factors among 6–59 months old children in Hossana Town, Southern Ethiopia. Journal of Clinical Research & Bioethics. 2015; 6(1):1. 2015.
92. Agho KE IK, Bowe SJ, Jacobs J, Dibley MJ. . Prevalence and risk factors for stunting and severe stunting among under-fives in North Maluku province of Indonesia. BMC pediatrics. 2009; 9(1):1–10. https://doi.org/10.1186/1471-2431-9-64 PMID: 19818167. 2009.
93. Tiwari R, Ausman M. L., & Emwinyore Agho, K.(2014). Determinants of stunting and severe stuntingamong under-fives: evidence from the 2011 Nepal Demographic and Health Survey. BMC pediatrics.14:239.
94. Rahman A, Chowdhury S. Determinants of chronic malnutrition among preschool children in Bangladesh. Journal of biosocial science. 2007;39(2):161-73.
95. Yimer G. Malnutrition among children in Southern Ethiopia: Levels and risk factors. 2000.
96. Goddard FG, Hunegnaw BM, Luu J, Haneuse S, Zeleke M, Mohammed Y, et al. Child stunting from birth to age two years: The Birhan Cohort in Ethiopia. medRxiv. 2023:2023.05. 20.23290246.
97. Habtu M, Agena AG, Umugwaneza M, Mochama M, Munyanshongore C. Effectiveness of integrated maternal nutrition intervention package on birth weight in Rwanda. Frontiers in Nutrition. 2022;9:874714.
98. Bigool M. Effects of free maternal healthcare on stunting in children under five years of age: Evidence from Ghana. Development Policy Review. 2024;42(4).
99. T. P. Review of Maternal Effects on Early Childhood Stunting. 2014. 2014.
100. Budhathoki SS BA, Gurung R, Gurung A, Ashish K. Stunting among under 5-year-olds in Nepal: trends and risk factors. Maternal and child health journal. 2020;24(1):39–47. 2020.
101. Mzumara B BP, Halwiindi H, Mugode R, Banda J. Factors associated with stunting among children below five years of age in Zambia: evidence from the 2014 Zambia demographic and health survey. BMC nutrition. 2018;4(1):1–8. pmid:32153912. 2018.
102. Akombi BJ AK, Hall JJ, Wali N, Renzaho A, Merom D. . Stunting, wasting and underweight in sub-Saharan Africa: a systematic review. International journal of environmental research and public health. 2017;14(8):863. pmid:28788108. 2017.
103. Makoka D, Masibo PK. Is there a threshold level of maternal education sufficient to reduce child undernutrition? Evidence from Malawi, Tanzania and Zimbabwe. BMC pediatrics. 2015;15(1):96.
104. Ickes S, Baguma C, Brahe CA, Myhre JA, Adair L, Bentley M, et al. Maternal participation in a nutrition education program in Uganda is associated with improved infant and young child feeding practices and feeding knowledge: a post-program comparison study. BMC nutrition. 2017;3(1):32.
105. Asgedom YS SB, Mare KU, Asmare ZA, Asebe HA, Kase BF, et al. Levels of stunting associated factors among under-five children in Ethiopia: A multi-level ordinal logistic regression analysis. 2024.
106. Finlay JE ÖE, Canning D. The association of maternal age with infant mortality, child anthropometric failure, diarrhea and anemia for first births: evidence from 55 low-and middle-income

countries. BMJ open. 2011;1(2):e000226. [DOI] [PMC free article] [PubMed] [Google Scholar][Ref list]. 2011.

107. Chilinda ZB, Wahlqvist ML, Lee M-S, Huang Y-C. Higher maternal autonomy is associated with reduced child stunting in Malawi. Scientific reports. 2021;11(1):3882.

108. Behailu T. AM, Mache T., Girma A. Prevalence and risk factors of child malnutrition in community based nutrition program implementing and nonimplementing districts from South East Amhara, Ethiopia. Open Access Library Journal. 2014;1(3):1–17. doi: 10.4236/oalib.1100425. 2014.

109. Shiferaw Abeway BG, 2 Rajalakshmi Murugan,2 Masresha Assefa,3, and Yohannes Mehretie Adinew. Stunting and Its Determinants among Children Aged 6–59 Months in Northern Ethiopia: A Cross-Sectional Study. 2018.

110. Abeway S GB, Murugan R, Assefa M, Adinew YM. . Stunting and its determinants among children aged 6–59 months in northern Ethiopia: a cross-sectional study. J Nutr Metab. 2018;2018:1–8. doi:10.1155/2018/1078480. 2018.

111. Bupe B. BML, Mapoma C. C., Mutombo N. . Factors associated with stunting among children aged 6–23 months in Zambian: Evidence from the 2007 Zambia Demographic and Health Survey. International Journal of Advanced Nutritional and Health Science. 2015;2(1). 2015.

112. Forero-Ramirez N GL, Bedi A, Sparrow R. . Child malnutrition and prenatal care: evidence from three Latin American countries. Revista Panamericana de Salud Pública. 2014;35:163–171. 2014.

113. Ramirez N GL, Bedi AS, Sparrow R. . Child malnutrition and antenatal care: evidence from three Latin American countries. ISS Working Paper Series. 2012;536(536):1–28. 2012.

114. Svefors P SO, Ekstrom E-C, et al. . Relative importance of prenatal and postnatal determinants of stunting: data mining approaches to the MINIMat cohort, Bangladesh. BMJ Open. 2019;9 (8):e025154. doi:10.1136/bmjopen-2018-025154. 2019.

115. Habibov NN FL. Does prenatal healthcare improve child birthweight outcomes in Azerbaijan? Results of the national Demographic and Health Survey. Econ Hum Biol. 2011;9(1):56–65. doi:10.1016/j.ehb.2010.08.003. 2011.

116. G. G-G. The impact of adequate prenatal care on urban birth outcomes: an analysis in a developing country context. Econ Dev Cult Change. 2013;62(1):95–130. doi:10.1086/671716. 2013.

117. Langlois ÉV, Miszkurka M, Zunzunegui MV, Ghaffar A, Ziegler D, Karp I. Inequities in postnatal care in low-and middle-income countries: a systematic review and meta-analysis. Bulletin of the World Health Organization. 2015;93:259-70G.

118. Abdulraheem A, Ononokpono D, Raimi M. Breaking barriers to safe motherhood: how social, cultural, and geographic inequalities shape skilled birth attendance in Nigeria. Sociol Int J. 2025;9(5):188-200.

119. Megabiaw B RA. Prevalence and determinants of chronic malnutrition among under-5 children in Ethiopia. International Journal of Child Health and Nutrition. 2013;2(3):230–6. 2013.

120. stunting UC. US Cww. stunting: and waht it is and what it means 2019 [cited 2021]. Available from: https://www.concernusa.org/story/what-is-stunting/. 2019.

121. Tadesse SE MT. Prevalence and Associated Factors of Stunting Among Children Aged 6–59 Months in Delanta District; North East Ethiopia. 2019.

122. Elisaria E, Mrema J, Bogale T, Segafredo G, Festo C. Effectiveness of integrated nutrition interventions on childhood stunting: a quasi-experimental evaluation design. BMC nutrition. 2021;7(1):17.

123. Mwamba MS, Gerrior S, Taylor R. Relationship between single motherhood status and stunting among children under 5 in Kgatleng, Botswana. Journal of Social, Behavioral, and Health Sciences. 2022;16(1):40-7.

124. Gibbs CM, Wendt A, Peters S, Hogue CJ. The impact of early age at first childbirth on maternal and infant health. Paediatric and perinatal epidemiology. 2012;26:259-84.
125. Prof Robert E Black MrjePCGV, MDb · Prof Susan P Walker, PhDc · Prof Zulfiqar A Bhutta, PhDd,* · Prof Parul Christian, DrPHa,* · Mercedes de Onis, MDe,*· et al. . Maternal and child undernutrition and overweight in low-income and middle-income countries. 2013.
126. Amaha ND, Woldeamanuel BT. Maternal factors associated with moderate and severe stunting in Ethiopian children: analysis of some environmental factors based on 2016 demographic health survey. Nutrition Journal. 2021;20:1-9.
127. Berhe K, Seid O, Gebremariam Y, Berhe A, Etsay N. Risk factors of stunting (chronic undernutrition) of children aged 6 to 24 months in Mekelle City, Tigray Region, North Ethiopia: An unmatched case-control study. Plos one. 2019;14(6):e0217736.
128. Kamal S. Socio-economic determinants of severe and moderate stunting among under-five children of rural Bangladesh. Malaysian journal of nutrition. 2011;17(1).
129. Kota K, Pongou R, Chomienne M-H. Impact of household food insecurity on the use of maternal health services in the Savanes region, Togo: a qualitative study. BMC Public Health. 2025;25(1):2040.
130. Okyere J, Budu E, Aboagye RG, Seidu AA, Ahinkorah BO, Yaya S. Socioeconomic determinants of the double burden of malnutrition among women of reproductive age in sub-Saharan Africa: A cross-sectional study. Health Science Reports. 2024;7(5):e2071.
131. Yaya S, Oladimeji O, Odusina EK, Bishwajit G. Household structure, maternal characteristics and children’s stunting in sub-Saharan Africa: evidence from 35 countries. International Health. 2022;14(4):381-9.
132. Bogale TY, Bala ET, Tadesse M, Asamoah BO. Prevalence and associated factors for stunting among 6–12 years old school age children from rural community of Humbo district, Southern Ethiopia. BMC public health. 2018;18:1-8.
133. Asefa A, Girma D, Kaso AW, Ferede A, Agero G, Beyen TK. Prevalence of stunting and associated factors among under-five children in Robe Woreda, Arsi zone, Ethiopia. International Journal of Africa Nursing Sciences. 2024;21:100782.
134. Desai S, Alva S. Maternal education and child health: Is there a strong causal relationship? Demography. 1998;35(1):71-81.
135. Sarma H KJ, Asaduzzaman M, Uddin F, Tarannum S, Hasan MM, et al. Factors influencing the prevalence of stunting among children aged below five years in Bangladesh. Food and nutrition bulletin. 2017;38(3):291–301. pmid:28758423. 2017.
136. Ali S AA. Factors Associated with Stunting among Children Under Five Years of Age in Pakistan: Evidence from PDHS 2012–13. J Community Public Health Nurs. 2018;4:1–4. 2012–13.
137. Mbuya NV CR, Morimoto T, Thitsy S. . Media and Messages for Nutrition and Health: Assessing Media Appropriateness for Nutrition and Health-Related Social and Behavior Change Communication in Four High Stunting-Burden Provinces of Lao PDR. World Bank; 2020. 2020.
138. Woldesenbet B, Tolcha A, Tsegaye B. Water, hygiene and sanitation practices are associated with stunting among children of age 24-59 months in Lemo district, South Ethiopia, in 2021: community based cross sectional study. BMC nutrition. 2023;9(1):17.
139. Seiler J, Libby TE, Jackson E, Lingappa J, Evans W. Social media–based interventions for health behavior change in low-and middle-income countries: systematic review. Journal of medical Internet research. 2022;24(4):e31889.
140. ZT. W. Magnitude and determinants of stunting among children in Africa: a systematic review. Current Research in Nutrition and Food Science Journal. 2014;2(2):88–93. 2014.
141. Akombi BJ, Agho KE, Hall JJ, Wali N, Renzaho AM, Merom D. Stunting, wasting and underweight in sub-Saharan Africa: a systematic review. International journal of environmental research and public health. 2017;14(8):863.

142. Budhathoki SS BA, Gurung R, Gurung A, Ashish K. . Stunting among under 5-year-olds in Nepal: trends and risk factors. Maternal and child health journal. 2020;24(1):39–47. 2020.

143. Tsega Y, Endawkie A, Kebede SD, Abeje ET, Enyew EB, Daba C, et al. Trends of wealth-related inequality in stunting and its contributing factors among under-five children in Ethiopia: Decomposing the concentration index using Ethiopian Demographic Health Surveys 2011–2019. PloS one. 2025;20(2):e0314646.

144. Kountchou A, Sonne S, Gadom G, editors. The local impact of armed conflict on children's nutrition and health outcomes: evidence from Chad. CSAE Conference: University of Oxford; 2019.

145. Baye K, Laillou A, Chitweke S. Socio-economic inequalities in child stunting reduction in sub-Saharan Africa. Nutrients. 2020;12(1):253.

146. Tariq J SA, Zakar R, Zakar MZ, Fischer F. . Factors associated with undernutrition in children under the age of two years: secondary data analysis based on the Pakistan demographic and health survey 2012–2013. Nutrients. 2018;10(6):676. 2018.

147. Woldeamanuel BT TT. Risk factors associated with under-five stunting, wasting, and underweight based on Ethiopian Demographic Health Survey datasets in Tigray region, ethiopia. Journal of nutrition and metabolism. 2019;2019. 2019.

148. Smith LC RM, Ndiaye A. Why is child malnutrition lower in urban than in rural areas? Evidence from 36 developing countries. World development. 2005;33(8):1285–305. 2005.

Ethical Letter

Click here to access/download

Other

AuthLetter_215093.pdf

Prevalence of Stunting and its Determinants Among Children Under Five in 35 Sub-Saharan African Countries (2011–2024): Insights from recent Demographic Health Survey Data Using a Generalized Linear Mixed-Effects Model with Robust Poisson Regression

~~**Authors name and Email address**~~

Author's Name and Email

[1]Abdulkerim Hassen Moloro*, Email: habdulkerim4@gmail.com

[1]Kebede Gemeda Sabo, Email: kebedegemeda2008@gmail.com

[1]Kusse Urmale Mare, Email: kussesinbo@gmail.com

[2]Beriso Furo Wengoro, Email: berisafuro@gmail.com

[3]Eshetu Elfios Endrias, Email: eshetuelf21@gmail.com

[4]Roda Mehadi Ibrahim, Email: ~~Rozimahdi04@gmail.com~~rozimahdi04@gmail.om

[5]Teshager Dubie, Email: teshagerdubie19@gmail.com

[6]Oumer Abdulkadir Ebrahim, Email: oumerbadana@gmail.com

[7]Begetayinoral Kussia Lahole, Email: begetakussia92@gmail.com

Authors Affiliation

[1]Department of Nursing, College of Medicine and Health Sciences, Samara University, Samara, Ethiopia

[2]Department of Biomedical Sciences, College of Medicine and Health Sciences, Samara University, Samara, Ethiopia

[3]Department of Nursing, School of Nursing, College of Medicine and Health Sciences, Wolaita Sodo University, Wolaita Sodo, Ethiopia

[4]Department of midwifery, College of Medicine and Health Sciences, Jigjiga University, Jigjiga, Ethiopia

[5]Department of Veterinary Medicine, College of Veterinary Medicine and Animal Sciences, Samara University, Semera, Ethiopia

[6]Department of ~~Public Health~~Veterinary Medicine, College of Veterinary Medicine and ~~Health Sciences~~Anima Science, Samara University, Samara, Ethiopia

[7]Department of ~~Biomedical Sciences~~Midwifery, College of Medicine and Health Sciences, ~~Samara~~Arba Minch University, ~~Samara~~Arba Minch, Ethiopia

Corresponding Authors

Abdulkerim Hassen Moloro*,

Department of Nursing

College of Medicine and Health Science

Samara University

Samara, Ethiopia
~~Phone: +251-935912095/+251952695335~~
Email: habdulkerim4@gmail.com, ORCID: https://orcid.org/0000-0002-6081-4108
~~Email: habdulkerim4@gmail.com~~

Formatted: Font color: Custom Color(RGB(0,0,153))

Abstract

Formatted: Font: 18 pt

Background: Despite efforts by initiatives like the World Bank's 'All Hands-on Deck' and UNICEF's programs to address stunting through multisectoral approaches, the burden of stunting remains alarmingly high in sub-Saharan Africa. This study utilized recent large-scale survey data from 35 SSA countries (2011–2024) to estimate the pooled prevalence of stunting and its determinants among children under ~~five.~~5 years of age. Key variables such as antenatal care visits, postnatal care, and maternal nutritional indicators, which previous ~~study~~studies did not ~~accounted~~account for, are incorporated ~~in to~~into the analysis.

Methods: A secondary analysis was conducted using recent demographic and health survey data (2011–2024) from 35 sub-Saharan African (SSA) countries. A total weighted sample of 191,~~954 under five~~953 children under 5 years of age was included in the analysis. Descriptive and inferential analyses were performed using STATA 17. Forest plots were utilized to illustrate both pooled and country-specific stunting rates. Determinants of stunting were identified through a multilevel mixed-effects Poisson regression model with robust variance. The adjusted prevalence ~~ratio~~ratios and their 95% confidence intervals were used to ~~indicate~~ assess the strength and statistical significance ~~and the strength~~ of associations.

Model comparison was evaluated by model statistical summary such as Akaike's Information Criterion (AIC), Bayesian Information Criterion (BIC), log-likelihood (LL), and deviance (-2*LL).

Result: The pooled prevalence of stunting among ~~under five~~ children under 5 years of age in ~~SSA~~35 sub-Saharan African countries was 29.~~72~~89% (95% CI: 26.~~55, 32.90~~63, 33.14%), with the lowest level in Gabon (13.~~74~~91%) and the highest in Burundi (55.~~74~~80%). Being male children (aPR~~: ~~=1.24, 95% CI: 1.21~~, ~~–1.26), ~~children~~ being aged 12 months or older ~~age~~(aPR~~ = 2.32, 95% CI: 2.23, 2.42), higher birth order(aPR = ~~: ≥1.~~08, 95% CI: 1.03, 1.12), small birth size(aPR = 1.16, 95% CI: 1.13, 1.19), maternal aged 15–24(aPR = 1.09, 95% CI: 1.06, 1.13~~81, p<0.01), insufficient antenatal care (ANC) visits (aPR~~ = ~~: ≥1.17, ~~95% CI: 1.13, 1.21~~p<0.01), lack of postnatal visits(aPR

= 1.03, 95% CI: 1.07, 1.05), lower maternal children perceived as small or average at birth (aPR: ≥1.16, p<0.01), mother without a higher education (aPR = : ≥1.94, 95% CI: 1.74, 2.18), home delivery(aPR = 1.11, 95% CI: 1.08, 1.14), single marital status(aPR = 1.04, 95% CI: 1.02, 1.07), maternal underweight(aPR = 1.12, 95% CI: 1.08, 1.16), lack of maternal media exposure(aPR = 1.11, 95% CI: 1.08, 1.13), use of unimproved water sources(aPR = 1.02, 95% CI: 1.03, 1.05), large family size (aPR = 1.04, 95% CI: 1.04, 1.08), using unclean cooking fuels(aPR = 1.25, 95% CI: 1.18, 1.32), lower household wealth (aPR = 1.31, 95% CI: 1.24, 1.38) andp<0.01), living in rural area (aPR = 1.07, 95% CI: 1.04, 1.11) a poor or average wealth household (aPR: ≥1.23, p<0.01) were significant predictorpredictors of stunting. Conversely, maternal overweight (aPR = 0.81, 95% CI: 0.77, 0.84) and obese mothers (aPR = 0.88, 95% CI: 0.85, 0.90) were associated lower prevalence of stunting.

Conclusion: studyStudy revealed a stunting prevalence of 29.7289% among children under five5 years of age across 35 sub-Saharan African countries, with significant country-level variations and rates exceeding 30% in 1415 countries, signaling a major public health concern. Key individual, household, and contextual factors associated with stunting were identified, emphasizing the need for targeted interventions, such as improvingimmediate actions expanding antenatal and postnatal care, promoting facility-based deliveries, enhancing maternal and child healthcareeducation, and media outreach. Long term strategies must tackle poverty, food systems, and equitable nutrition access, addressing economic disparities, and bridging the rural-urban divide.supported by governance and stability. A multisectoral approach integrating health, education, agriculture, and WASH sectors is crucial for combating stunting, alongside futureWASH, and social protection ensures substantiable child growth, complemented by longitudinal research to better understand systemic and causal determinants.for policy coherence.

Formatted: Font: Not Bold

Keywords: Stunting, Under-five, Demographic Health Survey, Sub-Saharan Africa

Introduction

Formatted: Font: 18 pt

The World Food Programme (WFP) defines malnutrition as a condition in which an individual's physical function is so compromised that they cannot maintain key bodily processes like growth, pregnancy, lactation, physical work, or recovering from illnesses(1). Stunting is a significant public health issue in developing nations especially in SSA, resulting from chronic and repeated malnutrition that impairs physical, psychological, and cognitive growth(2). The World Health

Organization (WHO) defines stunting as a length/height-for-age measurement that falls below -2 Standard Deviations (SD) from the median(3).

As of 2019, the proportion of malnourished people in Eastern and Middle sub-regions of Sub-Saharan Africa were estimated to be 27% and 29% of the total population, respectively(4). The Food and Agriculture Organization (FAO) reported that in 2020, 161 million children under the age of 5 were stunted(5). Between 2000 and 2017, the number of children under 5 experiencing stunting increased from 50.6 to 58.7 million, during the same period(6).

In 2020, the year of the pandemic, an estimated 149.2 million children under five were stunted, 59 million were wasted, 85 million were moderately or severely underweight, and 38.9 million were overweight worldwide (7, 8). Currently, Africa faces a high burden of undernutrition and more specifically stunting(9). A study conducted on under-five (U-5) children from the 2018 Nigeria Demographic and Health Survey (NDHS) in 2024 revealed that the prevalence of child stunting were 36.51% (10). Another study conducted in 35 sub-Saharan Africa reported that, the pooled prevalence of stunting was 35%, specifically Burundi had the highest rates of stunting at 59% while Gabon had the lowest rates at 17%(11).

Childhood stunting has both short-term and long-term consequences, such as increased child morbidity and mortality, impaired cognitive and physical development, and a higher risk of infectious and non-infectious diseases(12, 13). Additionally, stunting in female children can negatively affect maternal reproductive outcomes later in life(14, 15). Research has identified several significant predictors of stunting in children under five, including the child's sex and age, drinking water source, maternal age and education, place of residence, region, household wealth, birth interval, and duration of breastfeeding (16, 17). Other studies also found that, childhood stunting results from inadequate food intake, perceived birth weight, mother's BMI, diarrhea, infections, and poor sanitationStunting defined as a length/height-for-age measurement below -2 Standard Deviations from the median(1), remains a critical and persistent public health crisis in sub-Saharan Africa (SSA). It is the result of chronic malnutrition, a condition that compromises physical function and the maintenance of key bodily processes(2), and it leads to significant impairments in physical, psychological, and cognitive growth(3).

Sub-Saharan Africa carries a disproportionately heavy burden of childhood stunting, with pooled data from 35 countries indicating an pooled prevalence of 35%(4). National disparities are remarkable, ranging from as low as 17% in Gabon to as high as 59% in Burundi(5), while Nigeria reports a prevalence of 36.5% among children under five(6). This situation persists despite a global decline in stunting rates, as the absolute number of affected children in Africa has risen(7), underscoring a regional emergency within the worldwide context of 161 million stunted children(8).

The consequences in SSA are severe and lifelong, including increased child morbidity and mortality, impaired development, and higher disease risk(9, 10). Research has identified that stunting arises from a wide range of interrelated causes, which can be categorized into individual-level, household, and contextual factors. At the individual level, determinants include the child's sex and age, maternal age and educational attainment, and birth interval(11, 12). Additional contributors such as birth order (13), antenatal care utilization (14), and duration of exclusive breastfeeding(13, 15-19) have also been identified. Furthermore, studies report that inadequate food intake, low perceived birth weight, maternal body mass index (BMI), and poor sanitation significantly increase the risk of childhood stunting~~(18-20)~~(20-22).

~~Reducing stunting in sub-Saharan Africa (SSA) continues to pose a significant challenge, yet various programmatic initiatives are being introduced to drive progress. The World Bank's "All Hands on Deck" initiative advocates for a multisectoral strategy, involving sectors such as agriculture, education, social protection, and water, sanitation, and hygiene (WASH)(21). Similarly, UNICEF's programs prioritize maternal, infant, and young child nutrition, micronutrient supplementation, the management of severe acute malnutrition, nutrition in emergencies, and enhancing water and sanitation at the community level(22). Despite these efforts, the burden of stunting remains high in sub-Saharan African countries.~~

~~The Sustainable Development Goals (SDGs), specifically Target 2.2, aim to eliminate all forms of malnutrition(23). This includes an ambitious goal to reduce stunting by 40% by 2025, aiming for a global prevalence of around 15%(23). However, despite significant progress in reducing child stunting over the past two decades(24), it's unlikely that many Sub-Saharan African countries~~

would meet the Sustainable Development Goals 2.2 targets to end hunger and all forms of malnutrition by 2030(4).

This study estimated the pooled prevalence of stunting among all under-five children in 35 sub-Saharan African countries using recent survey data from 2011 to 2024. To address the potential overestimation of association strength that arises when using odds ratios for common binary outcomes in cross-sectional data, the study applied Poisson regression with robust error variance(25, 26). Additionally, this analysis included key variables such as antenatal care (ANC) visits during pregnancy, postnatal care visits, and maternal nutritional status indicators like BMI and height, which were not accounted for in previous studies(11).

Pooled analyses offer significant advantages, such as increasing the sample size, which enhances the study's statistical power to detect true effect sizes and improves the generalizability of the findings. They also enable comparisons of outcomes across sub-regions. From a public health perspective, pooled analyses are particularly valuable in sub-Saharan Africa, a region heavily burdened by childhood undernutrition especially stunting. Identifying common factors associated with stunting can assist international, regional, and sub-regional organizations in designing targeted public health programs. This, in turn, can foster collaboration among countries, allowing them to share successful stunting reduction strategies and pool resources to combat childhood stunting more effectively. Therefore, this study aimed to investigate the pooled prevalence of stunting and its determinants among children under five in 35 sub-Saharan Africa by utilizing generalized linear mixed-effects modeling with robust Poisson regression analysis, based on the most recent 2011-2024 Demographic and Health Survey (DHS) data.

Household-level determinants encompass household size, particularly households with multiple under-five children (14, 16, 23), access to improved drinking water sources(15), and availability of toilet facilities(24) and household wealth index(23, 25). Contextual factors including place of residence(14) and geographical sub-region(11, 12) further affects child nutrition outcomes, underscoring the multifaceted nature of stunting and its dependence on both biological and socio-environmental conditions(Fig 1).

Stunting remains a major child health challenge in sub-Saharan Africa (SSA), despite decades of global and regional initiatives aimed at reducing malnutrition. Programs such as the World Bank's

"All Hands-on Deck" initiative, which promotes multisectoral strategies across agriculture, education, social protection, and WASH sectors, and UNICEF's maternal and child nutrition programs have sought to accelerate progress(26, 27). Nevertheless, the burden of the stunting in SSA continues to be alarming high, and many countries are unlikely to achieve the Sustainable Developmental Goal (SSD) 2.2 target of reducing stunting by 40% by 2025 and eliminating all forms of malnutrition by 2030(28, 29).

While single country and regional studies have provided valuable insights, their scope is often limited, making it difficult to capture the broader patterns and disparities across the content. This study addresses that gap pooling nationally representative data from 35 SSA countries collected between 2011 and 2024. By combining data at this scale, the analysis enhances statistical power using Poisson regression with robust error variance(30, 31) to reduce potential overestimation of odds ratios for common binary outcomes, improves generalizability, and allows for the identifications of common risk factors and cross-country variations that may not be visible in isolated studies.

Additionally, this analysis incorporated key variables such as antenatal care (ANC) visits during pregnancy, postnatal care visits, and maternal nutritional status indicators including BMI and height factors that were not considered in previous studies(4). By integrating these variables, the pooled evidence is uniquely positioned to inform regional policy, guide resource allocation, and support collaborative interventions across countries. This study therefore provides a more comprehensive and representative picture of stunting in sub-Saharan Africa (SSA), underscoring the urgent need for coordinated, evidence-based strategies to address this persistent public health challenge. Accordingly, the study aimed to determine the pooled prevalence and key determinants of stunting among children under 5 years of age in 35 SSA countries, utilizing generalized linear mixed-effects modeling with robust Poisson regression analysis based on the most recent 2011–2024 Demographic and Health Survey (DHS) data

Conceptual frame work

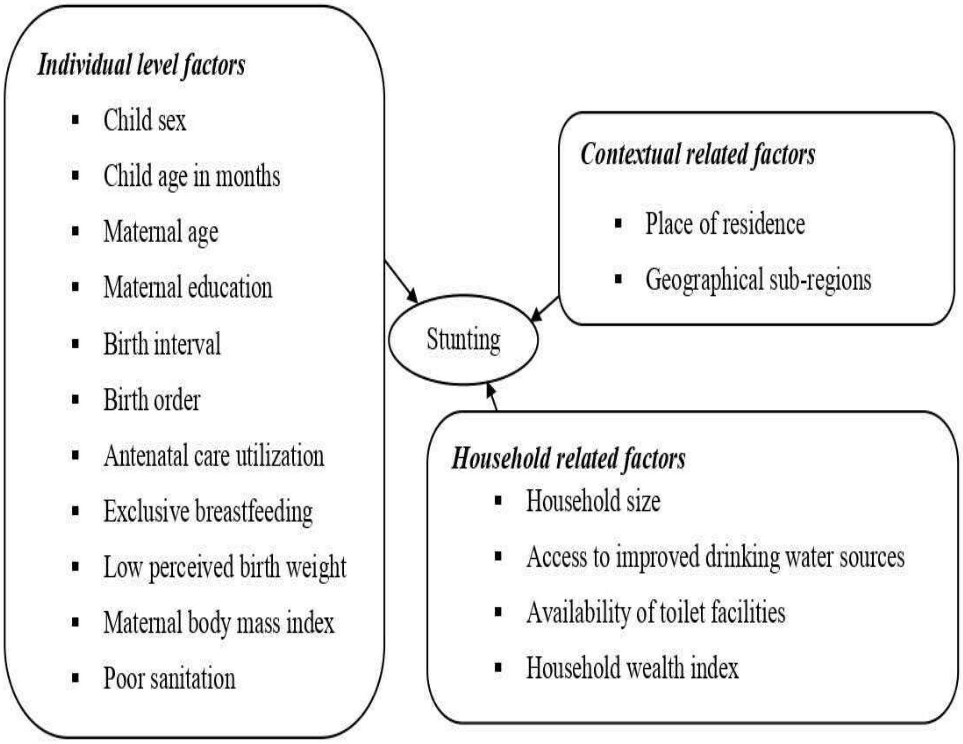

Figure 1: A conceptual framework showing link between stunting and its determinants among children under 5 years of age, 2011-2024.

Methods

Study Area, Design, and Period ~~and Data Source~~

This study conducted secondary analysis using data from the Demographic and Health Surveys (DHS) of 35 countries in Sub-Saharan Africa (SSA). The countries involved were Angola, Burkina Faso, Benin, Burundi, DR Congo, Republic of the Congo, Côte d'Ivoire, Cameroon, Ethiopia, Gabon, Ghana, Gambia, Guinea, Kenya, Comoros, Liberia, Lesotho, Madagascar, Mali, Mauritania, Malawi, Mozambique, Nigeria, Niger, Namibia, Rwanda, Sierra Leone, Senegal, Chad, Togo, Tanzania, Uganda, South Africa, Zambia, and Zimbabwe. The selection of country

Formatted: Font: 18 pt

Formatted: Font: 16 pt

Formatted: Font: 16 pt

Formatted: Font: 16 pt

was based on the recent survey year, availability of a standardized and unrestricted dataset, and presence of observations for the outcome variable in the datasets.

The DHS surveys across all countries employed a cross-sectional study design to collect data on basic sociodemographic characteristics and various health indicators, including nutrition, maternal, and child health. For the current analysis, we included the countries that have their recent DHS conducted between 2011 and 2024. Since its inception, DHS has been conducted in over 85 low- and middle-income countries (LMICs) worldwide. ~~The data analyzed in this study were derived from the DHS women's questionnaire, specifically targeting children under the age of 5 years (under 60 months), and from the Kids Record dataset (KR file) across 35 countries.~~ ~~**Population, sampling technique and data collection**~~

Formatted: Space After: 10 pt

Population, sampling technique and weight

The source population for this study comprised all children under five years old in 35 SSA countries. The study population included all ~~under-five~~ children under 5 years of age in the survey. Across all countries, the surveys used a multistage stratified cluster sampling technique to select the study participants. In the first stage, each country was divided into clusters, and clusters were randomly selected based on the probability proportional to their contribution to overall country's population. In the second stage, using the housing census as a sampling frame, a representative number of households was selected from each cluster.

~~A standardized data collection tool and face-to-face interview were used to collect the survey's data. For this study, since we intended to examine the pooled prevalence of stunting and its determinants, we used the data of the children under the age of 5 years (under 60 months) from the Kids Record dataset (KR file) across 35 countries. Based on DHS guideline (https://www.dhsprogram.com/pubs/pdf/DHSG1/Guide_to_DHS_Statistics_DHS-8.pdf), children with missing data were excluded from the study. Finally, after handling the missing observations, a weighted sample of **191,954** children aged 0 to 59 months with complete data on the variables of interest were included in the study (Table 1). Details about DHS methodology can be accessed online (https://dhsprogram.com/Methodology/index.cfm).~~

To account for the complex survey design, non-response, and to ensure comparability across countries while preventing any single country or survey year from disproportionally influencing

the pooled estimates, DHS sampling weights were applied as recommended. The weight variable (v005) was rescaled by dividing by 1,000,000 and incorporated into all analyses using the *svyset* command in Stata, accounting for primary sampling units (v021) and strata (v022). This produced a weighted analytic sample of **191, 953** children aged 0 to 59 months with complete data on the variables of interest (Table 1). Additionally, sensitivity analyses were conducted to assess the influence of individual countries by systematically excluding one country at a time and recalculating the pooled prevalence. This approach adjusts for unequal probabilities of selection and non-response within each survey, ensuring that each country's contribution to the pooled prevalence is proportional to its population size.

Table-1: Survey years and sample sizes of children under ~~five~~5 years of age from 35 Sub-Saharan African (SSA) countries included in the study, from 2011 to 2024

Country	Survey year	Unweighted sample size	Weighted sample size
**Angola**	~~2015-2016~~2023-2024	~~6,268~~ 5,039	~~5,837~~4,800
**Burkina Faso**	2021	5,~~712~~ 731	5,~~674~~696
**Benin**	2017-2018	11,~~631~~ 670	11,~~682~~717
**Burundi**	2016-2017	6,~~039~~ 048	6,~~213~~222
**DR Congo**	~~2013-2014~~2023-2024	~~8,059~~10,745	~~7,900~~9,734
**Congo**	2011-2012	4,475	3,859
**Cote d'Ivoire**	2021	4,~~738~~779	4,~~265~~300
**Cameroon**	2018-2019	4,~~445~~497	4,~~646~~707
**Ethiopia**	2019	5,~~056~~076	4,~~952~~972
**Gabon**	2019-2021	5,~~311~~ 343	~~4,979~~5,009
**Ghana**	2022-2023	4,~~395~~ 405	4,~~037~~051
**Gambia**	2019-2020	3,~~805~~ 811	3,~~503~~511
**Guinea**	2018	3,~~371~~466	3,~~278~~374
**Kenya**	2022	17,~~283~~ 327	15,~~294~~336
**Comoros**	2012	2,387	2,468
**Liberia**	2019-2020	2,~~440~~445	2,~~161~~166
**Lesotho**	2023-2024	1,~~089~~093	~~980~~983
**Madagascar**	2021	5,~~756~~778	5,~~695~~719
**Mali**	~~2018~~2023-2024	~~8,223~~13,936	~~8,764~~14,163
**Mauritania**	2019-2021	9,~~795~~830	9,~~916~~955

**Malawi**	2015-2016	5,~~110~~ 149	5,~~127~~160
**Mozambique**	2022-2023	3,~~723~~733	3,~~849~~858
**Nigeria**	~~2018~~ 2023-2024	~~11,314~~9,410	~~11,361~~9,341
**Niger**	2012	~~4,771~~5,576	5,~~113~~112
**Namibia**	2013	1,558	1,438
**Rwanda**	2019-2020	3,~~806~~809	3,~~914~~918
**Sierra Leone**	2019	4,~~100~~136	4,~~006~~044
**Senegal**	2023	4,~~466~~476	4,~~251~~258
**Chad**	2014-2015	9,826	9,805
**Togo**	2013-2014	3,185	3,058
**Tanzania**	2022	4,~~791~~807	4,~~854~~866
**Uganda**	2016	4,~~390~~423	4,~~315~~350
**South Africa**	2016	1,~~075~~113	1,~~049~~080
**Zambia**	~~2018-2019~~2024	~~8,681~~3,810	~~8,545~~3,710
**Zimbabwe**	2015	4,~~914~~ 957	5,~~166~~ 212
**Total**		**~~195,988~~ 197,044**	**191,~~954~~ 953**

Data source

The data for this study were obtained from the DHS women's questionnaire, focusing on children under five years of age (under 60 months), and from the Kids Record dataset (KR file) across 35 countries. All datasets were sourced directly from the official Demographic and Health Surveys (DHS) program website (http://dhsprogram.com/).

Data extraction and management of missing observations

Prior to data extraction, we identified countries with DHS datasets from surveys conducted between 2011 and 2024, as our aim was to include only those with data collected during this timeframe. A standardized data collection tool and face-to-face interview were used to collect the survey's data. To construct a pooled dataset, we first extracted variables relevant to the study from the data of the children under 5 years of age (under 60 months) from the Kids Record dataset (KR file) across 35 countries. The merged datasets from 35 sub-Saharan African countries were then appended by survey year, creating a pooled datasets for regional analysis of child stunting. Country

identifiers and survey weights were retained to account for sampling design and cross-country differences. Variables are then recoded and categorized consistently across all 35 DHS surveys using the Guide to DHS statistics to ensure compatibility.

The handling of anthropometric data in this analysis adhered to the standardized protocols outlined in the Guide to DHS Statistics(32). Children were excluded or dropped from the analyses of all anthropometric indices if they met any of the following criteria: (i) they were not weighed or measured during the survey, (ii) their recorded weight or height measurements were missing, (iii) their month or year of birth was unknown or missing (rendering accurate age calculation impossible for indices requiring it), or (iv) their derived height-for-age z-scores (HAZ) were flagged as biologically implausible (e.g., outside the range of -6 to +6 SD). These exclusions applied uniformly to both the denominator and numerator in prevalence analysis, constituting a complete-case analysis.

For the independent variables, complete case analysis was employed, retaining only observations with non-missing values for all covariates included in the final model. These steps enhance the transparency and validity of our findings in light of potential cross-country variations in data completeness. A detailed explanation of the DHS methodology, guidelines, and procedures for handling missing data is available on the DHS website(33).

Variables and measurements

Formatted: Font: 16 pt

Dependent Variable:

Stunting (height-for-age z-scores): height for age of children dichotomized as normal (not stunted) if height for age ≥-2 SD and stunted if height of age <-2 SD form WHO child growth reference ((height/age standard deviation (new who)). Weight measurements were obtained using light weight SECA mother-infant scales with a digital screen designed and manufactured under the guidance of UNICEF. Height measurements were obtained using the Shorr measuring board. Children younger than 24-months were measured for their height while lying down, and children older than 24 months were measured while standing.

Independent Variables: A total of 23 variables were ~~included in the~~incorporated into this study~~.~~ ~~Variables were chosen based on~~, selected through a systematic process that considered both their availability in the Demographic and Health Survey (DHS~~ dataset~~) and their documented

~~association~~associations with childhood stunting ~~from~~in prior ~~studies~~research~~(27-29)~~(34-36)~~. They~~. Each variable was chosen for its relevance to the study objectives and its role as a known or hypothesized determinants of stunting reflecting influences at individual, household, and contextual level. This study also included variables often overlooked in earlier work, such as antenatal care (ANC) visits during pregnancy postnatal care utilizations, and body mass index.

These variables were categorized into individual-level factors, household factors, and contextual factors. The individual-level factors included the sex of the child (male and female), age of the child in month, birth order (1, 2–4, and 5+), breastfeeding (still breastfeeding, ever breastfed and never breastfed) and perceived size at birth (large, average, and small). Other individual-level factors were maternal educational level (no formal education, primary, secondary, and higher), current maternal working status (yes and no), number of antenatal care visits during pregnancy (0, 1–3, and 4 or more) and postnatal checks within 2 months (yes and no). Variables such as maternal age (15–24 and 25–49), age at first birth, antenatal visits during pregnancy (0, 1–3, and 4 or more), place of delivery (home, health facility, other), marital status (single and married) and maternal nutritional status were also considered.

Household factors included ~~the source of drinking water (improved and unimproved), type of toilet facility (improved and unimproved),~~ household size (small, medium, and large), exposure to media (yes and no), ~~type of cooking fuel (clean and unclean),~~ and wealth index (~~poorest, poorer~~poor, middle, ~~richer, and richest~~rich). Contextual factors encompassed the place of residence (urban and rural) and geographical sub-regions (West, East, Central, and Southern).

Maternal nutritional status, described using Body Mass Index (BMI) according to the WHO adult BMI classification~~(30).~~(37). BMI was calculated by dividing each woman's weight in kilograms by the square of her height in meters (kg/m²). The survey utilized an electronic SECA 874 flat scale for weight measurements, specially designed for mobile use, and a shore measuring board for height. For analysis, women were categorized into four nutritional status groups based on their BMI: underweight (BMI < 18.5 kg/m²), normal weight (BMI 18.5–24.9 kg/m²), overweight (BMI 25.0–29.9 kg/m²), and obesity (BMI ≥ 30.0 kg/m²)~~(31).~~(38).

Data management and statistical analysis

Formatted: Font: 16 pt

Stata version 17 was utilized for data cleaning and analysis. Prior to the analysis, the presence of the outcome variable in the DHS dataset for each country was confirmed. All the variables considered in the study were reviewed for missing values. Subsequently, the datasets from 35 SSA countries were appended and weighted to maintain sample representativeness and obtain reliable estimates and standard errors. The pooled prevalence of child stunting was calculated using weighted data on the number of affected children with outcome variable and the total number of study participants in each country included in the analysis. The Stata command for meta-analysis “metan” was executed to present the country-specific and pooled estimates with 95% CI in a forest plot. To explore potential sources of heterogeneity, meta-regressions were conducted(39). Additionally, sensitivity analyses were performed to evaluate the influence of individual country on the overall pooled estimates (39).

A multilevel mixed-effects Poisson regression model with robust error variance was fitted to identify the determinants of malnutrition among under five children’s in SSA. We applied Poisson regression with robust error variance since the odds ratio estimated using a common binary outcome from cross-sectional data may significantly overestimate the strength of association(26, 32). In addition, to account for the dependency of data due to the nested nature of DHS (i.e., children were nested within the households, and households were nested within the clusters), a multilevel mixed-effects logistic regression modelling was applied. Bivariable multilevel robust Poisson regression analysis was done and all variables with a p-value of less than 0.25 in this analysis were considered for multivariable multilevel robust Poisson regression model(25, 26).

A multilevel mixed-effects Poisson regression model with robust error variance was fitted to identify determinants of stunting among children under five in SSA. This model was selected for two primary reasons. First, it directly estimates prevalence ratios, which are more interpretable and avoid the overestimation of association strength common with odds ratios from logistic regression when applied to common binary outcomes in cross-sectional data(31, 40). Second, its multilevel framework accounts for the hierarchical structure of the DHS data, where children are nested within households, and households within clusters, by including random intercepts at the household and cluster levels. Bivariable multilevel robust Poisson regression analysis was done and all variables with a p-value of less than 0.25 in this analysis were considered for multivariable multilevel robust Poisson regression model (31, 40).

Prior to model fitting, we assessed the key assumption of the Poisson regression model. A key assumption of this model is that observations are independent and that the mean and variance of the dependent variable are equal. Model adequacy was then confirmed through goodness-of-fit tests: the deviance statistic (151,692.69) and Pearson statistic (83,880.11) both yielded p-values of 1.000, indicating an excellent fit. The data had a mean of 0.306 and a variance of 0.294, producing a mean-to-variance ratio of approximately 1.04. This close alignment supports the equidispersion assumption and validates the use of the Poisson model. Based on these findings, we applied Poisson regression to examine the effects of predictors on stunting, while carefully monitoring for influential observations and residual patterns(41). Model fit was further assessed using Akaike (AIC) and Bayesian (BIC) Information Criteria, with lower values indicating better fit and parsimony.

In our analysis, five hierarchal models were fitted to select the model that best fits the data: null-model (a model with only outcome variable to assess the random variability in the intercept), model-I (a model with only individual-level explanatory variables), model-II (a model with only household-level explanatory variables), and model-III (a model with contextual-level factors) and model-IV (a model with only potential candidate variables from individual, household, and contextual-level factors). The " meglm" command in Stata was used to fit these models. ~~Sample weights (v005/1,000,000) were used to correct for over and under-sampling and to account for the complex survey design, enhancing the generalizability of the findings.~~

Random variability in stunting across clusters was assessed by intra-class correlation coefficient (ICC), explained variance or proportion change in variance (PCV), and median odds ratio (MOR). Akaike's information criteria (AIC), Bayesian information criteria (BIC), Log-likelihood (LL), and deviance (i.e.-2*LL) values were used for model comparison. The presence of multicollinearity between explanatory variables was checked using variance inflation factor values and the values for the included variables ranged from **1.01 to 1.95,** suggesting that there was no multi-collinearity. Finally, in the multivariable analysis, a p-value less than 0.05 and an adjusted prevalence ratio with the corresponding 95% confidence interval was used to identify the factors associated with stunting among ~~U-5~~ children under 5 years of age in 35 SSA countries.

Ethical considerations

Formatted: Font: 16 pt

For this study, we utilized ~~publicly available~~ Demographic and Health Survey (DHS) data from 35 sub-Saharan African ~~(SSA)~~ countries. The DHS survey procedures were approved by the ICF Institutional Review Board (IRB) and the respective host ~~country's~~country IRB~~. As a result~~; therefore, no additional ethical approval was required for this secondary analysis. ~~However, we obtained formal permission to access the data from ICF International (referenced as **AuthLetter_215093**). Furthermore, the~~ The dataset accessed ~~does not include any~~contain no identifiable participant information ~~about the individual participating in the study~~, ensuring confidentiality and privacy. Access to the data was formally authorized by the DHS program, which serve as the institutional custodian of the datasets, through an online request submitted at http://www.dhsprogram.com and supported by authorization letter AuthLetter_215093 on dated 01/19/2025.

Results

The study included and analyzed a weighted sample of 191,953 mothers with children aged 0 to 59 months. Of these children, 50.49% (96,907) were male, and 20.01% (42,185) were under 12 months old. More than half of the children, 55.34% (96,976), had been breastfed at some point but were no longer breastfeeding. Stunting was highly prevalent, affecting 21.98% (42,185) of children under 12 months, compared to older age groups. Among the included children, 65.63% (125,969) lived in rural areas, and ~~22.61% (43,396~~53.29% (25,499) were from households in the ~~poorest~~poor wealth index category. Additionally, 42.23% (81,068) of the children were from West African countries, and ~~28.60% (50,191~~31.51% (56,237) were delivered at home (Table~~-~~ 2).

Of the 191,953 mothers included in the study, 139,744 (72.80%) were between 25 and 49 years old, and 149,142(77.70%) were currently married. Over one-third of the mothers, 71,108(37.04%), had no formal education, while only 8,824(4.60%) had completed higher education. The majority, 110,643(57.64%), had their first child before the age of twenty, and 126,538 (65.92%) had access to mass media. Regarding nutritional status, 12,591(6.56%) of the mothers were underweight, while 91,612(47.73%) had a body mass index (BMI) within the normal range. Additionally, 26,149 (13.62%) were overweight, and 61,600(32.09%) were classified as obese

Table~~-~~ 2: Sociodemographic characteristic of the ~~under five~~ children under 5 years of age by stunting and their overall background status, in 35 SSA countries, 2011–2024 (n = **191,953**)

Formatted: Font: 17 pt

Formatted: Default Paragraph Font, Font: +Body (Calibri), 11 pt

Formatted: Font color: Light Blue

Formatted: Font color: Light Blue

Formatted: Font: 12 pt

Formatted: Font: 12 pt

Formatted: Font: 12 pt

Formatted: Font: 12 pt

Formatted: Font: 12 pt, Bold

Formatted: Font: 12 pt

Characteristics	Weighted Frequency (%)	Stunting
**Sex of the child**		
**Male**	96,907(50.49)	31,604(32.61)
**Female**	95,045(49.51)	26,642(28.03)
**Age of the child (months)**		
**Less than 12 months**	42,185(21.98)	7,156(16.96)
**12–23 months**	39,907(20.79)	13,492(33.81)
**24–35 months**	36,961(19.26)	14,225(38.49)
**36–47 months**	37,708(19.64)	13,108(34.76)
**48–59 months**	35,190(18.33)	10,264(29.17)
**Birth order**		
**First**	41,755(21.75)	11,640(27.88)
**2 to 4**	94,840(49.41)	27,615(29.12)
**5 or higher**	55,358(28.84)	18,991(34.31)
**Breastfeeding**		
**Ever breastfeed**	96,976(55.34)	34,331(35.40)
**Never breastfeed**	6,870(3.92)	2,149(31.28)
**Still breastfeeding**	71,397(40.74)	18,261(25.58)
**Perceived size at birth**		
**Large**	53,478(32.87)	15,044(28.13)
**Average**	81,999(50.40)	25,391(30.97)
**Small**	27,219(16.73)	10,537(38.71)
**Mothers' educational status**		
**No education**	71,108(37.04)	25,867(36.38)
**Primary**	61,015(31.79)	20,175(33.07)
**Secondary**	51,005(26.57)	11,385(22.32)
**Higher**	8,824(4.60)	818(9.28)
**Mother currently working**		
**Yes**	109,618(77,227)	33,980(31.00)
**No**	77,227(41.33)	22,379(28.98)
**Postnatal care visits**		
**Yes**	44,774(34.71)	11,787(26.33)
**No**	84,227(65.29)	25,394(30.15)

**Mother's age (years)**		
**15-24**	52,209(27.20)	16,514(31.63)
**25-49**	139,744(72.80)	41,732(29.86)
**Number of ANC visits**		
**No visit**	12,189(6.35)	4,919(40.36)
**1 to 3 visits**	39,646(20.65)	12,762(32.19)
**4 and above visit**	140,117(73.00)	40,565(28.95)
**Place of delivery**		
**Home**	~~50,191(28.60~~56,237(31.51)	~~20,132(40.11~~16,649(38.46)
**Health facility**	122,213(69.64)	~~33,747(27.~~38,187(61.54)
~~Others~~	~~3,095(1.76)~~	~~954(30.85)~~
**Marital status**		
**Single**	42,811(22.30)	12,499(29.20)
**Married**	149,142(77.70)	45,747(30.67)
**Mothers age at 1st birth**		
**Less than 20**	110,643(57.64)	36,334(32.84)
**20 and above**	81,309(42.36)	21,912(26.95)
**Maternal nutritional status**		
**Underweight**	12,591(6.56)	5,131(40.76)
**Normal weight**	91,612(47.73)	31,342(34.21)
**Overweight**	26,149(13.62)	6,036(23.08)
**Obese**	61,600(32.09)	15,736(25.55)
~~**Source of drinking water**~~		
~~Unimproved~~	~~62,833(32.74)~~	~~22,115(35.20)~~
~~Improved~~	~~129,105(67.26)~~	~~36,128(27.98)~~
~~**Type of toilet**~~		
~~Unimproved~~	~~58,136(40.43)~~	~~19,931(34.28)~~
~~Improved~~	~~85,649(59.57)~~	~~21,356(24.93)~~
**Household size**		
**Small**	16,665(8.68)	4,882(29.30)
**Medium**	12,723(6.63)	3,719(29.23)
**Large**	162,563(84.69)	49,644(30.54)
**Mass media exposure**		
**No**	65,415(34.08)	25,077(38.34)

**Yes**	126,538(65.92)	33,169(26.21)
~~**Cooking fuel**~~		
~~Unclean~~	~~157,830(83.80)~~	~~52,315(33.15)~~
~~Clean~~	~~30,507(16.20)~~	~~5,018(16.45)~~
**Wealth index**		
~~Poorest~~ Poor	84,032(43,~~396(22.61~~.78)	~~16,637(38.34~~25,499(53.29)
~~Poorer~~	~~40,635(21.17)~~	~~14,400(35.44)~~
**Middle**	38,~~450~~451(20.03)	11,~~958(31.10~~668(20.53)
~~Richer~~Rich	~~37,070(~~69,471(36,19~~.31~~)	~~9,793(26.42~~21,080(28.18)
~~Richest~~	~~32,400(16.88)~~	~~5,456(16.84)~~
**Place of residence**		
**Urban**	65,983(34.37)	14,229(21.57)
**Rural**	125,969(65.63)	44,017(34.94)
**Subregion**		
**West African Countries**	81,068(42.23)	22,671(27.97)
**East African Countries**	47,706(24.85)	14,833(31.09)
**Central African Countries**	37,024(19.29)	12,289(33.19)
**Southern African Countries**	26,154(13.63)	8,452(26,154)

Pooled prevalence of stunting among ~~under five~~ children under 5 years of age in sub–Saharan African

The pooled prevalence of stunting among children under ~~five~~5 years of age in 35 sub-Saharan African (SSA) countries was 29.~~72~~89% (95% CI: 26.~~55, 32.90~~63, 33.14%), with significant variation observed across countries~~.~~ (I^2 = 99.6, P-value = 0.000) (Fig 2). Gabon had the lowest stunting prevalence at 13.~~74~~91%, while Burundi reported the highest at 55.~~74~~80%. Among the 35 countries analyzed, ~~14~~15 had a stunting prevalence of 30% or higher among children under ~~five (see Fig. 1).~~5 years of age.

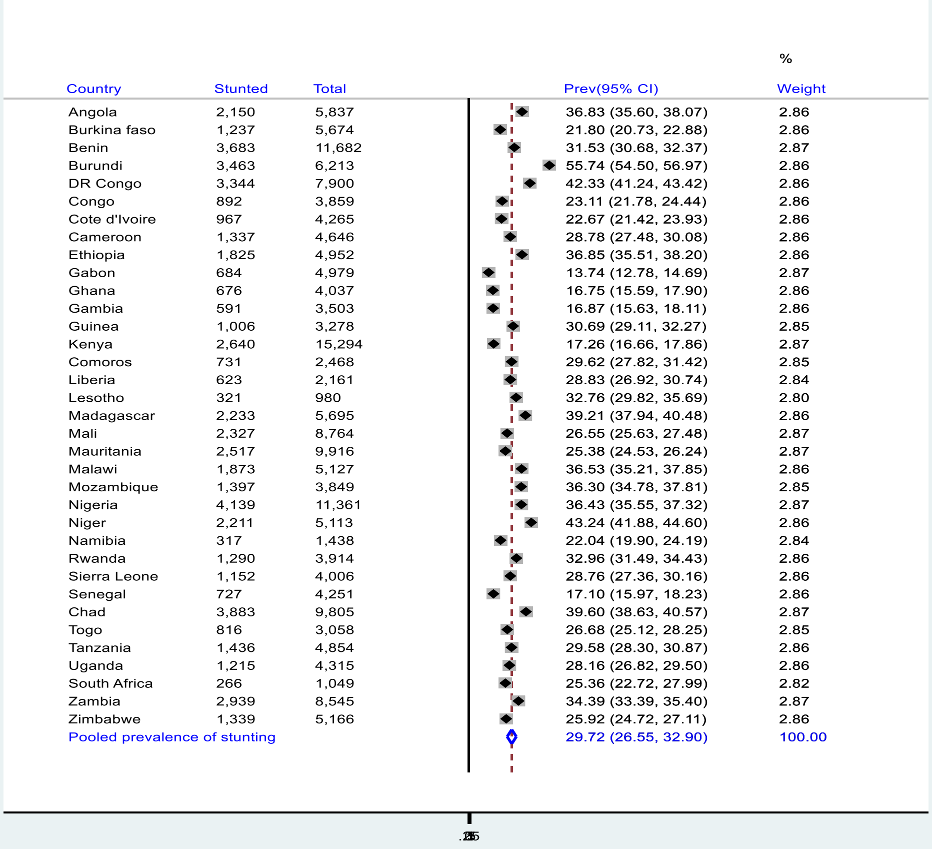

Handling heterogeneity

The random-effects model revealed considerable heterogeneity. To address this, sensitivity analysis, subgroup analysis, and meta-regression were conducted.

Sensitivity analysis

Sensitivity analysis was performed to evaluate the effect of individual country on the pooled estimated. When individual country was omitted, the pooled prevalence obtained was within the

95% CI of the overall pooled prevalence. This confirms the absence of single study impact on the overall pooled effect size. Therefore, from the random effects model, there were no country that excessively influence the overall pooled estimate of stunting (Fig. ~~1~~ 3).

Formatted: Font color: Light Blue

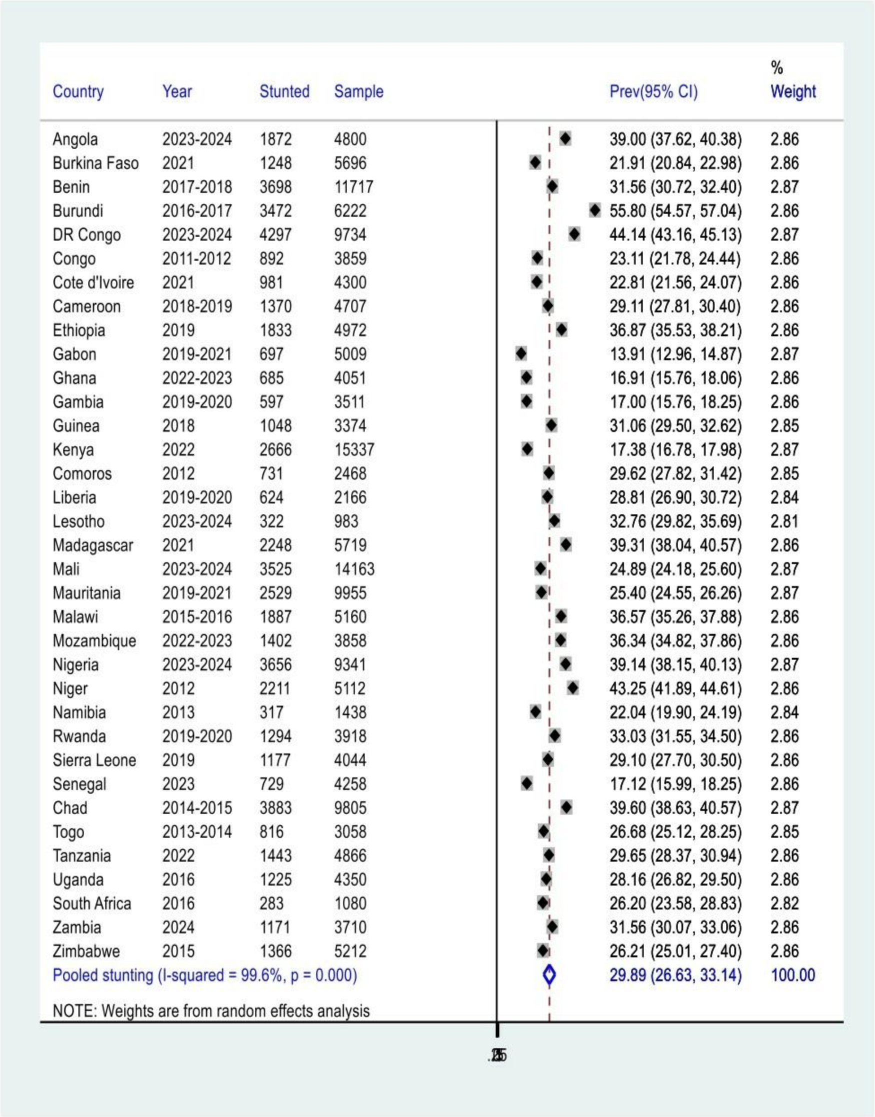

Figure 2: Forest plot of the country-level and pooled prevalence of stunting among ~~under five~~ children under 5 years of age across 35 SSA countries, 2011–2024

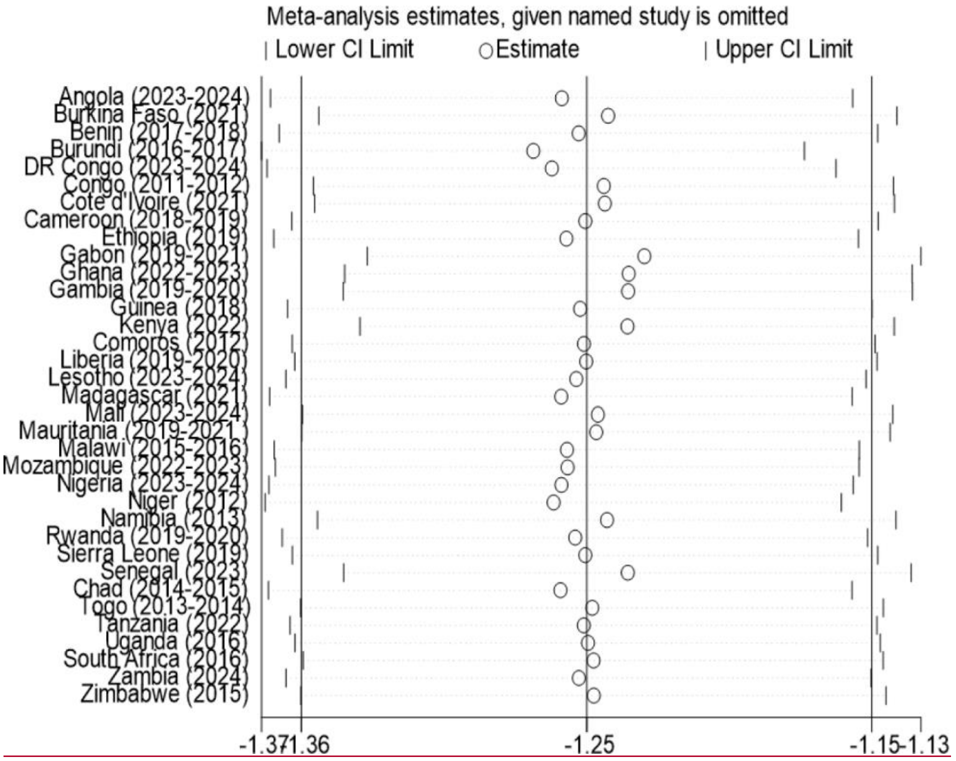

Figure 3: Sensitivity analysis plot forest plot of the country-level and pooled prevalence of stunting among children under 5 years of age across 35 SSA countries, 2011–2024

Sub-group analysis

Subgroup analyses were performed carried out by sub-region, and year of publication.

Stunting by Sub-Region

Subgroup analysis by sub-region indicated that Est African countries had the highest pooled prevalence of stunting among children under 5 years of age (33.73%, 95% CI: 24.19–43.26), followed by Southern African countries (31.37%, 95% CI: 27.20–35.54), Central African countries (29.98%, 95% CI: 18.24–41.71) and West African countries (26.83%, 95% CI: 22.89–30.77). Substantial heterogeneity was observed in East and Central Africa ($I^2 = 99.8\%$, $P = 0.00$), West Africa ($I^2 = 99.4\%$, $P = 0.00$), and Southern Africa ($I^2 = 98.1\%$, $P = 0.00$) (Fig 4).

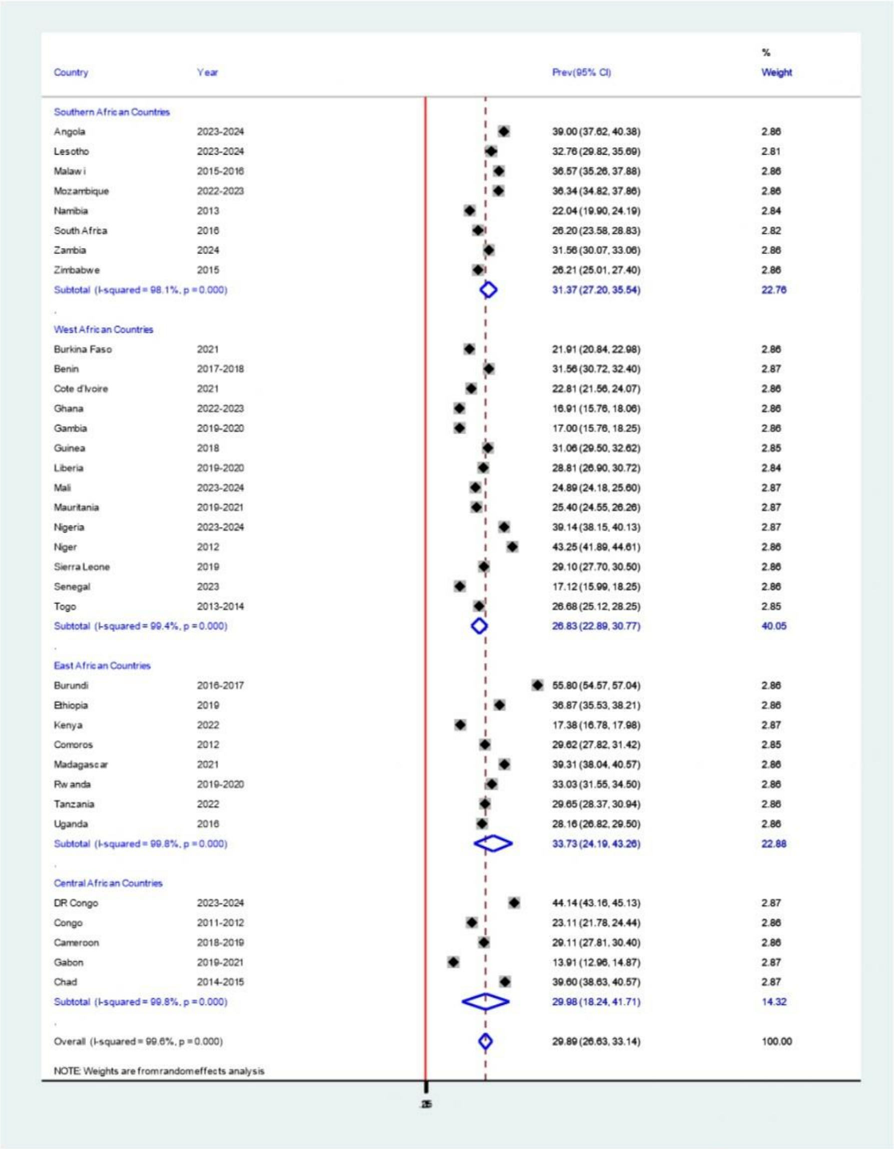

Figure 4: Forest plot showing the pooled prevalence of stunting among children under 5 years of age in sub-Saharan Africa, by subregion, based on a random-effects model analysis, 2011-2024.

Stunting by Year of Survey

Subgroup analysis by year of survey showed that the pooled prevalence of stunting was highest in 2011–2018 (32.32%, 95% CI: 27.19–37.44) and lowest in 2019–2024 (28.45%, 95% CI: 24.57–32.33). Both periods demonstrated significant heterogeneity (2011–2018: $I^2 = 99.5\%$, $p < 0.00$; 2020–2024: $I^2 = 99.6\%$, $p < 0.00$) (Fig 5).

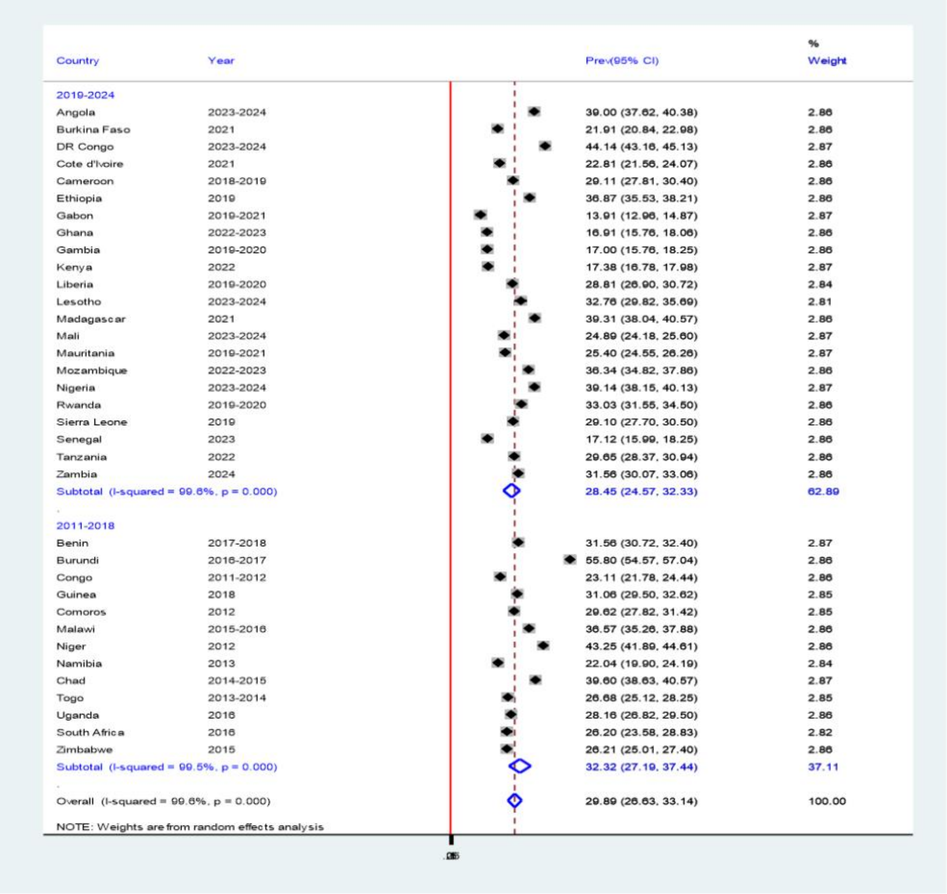

Figure 5: Forest plot showing the pooled prevalence of stunting among children under 5 years of age in sub-Saharan Africa, by survey year category, based on a random-effects model analysis, 2011-2024.

Meta-regression

A meta-regression was performed to assess whether the year of survey, sample size, country, or sub-region could explain the heterogeneity in stunting prevalence (Table 3). None of these variables were statistically significant predictors of heterogeneity (all p-values > 0.05). The high residual heterogeneity in the pooled prevalence of stunting suggests that other unmeasured factors are responsible for the variability across studies

Table 3: Meta-regression for the studies of pooled stunting among children under 5 years of age in 35 sub-Sharan Africa, 2011-2024.

Outcome categories	Variables	Coefficients	P-value	[95% conf. interval]
Pooled stunting among under five children	Year of Survey	0.99	0.68	0.96, 1.02
	Sample size	1.00	0.65	0.99, 1.00
	Countries	0.99	0.22	0.98, 1.00
	Sub-region	1.06	0.19	0.96, 1.17

Results of Random-Effects Analysis (Measures of Variation)

The ICC (Intraclass Correlation Coefficient) value from the null model revealed that only about 26.8% of the total variance in stunting among under five children is attributable to differences between clusters~~, while the remaining 73.2% was attributed to individual-level factors.~~. The remaining 73.2% of the variance reflects variation occurring within clusters, which encompasses individual, household, and broader contextual factors. This indicates that while cluster-level differences play a substantial role, the majority of the variability in stunting arises from determinants operating at multiple levels within clusters. In the final model (Model IV), the explained variance values demonstrated that around 47.2% of the total variation in the pooled prevalence of stunting among children under 5 years of age was influenced by the combined effects of individual level factors, household level factors and contextual factors-level factors.

Additionally, the presence of heterogeneity in stunting across clusters was supported by the MOR (Median Odds Ratio) values of 1.96 and 1.46 in the null and full models, respectively. This suggests that ~~under five~~ children under 5 years of age in clusters with higher pooled prevalence of stunting had approximately 1.96 times greater odds of stunting compared to those in clusters with lower levels of pooled prevalence of stunting. Model 4 was identified as the best-fitted model, as it exhibited the lowest AIC (Akaike Information Criterion), BIC (Bayesian Information Criterion), and deviance values (~~see~~ Table ~~3~~4).

Formatted: Font: 16 pt

Formatted: Space After: 10 pt

Formatted: Font color: Light Blue

Table ~~3~~ 4: Selection of most parsimonious model (measures of variation) at cluster or community level for stunting among ~~under five~~ children under 5 years of age in 35 SSA countries, 2011–2024

**Measure of variation**	**Models**				
	**Null-Model**	**Model I**	**Model II**	**Model III**	**Model IV**
Cluster-level variance (SE)	0.5(0.05)	0.15(0.001)	0.2(0.03)	0.3(0.04)	0.16(0.025)
Intra-class correlation (%) or VPC	26.8%	13.8%	12.7%	11.4%	10.2%
Explained variance (%) or PCV	***Reference***	70%	60%	40%	47.2%
Median odds ratio	1.96	1.44	1.5	1.68	1.46
Model summary					
Akaike's information criteria	245122.6	138191.7	169678.7	241377.5	**98254.67**
Bayesian information criteria	245153.1	138453.6	169807	241448.8	**98639.09**
Log-likelihood	-122558.28	-69068.872	-84826.365	-120681.75	**-49086.333**
Deviance	245116.55	138137.74	169652.73	241363.5	**98172.666**

VPC *= Variance Partitioning Coefficient,* ***PCV*** *= Proportion Change in Variance*

Determinants of stunting among ~~under five~~ children under 5 years of age in 35 sub–Saharan African Countries

Our analysis ~~found~~showed that the prevalence of stunting was 1.24 times higher among male children ~~(aPR:~~compared to female children (aPR = 1.24, 95% CI: 1.21~~, ~~–1.26~~) compared to female children's. Compared to~~). Using children under 12 months of age as the reference category, the prevalence of stunting was ~~significantly higher among older children:~~ 2.07 times higher among those aged 12–23 months (aPR = 2.07, 95% CI: 2.00~~, ~~–2.14~~) for those aged 12–23 months,~~). It was 2.41 times higher among children aged 24–35 months (aPR = 2.41, 95% CI: 2.32~~, ~~–2.50~~) for those aged 24–35 months,~~), 2.32 times higher among those aged 36–47 months (aPR = 2.32, 95% CI: 2.23~~, ~~–2.42~~) for those aged 36–47 months,~~), and 1.81 times higher ~~(~~among children aged 48–59 months (aPR = 1.81, 95% CI: 1.71~~, ~~–1.90) ~~for those aged 48–59 months (~~(Table~~ ~~4).

Children born in the fifth or higher birth order had an 8% higher prevalence of stunting ~~(aPR = 1.08, 95% CI: 1.03, 1.12)~~ compared to those born as first-order children~~. The prevalence of stunting was 16% higher~~ (aPR = 1.~~16~~08, 95% CI: 1.~~13, ~~03–1.~~19) among~~12). Using children perceived ~~by their mothers~~ as ~~having a small size~~large at birth ~~and 48~~as the reference category, the prevalence of stunting was 16% higher ~~(aPR = 1.48, 95% CI: 1.43, 1.52)~~ among those perceived as ~~having an~~ average size at birth~~.~~ (aPR = 1.16, 95% CI: 1.13–1.19) and 48% higher among those perceived as small size at birth (aPR = 1.48, 95% CI: 1.43–1.52). Compared to children whose mothers had ~~4 or more antenatal care (ANC) visits, the prevalence of stunting was 17% higher (aPR = 1.17, 95% CI: 1.13, 1.21) among children whose mothers had no ANC visits and 11% higher (aPR = 1.11, 95% CI: 1.09, 1.14) among those whose mothers had 1 to 3 ANC visits.~~

Formatted: Font: 12 pt
Formatted: Font: 12 pt
Formatted: Font: 12 pt
Formatted: Font: 12 pt
Formatted: Font: Bold, Italic
Formatted: Font: Italic
Formatted: Font: Bold, Italic
Formatted: Font: Italic
Formatted: Font: 16 pt
Formatted: Font: 16 pt
Formatted: Font: 16 pt
Formatted: Space After: 10 pt
Formatted: Font color: Blue
Formatted: Font color: Blue

Compared to children whose mothers had a higher level of formal education, the prevalence of stunting was 1.94 times higher (aPR = 1.94, 95% CI: 1.74, 2.18) among children whose mothers had no formal education, (aPR = 1.94, 95% CI: 1.74–2.18), 1.95 times higher (aPR = 1.95, 95% CI: 1.74, 2.19) among those whose mothers had only primary education, (aPR = 1.95, 95% CI: 1.74–2.19), and 1.68 times higher (aPR = 1.68, 95% CI: 1.51, 1.88) among those whose mothers had secondary education. The prevalence of stunting was 7% higher (aPR = 1.07, 95% CI: 1.04, 1.11) among children living in rural areas compared to their urban counterparts. Additionally, children whose mothers had no exposure to mass media (television, radio, or newspapers) showed an 11% higher prevalence of stunting (aPR = 1.11, 95% CI: 1.08, 1.13) than those whose mothers had access to media. (aPR = 1.68, 95% CI: 1.51–1.88).

Additionally, thisThis study revealed that, the prevalence of stunting was 1.03 times higher (aPR = 1.03, 95% CI: 1.07, 1.05) among children whose mothers did not attend postnatal visits, 1.09 times higher (aPR = 1.09, 95% CI: 1.06, 1.13) had a 3% higher prevalence of stunting compared to those whose mothers attended such visits (aPR = 1.03, 95% CI: 1.01–1.05). Using mothers aged 25 years and above as the reference category, the prevalence of stunting was 9% higher among children of mothers aged 15–24, 1.11 times higher (aPR = 1.11, 95% CI: 1.08, 1.14) years (aPR = 1.09, 95% CI: 1.06–1.13). Compared to children whose mothers had four or more antenatal care (ANC) visits, the prevalence of stunting was 17% higher among those whose mothers had no ANC visits (aPR = 1.17, 95% CI: 1.13–1.21) and 11% higher among those whose mothers had only one to three visits (aPR = 1.11, 95% CI: 1.09–1.14). In addition, children delivered at home, 1.04 times higher (aPR = 1.04, 95% CI: 1.02, 1.07) had an 11% higher prevalence of stunting compared to those delivered in health facilities (aPR = 1.11, 95% CI: 1.08–1.14). Furthermore, compared to children of married mothers, the prevalence of stunting was 1.04 times higher among children of single mothers, 1.02 times higher (aPR = 1.02, 95% CI: 1.03, 1.05) among children living in households using unimproved water sources, 1.04 times higher (aPR = 1.04, 95% CI: 1.04, 1.08) among children in large family households, and 1.25 times higher (aPR = 1.25, 95% CI: 1.18, 1.32) among children in households using unclean cooking fuels. (aPR = 1.04, 95% CI: 1.02–1.07).

Moreover, thisThis study revealed that, compared maternal BMI was significantly associated with childhood stunting. Compared to children whoseof mothers hadwith a normal body mass index (BMI),, the prevalence of stunting was 12% higher (aPR = 1.12, 95% CI: 1.08, 1.16) among

children of underweight mothers, (aPR = 1.12, 95% CI: 1.08–1.16). In contrast, the prevalence of stunting was 19% lower (aPR = 0.81, 95% CI: 0.77, 0.84) among thosechildren of overweight mothers, and compared to those of mothers with a normal BMI (aPR = 0.81, 95% CI: 0.77–0.84). Similarly, the prevalence of stunting was 12% lower (aPR = 0.88, 95% CI: 0.85, 0.90) among thosechildren of obese mothers. Furthermore, the study found that, compared to those of mothers with a normal BMI (aPR = 0.88, 95% CI: 0.85–0.90).

This study revealed that household size was significantly associated with childhood stunting. Compared to children from the richest households wealth indexsmaller households, the prevalence of stunting was 4% higher among children from large households (aPR = 1.04, 95% CI: 1.04–1.08). In addition, children whose mothers reported no exposure to mass media such as television, radio, or newspapers had an 11% higher prevalence of stunting compared to children whose mothers had some media exposure (aPR = 1.11, 95% CI: 1.08–1.13).

Moreover, compared to children from rich households, the prevalence of stunting was 31% higher among children from poor households (aPR = 1.31, 95% CI: 1.24, –1.38) among children from the poorest households wealth index, 29% higher (aPR = 1.29, 95% CI: 1.22, 1.35) among those from poorer households,). Similarly, the prevalence of stunting was 23% higher among children from middle wealth index households compared to those from rich households (aPR = 1.23, 95% CI: 1.17, –1.29) among those from middle-income households wealth index, and 22). Furthermore, children living in rural areas had a 7% higher prevalence of stunting compared to their urban counterparts (aPR = 1.2207, 95% CI: 1.16, 04–1.28) among those from richer households wealth index.11).

Table- 4: Bivariable and multivariable multilevel mixed-effects robust Poisson regression analysis of determinants of stunting among under five children under 5 years of age in 35 SSA countries, 2011–2024

Covariates	Stunting		cPR with 95%	aPR with 95%
	Yes	No		
**Sex of the child**				
Male	31,604(32.61)	65,303(67.39)	1.16(1.14, 1.18)*	***1.24(1.21, 1.26)*** **
Female	26,642(28.03)	68,402(71.97)	1	1

Formatted Table

**Age of the child (months)**				
Less than 12 months	7,156(16.96)	35,029(83.04)	1	1
12–23 months	13,492(33.81)	26,415(66.19)	1.99(1.93, 2.06) *	***2.07(2.00, 2.14) *****
24–35 months	14,225(38.49)	22,736(61.51)	2.26(2.19, 2.34) *	***2.41(2.32, 2.50) *****
36–47 months	13,108(34.76)	24,599(65.24)	2.05(1.98, 2.12) *	***2.32(2.23, 2.42) *****
48–59 months	10,264(29.17)	24,926(70.83)	1.72(1.66, 1.78) *	***1.81(1.71, 1.90) *****
**Birth order**				
First	11,640(27.88)	30,115(72.12)	1	1
2 to 4	27,615(29.12)	67,224(70.88)	1.04(1.01, 1.06) *	1.01(0.98, 1.05)
5 or higher	18,991(34.31)	36,367(65.69)	1.21(1.18, 1.24) *	***1.08(1.03, 1.12) *****
**Perceived size at birth**				
Large	15,044(28.13)	38,434(71.87)	1	1
Average	25,391(30.97)	56,608(69.03)	1.10(1.07, 1.12) *	***1.16(1.13, 1.19) *****
Small	10,537(38.71)	16,682(61.29)	1.37(1.34, 1.41) *	***1.48(1.43, 1.52) *****
**Mothers' educational status**				
No education	25,867(36.38)	45,241(63.62)	3.84(3.49, 4.22) *	***1.94(1.74, 2.18) *****
Primary	20,175(33.07)	40,839(66.93)	3.49(3.17, 3.84) *	***1.95(1.74, 2.19) *****
Secondary	11,385(22.32)	39,619(77.68)	2.37(2.15, 2.61) *	***1.68(1.51, 1.88) *****
Higher	818(9.28)	8,006(90.72)	1	1
**Postnatal care visits**				
Yes	11,787(26.33)	32,986(73.67)	1	1
No	25,394(30.15)	58,832(69.85)	1.14(1.11, 1.17) *	***1.03(1.07, 1.05) *****
**Mother's age (years)**				

15-24	16,514(31.63)	35,694(68.37)	1.05(1.03, 1.07) *	***1.09(1.06, 1.13) ** ***
25-49	41,732(29.86)	98,012(70.14)	1	1
**Number of ANC visits**				
No visit	4,919(40.36)	7,269(59.64)	1.36(1.32, 1.41) *	***1.17(1.13, 1.21) ** ***
1 to 3 visits	12,762(32.19)	26,884(67.81)	1.10(1.08, 1.12) *	***1.11(1.09, 1.14) ** ***
4 and above visit	40,565(28.95)	99,552(71.05)	1	1
**Place of delivery**				
Home	~~20,132(40.11~~16,649(38.46)	~~30,058(59.89~~32,199(26.68)	1.44(1.41, 1.47) *	***1.11(1.08, 1.14) ** ***
Health facility	~~33,747(27.~~38,186(61.54)	88,~~465(72.39~~466(73.32)	1	1
~~Others~~	~~954(30.85)~~	~~2,140(69.15)~~	~~1.11(1.04, 1.20) *~~	~~1.05(0.97, 1.13)~~
**Marital status**				
Single	12,499(29.20)	30,311(70.80)	0.94(0.92, 0.96) *	***1.04(1.02, 1.07) ** ***
Married	45,747(30.67)	103,39(69.33)	1	1
**Mothers age at 1st birth**				
Less than 20	36,334(32.84)	74,309(67.16)	1.20(1.18, 1.22) *	1(0.97, 1.02)
20 and above	21,912(26.95)	59,397(73.05)	1	1
**Maternal nutritional status**				
Underweight	5,131(40.76)	7,459(59.24)	1.19(1.15, 1.22) *	***1.12(1.08, 1.16) ** ***
Normal weight	31,342(34.21)	60,270(65.79)	1	1
Overweight	6,036(23.08)	20,113(76.92)	0.68(0.66 0.70) *	***0.81(0.77, 0.84) ** ***
Obese	15,736(25.55)	45,863(74.45)	0.75(0.73, 0.77) *	***0.88(0.85, 0.90) ** ***
~~**Source of drinking water**~~				
~~Unimproved~~	~~22,115(35.20)~~	~~40,718(64.80)~~	~~1.25(1.22, 1.27) *~~	~~***1.02(1.03, 1.05) ** ***~~
~~Improved~~	~~36,128(27.98)~~	~~92,977(72.02)~~	~~1~~	~~1~~

Formatted Table

**Household size**				
Small	4,882(29.30)	11,782(70.70)	1	1
Medium	3,719(29.23)	9,004(70.77)	1(0.95, 1.04)	0.99(0.94, 1.05)
Large	49,644(30.54)	112,919(69.46)	1.04(1.01, 1.07) *	***1.04(1.04, 1.08)*** **
**Mass media exposure**				
No	25,077(38.34)	40,337(61.66)	1.44(1.41, 1.47) *	***1.11(1.08, 1.13)*** **
Yes	33,169(26.21)	93,368(73.79)	1	1
~~**Cooking fuel**~~				
~~Unclean~~	~~52,315(33.15)~~	~~105,515(66.85)~~	~~1.99(1.90, 2.08) *~~	~~***1.25(1.18, 1.32)*** **~~
~~Clean~~	~~5,018(16.45)~~	~~25,489(83.55)~~	~~1~~	~~1~~
**Wealth index**				
~~Poorest~~ Poor	16,637(38.34)	~~26,759(61.66~~52,993(39.63)	2.26(2.17, 2.36) *	***1.31(1.24, 1.38)*** **
~~Poorer~~	~~14,400(35.44)~~	~~26,234(64.560)~~	~~2.09(2.00, 2.18) *~~	~~***1.29(1.22, 1.35)*** **~~
Middle	11,958(31.10)	26,492(~~68.90~~19.81)	1.83(1.75, 1.91) *	***1.23(1.17, 1.29)*** **
Richer	9,793(26.42)	~~27,277(73.58~~54,221(40.55)	~~1.55(1.49, 1.62)~~ *1	~~***1.22(1.16, 1.28)***~~ ***1
~~Richest~~	~~5,456(16.84)~~	~~26,943(83.16)~~	~~1~~	~~1~~
**Place of residence**				
Urban	14,229(21.57)	51,754(78.43)	1	1
Rural	44,017(34.94)	81,952(65.06)	1.61(1.56, 1.66) *	***1.07(1.04, 1.11)*** **

*__cPR__: Crude Prevalence Ratio, __aPR__: Adjusted Prevalence Ratio, *: Significant in crude prevalence ratio, **: Significant in adjusted prevalence ratio*

Discussion

Reducing stunting in sub-Saharan Africa (SSA) continues to pose a significant challenge, yet various programmatic initiatives are being introduced to drive progress. The World Bank's "All Hands-on Deck" initiative advocates for a multisectoral strategy, involving sectors such as agriculture, education, social protection, and water, sanitation, and hygiene (WASH)~~(21).~~(42).

Similarly, UNICEF's programs prioritize maternal, infant, and young child nutrition, micronutrient supplementation, the management of severe acute malnutrition, nutrition in emergencies, and enhancing water and sanitation at the community level~~(22).~~(43). Despite these efforts, the burden of stunting remains high in sub-Saharan African countries. These integrated approaches are specifically designed to accelerate stunting reduction in SSA, with a particular emphasis on East Africa, where the challenge persists~~(11)~~(4).

This study analyzed pooled data from nationally representative surveys conducted across 35 sub-Saharan African (SSA) countries between 2011 and 2024. The findings revealed that the overall pooled prevalence of stunting among ~~under five~~ children under 5 years of age in SSA was 29.~~72~~89% (95% CI: 26.~~55, 32.90~~63, 33.14%). The lowest stunting prevalence rate was recorded in Gabon at 13.74%, while the highest prevalence of stunting was observed in Burundi at 55.74%. Among the 35 countries analyzed, 14 had a stunting prevalence of 30% or higher among children under ~~five.~~5 years of age. This finding is consistent with World Health Organization prevalence threshold or cut-off values for public health significance in which prevalence of stunting 20 to <30% is high and greater than or equal to 30% is very high~~(33).~~(44). Additionally, the pooled prevalence of stunting aligns with findings from studies carried out in 65 Low- and Middle-Income Countries(29.0%)~~(34)~~(45), 62 LMICs worldwide(29.1%)~~(35)~~(46), Tajikistan (27%)~~(36)~~(47), and Tanzania (28%)~~(37).~~(48).

However, the observed prevalence is lower than that reported in previous studies conducted in Ethiopia (33.58%)~~(38), Low- and Middle-Income Countries(38.8%)(39), East Africa(33.3%, 39%)(40, 41), Sub-Saharan Africa(33.2%)(41), Pakistan(44%)(42), Ethiopia(37%)(43), Kenya (39%)(44), Sub-Saharan Africa(35%)(11), Tanzania(35%)(45), Vietnam (44%)(46), Chitwan (37.3%)(47), and Belahara VDC (37%)(48),. Additionally, this finding was higher than the prevalence of stunting reported in studies conducted in China (4.4%)(49), Bangladesh (25%)(50), Bhutan(21%)(51), and UNICEF-WHO-WB Joint Child Malnutrition Estimates(22.3%)(52)~~(49), Low- and Middle-Income Countries(38.8%)(50), East Africa(33.3%, 39%)(51, 52), Sub-Saharan Africa(33.2%)(52), Pakistan(44%)(53), Ethiopia(37%)(54), Kenya (39%)(55), Sub-Saharan Africa(35%)(4), Tanzania(35%)(56), Vietnam (44%)(57), Chitwan (37.3%)(58), and Belahara VDC (37%)(59),. Additionally, this finding was higher than the prevalence of stunting reported in

studies conducted in China (4.4%)(60), Bangladesh (25%)(61), Bhutan(21%)(62), and UNICEF-WHO-WB Joint Child Malnutrition Estimates(22.3%)(63).

A potential explanation for this finding could be the differences in nutritional practices, sociocultural and environmental factors across the countries. Variations in the scope of the studies, the year of research, population characteristics, and methodologies employed might have contributed to the observed differences. For instance, earlier studies were limited in scope, focusing on individual countries and utilizing diverse analytical approaches~~(11, 53-57)~~(4, 64-68) and some studies also included a restricted number of countries and population characteristics by ages ~~(11). Moreover, another main reason could be attributed to the prolonged malnutrition among children~~(4). Moreover, another main reason could be attributed to the prolonged malnutrition among children under 5 years of age in Sub-Saharan Africa (SSA), stemming from persistent poverty in the region~~(58).~~(69). Furthermore, the limited economic resources of many African nations can hinder access to sufficient and nutritious food, contributing to the prevalence of stunting(~~59~~70).

Although the pooled prevalence of stunting among children under 5 years of age in 35 SSA countries was estimated at 29.89% (95% CI: 26.63, 33.14%), the exceptional high heterogeneity ($I^2 = 99.6\%$, $p < 0.000$) underscores substantial contextual variation across countries. This variation is not statistical only but also reflects meaningful differences in underlying determinants, health system capacity, and sociopolitical environments. Subgroup analysis revealed that East African countries had highest stunting burden (33.73%), while West African showed comparatively lower prevalence (26.83%), suggesting region drivers such as climate vulnerability, food system resilience, or maternal education levels.

However, meta-regression indicated that broad variables like survey year, sample size, country, and sub-region did not significantly explain this heterogeneity (all p-values > 0.05), pointing to the influence of more unmeasured factors such as governance quality, conflict exposure, or local nutrition policies. These findings highlight the limitations of the relying solely on pooled estimate for policy guidance and emphasizes the need for country level diagnostics to identify context specific risk factors. Future research should explore these modifiers systematically to inform tailored interventions rather than one size fits all strategies.

Field Code Changed

Field Code Changed

Our findings revealed that stunting among children under ~~five~~5 years of age in sub-Saharan African countries is shaped by a mix of individual, household-level, and contextual factors. ~~In this study, the study identified the sex of the child as a~~One significant predictor was the sex of ~~stunting~~the child, with male children ~~being~~ more likely to experience stunting than ~~their female counterparts.~~females. This finding aligns with a study conducted in Sub-Saharan African (SSA) countries, as well as studies conducted in Zambia and Ethiopia~~(11, 56, 60, 61)~~(4, 67, 71, 72)~~. A potential explanation for this could be that male child, particularly in developing regions like SSA, are more susceptible to childhood illnesses, such as acute respiratory infections and diarrheal diseases, compared to female children, although the biological mechanisms behind this remain unclear(62, 63). Conversely, a study in Ethiopia found that female children had higher odds of being stunted than males(64). This discrepancy may be attributed to socio-cultural practices, such as preferential treatment of male children in terms of nutrition and care, especially during illness, in certain societies(65, 66).~~

~~The prevalence of stunting was significantly higher among older children in the age groups of 12-23 months, 24-35 months, 36-47 months, and 48-59 months compared to children under 12 months of age. This finding indicates that the risk of stunting rises as a child grows older. This aligns with studies conducted in Ethiopia(38, 43, 56, 67), Somalia(68), Rwanda(69), and Zimbabwe(70). A potential explanation for this trend is that caregiving practices often diminish as children transition from infancy to older ages and begin consuming adult foods. Additionally, the introduction of complementary foods or the discontinuation of breastfeeding can reduce the energy and nutritional density of their diet, potentially compromising their overall health. Meeting the nutritional needs of this age group is crucial to fostering healthy growth and development. Furthermore, without breastmilk and its immune-boosting components, children lose a significant level of protection against illnesses(50).~~

~~Children born in the fifth or higher birth order had an 8% higher prevalence of stunting compared to those born as first-order children. This suggests that a higher birth order is associated with an increased likelihood of a child being stunted, independent of the presence of other children, maternal factors, and socioeconomic conditions.~~ . The consistency of this association across diverse setting suggests a robust pattern that may reflect biological vulnerability, differential care practices, or gendered exposure to environmental risks. Evidence from longitudinal studies in sub-

Saharan Africa indicates that male children are more prone to growth faltering during the first year of life(73). However, findings from certain interventions program such as nutritional supplementation and maternal education initiatives suggest that these sex-based disparities in stunting may diminish over time(74).

This indicates that while male children may be biologically predisposed to stunting, contextual factors such as household factors, caregiving practices, and health system responsiveness can modify these risks. What remain uncertain is whether the sex differential is primarily driven by biological susceptibility or by modifiable social determinants. Future research, particularly longitudinal interventions studies, is needed to clarify and separate the overlapping biological and socio-behavioral pathways, in order to determine whether targeted strategies for boys could achieve greater reductions in stunting prevalence.

The prevalence of stunting was significantly higher among older children in the age groups of 12–23 months, 24–35 months, 36–47 months, and 48–59 months compared to children under 12 months of age. This finding indicates that the risk of stunting rises as a child grows older. Similar age-related patterns have been reported in studies conducted in Ethiopia(49, 54, 67, 75), Somalia(76), Rwanda(77), and Zimbabwe(78), suggesting that growth faltering often becomes more pronounced after infancy.

A prospective birth cohort studies have demonstrated that growth deficit often accelerates between 6 and 24 months, reflecting both biological vulnerability and the transition to inadequate diets and increased exposure to infections(79, 80). Interventions trial have further shown that timely interventions during this critical window period can reduce stunting prevalence(81). The novel contribution of this study is the documentation of this established association within our specific, previously less investigated population, thereby strengthening the geographical evidence base. What remain uncertain, however is, the extent to which age-related differences are modifiable through targeted interventions and whether the persistency of stunting in to later childhood reflects irreversible early childhood damage or ongoing exposure to adverse conditions.

Children born in the fifth or higher birth order had an 8% higher prevalence of stunting compared to those born as first-order children. This finding aligns with earlier studies that have examined child nutritional status, considering birth order as a significant confounding factor~~(71-74)~~(82-85)~~.~~

A potential explanation for this association could be that higher-order births are more likely to be unintended, leading to reduced parental attention and care. This may result in decreased access to antenatal and postnatal care, as well as fewer child checkups, as birth order increases(75, 76).

The perceived size of a baby at birth showed a significant association with stunting status, with children born small and average size more likely to be stunted compared to those born large. This finding is consistent with studies conducted in Africa and other developing countries, such as Indonesia and Ethiopia(77-79) as well as Nepal(80), Bangladesh(81) and Ethiopia(82). The possible explanation for this could be that small birth size may reflect underlying conditions such as preterm birth, inadequate maternal nutrition, or illness during pregnancy, all of which can restrict a child's growth and development (83). Additionally, small birth size is linked to childhood illnesses like diarrhea and acute febrile infections, which can further contribute to stunting(84).

In this study, maternal education level was significantly associated with the stunting status of children under five. . Cohort studies have shown that later born children often experience reduced access to household resources, shorter breastfeeding duration, and greater exposure to infections, which collectively contribute to growth faltering(86, 87). Interventions study also demonstrated that empowering mothers to space births and manage household resources can mitigate the nutritional advantages faced by higher order children(88, 89). However, the extent to which birth order effects are biologically driven versus socially mediated, and whether targeted interventions such as birth spacing programs or nutrition support for larger family can fully offset these risks were remains uncertain.

The perceived size of a baby at birth showed a significant association with stunting status, with children born small and average size more likely to be stunted compared to those born large. This finding is consistent with studies conducted in Africa and other developing countries, such as Indonesia and Ethiopia(90-92) as well as Nepal(93), Bangladesh(94) and Ethiopia(95). Cohort studies conducted in Ethiopia and Malawi have shown that children born small remain at elevated risk of stunting well into the first two years of life, even after accounting for postnatal feeding practices(96). Another interventional study on maternal nutrition supplementation programs in Ghana and Tanzania, further confirmed that improving maternal diet and antenatal care can reduce the incidence of the low birth weight and subsequently lower stunting prevalence(97, 98). However, uncertainties remain regarding the extent to which postnatal interventions (e.g.,

complementary feeding programs) can fully offset the disadvantage of being born small, suggesting that prevention strategies must prioritize maternal health and antenatal care.

The study revealed that compared to children whose mothers had a higher level of formal education, the prevalence of stunting was 1.94 times higher among children whose mothers had no formal education, 1.95 times higher among those whose mothers had only primary education, and 1.68 times higher among those whose mothers had secondary education. This showed that, as maternal education increased from no education to primary, secondary, and higher levels, the prevalence of stunting among children decreased. This finding is supported by a systematic review of studies published worldwide between 2004 and 2014, as well as by research conducted in Ethiopia, Zambia and Nepal(11, 85-88)(4, 99-102). This association can be explained by the fact that educated mothers are more likely to have knowledge about proper child feeding practices and understand the importance of nutrition for their children's growth and development(89). Furthermore, maternal education often leads to improved health literacy and higher household income, both of which positively influence the health and nutritional status of children(90).. Previous intervention studies have established that maternal education as protective factors. For example, community based nutritional programs in Uganda and Tanzania show that incorporating maternal literacy and health educations components resulted in significantly lower stunting prevalence versus control group(103, 104). However, maternal nutritional counselling can only compensate for the disadvantage of low maternal education in resource constrained setting remain uncertain. Future research should explore whether combining educational empowerment with direct nutrition interventions yields synergistic effects in reducing stunting.

Study revealed that, postnatal visit associated with child stunting. Mothers who did not attend postnatal care services were 1.03 more likely to have a stunted child compared to those who did attend postnatal care. This finding consistent with study conducted in Ethiopia(91). This could be because, during postnatal care visits, mothers receive nutrition education from healthcare providers about optimal child feeding practices, which can be implemented to support the baby's growth. This guidance may positively influence the physical development of the child.

Children born to mothers aged 15–24 were more likely to experience higher levels of stunting compared to those born to mothers aged 25-49. This finding consistent with study conducted in Ethiopia(92). (105). This may be due to the fact that babies born to younger mothers are more

prone to preterm birth and low birth weight, which can increase the risk of neonatal infections and malnutrition, including stunting~~(93). Additionally, younger mothers, who are still in their own growth phase, may struggle to provide adequate nutritional care for their children, potentially hindering their growth and development and leading to malnutrition and other growth-related challenges(94).~~(106). Another study also showed that younger maternal age is often associated with limited caregiving experience, reduced autonomy in household decision-making, and lower access to health and nutrition services contributing higher stunting prevalence(107). However further research needs maternal age itself act as independent risk factors versus being mediated by socioeconomic status, maternal education or health service utilization.

Mothers' antenatal care (ANC) visits were strongly associated with child stunting. Children whose mothers did not attend any antenatal follow-up and 1 to 3 ANC visits more likely to be stunted compared to those whose mothers had four or more ANC visits. This finding is supported by a study conducted in Ethiopia~~(95-97)~~(108-110)~~, Zambia(98), Latin America(99, 100), and Bangladesh(101). This may be attributed to the fact that antenatal care (ANC) programs are structured to identify high-risk mothers and deliver nutritional and educational support, such as advice on food hygiene, diet, and lifestyle. These initiatives target key factors that play a significant role in enhancing child nutrition. ANC programs are advocated as an effective approach to lowering the prevalence of low birth weight, and evidence highlighting their effectiveness in reducing adverse pregnancy outcomes in developing countries is steadily increasing(102, 103)~~, Zambia(111), Latin America(112, 113), and Bangladesh(114). This may be attributed to the fact that antenatal care (ANC) programs are structured to identify high-risk mothers and deliver nutritional and educational support, such as advice on food hygiene, diet, and lifestyle. These initiatives target key factors that play a significant role in enhancing child nutrition. ANC programs are advocated as an effective approach to lowering the prevalence of low birth weight, and evidence highlighting their effectiveness in reducing adverse pregnancy outcomes in developing countries is steadily increasing(115, 116). However, barriers such as inequitable health system coverage, geographical isolation and weak governance in service delivery restrict women access to timely and quality antenatal care services(117, 118).

In this study, the place of delivery showed a significant association with child stunting. Specifically, children born at home were more likely to be stunted compared to those born at health

facilities. This finding aligns with a study conducted in Ethiopia(11, 104).(4, 119). A possible explanation for this association is that mothers who deliver at home are less likely to receive proper postnatal care, including vaccinations, which are critical for the healthy growth and development of children. Vaccinations help prevent several vaccine-preventable diseases and ensure children receive essential nutrients, contributing to better overall health outcomes(105120).

Other factors associated with stunting were single marital status and household with large family size. Child stunting higher among mother with single marital status.The study revealed that postnatal care visits were associated with childhood stunting. Mothers who did not attend postnatal care services were 1.03 times more likely to have a stunted child compared to those who did. This finding is consistent with a study conducted in Ethiopia(121). Study confirmed that strengthening maternal contact with health systems during the postnatal period contributes to improved child growth outcomes(122). However, limited attendance at postnatal care is not solely a matter of maternal choice but reflects broader structural determinants. Barriers such as poverty, inequitable health system coverage, weak governance in service delivery and in some context conflict or geographical isolation restrict women access to timely and quality postnatal services(117, 118).

Addressing stunting therefore requires structural interventions such as equitable health infrastructure, improving governance and accountability in maternal and child health programs, and strengthening social protection alongside efforts to promote household-level health-seeking behaviors. Additionally, the extent to which postnatal care independently reduces stunting versus acting synergistically with other maternal and child health interventions such as antenatal care, breastfeeding promotion, and community nutrition programs remains uncertain.

Another factor associated with stunting was marital status. In the study, the prevalence of child stunting was higher among children of single mothers than among children with married mothers. This finding consistent with previous study(106).(123). The possible explanation may be attributed to socioeconomic challenges, such as limited financial resources, reduced access to healthcare, and lower social support. Single mothers often face greater difficulties in providing adequate nutrition and care for their children due to these constraints. Additionally, the stress and time demands associated with single parenthood may further hinder their ability to ensure optimal child-rearing conditions, contributing to poorer growth outcomes. These factors collectively increase the risk of stunting in children of single mothers(107124).

Field Code Changed
Field Code Changed
Field Code Changed
Field Code Changed

This study found that, maternal body mass index associated with stunting. Compared to children whose mothers had a normal body mass index (BMI), the prevalence of stunting was higher among children of underweight mothers and lower among those of overweight mothers, and obese mothers. These findings were consistent with previous study conducted in low-income and middle-income countries(108), Ethiopia(109, 110), Bangladesh(111) and Nigeria(10). The likely explanation for this association is that the higher prevalence of stunting among children of underweight mothers may be linked to poor maternal nutrition, which can lead to inadequate nutrient transfer during pregnancy and lactation, affecting the child's growth. Conversely, lower stunting rates among children of overweight or obese mothers might be attributed to better access to food and higher caloric intake, though this does not necessarily equate to optimal nutrition. Overweight and obese mothers may also have greater resources and socioeconomic advantages, contributing to better child health outcomes. However, these findings highlight the complex relationship between maternal BMI and child growth, emphasizing the need for balanced maternal nutrition to ensure healthy development(10, 112).

In this analysis, the use of unimproved water sources was positively associated with child stunting. Children from households without access to improved water sources were more likely to be stunted compared to those from households with access to such facilities. These findings are consistent with a systematic review and meta-analysis of 171 Demographic and Health Surveys (DHS) from 70 low- and middle-income countries conducted between 1984 and 2007, as well as a study conducted in sub-Saharan Africa and Ethiopia(11, 113, 114). The likely explanation for this association is that poor sanitation and the use of low-quality drinking water, which may be contaminated with pathogens like Escherichia coli, can negatively impact children's health and nutritional status. This can occur through mechanisms such as repeated episodes of diarrhea, parasitic infections, environmental enteropathy, and other factors that hinder nutrient absorption and utilization(115).

This study found that maternal body mass index was associated with childhood stunting. Compared to children whose mothers had a normal body mass index (BMI), the prevalence of stunting was higher among children of underweight mothers and lower among those of overweight mothers, and obese mothers. These findings were consistent with previous study conducted in low-income and middle-income countries(125), Ethiopia(126, 127), Bangladesh(128) and Nigeria(6).

While poor maternal nutrition likely contributes to inadequate nutrient transfer during pregnancy and lactation, resulting in impaired child growth, the relationship between maternal BMI and stunting extends beyond individual level behaviors. Lower stunting rates among children of overweight or obese mothers may reflect not only higher caloric intake but also broader socioeconomic advantages, such as improved household food security, access to diverse diets, and greater resilience to shocks(129).

These maternal outcomes are shaped by structural determinants, including poverty, food system functioning, governance and conflict that influence both maternal nutrition and child health. For instance, household insecure regions or conflict affected setting may face systematic barriers to adequate nutrition regardless of maternal practices(129, 130). Thus, while balanced maternal nutrition remains critical, these findings underscore the needs to situate maternal and household factors within wider structural contexts. Addressing stunting requires not only promoting individual behavior change but also tackling systematic inequities through poverty reduction, strengthening food systems, improving governance and ensuring stability.

This study revealed that child stunting high among household with large family size. This finding similar with previous study conducted in sub-Saharan Africa~~(116)~~(131), and Ethiopia~~(117, 118).~~(132, 133). This may be due to the fact that higher prevalence of child stunting in households with large family sizes may be due to resource dilution, where limited financial and food resources are spread thinly among many family members, reducing the quantity and quality of nutrition available for each child. Larger families may also face challenges in providing adequate healthcare and sanitation, further exacerbating the risk of stunting. Additionally, caregivers in large families may have less time and capacity to ensure proper feeding practices and childcare, contributing to poorer growth outcomes(~~119~~134).

The results of this study also revealed that mothers' exposure to mass media was significantly associated with high prevalence of child stunting. Children whose mothers had not media exposure were more likely to be stunted compared to those whose mothers had exposure to media. This finding consistent with studies conducted in Pakistan, Bangladesh and Ethiopia~~(11, 120, 121)~~(4, 135, 136)~~. This can be attributed to the influential role of media in shaping health and nutrition-related behaviors, attitudes, and practices. Additionally, media plays a vital role in promoting~~

Field Code Changed

Field Code Changed

sociocultural and economic development, which can contribute to improved nutritional outcomes(122).

Our study revealed that positive association between stunting and household fuel type. Stunting was higher among children in households using unclean cooking fuels. This finding similar with previous systematic review and meta-analysis study(123). The possible explanation for this association may be due to increased exposure to indoor air pollution. This pollution can lead to respiratory infections and chronic illnesses in children, impairing their nutrient absorption and overall growth. Additionally, households relying on unclean fuels often face socioeconomic disadvantages, including limited access to nutritious food and healthcare, further exacerbating the risk of stunting. The combination of environmental and socioeconomic factors likely contributes to the higher prevalence of stunting in such households(124).

Our study revealed . Media plays a vital role in promoting sociocultural and economic development, which can contribute to improved nutritional outcomes(137). However, access to mass media is shaped by systemic issues such as poverty, inequitable information and communication infrastructures, governance of public health messaging, and in some contexts, conflict that limits dissemination of reliable information(138). These structural barriers constrain households' ability to access knowledge about nutrition, sanitation, and child health, thereby reinforcing cycles of stunting(139). Therefore, addressing these challenges require system level interventions.

Our study revealed a significant positive association between the household wealth index and child stunting. Compared to children from the richest household wealth index, child from the poorest, poorer, poor and middle and richer households' wealth index had higher odds of being stunted. TheseWhile these findings align with a systematic review and meta-analysis conducted in Africa between 2000 and 2013 and study conducted in Sub-Saharan Africa(11, 125)(4, 140), as well as studies from low-income countries such as Nepal, Zambia, and Nigeria(87, 126, 127)(101, 141, 142). This association is likely due to the fact that richest households are more likely to have access to food security and dietary diversity(128). Additionally, wealthier households tend to exhibit better health-seeking behaviors, such as promptly visiting health facilities when their children fall ill, which positively contributes to children's health, well-being, and proper growth and development(129).

, the implications extend beyond household-level economic status(143). Household wealth reflects broader structural determinants including entrenched poverty, inequitable food systems, weak governance, and in some settings, the destabilizing effects of conflict that constrain access to adequate nutrition, health services, and safe environments for children(144, 145). Thus, stunting should be understood not only as a consequence of limited household resources but also as a manifestation of systemic inequities.

Furthermore, contextual-level variables specifically, place of residence were significantly associated with child stunting. Children from rural areas had higher prevalence of being stunted compared to their urban counterparts. A finding supported by studies conducted in Pakistan and Ethiopia(130, 131).(146, 147). This may be attributed to the better nutritional status of urban children, which stems from improved maternal prenatal and postnatal care, higher-quality complementary feeding practices, and better immunization coverage. Additionally, urban children benefit from advantages such as family employment opportunities, stronger social and family networks, and greater access to healthcare and social services, all of which contribute to their improved nutritional status compared to rural children(132148).

Field Code Changed

Field Code Changed

Strength and limitation of the study

Formatted: Font: 16 pt

TheThis study utilized a recent large dataset, nationally representative sampledataset encompassing 191,953 children under five from 35 countries from Subsub-Saharan African (SSA) countries conductedsurveyed between 2011- and 2024, which enhances its. The large sample size substantially enhanced the statistical power to detect true effect sizes and improvesimproved the generalizabilitygeneratability of the findings. The study employed the application of advanced across diverse settings. Advanced statistical methods-, including multilevel mixed-effects Poisson regression with robust variance, were employed to strengthen the analysis. By combining and account for the hierarchical nature of the DHS data from 35 SSA. By pooling data across countries, the study estimated the pooled prevalence of stunting among childrenand its determinants, revealing significant and concerning heterogeneity between countries. Additionally, the study included that persisted even after subgroup and sensitivity analyses. Importantly, the inclusion of key maternal and child health variables such as antenatal care (ANC) and postnatal care (PNC) visits, and maternal nutritional status (e.g., body mass index (BMI and height), which were

Formatted: fontstyle21, Font: Times New Roman, 12 pt

~~excluded from the analysis~~) addressed gaps overlooked in ~~the~~ previous ~~study(11).~~studies(4), thereby providing a more comprehensive understanding of stunting determinants.

~~However, health~~Nonetheless, several limitations should be acknowledged. Health system variables, including health insurance coverage, the ~~number~~availability and type of health facilities, ~~as well as~~and broader country-level contextual factors ~~like~~such as political stability, were not assessed~~, representing additional limitations of the study.~~. Furthermore, ~~due to~~ the cross-sectional design of the ~~survey,~~DHS surveys precludes establishing ~~a~~ causal ~~relationship~~relationships between ~~the~~ independent and dependent variables. While significant associations were identified, determinants such as maternal nutritional status and household media exposure are likely influenced by broader socioeconomic conditions that also directly affect child stunting. Maternal BMI, for example, may shape child nutrition but is ~~not possible~~itself conditioned by household food insecurity and poverty, while media exposure may influence health behaviors yet remains dependent on socioeconomic access. Without longitudinal data, the temporal of these relationships remains uncertain. Therefore, the findings should be interpreted as highlighting important populations-level correlates rather than confirming causal pathways. Future prospective cohort studies are warranted to disentangle these relationships and clarify the causal mechanisms underlying child stunting in SSA.

Conclusion

In conclusion, this study analyzed pooled data from 35 sub-Saharan African (SSA) countries and found an overall stunting prevalence of 29.72% among children under ~~five~~5 years of age, with significant variations across countries, ranging from 13.74% in Gabon to 55.74% in Burundi. Fourteen countries had stunting rates exceeding 30%, indicating a very high public health concern as per WHO thresholds.

Our study identified individual, household, and contextual factors significantly linked to stunting in children under ~~five.~~5 years of age. At the individual level, key predictors included being male, increased age of child, higher birth order (fifth or more), small or medium birth size, insufficient antenatal care (ANC) visits (none or 1–3), lack of postnatal visits, lower maternal education (no formal, primary, or secondary education), home delivery, single marital status, and maternal underweight, all of which were associated with increased odds of stunting. In contrast, maternal

Formatted: Font: 16 pt

Formatted: Space After: 0 pt

overweight or obesity were linked to reduced odds of stunting. At the household level, factors such as lack of maternal media exposure (television, radio, or newspapers), ~~use of unimproved water sources,~~ large family size, ~~reliance on unclean cooking fuels,~~ and lower household wealth (~~poorest, poorer~~poor, middle, ~~or richer~~rich) were associated with higher odds of stunting. Additionally, at the contextual level, being rural residence was a significant predictor of stunting.

To address ~~the~~persistently high prevalence of stunting in sub-Saharan Africa, ~~targeted~~ interventions should ~~focus on~~ be clearly prioritized by feasibility and timeline but framed within both household level and structural determinants. Immediate actions remain critical such as improving maternal and child healthcare access~~, including increasing~~ through increased antenatal and postnatal care ~~visits~~visit, promoting facility-based deliveries, and enhancing maternal education to ~~improve~~strengthen feeding practices and nutrition awareness. ~~Household level interventions should prioritize~~Expanding media outreach can also provide rapid gains in health and nutrition knowledge.

However, this effort must be complemented by long term structural and systems level investments that tackle the root cause of the stunting. Poverty alleviation, resilient food systems, and equitable access to ~~clean water~~nutritious diets are central to reducing vulnerability. Governance and political stability play a decisive role in ensuring effective implementation of nutrition policies while conflict and displacement exacerbate food insecurity and health service disruptions. Addressing gender inequality in child nutrition, improved access to clean water and sanitation, ~~and~~expanding clean cooking fuels, ~~alongside poverty alleviation programs to address economic~~and bridging between urban-rural disparities~~. Contextual efforts should aim to bridge the rural-urban gap by strengthening~~ through strengthened healthcare infrastructure~~, expanding~~ and social protection programs~~, and increasing media outreach to disseminate health and nutrition information.~~ are equality vital.

A multisectoral approach ~~involving~~like integrating health, education, agriculture, food systems and WASH sectors is essential to ~~tackle the root causes of stunting and~~ ensure both short term impact and sustainable improvements in child growth and ~~development across the region~~developments. Future research should incorporate health systems variable like facility availability and quality alongside country level factors like governance, policy coherence and

conflict dynamic. Longitudinal design is needed to establish casual pathways and guide more effective policy strategies that balance individual behavior change with structural reforms.

Additionally, future studies should incorporate health system variables, such as number and type of health facilities, and country-level factors like political stability, to provide a more comprehensive understanding of stunting determinants. Furthermore, longitudinal research designs are recommended to establish causal relationships between independent and dependent variables, enabling more effective policy and intervention strategies to address stunting in sub-Saharan Africa.

Abbreviations and Acronyms

Formatted: Font: 16 pt

AIC: Akaike's Information Criteria

APR: Adjusted Prevalence Ratio

BIC: Bayesian Information Criteria

CI: Confidence Interval

CPR: Crude Prevalence Ratio

DHS: Demographic and Health Survey

ICC: Intra Class Correlation Coefficient

IRB: Institutional Review Board

LL: Log-Likelihood

MOR: Median Odds Ratio

PCV: Proportional Change in Variance

SSA: Sub-Saharan Africa

Acknowledgements

Formatted: Font: 16 pt

The authors thank ICF International for granting access to the SSA DHS data set used in this study.

Authors' Contributions

Formatted: Font: 16 pt

A.H. ~~and K.U. conceived and designed the study, obtained and analyzed the data, and interpreted the results of the manuscript. A.H and O.A were drafted and revised the manuscript. K.G. and B.F. were involved in the study design. E.E and R.M. were involved in data acquisition. T.D and B.K. were in drafting of the manuscript. All authors read and approved the final manuscript and agreed to take responsibility for the contents of this work~~conceptualization, formal analysis, investigation, methodology, writing original draft, review and editing. K.G. methodology, review and editing. K.U. methodology, software, review and editing. B.F. review and editing. E.E. methodology, software, review and editing. R.M. methodology, review and editing. T.D. methodology, software, review and editing. B.K. methodology, review and editing.

Funding

Formatted: Font: 16 pt

Formatted: Space After: 0 pt

No specific fund was received for this work.

Data availability

Formatted: Font: 16 pt

~~The raw dataset used and analyzed in this study can be accessed from the DHS website (http://www.measuredhs.com)~~The datasets analyzed during current study are publicly available from the Demographic and Health Survey (DHS) program, managed by ICF international. The data can be accessed upon reasonable request through the DHS program website at http://www.dhsprogram.com. Researcher must register and submit a data access request through the site. For inquires regarding data access, please contact the DHS Data Archivist at archive@dhsprogram.com. The data used in this study are cited as: ICF. Demographic and Health Surveys (various years). Funded by USAID. Rockville, Maryland. Available at: http://dhsprogram.com.

Declarations

Formatted: Font: 16 pt

Consent for publication

Not Applicable

Competing interests

The authors declare no competing interests.

~~**Ethics approval and consent to participate**~~

~~Access to the data used in this study was authorized by the Data Archivist of Demographic and Health Survey (DHS) Program through an online request made at http://www.dhsprogram.com , supported by an authorization letter AuthLetter_215093 on dated 01/19/2025.~~

References

Formatted: Font: 16 pt

1. De Onis M, Branca F. Childhood stunting: a global perspective. Maternal & child nutrition. 2016;12:12-26.

~~1~~2. Callanan A. Food and Nutrition Handbook2015.

~~2~~3. Bharali N, Singh KN, Mondal N. Composite Index of Anthropometric Failure (CIAF) among Sonowal Kachari tribal preschool children of flood effected region of Assam, India. Anthropological Review. 2019;82(2):163-76.

4. Takele BA, Gezie LD, Alamneh TS. Pooled prevalence of stunting and associated factors among children aged 6–59 months in Sub-Saharan Africa countries: A Bayesian multilevel approach. Plos one. 2022;17(10):e0275889.

~~3. De Onis M, Branca F. Childhood stunting: a global perspective. Maternal & child nutrition. 2016;12:12-26.~~

~~4. Giancola A, Hauerslev M, Ndinda C, Farmer M, Musah L, Msengi G, et al. Addressing the double-burden of malnutrition among children and adolescents in sub-Saharan Africa. Journal of Global Health Reports. 2022;6:e2022033.~~

5. ~~UNICEF. In brief to the state of food~~FOOD R, AFFORDABLE HDM. Food security and nutrition in the ~~world~~World. 2022.~~ 2022.~~

6. Lawal SA, Okunlola DA, Adegboye OA, Adedeji IA. Mother's education and nutritional status as correlates of child stunting, wasting, underweight, and overweight in Nigeria: Evidence from 2018 Demographic and Health Survey. Nutrition and Health. 2024;30(4):821-30.

~~. Unicef.~~7. UNICEF, UNICEF. WHO/World Bank ~~Group~~ Joint Child Malnutrition Estimates ~~2019 edition.~~, 2021 Edition. Disponibil la: https://data unicef org/topic/nutrition/malnutrition. 2022.

~~7. WHO W. WHO. WHO. (2021). The UNICEF/WHO/WB Joint Child Malnutrition Estimates (JME) group released new data for 2021. World Health Organization. https://www.who.int/home/search?indexCatalogue=genericsearchindex1&searchQuery=stunting is&wordsMode=AnyWord. In: World Health Organization [Internet]. 2021. Available: https://www.who.int/data/gho/data/themes/malaria. 2021.~~

~~8. UNICEF. The state of food security and nutrition in the world 2021. 2021.~~

~~9~~8. Mbogori T, Kimmel K, Zhang M, Kandiah J, Wang Y. Nutrition transition and double burden of malnutrition in Africa: a case study of four selected countries with different social economic development. AIMS Public Health. 2020;7(3):425.

~~10~~9~~. Lawal SA, Okunlola DA, Adegboye OA, Adedeji IA. Mother's education and nutritional status as correlates of child stunting, wasting, underweight, and overweight in Nigeria: Evidence from 2018 Demographic and Health Survey. Nutrition and Health. 2024;30(4):821-30.~~

~~11. Takele BA, Gezie LD, Alamneh TS. Pooled prevalence of stunting and associated factors among children aged 6–59 months in Sub-Saharan Africa countries: A Bayesian multilevel approach. Plos one. 2022;17(10):e0275889.~~

~~12~~. Deshpande A, Ramachandran R. Early childhood stunting and later life outcomes: A longitudinal analysis. Economics & Human Biology. 2022;44:101099.

~~13~~10. Soliman A, De Sanctis V, Alaaraj N, Ahmed S, Alyafei F, Hamed N, et al. Early and long-term consequences of nutritional stunting: From childhood to adulthood. Acta Bio Medica: Atenei Parmensis. 2021;92(1):e2021168.

~~14. Dewey KG, Begum K. Long-term consequences of stunting in early life. Maternal & child nutrition. 2011;7:5-18.~~

~~15. Black RE, Allen LH, Bhutta ZA, Caulfield LE, De Onis M, Ezzati M, et al. Maternal and child undernutrition: global and regional exposures and health consequences. The lancet. 2008;371(9608):243-60.~~

~~16~~11. Ahmed KY, Agho KE, Page A, Arora A, Ogbo FA, Maternal G, et al. Mapping geographical differences and examining the determinants of childhood stunting in Ethiopia: a Bayesian geostatistical analysis. Nutrients. 2021;13(6):2104.

~~17~~12. Quamme SH, Iversen PO. Prevalence of child stunting in Sub-Saharan Africa and its risk factors. Clinical Nutrition Open Science. 2022;42:49-61.

~~18.~~13. Dapi Nzefa L, Monebenimp F, Äng C. Undernutrition among children under five in the Bandja village of Cameroon, Africa. South African Journal of Clinical Nutrition. 2019;32(2):46-50.

14. Amare M, Benson T, Fadare O, Oyeyemi M. Study of the determinants of chronic malnutrition in Northern Nigeria: quantitative evidence from the Nigeria demographic and health surveys international food policy research institute (IFPRI) working paper 45 (September 2017). Food and nutrition bulletin. 2018;39(2):296-314.

15. Wolde T, Adeba E, Sufa A. Prevalence of chronic malnutrition (stunting) and determinant factors among children aged 0–23 months in Western Ethiopia: a cross-sectional study. Journal of Nutritional Disorders & Therapy. 2014;4(4):148-54.

16. Awoke A, Ayana M, Gualu T. Determinants of severe acute malnutrition among under five children in rural Enebsie Sarmidr District, East Gojjam Zone, North West Ethiopia, 2016. BMC nutrition. 2018;4(1):4.

17. Ahmed MM, Hokororo A, Kidenya BR, Kabyemera R, Kamugisha E. Prevalence of undernutrition and risk factors of severe undernutrition among children admitted to Bugando Medical Centre in Mwanza, Tanzania. BMC Nutrition. 2016;2(1):49.

18. Rose ES, Blevins M, González-Calvo L, Ndatimana E, Green AF, Lopez M, et al. Determinants of undernutrition among children aged 6 to 59 months in rural Zambézia Province, Mozambique: results of two population-based serial cross-sectional surveys. BMC nutrition. 2015;1(1):41.

19. Mukabutera A, Thomson DR, Hedt-Gauthier BL, Basinga P, Nyirazinyoye L, Murray M. Risk factors associated with underweight status in children under five: an analysis of the 2010 Rwanda Demographic Health Survey (RDHS). BMC Nutrition. 2016;2(1):40.

20. Tibilla M. The nutritional impact of the world food programmesupported supplementary feeding programme on children less than five years in rural tamale. Ghana. 2007.

~~19~~21. Yalew B, Amsalu F, Bikes D. Prevalence and factors associated with stunting, underweight and wasting: a community based cross sectional study among children age 6-59 months at Lalibela Town, Northern Ethiopia. J Nutr Disorders Ther. 2014;4(147):2161-0509.1000147.

~~20~~22. Amare ZY, Ahmed ME, Mehari AB. Determinants of nutritional status among children under age 5 in Ethiopia: further analysis of the 2016 Ethiopia demographic and health survey. Globalization and health. 2019;15:1-11.

~~21. Skoufias E, Vinha K, Sato R. All hands on deck: reducing stunting through multisectoral efforts in Sub-Saharan Africa: World Bank Publications; 2019.~~

~~22. (2019) U.~~23. Poda GG, Hsu C-Y, Chao JC-J. Factors associated with malnutrition among children< 5 years old in Burkina Faso: evidence from the Demographic and Health Surveys IV 2010. International Journal for Quality in Health Care. 2017;29(7):901-8.

24. Haile D, Azage M, Mola T, Rainey R. Exploring spatial variations and factors associated with childhood stunting in Ethiopia: spatial and multilevel analysis. BMC pediatrics. 2016;16(1):49.

25. Talukder A. Factors associated with malnutrition among under-five children: illustration using Bangladesh demographic and health survey, 2014 data. Children. 2017;4(10):88.

26. Skoufias E, Vinha K, Sato R. Reducing stunting through multisectoral efforts in sub-Saharan Africa. Journal of African Economies. 2021;30(4):324-48.

27. UNICEF. ~~(2019).~~ Nutrition Strategy 2020-2030: Accelerating Stunting Reduction in Sub~~-~~ Saharan Africa. New York: UNICEF. 2019. . 2019.

28. ~~23.~~ Desa U. Transforming our world: The 2030 agenda for sustainable development. 2016.

~~24~~29. Stevens GA, Finucane MM, Paciorek CJ, Flaxman SR, White RA, Donner AJ, et al. Trends in mild, moderate, and severe stunting and underweight, and progress towards MDG 1 in 141 developing countries: a systematic analysis of population representative data. The lancet. 2012;380(9844):824-34.

~~25~~30. Bursac Z, Gauss CH, Williams DK, Hosmer DW. Purposeful selection of variables in logistic regression. Source code for biology and medicine. 2008;3~~:~~(1~~-8~~):17.

~~26~~31. Hosmer Jr DW, Lemeshow S, Sturdivant RX. Applied logistic regression: John Wiley & Sons; 2013.

~~27~~32. Croft TN, Marshall AM, Allen CK, Arnold F, Assaf S, Balian S. Guide to DHS statistics. Rockville: ICF. 2018;645:292-303.

33. USAID. Demographic and health survey(DHS) methodology. https://dhsprogram.com/methodology/Survey-Types/DHS-Methodology.cfm n.d. Available.

34. Rahman MS, Howlader T, Masud MS, Rahman ML. Association of low-birth weight with malnutrition in children under five years in Bangladesh: do mother’s education, socio-economic status, and birth interval matter? PloS one. 2016;11(6):e0157814.

~~28~~35. Ntenda PAM. Association of low birth weight with undernutrition in preschool-aged children in Malawi. Nutrition journal. 2019;18:1-15.

~~29~~36. Aboagye RG, Seidu A-A, Ahinkorah BO, Arthur-Holmes F, Cadri A, Dadzie LK, et al. Dietary diversity and undernutrition in children aged 6–23 months in Sub-Saharan Africa. Nutrients. 2021;13(10):3431.

~~30~~37. WHO. WHO . Obesity and overweight. 2021. Available: https://www.who.int/news-room/fact-sheets/detail/obesity-and-overweight [Ref list]. 2021.

~~31~~38. Weir CB, Jan A. BMI classification percentile and cut off points. 2019.

39. Cooper H, Hedges LV, Valentine JC. The handbook of research synthesis and meta-analysis: Russell Sage Foundation; 2019.

40. Bursac Z, Gauss CH, Williams DK, Hosmer DW. Purposeful selection of variables in logistic regression. Source code for biology and medicine. 2008;3:1-8.

41. Greene W. Functional forms for the negative binomial model for count data. Economics Letters. 2008;99(3):585-90.

42. Skoufias E, Vinha K, Sato R. All hands on deck: reducing stunting through multisectoral efforts in Sub-Saharan Africa: World Bank Publications; 2019.

~~32. Tamhane AR, Westfall AO, Burkholder GA, Cutter GR. Prevalence odds ratio versus prevalence ratio: choice comes with consequences. Statistics in medicine. 2016;35(30):5730-5.~~

~~33~~43. (2019) U. UNICEF. (2019). Nutrition Strategy 2020-2030: Accelerating Stunting Reduction in Sub-Saharan Africa. New York: UNICEF. 2019.

44. de Onis M BE, Arimond M, Webb P, Croft T, Saha K et al. de Onis M, Borghi E, Arimond M, Webb P, Croft T, Saha K et al. Prevalence thresholds for wasting, overweight and stunting in children under 5 years. Public Health Nutrition. 2018;1-5. doi:10.1017/S1368980018002434. 2018.

~~34~~45. Akombi BJ, Chitekwe S, Sahle BW, Renzaho AM. Estimating the double burden of malnutrition among 595,975 children in 65 low-and middle-income countries: a meta-analysis of demographic and health surveys. International journal of environmental research and public health. 2019;16(16):2886.

~~35~~46. Ssentongo P, Ssentongo AE, Ba DM, Ericson JE, Na M, Gao X, et al. Global, regional and national epidemiology and prevalence of child stunting, wasting and underweight in low-and middle-income countries, 2006–2018. Scientific reports. 2021;11(1):5204.

~~36~~47. (2016). U. The state of the world's children 2016 report. New York: UNICEF. 2016.

~~37~~48. Kejo D, Mosha TC, Petrucka P, Martin H, Kimanya ME. Prevalence and predictors of undernutrition among underfive children in Arusha District, Tanzania. Food science & nutrition. 2018;6(8):2264-72.

~~38~~49. Seifu BL, Tesema GA, Fentie BM, Yehuala TZ, Moloro AH, Mare KU. Geographical variation in hotspots of stunting among under-five children in Ethiopia: A geographically weighted regression and multilevel robust Poisson regression analysis. Plos one. 2024;19(5):e0303071.

~~39~~50. Li Z, Kim R, Vollmer S, Subramanian S. Factors associated with child stunting, wasting, and underweight in 35 low-and middle-income countries. JAMA network open. 2020;3(4):e203386-e.

~~40~~51. Tesema GA, Yeshaw Y, Worku MG, Tessema ZT, Teshale AB. Pooled prevalence and associated factors of chronic undernutrition among under-five children in East Africa: a multilevel analysis. PLoS One. 2021;16(3):e0248637.

~~41~~52. Akombi BJ, Agho KE, Merom D, Renzaho AM, Hall JJ. Child malnutrition in sub-Saharan Africa: A meta-analysis of demographic and health surveys (2006-2016). PloS one. 2017;12(5):e0177338.

~~42~~53. Khan S, Zaheer S, Safdar NF. Determinants of stunting, underweight and wasting among children< 5 years of age: evidence from 2012-2013 Pakistan demographic and health survey. BMC public health. 2019;19:1-15.

~~43~~54. Raru TB, Ayana GM, Merga BT, Negash B, Deressa A, Birhanu A, et al. Magnitude of under-nutrition among under five children in Ethiopia based on 2019 Mini-Ethiopia Demographic and Health Survey: Generalized Linear Mixed Model (GLMM). BMC nutrition. 2022;8(1):113.

~~44~~55. Badake Q, Maina I, Mboganie M, Muchemi G, Kihoro E, Chelimo E, et al. Nutritional status of children under five years and associated factors in Mbeere South District, Kenya. African Crop Science Journal. 2014;22:799-806.

~~45~~56. Mamiro PS, Kolsteren P, Roberfroid D, Tatala S, Opsomer AS, Van Camp JH. Feeding practices and factors contributing to wasting, stunting, and iron-deficiency anaemia among 3-23-month old children in Kilosa district, rural Tanzania. Journal of Health, Population and Nutrition. 2005:222-30.

~~46~~57. Hien NN, Kam S. Nutritional status and the characteristics related to malnutrition in children under five years of age in Nghean, Vietnam. Journal of Preventive Medicine and Public Health. 2008;41(4):232-40.

~~47~~58. Ruwali D. Nutritional status of children under five years of age and factors associated in Padampur VDC, Chitwan. Health prospect. 2011;10:14-8.

~~48~~59. Sapkota V, Gurung C. Community Medicine and Family Health Department, Maharajgunj Campus, Institute of Medicine, Tribhuwan University. Journal of Nepal Health Research Council. 2009;7(2):120-6.

~~49~~60. Li H, Yuan S, Fang H, Huang G, Huang Q, Wang H, et al. Prevalence and associated factors for stunting, underweight and wasting among children under 6 years of age in rural Hunan Province, China: a community-based cross-sectional study. BMC Public Health. 2022;22(1):483.

~~50~~61. Sultana P, Rahman MM, Akter J. Correlates of stunting among under-five children in Bangladesh: a multilevel approach. BMC nutrition. 2019;5:1-12.

~~51~~62. Kang Y, Aguayo VM, Campbell RK, Dzed L, Joshi V, Waid JL, et al. Nutritional status and risk factors for stunting in preschool children in Bhutan. Maternal & child nutrition. 2018;14:e12653.

~~52~~63. Estimates U-W-WJCM. Child malnutrition estimates for the indicators stunting, wasting, overweight and underweight describe the magnitude and patterns of under- and overnutrition. UNICEF-WHO-WB Joint Child Malnutrition Estimates inter-agency group updates regularly the global and regional estimates in prevalence and numbers for each indicator. 2022.

~~53~~64. Aboagye RG, Ahinkorah BO, Seidu A-A, Frimpong JB, Archer AG, Adu C, et al. Birth weight and nutritional status of children under five in sub-Saharan Africa. Plos one. 2022;17(6):e0269279.

5465. Haile D, Azage M, Mola T, Rainey R. Exploring spatial variations and factors associated with childhood stunting in Ethiopia: spatial and multilevel analysis. BMC pediatrics. 2016;16. Haile D, Azage M, Mola T, Rainey R. Exploring spatial variations and factors associated with childhood stunting in Ethiopia: spatial and multilevel analysis. BMC pediatrics. 2016;16:1-14.

5566. Hailu BA, Bogale GG, Beyene J. Spatial heterogeneity and factors influencing stunting and severe stunting among under-5 children in Ethiopia: spatial and multilevel analysis. Scientific reports. 2020;10(1):16427.

5667. Muche A, Gezie LD, Baraki AG-e, Amsalu ET. Predictors of stunting among children age 6–59 months in Ethiopia using Bayesian multi-level analysis. Scientific Reports. 2021;11(1):3759.

5768. Tamir TT, Techane MA, Dessie MT, Atalell KA. Applied nutritional investigation spatial variation and determinants of stunting among children aged less than 5 y in Ethiopia: A spatial and multilevel analysis of Ethiopian Demographic and Health Survey 2019. Nutrition. 2022;103:111786.

5869. Harttgen K, Klasen S, Vollmer S. Economic growth and child undernutrition in sub-Saharan Africa. Population and development review. 2013;39(3):397-412.

5970. Ahmad D, Afzal M, Imtiaz A. Effect of socioeconomic factors on malnutrition among children in Pakistan. Future Business Journal. 2020;6:1-11.

6071. Fantay Gebru K, Mekonnen Haileselassie W, Haftom Temesgen A, Oumer Seid A, Afework Mulugeta B. Determinants of stunting among under-five children in Ethiopia: a multilevel mixed-effects analysis of 2016 Ethiopian demographic and health survey data. BMC pediatrics. 2019;19:1-13.

6172. Wamani H, Åstrøm AN, Peterson S, Tumwine JK, Tylleskär T. Boys are more stunted than girls in sub-Saharan Africa: a meta-analysis of 16 demographic and health surveys. BMC pediatrics. 2007;7:1-10.

62. Hill K, Upchurch DM. Gender differences in child health: evidence from the demographic and health surveys. Population and development review. 1995:127-51.

63. Green MS. The male predominance in the incidence of infectious diseases in children: a postulated explanation for disparities in the literature. International journal of epidemiology. 1992;21(2):381-6.

64. Abeway S, Gebremichael B, Murugan R, Assefa M, Adinew YM. Stunting and its determinants among children aged 6–59 months in Northern Ethiopia: A cross-sectional study. Journal of nutrition and metabolism. 2018;2018(1):1078480.

65. Demissie S, Worku A. Magnitude and factors associated with malnutrition in children 6-59 months of age in pastoral community of Dollo Ado district, Somali region, Ethiopia. Sci J Public Health. 2013;1(4):175-83.

66. Pandit D, Pai N. A total care of the female child--a step towards safe motherhood. Indian journal of maternal and child health: official publication of Indian Maternal and Child Health Association. 1991;2(2):64.

6773. Mertens A, Benjamin-Chung J, Colford Jr JM, Coyle J, Van der Laan MJ, Hubbard AE, et al. Causes and consequences of child growth faltering in low-resource settings. Nature. 2023;621(7979):568-76.

74. Nyamasege C, Kimani-Murage E, Wanjohi M, Kaindi D, Wagatsuma Y. Effect of maternal nutritional education and counselling on children's stunting prevalence in urban informal settlements in Nairobi, Kenya. Public health nutrition. 2021;24(12):3740-52.

75. Teshome B, Kogi-Makau W, Getahun Z, Taye G. Magnitude and determinants of stunting in children underfive years of age in food surplus region of Ethiopia: the case of west gojam zone. Ethiopian Journal of Health Development. 2009;23(2).

6876. Nor SM, Mwajuma J, Mohamed MAA. Determinants of Malnutrition among Children under five years in SOS Hospital, Mogadishu. 2016.

6977. Niyigena DI, Semayira CA, Mutabazi M, Ntirushwamaboko N, de Dieu Habimana J, Iyakaremye D, et al. Feeding practices and nutritional status among children aged from six to 23 months in Western Province, Rwanda: a cross-sectional study. Rwanda Journal of Medicine and Health Sciences. 2023;6(2):228-38.

~~70~~78. Marume A, Archary M, Mahomed S. Predictors of stunting among children aged 6–59 months, Zimbabwe. Public Health Nutrition. 2023;26(4):820-33.

~~71~~79. Investigators M-EN. Relationship between growth and illness, enteropathogens and dietary intakes in the first 2 years of life: findings from the MAL-ED birth cohort study. BMJ global health. 2017;2(4):e000370.

80. González-Fernández D, Cousens S, Rizvi A, Chauhadry I, Soofi SB, Bhutta ZA. Infections and nutrient deficiencies during infancy predict impaired growth at 5 years: Findings from the MAL-ED study in Pakistan. Frontiers in Nutrition. 2023;10:1104654.

81. Jardí C, Casanova BD, Arija V. Nutrition education programs aimed at african mothers of infant children: a systematic review. International Journal of Environmental Research and Public Health. 2021;18(14):7709.

82. Rahman M. Association between order of birth and chronic malnutrition of children: a study of nationally representative Bangladeshi sample. Cadernos de saude publica. 2016;32:e00011215.

~~72~~83. Khan REA, Raza MA. Nutritional status of children in Bangladesh: measuring composite index of anthropometric failure (CIAF) and its determinants. 2014.

~~73~~84. Shapiro-Mendoza C, Selwyn BJ, Smith DP, Sanderson M. Parental pregnancy intention and early childhood stunting: findings from Bolivia. International journal of epidemiology. 2005;34(2):387-96.

~~74~~85. Ukwuani FA, Suchindran CM. Implications of women's work for child nutritional status in sub-Saharan Africa: a case study of Nigeria. Social science & medicine. 2003;56(10):2109-21.

~~75. Research NIoP, Training. Mitra and Associates, and ICF International. Bangladesh Demographic and Health Survey 2014. 2016.~~

~~76. Research NIoP, Training, Mitra, Associates, International M. Bangladesh demographic and health survey, 2007: NIPORT; 2009.~~

~~77~~86. Saaka M, Aggrey B. Effect of birth interval on foetal and postnatal child growth. Scientifica. 2021;2021(1):6624184.

87. Zhao M, Wu H, Liang Y, Liu F, Bovet P, Xi B. Breastfeeding and mortality under 2 years of age in sub-Saharan Africa. Pediatrics. 2020;145(5):e20192209.

88. Dasgupta DP, Sujon MSH, Afroz M, Mazumder S, Suman SM, Khan MTF. Impact of Birth Spacing and Maternal-Child Risk Factors on Childhood Stunting, Wasting, Underweight, and their Coexistence in South Asia. Wasting, Underweight, and their Coexistence in South Asia.

89. Damtie Y, Kefale B, Yalew M, Arefaynie M, Adane B. Short birth spacing and its association with maternal educational status, contraceptive use, and duration of breastfeeding in Ethiopia. A systematic review and meta-analysis. PLoS One. 2021;16(2):e0246348.

90. Gebru KF HW, Temesgen AH, Seid AO, Mulugeta BA. Determinants of stunting among under-five children in Ethiopia: a multilevel mixed-effects analysis of 2016 Ethiopian demographic and

health survey data. BMC pediatrics. 2019; 19(1):1–13. 2019.

~~78~~91. Moges B FA, Meseret S, Doyore F. Magnitude of stunting and associated factors among 6–59 months old children in Hossana Town, Southern Ethiopia. Journal of Clinical Research & Bioethics.

2015; 6(1):1. 2015.

~~79~~92. Agho KE IK, Bowe SJ, Jacobs J, Dibley MJ. . Prevalence and risk factors for stunting and severe stunting among under-fives in North Maluku province of Indonesia. BMC pediatrics. 2009; 9(1):1–10.

https://doi.org/10.1186/1471-2431-9-64 PMID: 19818167. 2009.

~~80~~93. Tiwari R, Ausman M. L., & Emwinyore Agho, K.(2014). Determinants of stunting and severe stuntingamong under-fives: evidence from the 2011 Nepal Demographic and Health Survey. BMC pediatrics.14:239.

8194. Rahman A, Chowdhury S. Determinants of chronic malnutrition among preschool children in Bangladesh. Journal of biosocial science. 2007;39(2):161-73.

8295. Yimer G. Malnutrition among children in Southern Ethiopia: Levels and risk factors. 2000.

83. Anil K BP, Singh S. . Low birth weight and its associated risk factors: Health facility-based casecontrol study. PloS one. 2020; 15(6):e0234907. https://doi.org/10.1371/journal.pone.0234907 PMID:32569281. 2020.

84. Adedokun ST YS. Childhood morbidity and its determinants: evidence from 31 countries in subSaharan Africa. BMJ Global Health. 2020; 5(10):e003109. https://doi.org/10.1136/bmjgh-2020-003109PMID: 33046457. 2020.

8596. Goddard FG, Hunegnaw BM, Luu J, Haneuse S, Zeleke M, Mohammed Y, et al. Child stunting from birth to age two years: The Birhan Cohort in Ethiopia. medRxiv. 2023:2023.05. 20.23290246.

97. Habtu M, Agena AG, Umugwaneza M, Mochama M, Munyanshongore C. Effectiveness of integrated maternal nutrition intervention package on birth weight in Rwanda. Frontiers in Nutrition. 2022;9:874714.

98. Bigool M. Effects of free maternal healthcare on stunting in children under five years of age: Evidence from Ghana. Development Policy Review. 2024;42(4).

99. T. P. Review of Maternal Effects on Early Childhood Stunting. 2014. 2014.

86100. Budhathoki SS BA, Gurung R, Gurung A, Ashish K. Stunting among under 5-year-olds in Nepal: trends and risk factors. Maternal and child health journal. 2020;24(1):39–47. 2020.

87101. Mzumara B BP, Halwiindi H, Mugode R, Banda J. Factors associated with stunting among children below five years of age in Zambia: evidence from the 2014 Zambia demographic and health survey. BMC nutrition. 2018;4(1):1–8. pmid:32153912. 2018.

88102. Akombi BJ AK, Hall JJ, Wali N, Renzaho A, Merom D. . Stunting, wasting and underweight in sub-Saharan Africa: a systematic review. International journal of environmental research and public health. 2017;14(8):863. pmid:28788108. 2017.

89. YM.103. Makoka D. Factors, Masibo PK. Is there a threshold level of maternal education sufficient to reduce child undernutrition? Evidence from Malawi, Tanzania and Zimbabwe. BMC pediatrics. 2015;15(1):96.

104. Ickes S, Baguma C, Brahe CA, Myhre JA, Adair L, Bentley M, et al. Maternal participation in a nutrition education program in Uganda is associated with mothers' knowledge onimproved infant and young child feeding recommendation in slum areas of Bahir Dar City, Ethiopia: cross sectionalpractices and feeding knowledge: a post-program comparison study. BMC research notesnutrition. 2017;10(1):3(1–7. 2017):32.

90. P. G. Why does mother's schooling raise child health in developing countries? Evidence from Morocco. Journal of human resources. 1999:124–59. 1999.

91. Tadesse SE MT. Prevalence and Associated Factors of Stunting Among Children Aged 6–59 Months in Delanta District; North East Ethiopia. 2019.

92105. Asgedom YS SB, Mare KU, Asmare ZA, Asebe HA, Kase BF, et al. Levels of stunting associated factors among under-five children in Ethiopia: A multi-level ordinal logistic regression analysis. 2024.

93106. Finlay JE ÖE, Canning D. The association of maternal age with infant mortality, child anthropometric failure, diarrhea and anemia for first births: evidence from 55 low-and middle-income countries. BMJ open. 2011;1(2):e000226. [DOI] [PMC free article] [PubMed] [Google Scholar][Ref list]. 2011.

94. Habimana S BE. Risk factors of stunting among children under 5 years of age in the eastern and western provinces of Rwanda: analysis of Rwanda demographic and health survey 2014/2015. Pediatric health, medicine, and therapeutics. 2019:115–30. doi: 10.2147/PHMT.S222198 [DOI] [PMC free article] [PubMed] [Google Scholar][Ref list]. 2014/2015.

~~95~~107. Chilinda ZB, Wahlqvist ML, Lee M-S, Huang Y-C. Higher maternal autonomy is associated with reduced child stunting in Malawi. Scientific reports. 2021;11(1):3882.

108. Behailu T. AM, Mache T., Girma A. Prevalence and risk factors of child malnutrition in community based nutrition program implementing and nonimplementing districts from South East Amhara, Ethiopia. Open Access Library Journal. 2014;1(3):1–17. doi: 10.4236/oalib.1100425. 2014.

~~96~~109. Shiferaw Abeway BG, 2 Rajalakshmi Murugan,2 Masresha Assefa,3, and Yohannes Mehretie Adinew. Stunting and Its Determinants among Children Aged 6–59 Months in Northern Ethiopia: A Cross-Sectional Study. 2018.

~~97~~110. Abeway S GB, Murugan R, Assefa M, Adinew YM. . Stunting and its determinants among children aged 6–59 months in northern Ethiopia: a cross-sectional study. J Nutr Metab. 2018;2018:1–8. doi:10.1155/2018/1078480. 2018.

~~98~~111. Bupe B. BML, Mapoma C. C., Mutombo N. . Factors associated with stunting among children aged 6–23 months in Zambian: Evidence from the 2007 Zambia Demographic and Health Survey. International Journal of Advanced Nutritional and Health Science. 2015;2(1). 2015.

~~99~~112. Forero-Ramirez N GL, Bedi A, Sparrow R. . Child malnutrition and prenatal care: evidence from three Latin American countries. Revista Panamericana de Salud Pública. 2014;35:163–171. 2014.

~~100~~113. Ramirez N GL, Bedi AS, Sparrow R. . Child malnutrition and antenatal care: evidence from three Latin American countries. ISS Working Paper Series. 2012;536(536):1–28. 2012.

~~101~~114. Svefors P SO, Ekstrom E-C, et al. . Relative importance of prenatal and postnatal determinants of stunting: data mining approaches to the MINIMat cohort, Bangladesh. BMJ Open. 2019;9

(8):e025154. doi:10.1136/bmjopen-2018-025154. 2019.

~~102~~115. Habibov NN FL. Does prenatal healthcare improve child birthweight outcomes in Azerbaijan? Results of the national Demographic

and Health Survey. Econ Hum Biol. 2011;9(1):56–65. doi:10.1016/j.ehb.2010.08.003. 2011.

~~103~~116. G. G-G. The impact of adequate prenatal care on urban birth outcomes: an analysis in a developing country context. Econ Dev Cult Change. 2013;62(1):95–130. doi:10.1086/671716. 2013.

~~104~~117. Langlois ÉV, Miszkurka M, Zunzunegui MV, Ghaffar A, Ziegler D, Karp I. Inequities in postnatal care in low-and middle-income countries: a systematic review and meta-analysis. Bulletin of the World Health Organization. 2015;93:259-70G.

118. Abdulraheem A, Ononokpono D, Raimi M. Breaking barriers to safe motherhood: how social, cultural, and geographic inequalities shape skilled birth attendance in Nigeria. Sociol Int J. 2025;9(5):188-200.

119. Megabiaw B RA. Prevalence and determinants of chronic malnutrition among under-5 children in Ethiopia. International Journal of Child Health and Nutrition. 2013;2(3):230–6. 2013.

~~105~~120. stunting UC. US Cww. stunting: and waht it is and what it means 2019 [cited 2021]. Available from: https://www.concernusa.org/story/what-is-stunting/. 2019.

~~106~~121. Tadesse SE MT. Prevalence and Associated Factors of Stunting Among Children Aged 6–59 Months in Delanta District; North East Ethiopia. 2019.

122. Elisaria E, Mrema J, Bogale T, Segafredo G, Festo C. Effectiveness of integrated nutrition interventions on childhood stunting: a quasi-experimental evaluation design. BMC nutrition. 2021;7(1):17.

123. Mwamba MS, Gerrior S, Taylor R. Relationship between single motherhood status and stunting among children under 5 in Kgatleng, Botswana. Journal of Social, Behavioral, and Health Sciences. 2022;16(1):40-7.

~~107~~124. Gibbs CM, Wendt A, Peters S, Hogue CJ. The impact of early age at first childbirth on maternal and infant health. Paediatric and perinatal epidemiology. 2012;26:259-84.

~~108~~125. Prof Robert E Black MrjePCGV, MDb · Prof Susan P Walker, PhDc · Prof Zulfiqar A Bhutta, PhDd,* · Prof Parul Christian, DrPHa,* · Mercedes de Onis, MDe,*· et al. . Maternal and child undernutrition and overweight in low-income and middle-income countries. 2013.

~~109~~126. Amaha ND, Woldeamanuel BT. Maternal factors associated with moderate and severe stunting in Ethiopian children: analysis of some environmental factors based on 2016 demographic health survey. Nutrition Journal. 2021;20:1-9.

~~110~~127. Berhe K, Seid O, Gebremariam Y, Berhe A, Etsay N. Risk factors of stunting (chronic undernutrition) of children aged 6 to 24 months in Mekelle City, Tigray Region, North Ethiopia: An unmatched case-control study. Plos one. 2019;14(6):e0217736.

~~111~~128. Kamal S. Socio-economic determinants of severe and moderate stunting among under-five children of rural Bangladesh. Malaysian journal of nutrition. 2011;17(1).

~~112. Black RE, Victora CG, Walker SP, Bhutta ZA, Christian P, De Onis M, et al. Maternal and child undernutrition and overweight in low-income and middle-income countries. The lancet. 2013;382(9890):427-51.~~

~~113. Fink G GI, Hill K. . The effect of water and sanitation on child health: evidence from the demographic and health surveys 1986–2007. International journal of epidemiology. 2011;40(5):1196–204. pmid:21724576. 2011.~~

~~114. B. M. Mengistu B. The impact of access to water and sanitation on child stunting in Ethiopia: ACIPH; 2020. 2020.~~

~~115. Joseph G HS, Moqueet N, Hoo YR. Children Need Clean Water to Grow: E. Coli Contamination of Drinking Water and Childhood Nutrition in Bangladesh: The World Bank; 2019. 2019.~~

~~116~~129. Kota K, Pongou R, Chomienne M-H. Impact of household food insecurity on the use of maternal health services in the Savanes region, Togo: a qualitative study. BMC Public Health. 2025;25(1):2040.

130. Okyere J, Budu E, Aboagye RG, Seidu AA, Ahinkorah BO, Yaya S. Socioeconomic determinants of the double burden of malnutrition among women of reproductive age in sub-Saharan Africa: A cross-sectional study. Health Science Reports. 2024;7(5):e2071.

131. Yaya S, Oladimeji O, Odusina EK, Bishwajit G. Household structure, maternal characteristics and children's stunting in sub-Saharan Africa: evidence from 35 countries. International Health. 2022;14(4):381-9.

~~117~~132. Bogale TY, Bala ET, Tadesse M, Asamoah BO. Prevalence and associated factors for stunting among 6–12 years old school age children from rural community of Humbo district, Southern Ethiopia. BMC public health. 2018;18:1-8.

~~118~~133. Asefa A, Girma D, Kaso AW, Ferede A, Agero G, Beyen TK. Prevalence of stunting and associated factors among under-five children in Robe Woreda, Arsi zone, Ethiopia. International Journal of Africa Nursing Sciences. 2024;21:100782.

~~119~~134. Desai S, Alva S. Maternal education and child health: Is there a strong causal relationship? Demography. 1998;35(1):71-81.

~~120~~135. Sarma H KJ, Asaduzzaman M, Uddin F, Tarannum S, Hasan MM, et al. Factors influencing the prevalence of stunting among children aged below five years in Bangladesh. Food and nutrition bulletin. 2017;38(3):291–301. pmid:28758423. 2017.

~~121~~136. Ali S AA. Factors Associated with Stunting among Children Under Five Years of Age in Pakistan: Evidence from PDHS 2012–13. J Community Public Health Nurs. 2018;4:1–4. 2012–13.

~~122~~137. Mbuya NV CR, Morimoto T, Thitsy S. . Media and Messages for Nutrition and Health: Assessing Media Appropriateness for Nutrition and Health-Related Social and Behavior Change Communication in Four High Stunting-Burden Provinces of Lao PDR. World Bank; 2020. 2020.

~~123. Bruce NG, Dherani MK, Das JK, Balakrishnan K, Adair-Rohani H, Bhutta ZA, et al. Control of household air pollution for child survival: estimates for intervention impacts. BMC public health. 2013;13:1-13.~~

~~124. Smith KR, Bruce N, Balakrishnan K, Adair-Rohani H, Balmes J, Chafe Z, et al. Millions dead: how do we know and what does it mean? Methods used in the comparative risk assessment of household air pollution. Annual review of public health. 2014;35(1):185-206.~~

~~125~~138. Woldesenbet B, Tolcha A, Tsegaye B. Water, hygiene and sanitation practices are associated with stunting among children of age 24-59 months in Lemo district, South Ethiopia, in 2021: community based cross sectional study. BMC nutrition. 2023;9(1):17.

139. Seiler J, Libby TE, Jackson E, Lingappa J, Evans W. Social media–based interventions for health behavior change in low-and middle-income countries: systematic review. Journal of medical Internet research. 2022;24(4):e31889.

140. ZT. W. Magnitude and determinants of stunting among children in Africa: a systematic review. Current Research in Nutrition and Food Science Journal. 2014;2(2):88–93. 2014.

~~126~~141. Akombi BJ, Agho KE, Hall JJ, Wali N, Renzaho AM, Merom D. Stunting, wasting and underweight in sub-Saharan Africa: a systematic review. International journal of environmental research and public health. 2017;14(8):863.

~~127~~142. Budhathoki SS BA, Gurung R, Gurung A, Ashish K. . Stunting among under 5-year-olds in Nepal: trends and risk factors. Maternal and child health journal. 2020;24(1):39–47. 2020.

~~128. Taruvinga A MV, Mushunje A. Determinants of rural household dietary diversity: The case of Amatole and Nyandeni districts, South Africa. International Journal of Development and Sustainability. 2013;2(4):2233–47. 2013.~~

~~129. Fagbamigbe AF BE, Yusuf BO, Akinyemi JO, Issa BK, Ngige E, et al. . The Nigeria wealth distribution and health seeking behaviour: evidence from the 2012 national HIV/AIDS and reproductive health survey. Health economics review. 2015;5(1):1–10. pmid:25853003. 2015.~~

~~130~~143. Tsega Y, Endawkie A, Kebede SD, Abeje ET, Enyew EB, Daba C, et al. Trends of wealth-related inequality in stunting and its contributing factors among under-five children in Ethiopia: Decomposing the concentration index using Ethiopian Demographic Health Surveys 2011–2019. PloS one. 2025;20(2):e0314646.

144. Kountchou A, Sonne S, Gadom G, editors. The local impact of armed conflict on children's nutrition and health outcomes: evidence from Chad. CSAE Conference: University of Oxford; 2019.

145. Baye K, Laillou A, Chitweke S. Socio-economic inequalities in child stunting reduction in sub-Saharan Africa. Nutrients. 2020;12(1):253.

146. Tariq J SA, Zakar R, Zakar MZ, Fischer F. . Factors associated with undernutrition in children under the age of two years: secondary data analysis based on the Pakistan demographic and health survey 2012–2013. Nutrients. 2018;10(6):676. 2018.

~~131~~147. Woldeamanuel BT TT. Risk factors associated with under-five stunting, wasting, and underweight based on Ethiopian Demographic Health Survey datasets in Tigray region, ethiopia. Journal of nutrition and metabolism. 2019;2019. 2019.

~~132~~148. Smith LC RM, Ndiaye A. Why is child malnutrition lower in urban than in rural areas? Evidence from 36 developing countries. World development. 2005;33(8):1285–305. 2005.

Author response to reviewer

Manuscript ID: PONE-D-25-15249

Manuscript Title: Prevalence of Stunting and its Determinants Among Children Under Five in 35 Sub-Saharan African Countries (2011–2024): Insights from recent Demographic Health Survey Data Using a Generalized Linear Mixed-Effects Model with Robust Poisson Regression

1. Response to Reviewer 1

Comments	Author Response	Page number in the manuscript
1. I want to thank the editor and the authors for the chance to review this manuscript. The subject of childhood stunting in sub-Saharan Africa is deeply important, and I appreciate the careful work that has gone into this study. It was a privilege to read and reflect on the findings, and I hope my comments will be useful as you move the paper forward.<br>2. General feedback: This paper is very well put together and speaks to a pressing public health concern. The authors draw on DHS data from 35 countries and use appropriate statistical approaches, including robust Poisson regression and mixed-effects modeling, which help account for the survey design and avoid common issues with odds ratios. The sample size is impressive (over 190,000 children), which gives the study strong statistical power and makes the findings more widely applicable. The results are presented clearly, the methods are solid, and the conclusions are well supported by the data.	**Thank you very much for your encouragement and constructive comments.**	
**Introduction**		
1. The introduction does a good job of highlighting the global and regional burden of stunting, but it feels crowded with statistics. The numbers are useful, yet they sometimes overwhelm the main message, which should be that stunting remains a serious child health issue in sub-Saharan Africa despite global progress.	**Revised as commented.**	**3**

2. The authors note that their study fills a gap by using pooled data from 35 countries, but this point could be made more directly. At present, it is not fully clear how this analysis differs from single-country or regional studies and what unique value it adds. Readers should come away with a stronger sense of why this pooled approach is important.	**Revised as commented**	**3**
3. The introduction leans heavily on prevalence estimates and less on the "why." It would be stronger if it included more about the pathways linking maternal, household, and structural factors to stunting. This would help readers understand the reasoning behind the choice of variables and provide a clearer foundation for the study. A conceptual framework showing and visualizing pathways would be complementary.	**Revised as commented**	**5**
4. The section moves quickly between global, regional, and national levels. While this shows the breadth of the issue, the flow could be improved by focusing earlier and more directly on sub-Saharan Africa, since that is the real focus of the paper.	**Revised as commented**	**3**
5. The writing is generally clear but could benefit from light editing to improve flow and fix awkward wording. For example, phrases like "which previous study not accounted incorporated in to analysis" should be revised for clarity.	**Revised as commented**	**All**
**Methods**		
1. The paper mentions that 23 variables were included, but it is not entirely clear how these were chosen. While prior studies are referenced, the rationale for why these specific variables were prioritized over others could be explained better. Without that clarity, it is hard to know whether the model might be at risk of being overloaded.	**Revised as commented**	**8-9**
2. The manuscript notes that children with missing data were excluded, but it is not clear how the missingness was assessed by the authors or whether its impact on representativeness was considered. Because this is a secondary dataset covering 35 countries, it is possible that patterns in missing data during collection shaped which cases were ultimately included. Adding a short note about this would help bring more clarity and transparency.	**Revised as commented**	**8-9**
3. The study draws on surveys from 35 countries collected over more than a decade. Since survey years and tools may vary a bit, it would be helpful to explain how this variation was handled. For example, what steps were taken to	**Revised as commented**	**6-7**

make sure that one country or survey year did not carry too much weight in the pooled results?		
4. The study draws on surveys from 35 countries collected over more than a decade. Since survey years and tools may vary a bit, it would be helpful to explain how this variation was handled. For example, what steps were taken to make sure that one country or survey year did not carry too much weight in the pooled results?	**Revised as commented**	**6**
5. The authors mention applying weights, but it is not clear whether these were adjusted for pooling across countries. Since sample sizes vary widely, larger countries may dominate the results if weights are not harmonized. DHS weights make each survey nationally representative, but when pooled they do not automatically align, so rescaling or normalizing is often needed to ensure balanced contributions.	**Revised as commented**	**6-7**
6. The authors also report AIC and BIC, but there is no discussion of whether Poisson assumptions were checked. Because stunting data are often over dispersed, ignoring this can bias standard errors and inferences. Robust Poisson regression helps, but a short note on whether overdispersion was considered would add confidence in the analysis.	**Revised as commented**	**11**
7. Maternal BMI could affect child nutrition, but it could also be shaped by the same household food insecurity or poverty that drives stunting. Similarly, media exposure might influence health behaviors, but access to media is itself determined by socioeconomic status. Without temporal ordering, the relationships in the model can run both ways, which means the results should be interpreted as associations rather than causal effects. Authors should acknowledge the limits of causal interpretation given the cross-sectional design.	**Revised as commented**	**33**
**Result**		
1. There are places where the confidence intervals seem disordered (for example, lower bounds listed higher than upper bounds). These appear to be typos	**Revised as commented**	**21-22**
2. In the country level variation, it is not clear whether differences across countries were statistically tested or whether these patterns are being highlighted simply for descriptive interest. Greater clarity here would help	**Revised as commented. We added sensitivity analysis, sub-group and meta-regression for heterogeneity**	**15-19**
3. Some of the associations are discussed in a way that suggests causal direction (for example, maternal BMI "reduces risk"), but the results come from a cross-	**Revised as commented**	**21-24**

sectional dataset. The narrative would be strengthened by consistently framing these as associations rather than causal effects. For instance, maternal BMI is described as being "associated with stunting," but the explanation reads as though underweight mothers cause poor nutrient transfer and overweight or obese mothers prevent stunting by having better food access. Media exposure is also presented as an association, yet the discussion suggests media itself changes behaviors and outcomes. In the same way, antenatal and postnatal care are framed as if they directly reduce stunting, with wording that children of mothers who missed visits were "more likely" to be stunted because they missed out on nutrition education.		
**Discussion**		
1. Although the authors report associations in the results, parts of the discussion read as if the study demonstrates cause and effect. For example, maternal BMI, media exposure, and health service use are described in ways that imply they directly reduce or increase stunting. Because this is a cross-sectional study, the findings should be framed consistently as associations rather than causal effects.	**Revised as commented**	**29-30**
2. The discussion does not fully explore the variation across countries. While the pooled analysis is informative, it can mask important differences between contexts. Reflecting on why some countries deviate from the overall pattern, and what that means for interventions, would add more depth.	**Revised as commented**	**25-26**
3. Policy implications are mentioned but not clearly prioritized. For instance, maternal education, ANC/PNC attendance, and socioeconomic status are all important, but it would help to distinguish which determinants are more feasible targets for immediate policy action and which require longer-term structural investments.	**Revised as commented**	**33-34**
4. The study is cited alongside some prior work, but the discussion could more strongly situate the findings within the broader literature on child stunting in sub-Saharan Africa. How do these results compare with evidence from longitudinal studies or interventions? This would give readers a clearer sense of what is confirmed, what is new, and what remains uncertain.	**Revised as commented**	**24-32**
5. The limitations section is rather brief. Issues such as missing data, the cross-sectional design of DHS surveys, and the possibility of residual confounding	**Revised as commented**	**32-33**

deserve more explicit acknowledgment. Without this, readers may overestimate the strength of the conclusions.		
6. Much of the interpretation centers on maternal and household factors. Bringing in structural determinants such as poverty, food systems, governance, or conflict would provide a fuller picture. Without this perspective, the discussion risks placing too much emphasis on individual behavior change as the solution. Think structural and systems-level changes.	**Revised as commented**	**24-32**

2. Response to Reviewer 2

**Comments**	**Author Response**	**Page number in the manuscript**
1. Thank you for submitting your manuscript to PLOS ONE. After careful consideration, we feel that it has merit but does not fully meet PLOS ONE's publication criteria as it currently stands. Therefore, we invite you to submit a revised version of the manuscript that addresses the points raised during the review process.<br>2. This is a well-conceived and timely study that addresses a critical public health issue—stunting among under-five children in Sub-Saharan Africa (SSA). The manuscript demonstrates a solid grasp of epidemiological analysis and effectively utilizes secondary data from the Demographic and Health Surveys (DHS). The topic aligns with global priorities such as the Sustainable Development Goals (SDG 2.2), and the methodological rigor is commendable.	**Thank you very much for your encouragement and constructive comments.**	
**General comments**		
1. You did not line-number your manuscript to help reviewers pinpoint the exact areas requiring revision in the manuscript.	**Revised as commented**	**All**
2. There is inconsistent use of terms for your outcome variable: "stunting among U-5 children", "stunting", "childhood stunting", "stunting among under five children",	**Revised as commented**	**All**
**Abstract**		
1. Your abstract exceeds 300 words, so you need to be parsimonious in the reporting of your findings. You can report the most salient findings: the child's	**Revised as commented**	**2-3**

sex, age & birth size; the mother's education; and household wealth, i.e, those with aPR ≥±20%		
2. Please revise this statement "Key variables such as antenatal care visits, postnatal care, and maternal nutritional indicators which previous study not accounted incorporated in to analysis". Suggestion: Key variables such as antenatal care visits, postnatal care, and maternal nutritional indicators, which previous studies did not account for, are incorporated into the analysis.	**Revised as commented**	**2**
3. Please revise this statement: "The adjusted prevalence ratio and 95% confidence intervals were used to indicate statistical significance and the strength of associations". Suggestion: "The adjusted prevalence ratios and their 95% confidence intervals were used to assess the strength and statistical significance of associations".	**Revised as commented**	**2**
4. The abstract results are overloaded with too much information. Kindly reduce the results to the salient ones as suggested above. In fact, the entire multivariate analysis results in the result section need to be rewritten. Again, the term "children older age" is ambiguous, and the aPR value of 2.32, which you stated, is misleading. It is better to say: "being aged 12 months or older" (aPR: ≥1.81, p<0.01). Also, "mother without a higher education (aPR: ≥1.68, p<0.01), and living in a poor or average household (aPR: ≥1.23, p<0.01).	**Revised as per the comments. We tried as much as possible.**	**2**
**Methods**		
1. I suggest you merge the poorest and the poorer categories into one category, "poor", and merge the richer and the richest into one category as "rich", thus you will have three categories for the wealth index. This will provide a much better explanation of your findings.	**Revised as commented**	**10**
2. The 2nd paragraph on Page 8: I see that you included "source of drinking water", "type of toilet facility", and "type of cooking" in your independent variable list. I suggest you exclude these variables because they have already been used to estimate the household wealth index (composite index). Using them again with the household wealth index in the model may not be statistically appropriate.	**Revised as commented**	**10**
3. The first paragraph on Page 9: You mentioned that you used a multilevel mixed-effects Poisson regression. Later on in the paragraph, you mentioned that you	**Revised as commented**	**9-10**

applied a mixed-effects logistic regression model. Kindly review and revise the entire paragraph.		
**Result**		
1. I notice a haphazard presentation of your findings. Your presentation needs to be systematic and must align with the order of arrangement of the factors in the Tables.	**Revised as commented**	**21-24**
2. Page 14: In the first paragraph, you might need to review this statement: "The ICC (Intraclass Correlation Coefficient) value from the null model revealed that only about 26.8% of the total variance in stunting among under five children is attributable to differences between clusters, while the remaining 73.2% was attributed to individual-level factors". How was the remaining attributed to individual-level factors alone, and what about the household and contextual factors?	**Revised as commented**	**20-21**
3. Page 15, 1st paragraph: This statement totally negates what is in Table 4: "The prevalence of stunting was 16% higher (aPR = 1.16, 95% CI: 1.13, 1.19) among children perceived by their mothers as having a small size at birth and 48% higher (aPR = 1.48, 95% CI: 1.43, 1.52) among those perceived as having an average size at birth."	**Revised as commented**	**All pages**
4. Page 15, 3rd paragraph: The entire paragraph needs to be rewritten because it is ambiguous, and the findings are lumped together. Kindly use a sentence to present each finding, stating the reference category for each finding.	**Revised as commented**	**21-24**
5. Table 4 has no notation for the asterisk ** (page 18)	**Revised as commented**	**24**
**Discussion section**		
1. Page 21, Paragraph 2: Please revise the grammatical structure of this statement: "Study revealed that, postnatal visit associated with child stunting. Mothers who did not attend postnatal care services were 1.03 more likely to have a stunted child compared to those who did attend postnatal care. This finding consistent with study conducted in Ethiopia (91)."	**Revised as commented**	**30**
2. The discussion lacks coherence. For instance, begin with ANC, then address the Place of delivery, followed by PNC (pages 21-22).	**Revised as commented**	**21-24**

3. Page 22, paragraph 4: "This study found that, maternal body mass index associated with stunting". "was" is missing.	**Revised as commented**	

3. Response to Journal Requirements:

**Comments**	**Author Response**	**Page number in the manuscript**
1. Please ensure that your manuscript meets PLOS ONE's style requirements, including those for file naming. The PLOS ONE style templates can be found at: **https://journals.plos.org/plosone/s/file?id=wjVg/PLOSOne_formatting_sample_main_body.pdf** and **https://journals.plos.org/plosone/s/file?id=ba62/PLOSOne_formatting_sample_title_authors_affiliations.pdf**	**Revised as commented**	**All**
2. We noticed you have some minor occurrence of overlapping text with the following previous publication(s), which needs to be addressed: **https://journals.plos.org/plosone/article?id=10.1371/journal.pone.0248637** **https://bmcpregnancychildbirth.biomedcentral.com/articles/10.1186/s12884-024-07105-7** . In your revision ensure you cite all your sources (including your own works), and quote or rephrase any duplicated text outside the methods section. Further consideration is dependent on these concerns being addressed.	**Revised as commented**	**All**
3. Please note that PLOS ONE has specific guidelines on code sharing for submissions in which author-generated code underpins the findings in the manuscript. In these cases, we expect all author-generated code to be made available without restrictions upon publication of the work. Please review our guidelines at https://journals.plos.org/plosone/s/materials-and-software-sharing#loc-sharing-code and ensure that your code is shared in a way that follows best practice and facilitates reproducibility and reuse.	**Our manuscript does not strictly require code sharing because no novel code underpins in our findings**	**All**

4. In the online submission form you indicate that your data is not available for proprietary reasons and have provided a contact point for accessing this data. Please note that your current contact point is a co-author on this manuscript. According to our Data Policy, the contact point must not be an author on the manuscript and must be an institutional contact, ideally not an individual. Please revise your data statement to a non-author institutional point of contact, such as a data access or ethics committee, and send this to us via return email. Please also include contact information for the third-party organization, and please include the full citation of where the data can be found.	**Revised as commented**	**36**
5. Please amend either the abstract on the online submission form (via Edit Submission) or the abstract in the manuscript so that they are identical.	**Revised as commented**	**2-3**
6. Your ethics statement should only appear in the Methods section of your manuscript. If your ethics statement is written in any section besides the Methods, please delete it from any other section.	**Revised as commented**	**12**
