# Supplementary material for: Prevalence of stunting and its determinants among children under five in 35 Sub-Saharan African countries (2011–2024): Insights from recent demographic health survey data using a generalized linear mixed-effects model with robust poisson regression
Source: PLoS One. 2026 Mar 13;21(3):e0344358. doi: 10.1371/journal.pone.0344358 (PMC12987452; doi:10.1371/journal.pone.0344358)
Supplement: Attachment — Submitted filename: PLoS_One_stunting.docx [file pone.0344358.s001.docx]

I want to thank the editor and the authors for the chance to review this manuscript. The subject of childhood stunting in sub-Saharan Africa is deeply important, and I appreciate the careful work that has gone into this study. It was a privilege to read and reflect on the findings, and I hope my comments will be useful as you move the paper forward.

General feedback: This paper is very well put together and speaks to a pressing public health concern. The authors draw on DHS data from 35 countries and use appropriate statistical approaches, including robust Poisson regression and mixed-effects modeling, which help account for the survey design and avoid common issues with odds ratios. The sample size is impressive (over 190,000 children), which gives the study strong statistical power and makes the findings more widely applicable. The results are presented clearly, the methods are solid, and the conclusions are well supported by the data.

Introduction

- The introduction does a good job of highlighting the global and regional burden of stunting, but it feels crowded with statistics. The numbers are useful, yet they sometimes overwhelm the main message, which should be that stunting remains a serious child health issue in sub-Saharan Africa despite global progress.
- The authors note that their study fills a gap by using pooled data from 35 countries, but this point could be made more directly. At present, it is not fully clear how this analysis differs from single-country or regional studies and what unique value it adds. Readers should come away with a stronger sense of why this pooled approach is important.
- The introduction leans heavily on prevalence estimates and less on the “why.” It would be stronger if it included more about the pathways linking maternal, household, and structural factors to stunting. This would help readers understand the reasoning behind the choice of variables and provide a clearer foundation for the study. A conceptual framework showing and visualizing pathways would be complementary.
- The section moves quickly between global, regional, and national levels. While this shows the breadth of the issue, the flow could be improved by focusing earlier and more directly on sub-Saharan Africa, since that is the real focus of the paper.
- The writing is generally clear but could benefit from light editing to improve flow and fix awkward wording. For example, phrases like “which previous study not accounted incorporated in to analysis” should be revised for clarity.

Methods

- The paper mentions that 23 variables were included, but it is not entirely clear how these were chosen. While prior studies are referenced, the rationale for why these specific variables were prioritized over others could be explained better. Without that clarity, it is hard to know whether the model might be at risk of being overloaded.
- The manuscript notes that children with missing data were excluded, but it is not clear how the missingness was assessed by the authors or whether its impact on representativeness was considered. Because this is a secondary dataset covering 35 countries, it is possible that patterns in missing data during collection shaped which cases were ultimately included. Adding a short note about this would help bring more clarity and transparency.
- The study draws on surveys from 35 countries collected over more than a decade. Since survey years and tools may vary a bit, it would be helpful to explain how this variation was handled. For example, what steps were taken to make sure that one country or survey year did not carry too much weight in the pooled results?
- The authors mention applying weights, but it is not clear whether these were adjusted for pooling across countries. Since sample sizes vary widely, larger countries may dominate the results if weights are not harmonized. DHS weights make each survey nationally representative, but when pooled they do not automatically align, so rescaling or normalizing is often needed to ensure balanced contributions.
- The authors also report AIC and BIC, but there is no discussion of whether Poisson assumptions were checked. Because stunting data are often overdispersed, ignoring this can bias standard errors and inferences. Robust Poisson regression helps, but a short note on whether overdispersion was considered would add confidence in the analysis.
- Maternal BMI could affect child nutrition, but it could also be shaped by the same household food insecurity or poverty that drives stunting. Similarly, media exposure might influence health behaviors, but access to media is itself determined by socioeconomic status. Without temporal ordering, the relationships in the model can run both ways, which means the results should be interpreted as associations rather than causal effects. Authors should acknowledge the limits of causal interpretation given the cross-sectional design.

Results

- There are places where the confidence intervals seem misordered (for example, lower bounds listed higher than upper bounds). These appear to be typos
- In the country level variation, it is not clear whether differences across countries were statistically tested or whether these patterns are being highlighted simply for descriptive interest. Greater clarity here would help.
- Some of the associations are discussed in a way that suggests causal direction (for example, maternal BMI “reduces risk”), but the results come from a cross-sectional dataset. The narrative would be strengthened by consistently framing these as associations rather than causal effects. For instance, maternal BMI is described as being “associated with stunting,” but the explanation reads as though underweight mothers cause poor nutrient transfer and overweight or obese mothers prevent stunting by having better food access. Media exposure is also presented as an association, yet the discussion suggests media itself changes behaviors and outcomes. In the same way, antenatal and postnatal care are framed as if they directly reduce stunting, with wording that children of mothers who missed visits were “more likely” to be stunted because they missed out on nutrition education.

Discussion

- Although the authors report associations in the results, parts of the discussion read as if the study demonstrates cause and effect. For example, maternal BMI, media exposure, and health service use are described in ways that imply they directly reduce or increase stunting. Because this is a cross-sectional study, the findings should be framed consistently as associations rather than causal effects.
- The discussion does not fully explore the variation across countries. While the pooled analysis is informative, it can mask important differences between contexts. Reflecting on why some countries deviate from the overall pattern, and what that means for interventions, would add more depth.
- Policy implications are mentioned but not clearly prioritized. For instance, maternal education, ANC/PNC attendance, and socioeconomic status are all important, but it would help to distinguish which determinants are more feasible targets for immediate policy action and which require longer-term structural investments.
- The study is cited alongside some prior work, but the discussion could more strongly situate the findings within the broader literature on child stunting in sub-Saharan Africa. How do these results compare with evidence from longitudinal studies or interventions? This would give readers a clearer sense of what is confirmed, what is new, and what remains uncertain.
- The limitations section is rather brief. Issues such as missing data, the cross-sectional design of DHS surveys, and the possibility of residual confounding deserve more explicit acknowledgment. Without this, readers may overestimate the strength of the conclusions.
- Much of the interpretation centers on maternal and household factors. Bringing in structural determinants such as poverty, food systems, governance, or conflict would provide a fuller picture. Without this perspective, the discussion risks placing too much emphasis on individual behavior change as the solution. Think structural and systems-level changes.
