# Supplementary material for: Prevalence of stunting and its determinants among children under five in 35 Sub-Saharan African countries (2011–2024): Insights from recent demographic health survey data using a generalized linear mixed-effects model with robust poisson regression
Source: PLoS One. 2026 Mar 13;21(3):e0344358. doi: 10.1371/journal.pone.0344358 (PMC12987452; doi:10.1371/journal.pone.0344358)
Supplement: Attachment — Submitted filename: Response to Reviewers Stunting Final.docx [file pone.0344358.s005.docx]

# **Author response to reviewer**

#### Manuscript ID: PONE-D-25-15249R1

#### Manuscript Title: Prevalence of Stunting and its Determinants Among Children Under Five in 35 Sub-Saharan African Countries (2011–2024): Insights from recent Demographic Health Survey Data Using a Generalized Linear Mixed-Effects Model with Robust Poisson Regression

#### 1. Response to Reviewer 1

| **Comments** | **Author Response** | **Page number in the manuscript** |
| --- | --- | --- |
| 1. Thank you for submitting your manuscript to PLOS ONE. After careful consideration, we feel that it has merit but does not fully meet PLOS ONE’s publication criteria as it currently stands. Therefore, we invite you to submit a revised version of the manuscript that addresses the points raised during the review process. | **Thank you very much for your encouragement and constructive comments.** |  |
| **Typographic and grammatical issues** | | |
| 1. After a careful review of the revised manuscript, a few typographic and grammatical issues were identified and need to be addressed. They are highlighted with comments or suggestions in the attached document for your action. They are located in the following lines: 49-51; 61-64; 126; 306-307 (Table 2); 401-403; 431-432; 470;495; 532-533;551-552; 554-556; 560-561; 566-567;631-632; 643; 707; 708; 709; 711, 719; 726, & 729. | **All are revised as commented.** |  |

2. **Response to Journal Requirements:**

| **Comments** | **Author Response** | **Page number in the manuscript** |
| --- | --- | --- |
| 1. If the reviewer comments include a recommendation to cite specific previously published works, please review and evaluate these publications to determine whether they are relevant and should be cited. There is no requirement to cite these works unless the editor has indicated otherwise. | **Evaluated as commented** |  |
| 2. Please review your reference list to ensure that it is complete and correct. If you have cited papers that have been retracted, please include the rationale for doing so in the manuscript text, or remove these references and replace them with relevant current references. Any changes to the reference list should be mentioned in the rebuttal letter that accompanies your revised manuscript. If you need to cite a retracted article, indicate the article’s retracted status in the References list and also include a citation and full reference for the retraction notice. | **No retracted paper has been cited as much as we evaluated** |  |
| 1. To ensure your figures meet our technical requirements, please review our figure guidelines: https://journals.plos.org/plosone/s/figures 2. You may also use PLOS’s free figure tool, NAAS, to help you prepare publication quality figures: https://journals.plos.org/plosone/s/figures#loc-tools-for-figure-preparation. 3. NAAS will assess whether your figures meet our technical requirements by comparing each figure against our figure specifications.. | **Revised as commented** | **All** |
